# Looking for peptides from rice starch processing by-product: Bioreactor production, anti-tyrosinase and anti-inflammatory activity, and *in silico* putative taste assessment

**DOI:** 10.3389/fpls.2022.929918

**Published:** 2022-07-15

**Authors:** Maura Ferri, Tullia Tedeschi, Barbara Prandi, Elisa Michelini, Maria Maddalena Calabretta, Elena Babini, Jürgen Graen-Heedfeld, Karlheinz Bretz, Noura Raddadi, Andrea Gianotti, Matteo Lamborghini, Annalisa Tassoni

**Affiliations:** ^1^Department of Biological, Geological and Environmental Sciences, University of Bologna, Bologna, Italy; ^2^Department of Food and Drug, University of Parma, Parma, Italy; ^3^Department of Chemistry “G. Ciamician”, University of Bologna, Bologna, Italy; ^4^Department of Agricultural and Food Sciences, University of Bologna, Cesena, Italy; ^5^Interdepartmental Centre of Agri-Food Industrial Research, University of Bologna, Cesena, Italy; ^6^Fraunhofer Institute for Environmental, Safety, and Energy Technology, Oberhausen, Germany; ^7^Department of Civil, Chemical, Environmental and Materials Engineering, University of Bologna, Bologna, Italy; ^8^Carminia snc, Bologna, Italy

**Keywords:** anti-inflammatory activity, antioxidants, anti-tyrosinase activity, bioactive peptides, bitter taste, rice by-product

## Abstract

One of the major challenges for the modern society, is the development of a sustainable economy also aiming at the valorization of agro-industrial by-products in conjunction with at a significant reduction of generated residues from farm to retail. In this context, the present study demonstrates a biotechnological approach to yield bioactive peptides from a protein fraction obtained as a by-product of the rice starch production. Enzymatic hydrolysis, with the commercial proteases Alcalase and Protamex, were optimized in bioreactor up to 2 L of volume. The two best digestates, selected with respect to peptide release and extract antioxidant capacity, were further fractionated (cut-offs of 10, 5, and 1 kDa) *via* cross-flow filtration. Amino acid composition indicated that most of the fractions showed positive nutritional characteristics, but a putative bitter taste. A fraction obtained with Alcalase enzyme (retentate 8 kDa) exerted anti-inflammatory potential, while the smaller molecular weight fractions (retentate 1–5 kDa and permeate < 1 kDa) were more active in tyrosinase inhibition. The latter were further sub-fractionated by size-exclusion chromatography. From the 15 most anti-tyrosinase sub-fractions, 365 peptide sequences were identified *via* liquid chromatography coupled with high resolution mass spectrometry. The present data support the possible exploitation of bioactive peptide from rice starch by-product as ingredients into food, nutraceutical, pharmaceutical, and cosmetic formulations.

## Introduction

Modern society faces several challenges regarding the reduction of the overall amount of by-products and wastes. In recent years, the scientific and applied research efforts focusing on by-product valorization as source of valuable compounds, largely increased.

Rice is one of the main staple crops for humans, with a production around 756 Mton in the world and 2.9 Mton in European Union in 2020 (FAOSTAT, 2020). Agricultural and industrial rice supply and processing chains produce different type of by-products, representing worldwide a large economic and environmental issue (Spaggiari et al., 2021). Rice bran and broken rice are the two main by-products but still underestimated and not yet fully exploited. In general, rice by-products have been indicated as a renewable and cost-effective source of antioxidant and bioactive compounds (e.g., arabinoxylans, phenolic acids, γ-oryzanol, tocopherols, and group B vitamins) and peptides, and several recovery and valorization strategies have been set up in recent years (Rani et al., 2018; Görgüç et al., 2020; Spaggiari et al., 2021).

In particular, the most relevant valorization route for the broken rice by-product (composed by 80% starch and 8% proteins) is actually starch production (Shih, 2012). During this process, the protein fraction of the raw material is lost in a residual fraction (approximately 60–85% of by-product in the process of rice starch), which is currently discarded, or underutilized as animal feed, given the poor protein solubility (Ferri et al., 2017; Rani et al., 2018). The valorization of such rice protein by-product is, therefore, a challenge in view of residual feedstock valorization as source of useful compounds. In fact, particular interest was recently addressed to rice proteins, most of which are storage proteins which were classified in four groups according to their solubility: albumins (water soluble), globulins (salt soluble), glutelins (alkali/acid soluble), and prolamins (alcohol soluble) (Rani et al., 2018). Rice proteins were reported to have high-quality, good digestibility, various biological activities, and hypo-allergenicity (Rani et al., 2018; Görgüç et al., 2020). These properties are maintained or even enhanced in peptides derived from such proteins. In fact, the enzymatic hydrolysis occurring during digestion, can release fragments which were inactive within the parent sequences. These peptides are usually 2–20 amino acids long and their functional properties and biological activities depends on their amino acid composition, conformation, and hydrophobicity (Rani et al., 2018; Görgüç et al., 2020). Moreover, low molecular weight peptides can be easily absorbed in the gastrointestinal tract and cardiovascular circulation system and finally exhibit physiological-regulating properties (Rani et al., 2018). Some of the beneficial properties ascribed to rice peptides are antioxidant, anti-hypertensive, anti-tyrosinase, anti-inflammatory, and antimicrobial activities (Ferri et al., 2017; Babini et al., 2020; Görgüç et al., 2020). Nevertheless, a common shortcoming on the use of bioactive peptides as nutraceutical or food ingredients is their bitter taste due to the hydrophobic amino acid content (Görgüç et al., 2020; Selamassakul et al., 2020).

Actually, the possible valorization routes of rice starch processing protein by-product are still largely unexplored with only few studies applying biotechnological approaches (Dei Piu et al., 2014; Ferri et al., 2017; Babini et al., 2020). This by-product has been fermented by Lactic Acid Bacteria (LAB) strains selected with the aim to release bioactive peptides, the process was scaled up to 1 L volume and several bioactive peptides were sequenced (Dei Piu et al., 2014; Babini et al., 2020). Additionally, promising results were obtained in small laboratory scale by treating the same residue with commercial proteolytic enzymes (Dei Piu et al., 2014; Ferri et al., 2017), leading to the need of validating the hydrolysis methodology in a larger scale.

The present study evaluated the possibility to exploit peptides, obtained from a protein-rich rice starch processing by-product, as ingredients in food, nutraceutical, pharmaceutical or cosmetic preparations. Key enzymatic hydrolysis processing parameters were optimized in small-scale bioreactor (1 and 2 L) and total digestates were fractionated by means of cross-flow filtration and sub-fractionated by size exclusion chromatography. Valuable bioactivities (antioxidant, anti-tyrosinase, anti-inflammatory properties) and/or nutritional/structural characteristics were analyzed in the different molecular weight peptide fractions. By means of a predictive *in silico* analysis, taste properties were estimated on the basis of amino acids contents.

## Materials and methods

### Rice starch by-product

The rice by-product was supplied from a local company (Carminia snc, Bologna, Italy) and was derived from the processing of Italian broken rice (composed by a mix of several varieties of *Oryza japonica* L.) (Ferri et al., 2017). The same batch of by-product was used to perform all experimental trials. The feedstock consisted of a water-based slurry with solid particles in suspension, pH 6.5 and protein content of 6.6 ± 1.5 g BSA eq/L (g of bovine serum albumin equivalent per liter), of which 0.2 g BSA eq/L in the liquid phase, according to Lowry assay (Lowry et al., 1951).

### Protease treatments

Two commercial proteases, Alcalase^®^ 2.4 L (alc, Novozymes A/S, Denmark) and Protamex^®^ (pro, Novozymes A/S, Denmark), were previously selected (Ferri et al., 2017). Twelve different hydrolysis processes were performed in 3 L bioreactor (BIOSTAT^®^ B, Sartorius Stedim Italy S.r.l., Antella-Bagno a Ripoli, FI, Italy) equipped with two rushton turbines, by testing six different conditions for each enzyme (Table 1): three enzyme/substrate ratios (E/S; 0.5, 0.2, and 0.05 U enzyme/g protein), two reaction temperatures (60 and 55°C) and two working volumes (1 and 2 L). Temperature, pH (alc pH 7.0, pro pH 8.0), and stirring rate (300 rpm) were kept constant. No aeration was supplied. No foam formation was observed in all the tested digestion scales, therefore no anti-foam addition was required. Hydrolyses were carried out for 120 min and sample aliquots were collected every 30 min. Each intermediate sample and the final total digestates (TD) were boiled 10 min for enzyme inactivation and then stored at −20°C until further analyses.

**TABLE 1:** Bioreactor processing conditions applied during enzymatic digestion up to 120 min of incubation with both Alcalase and Protamex.

Digestion condition code	E/S ratio (Unit enzyme/g protein)	Temperature (°C)	Volume (L)
1	0.5	60	1
2	0.5	55	1
3	0.2	55	1
4	0.05	55	1
5	0.5	55	2
6	0.2	55	2

E/S, enzyme/substrate ratio.

### Fractionation and sub-fractionation processes

The TD obtained from both alc and pro digestion processes in condition number 5 (Table 1) were centrifuged (10 min, 4500 g, room temperature) and the liquid supernatants were fractionated by cross-flow filtration as reported by Ferri et al. (2017), to isolate peptide samples having different molecular weight (MW) ranges. The obtained fractions were named as follows: retentates (R) R0.2μm (particle size > 0.2 μm), R8 (0.2 μm > MW > 8 kDa), R5 (8 kDa > MW > 5 kDa) and R1 (5 kDa > MW > 1 kDa) and permeate (P) P1 (1 kDa > MW). Each fraction was divided in two aliquots: the first was directly frozen and stored at −20°C, while the second (25 mL) was lyophilized using an Alpha 2–4 LSCplus with Lyocube system (Martin Christ Gefriertrockungsanlagen GmbH, Osterode am Harz, Germany) with the manufacturers drying protocol. Lyophilization was not performed on the original by-product (ND, not digested) and on the final TD supernatants.

Fractions R1 and P1 (lyophilized powders resuspended in MilliQ water), containing the smallest MW peptides, were further sub-fractionated by size exclusion chromatography using an AKTA FPLC system (GE Healthcare, Chicago, IL, United States) equipped with a TSK gel G2500PWXL column (Tosoh Bioscience, Tokyo, Japan). Aliquots of 25 μL of filtered samples were loaded on the column, equilibrated with phosphate buffer 100 mM, KCl 25 mM, pH 7.4, at a flow rate of 0.5 mL/min. Samples with different retention time (RT) were manually collected.

### Protein and amino acid contents assessment and predictive taste properties evaluation

Hydrolyzed samples were centrifuged (10 min, 4500 g, room temperature) and the liquid supernatants were analyzed for protein content and bioactivities. Liquid peptide fractions were directly analyzed, while lyophilized powders were dissolved in MilliQ water at the same g/L ratio.

Protein content was quantified in the initial rice by-product, in all the samples collected during hydrolysis process and in all peptide fractions and sub-fractions, by Lowry assay (Lowry et al., 1951), and results were expressed as g of bovine serum albumin equivalent per liter (g BSA eq/L).

Protein and peptide molecular mass distributions were analyzed by mono-dimensional 20% w/v acrylamide SDS-PAGE (Sodium Dodecyl Sulfate-Polyacrylamide Gel Electrophoresis) (Laemmli, 1970) in the not hydrolyzed by-product, TD and R and P fractions. In the same samples, free amino acids were quantified by HPLC by means of Waters AccQ Tag Amino Acid Analysis Method kit (Waters Corporation, Milford, MA, United States) following manufacturer’s instructions. Amino acid derivatization (Cohen and Michaud, 1993) with AccQ Tag reagents and analyses were carried out as reported by Monari et al. (2019). Results were reported as μmol/L. Free amino acid data were elaborated for their predicted contribution to taste properties (Selamassakul et al., 2016) and nutritional/structural characteristics (Selamassakul et al., 2020). In brief, amino acid contents were grouped as follows: bitter (GLY, HIS, PRO, VAL, MET, ILE, LEU, PHE), umami (ASP, GLU), sweet (SER, ARG, ALA, THR) and salty (CYS) tastes, tasteless (TYR, LYS); total essential amino acid (EAA: HIS, THR, TYR, VAL, LYS, ILE, LEU, PHE), total branched-chain amino acid (BCAA: ILE, LEU, VAL), total hydrophobic amino acid (HAA: ALA, ILE, LEU, MET, PHE, VAL, PRO, GLY).

### Biological activities assessment

Antioxidant activity was assessed *via* ABTS assay (2,2-azino-bis-3-ethylbenzothiazoline-6-sulfonic acid) (Ferri et al., 2013) and results expressed as ascorbic acid equivalents/L (g AA eq/L) by means of a dose-response calibration curve (between 0 and 2 μg of AA). This activity was chosen as reference in following the bioactive peptide release and, therefore, was measured in the initial rice by-product, in all the samples collected during hydrolysis process and in all peptide fractions.

Anti-tyrosinase activity was assessed by a tyrosinase inhibition assay (Ferri et al., 2017) and results expressed as kojic acid (a well-known tyrosinase inhibitor) equivalents/L (g KA eq/L) (calibration curve between 1 and 10 μg of KA).

Anti-inflammatory activity was performed with a dual-color reporter assay based on human embryonic kidney HEK293 cells as reported by Ferri et al. (2017). Bioluminescent (BL) and fluorescent (FL) signals were corrected according to cell viability.

### Peptide identification

Peptides were identified by means of reverse phase liquid chromatography coupled with high resolution mass spectrometry. The μHPLC-LTQ-OrbiTRAP (μHPLC by Dionex Ultimate 3000, Sunnyvale, CA, United States, coupled to Orbitrap LTQ XL mass spectrometer by Thermo Scientific, Waltham, MA, United States). Analysis was performed as described in Prandi et al. (2019). Briefly, Eluent A was water + 0.2% formic acid, eluent B was acetonitrile with 0.2% formic acid. Sample loading conditions were: enrichment cartridge: μ-Precolumn Cartridge, Acclaim PepMap100 C18 5 μm, 100 Å, 300 μm × 5 mm, loading flow: 30 μL/min, 50% eluent A and 50% eluent B. Sample elution condition were: column: Phenomenex Jupiter 4 μm Proteo 90Å 150 mm × 0.3 mm, column temperature: 35°C, gradient: 0–4 min 10% B, 4–60 min linear from 10% B to 50% B, 60–62 min from 50 to 95% B, 62–72 min 95% B (column washing), 72–73 min from 95% B to 10% B, 73–82 min 10% B (column equilibration). HRMS acquisition was performed through 5 subsequent events: event 1: full scan acquisition from 250 to 2000 *m/z* in high resolution mode (resolution at 400 *m/z* = 30,000); events from 2 to 5: data dependent scan, at each cycle the four most intense ions (with charge z > 1 and with a minimum signal of 500 counts) identified in event 1 are fragmented. The same ion (tolerance 10 ppm and isolation window 2 *m/z*) can be observed for a maximum of 2 cycles, and then it is automatically inserted in the exclusion list for a maximum time of 20 s. Fragmentation is performed in the linear trap of the instrument in CID mode with collision energy of 35.

Data processing was performed using the software Peaks Studio (Bioinformatics Solutions Inc., Waterloo, ON, Canada). Searches were run both on the specific database *Oryza sativa* subsp. japonica (Rice). A minimum threshold of −10lgP of 20 was fixed to select only high confidence identifications.

### Statistical analysis

All the assays and the analyses were performed at least in triplicate in two technical replicates each and the results are expressed as the mean (*n* = 3) ± SD. Statistically significant differences among samples were analyzed by using one-way ANOVA (Analysis of Variance) test followed by *post hoc* corrected two-tailed Tukey test assuming equal variance (*p* < 0.05).

## Results

### Peptide production processes

**FIGURE 1:**
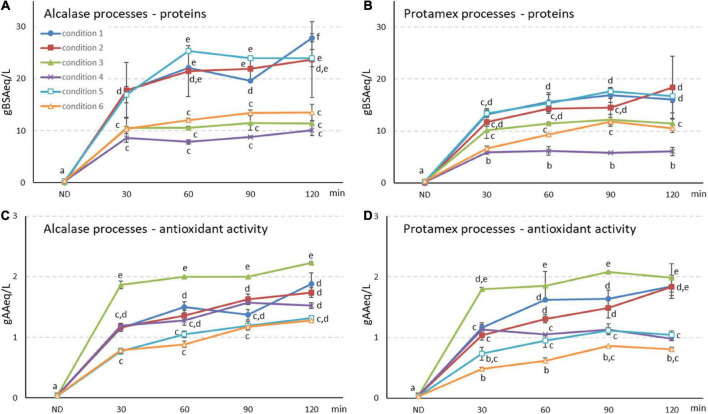
**(A,B)** Protein and **(C,D)** antioxidant activity quantifications in **(A,C)** Alcalase and **(B,D)** Protamex processes at in-creasing incubation time (up to 120 min) and in 1 and 2 L bioreactor working volume. Conditions 1–6 are reported in Table 1. Protein results are expressed as g of bovine serum albumin equivalent (g BSA eq/L) and antioxidant activity as g of ascorbic acid per liter (g AA eq/L) ± SD (*n* = 3). ND, not digested initial by-product. Different letters indicate a statistically significant difference (one-way ANOVA test followed by *post hoc* corrected two-tailed Tukey test, *p* < 0.05) between the same type of data (proteins, antioxidant activity), from the lowest (a) to the highest (e).

The hydrolysis of the protein feedstock, obtained from a rice starch industry, by Alcalase (alc) and Protamex (pro) commercial proteases was optimized in bioreactor scale. Six different conditions were tested (Table 1) and 3 key parameters were considered: enzyme/substrate ratio (E/S), temperature and volume. Peptide release in the liquid phase was followed at increasing times (Figures 1A,B). All liquid supernatant samples were analyzed for protein content (Figures 1A,B), peptide molecular weight (MW) distribution (Supplementary Figure 1) and antioxidant activity (Figures 1C,D), to assess digestion yield and final product profile. All the tested conditions with both enzymes were able to release peptides within 2 h, with a fast hydrolysis in the first 30 min and a further trend depending on the conditions, sometimes reaching a plateau (i.e., conditions 3 and 4) (Figures 1A,B). At the same time, the antioxidant activity of the liquid phase largely increased with similar trends (Figures 1C,D).

The initial water-based slurry feedstock from starch processing (ND, not digested) contained only 0.22 g BSA eq/L in the liquid phase. By applying digestion condition 1 (Table 1), peptides were released with final yields of 27.9 and 16.1 g BSA eq/L in alc and pro processes (Figures 1A,B, 120 min samples). Total digestate (TD) antioxidant activity was similar in both alc and pro bioreactor processes (1.88 and 1.85 g AA eq/L, respectively) (Figures 1C,D, 120 min samples).

Other three different conditions were tested for process optimization (Table 1, conditions 2–4). Temperature reduction did not significantly affect peptide yield nor antioxidant activity (Figure 1, condition 2), while enzyme units decrease determined lower peptide yields both for alc and pro (0.5 U enz/g prot > 0.2 U enz/g prot > 0.05 U enz/g prot) (Figures 1A–D, Conditions 3 and 4). The highest antioxidant activity was obtained for both enzymes with the intermediate E/S (condition 3, 0.2 U enz/g prot, Figures 1C,D).

The two best performing processes were conditions 2 and 3 (0.5 and 0.2 U enz/g prot, 55°C, 1 L working volume), therefore they were repeated with a 2 L reaction volume (conditions 5 and 6, respectively). A quicker peptide release was observed but final yields similar to 1 L scale were reached (Figures 1A,B). On the other hand, antioxidant activity seemed to be negatively affected by the reaction volume increase (Figures 1C,D). Condition 5 led to the highest TD peptides content in 2 L scale, with a final yield of 24.0 g BSA eq/L.

The complete protein digestion and the presence of small peptides (size smaller than 12 kDa) in supernatant samples was verified by mono-dimensional SDS-PAGE analyses (Supplementary Figure 1). In fact, gel electrophoresis separation demonstrated that the peptides, almost absent in ND, were present in the digestates as consequence of the complete digestion by alc and pro of the higher MW proteins, initially detectable in ND not centrifuged samples.

### Peptide fractions characterization

The liquid total digestate (TD) supernatants (120 min samples) obtained from both alc and pro processes with condition 5 (Figure 1), were fractionated by cross-flow filtration with the aim of isolating peptide fractions with different MW. Condition 5 was selected thanks to its high protein content and peptide release and, at least in pro hydrolysis, to a higher antioxidant activity in TD respect to condition 6 (Figure 1).

**FIGURE 2:**
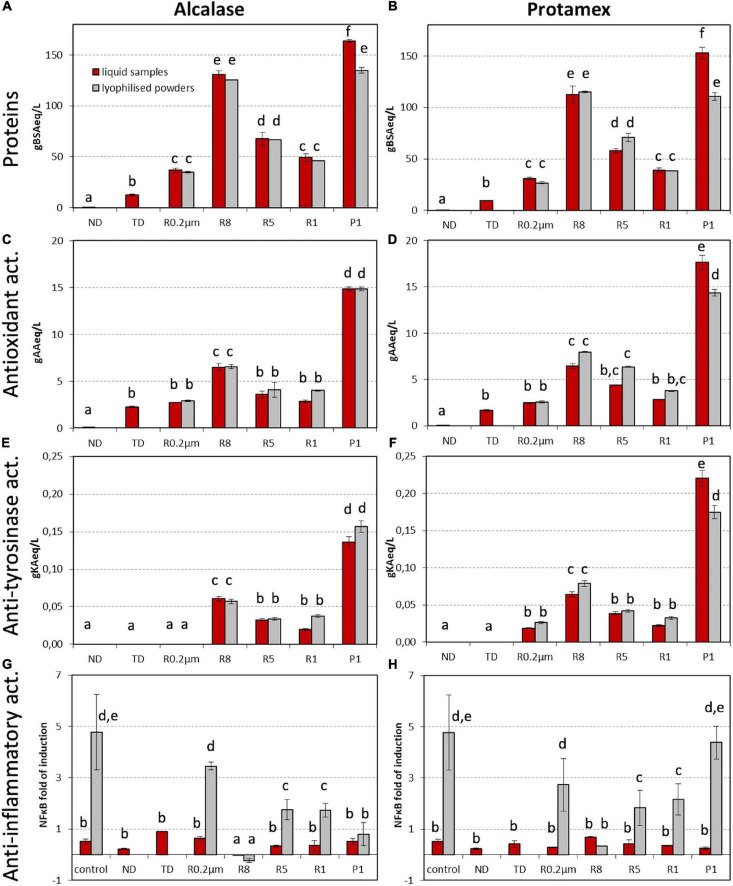
**(A,B)** Protein content, **(C,D)** antioxidant, **(E,F)** anti-tyrosinase, and **(G,H)** anti-inflammatory activities in **(A,C,E,G)** Alcalase and **(B,D,F,H)** Protamex processes at 55°C, 120 min, 0.5 U enz/g proteins, 2 L reaction volume (condition 5). Data of liquid samples and of related lyophilized powders are reported; lyophilization was not performed on ND and TD. Fractions were named as follows: ND, not digested initial by-product; TD, total digestate condition 5; retentates of 0.2 μm (R 0.2 μm), 8 kDa (R8), 5 kDa (R5), and 1 kDa (R1), and 1 kDa permeate (P1). Spectrophotometric results are expressed as g of standard compound (BSA, bovine serum albumin; AA, ascorbic acid; KA, kojic acid) equivalent per liter. Cell-based assay results are expressed as fold of NFκB induction in presence of TNFα; results are corrected according to cell viability. Data represent the mean ± SD (*n* = 3). Different letters indicate a statistically significant difference (one-way ANOVA test followed by *post hoc* corrected two-tailed Tukey test, *p* < 0.05) between the same type of data (proteins, antioxidant, anti-tyrosinase, and anti-inflammatory activities), from the lowest (a) to the highest (f).

Protein (Figures 2A,B) and amino acid (Supplementary Table 1) contents were assessed in not digested (ND) control, TD, retentates (R) of 0.2 μm (R 0.2 μm), 8 kDa (R8), 5 kDa (R5) and 1 kDa (R1) membranes and 1 kDa permeate (P1). The same samples and MW fractions were analyzed for antioxidant (Figures 2C,D), anti-tyrosinase (Figures 2E,F), and anti-inflammatory (Figures 2G,H) activities.

The ND liquid phase only contained proteins in trace. Peptides were released during the hydrolysis (total content in TD sample) and they were further fractionated and concentrated during the cross-flow membrane fractionation process (Figures 2A,B). After both enzyme treatments, fractions R8 and P1 showed the highest protein concentrations (above 100 g/L; Figures 2A,B).

To investigate whether the stabilization by lyophilization could have affected the sample performance, the liquid supernatants were divided into two parts, one of which was powdered by lyophilization. Protein concentrations were quantified in the liquid fractions, then powders were dissolved in water at the same g/L ratio as original liquid fractions, and protein contents were quantified again. All analytical data obtained from the liquid samples were compared with those of the correspondent powdered and resuspended samples (Figure 2). Results showed that fraction powder weight corresponded to protein weight (Figures 2A,B, red and gray bars comparison). The only exception was P1 (from both alc and pro digestions) in which the protein weight was 18–28% lower than powder weight.

Antioxidant capacity reflected the detected amounts of protein contents with P1 and R8 from both enzyme treatments being the most active fractions (Figures 2A–D). As expected, TD and all isolated fractions showed higher activity with respect to ND. Pro enzyme produced slightly more active peptides than alc, mainly in R8 and R5 fractions. Maximum bioactivity levels were detained by P1 samples (14.9 and 17.7 g AA eq/L from alc and pro digestions, respectively) (Figures 2C,D).

*In vitro* anti-tyrosinase activity was higher in lower MW fractions and in pro than in alc fractions. No activity was measured in ND, TD and alc R 0.2 μm (Figures 2E,F). Anti-inflammatory activity was assessed by a dual-color reporter bioluminescent (BL) assay in HEK293 cell system. Only alc R8 (both in the powder and liquid form) was able to reduce NFkB fold of induction in the presence of the pro-inflammatory cytokine TNFα (Figures 2G,H), indicating a possible anti-inflammatory action.

Lyophilized fractions exerted the same levels of antioxidant and anti-tyrosinase activities of liquid samples, with the only exception of pro P1 (Figures 2C–F). Moreover, lyophilization seemed to increase 21.3-times alc R8 anti-inflammatory activity (Figures 2G,H).

### Amino acid profile and *in silico* nutritional characteristics and peptide fractions predicted taste

Free amino acids were quantified in ND by-product, TD (condition 5) and peptide fraction samples from alc and pro treatments, in liquid and lyophilized forms. Detailed data are reported in Supplementary Table 1.

**FIGURE 3:**
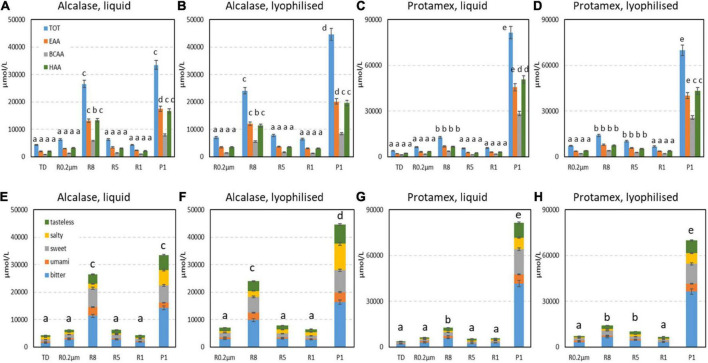
Elaboration of free amino acid contents in total digestate (TD condition 5) and peptide fraction samples from **(A,B,E,F)** Alcalase to **(C,D,G,H)** Protamex treatments, in liquid and lyophilized forms. **(A–D)** Predicted nutritional/structural characteristics and **(E–H)** taste property contributions were quantified in μmol/L, by elaboration of data reported in Supplementary Table 1. TOT, total amino acid content; EAA, total essential amino acid; BCAA, total branched-chain amino acid; HAA, total hydrophobic amino acid. Data represent the mean ± SD (*n* = 3). Different letters indicate a statistically significant difference (one-way ANOVA test followed by *post hoc* corrected two-tailed Tukey test, *p* < 0.05) between the same type of data (**A–D**: TOT, EAA, BCAA, HAA; **E–H**: total levels), from the lowest (a) to the highest (e).

Free amino acids increased in TD samples (on average 59-fold higher) when compared with ND (Supplementary Table 1). All hydrolysates showed high amounts of free amino acids, and fractions containing highest concentrations were P1 (on average 39000 and 76000 mmol/L in alc and pro samples, respectively), followed by R8 (on average 25000 and 13000 mmol/L in alc and pro samples, respectively). Amino acids were grouped according to particular characteristics: essential (EAA), branched-chain (BCAA), hydrophobic (HAA) amino acids (Figures 3A–D). All samples were rich in EAA (Figures 3A–D), that represent on average 50 and 55% of total free amino acids (TOT) in alc and pro fractions respectively. In particular, the most concentrated EAA were VAL, MET, LYS, ILE, LEU, and PHE (Supplementary Table 1). BCAA were on average 22 and 30% and HAA were on average 50–55% of TOT in alc and pro fractions, respectively (Figures 3A–D).

*In silico* flavor prediction was also performed and the tasteless, salty, sweet, umami and bitter taste contributions were analyzed for each fraction on the base of amino acids content (Figures 3E–H). Bitter flavor was predicted as the most abundant type of taste in all fractions, while umami taste amino acid contribution was on average 6.2-times lower that bitter (Supplementary Table 1; Figures 3E–H).

### Sub-fractionation and anti-tyrosinase activity analysis

**FIGURE 4:**
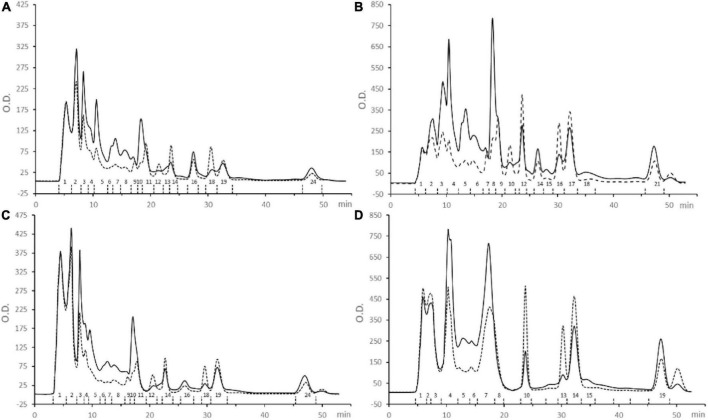
Sub-fractionation of samples **(A)** Alcalase R1, **(B)** Alcalase P1, **(C)** Protamex R1, and **(D)** Protamex P1, by FPLC size exclusion chromatography. Sub-fractions are indicated by numbers. Continuous line: absorbance at 280 nm; dashed line: absorbance at 254 nm.

Fractions containing the smallest MW peptides (R1 and P1) were further sub-fractionated by FPLC size exclusion chromatography to obtain peptide sub-fractions with reduced MW range (Figure 4). Sub-fractions were numbered sequentially from highest to lowest MW peptides [i.e., from the lowest to the highest retention time (RT)] and were analyzed for protein content and anti-tyrosinase activity (Table 2).

**TABLE 2:** Protein content (g BSA eq/L) and anti-tyrosinase activity (mg KA eq/g prot) in peptide sub-fractions.

Alcalase R1			Alcalase P1			Protamex R1			Protamex P1		
Sf n.	Protein content (g BSA eq/L)	Anti-tyrosinase activity (mg KA eq/g prot)	Sf n.	Protein content (g BSA eq/L)	Anti-tyrosinase activity (mg KA eq/g prot)	Sf n.	Protein content (g BSA eq/L)	Anti-tyrosinase activity (mg KA eq/g prot)	Sf n.	Protein content (g BSA eq/L)	Anti-tyrosinase activity (mg KA eq/g prot)
1	3.92 ± 0.10^g^	36.8 ± 4.1^c^	1	3.88 ± 0.23^g^	27.2 ± 2.7^c^	1	3.80 ± 0.35^g^	39.3 ± 6.6^c^	1	4.01 ± 0.04^g^	32.4 ± 1.9^c^
2	5.18 ± 0.04^h^	26.4 ± 2.7^b^	2	14.96 ± 0.71^j^	14.5 ± 1.5^a^	2	5.55 ± 0.67^h^	32.64 ± 4.3^b,c^	2 + 3	14.18 ± 0.97^j^	14.4 ± 0.3^a^
3	3.17 ± 0.10^e^	29.3 ± 12.3^b,c^	3	12.27 ± 0.84^j^	10.7 ± 1.1^a^	3	3.45 ± 0.06^f^	27.4 ± 4.9^b,c^	4	17.93 ± 0.72^k^	14.6 ± 3.5^a^
4	1.39 ± 0.04^d^	67.2 ± 10.3^d^	4	9.94 ± 1.27^i,j^	12.4 ± 1.2^a^	4	1.19 ± 0.09^c^	63.4 ± 7.7^d^	5	4.34 ± 0.40^g,h^	37.3 ± 5.9^c^
5	2.81 ± 0.20^e^	48.0 ± 14.9^c,d^	5	5.64 ± 0.55^h^	28.6 ± 2.9^b,c^	5	2.37 ± 0.30^e^	58.3 ± 5.5^d^	6	3.58 ± 0.05^f^	39.5 ± 3.8^c^
6	0.87 ± 0.09^c^	67.4 ± 3.3^d^	6	3.57 ± 0.09^f^	51.2 ± 5.1^d^	6 + 7	1.42 ± 0.04^d^	83.1 ± 10.8^d,f^	7 + 8	8.12 ± 0.22^i^	58.9 ± 11.2^d^
7	1.16 ± 0.08^c^	73.8 ± 22.0^d^	7	1.05 ± 0.06^c^	89.7 ± 9.0^e^	8	0.81 ± 0.09^c^	122.2 ± 7.7^f^	**10**	**0.18 ± 0.02** ^a,b^	**748.1 ± 18.7** ^i^
8	0.77 ± 0.05^c^	124.7 ± 39.2^f^	8	3.44 ± 0.02^f^	34.0 ± 3.3^c^	9	0.27 ± 0.04^b^	266.0 ± 51.0^h^	**13**	**0.26 ± 0.10** ^a,b^	**523.1 ± 58.5** ^i^
9	0.31 ± 0.09^b^	287.4 ± 16.3^h^	9	0.91 ± 0.09^c^	177.6 ± 17.8^g^	10 + 11	1.22 ± 0.01^d^	248.1 ± 11.6^g,h^	14	0.53 ± 0.16^b^	281.6 ± 15.2^h^
10	0.78 ± 0.03^c^	155.6 ± 35.4^f^	10	0.77 ± 0.07^c^	160.0 ± 16.0^f^	**12**	**0.09 ± 0.01** ^a^	**1161.5 ± 146.5** ^j^	15	0.72 ± 0.13^c^	201.1 ± 11.7^g^
11	0.44 ± 0.08^b^	282.9 ± 98.9^h^	12	1.24 ± 0.24^d^	113.5 ± 11.4^e,f^	**14**	**0.05 ± 0.01** ^a^	**1675.8 ± 171.8** ^j^	19	0.53 ± 0.07^b^	169.5 ± 3.8^g^
**12**	**0.18 ± 0.02** ^a,b^	**555.1 ± 200.7** ^i^	14	0.50 ± 0.05^b^	199.3 ± 20.0^g^	**16**	**0.08 ± 0.01** ^a^	**1187.9 ± 175.5** ^j^			
**13**	**0.07 ± 0.01** ^a^	**764.8 ± 277.6** ^i^	15	0.38 ± 0.4^b^	260.0 ± 25.9^h^	**18**	**0.05 ± 0.01** ^a,b^	**1896.5 ± 237.5** ^j^			
**14**	**0.19 ± 0.02** ^a,b^	**398.8 ± 94.4** ^h,i^	16	0.77 ± 0.08^c^	186.1 ± 18.6^g^	**19**	**0.17 ± 0.02** ^a^	**771.4 ± 46.6** ^i^			
**16**	**0.13 ± 0.01** ^a^	**669.4 ± 168.6** ^i^	17	0.71 ± 0.07^c^	237.5 ± 23.8^h^	**24**	**0.14 ± 0.01**	**633.6 ± 33.1** ^i^			
**18**	**0.13 ± 0.01** ^a^	**698.1 ± 179.7** ^i^	18	0.51 ± 0.05^b^	253.0 ± 25.3^h^						
**19**	**0.24 ± 0.02** ^b^	**564.6 ± 71.7** ^i^	21	0.43 ± 0.04^b^	297.4 ± 29.7^h^						
**24**	**0.07 ± 0.01** ^a^	**1364.4 ± 202.6** ^j^									

Sub-fractions in bold were subjected to peptide identification. Different letters indicate a statistically significant difference (one-way ANOVA test followed by post hoc corrected two-tailed Tukey test, p < 0.05) between the same type of data (protein content or anti-tyrosinase activity), from the lowest (a) to the highest (k). Data are the mean ± SD (n = 3). Sf n., sub-fraction number; BSA, bovine serum albumin; KA, kojic acid.

The highest anti-tyrosinase capacity was detected in sub-fraction n.18 from Protamex R1 (1896.5 mg KA eq/g prot) and 15 sub-fractions had an activity higher than 500 mg KA eq/g prot. In general, anti-tyrosinase activity increased with decreasing MW of the peptide sub-fractions (Table 2).

### Peptide identification

Fifteen sub-fractions showed anti-tyrosinase activity higher than 500 mg kojic acid (KA) eq/g prot (Table 2, data in bold) and were selected for peptide sequencing. Most of them were sub-fractions collected at higher RT and coming from R1 fractions (7 from alc R1, 6 from pro R1) (Figure 3). Two active sub-fractions were selected from pro P1 while none from alc P1 (Table 2). These sub-fractions were subjected to liquid chromatography coupled with high resolution mass spectrometry to perform peptide identification and a total of 365 peptide sequences were obtained (Table 3).

**TABLE 3:** Characteristics of the peptide pool present in sub-fractions obtained from Alcalase and Protamex R1, and Protamex P1 fractions and showing the highest anti-tyrosinase activity.

Sub-fraction n.	Peptide n.	Length (amino acid n.)	MW range (Da)
**Alcalase R1**			
12	20	8–13	731–1451
13	12	7–11	855–1281
14	29	7–11	687–1281
16	33	7–18	716–1408
18	26	7–11	784–1309
19	22	7–14	784–1309
24	13	7–11	784–1327
**Protamex R1**			
12	19	7–12	714–1281
14	21	7–11	784–1281
16	24	7–16	692–1420
18	29	7–17	766–1566
19	24	7–15	733–1468
24	17	7–18	746–2005
**Protamex P1**			
10	27	7–13	784–1281
13	49	7–15	784–1422

Most of the analyzed fractions showed a similar profile, with few peptides present and sequenced and a low protein content. Most of the identified peptides had a length between 7 and 11 amino acid residues and a MW between 750 and 1500 Da. The complete peptide amino acid sequences with rice protein accession are listed in Supplementary Table 2.

In particular, regarding the samples belonging to alc R1 fractions, the most abundant peptides found were NLNNNPYFKGT, SAPIYTQPRH, and TPIQYKSY, which are, respectively, fragments of granule-bound starch synthase I, glucose-1-phosphate adenylyltransferase, and glutelin. In pro R1 they were NLNNNPYFKGT, HGAFTPR, LDWYKGPT, TNPWHSPRQ, which are, respectively, fragments of granule-bound starch synthase I, glutein, starch synthase, and glutein. In pro P1 samples the most abundant peptide is again NLNNNPYFKGT. Peptide distribution in all fractions was similar in alc and pro samples.

## Discussion

### Peptide production processes

The rice starch industry feedstock was hydrolyzed by Alcalase (alc) and Protamex (pro) commercial proteases, with the aim to produce bioactive peptides in bioreactor scale.

By applying digestion condition 1 (0.5 U enz/g prot, 60°C, 120 min, 1 L of final reaction volume, Table 1) significantly higher peptide yields were observed in bioreactor compared to flask scale (Ferri et al., 2017) at the end of the 120 min hydrolysis, with an increase of +5.4-times with alc and of +3.7-times with pro, suggesting a more efficient hydrolysis occurring in bioreactor (Figures 1A,B, 120 min samples). Antioxidant activity of the final total digestates (TD) was also increased, with 4.1 to 18-fold higher alc hydrolysates respect to other alc digestates (Dei Piu et al., 2014; Ferri et al., 2017). On the other hand, bioreactor microorganism fermentation of a similar feedstock led to a maximum antioxidant activity of 0.104 g AA eq/L after 72 h (Babini et al., 2020), significantly lower than values here reported (Figures 1C,D, 120 min samples) obtained by using isolated proteolytic enzymes with a faster digestion process.

Further optimization steps considered temperature reduction to 55°C (condition 2), decrease of E/S ratio to 0.2 and 0.05 U enz/g protein (conditions 3 and 4) and scale up to 2 L of final reaction volume (conditions 5 and 6, Table 1) (Figure 1). In all processes and for both enzymes, peptides were quickly released during the first 30 min and then their level increase slowly sometimes reaching a plateau (i.e., conditions 3 and 4) (Figures 1A,B). Similar trends were also observed for antioxidant capacity.

Temperature reduction to 55°C was selected as process temperature allowing to save energy in view of a more sustainable future industrial process. On the contrary, the use of lower amounts of enzyme was not implemented as it led to a significative yield loss (Figure 1).

Conditions 2 and 3, at 55°C and 1 L working volume (Table 1), allowed to reach the best balance between TD characteristics (peptide release yield and bioactivity) and process condition (temperature and enzyme units). These were further up-scaled to 2 L reaction volume (conditions 5 and 6, respectively), confirming peptide content in TD (Figure 1). It was previously demonstrated for alc, that, when this enzyme is used at optimal conditions, it keeps digesting the released peptides leading to their degradation and, therefore, to a decreased antioxidant activity (Ferri et al., 2017). Proteolytic enzymes can therefore produce different peptides in different conditions and also modify them during the time of hydrolysis. These observations are in agreement with the present results that showed similar final total peptide yields in condition 5 (2 L volume, 0.5 U enz/g prot) and in condition 2 (1 L volume, 0.5 U enz/g prot) (Supplementary Figure 1), but with a lower antioxidant activity in condition 5 digestate, most probably due to a more efficient hydrolysis in larger scale (i.e., better stirring) (Figure 1). A similar explanation could be applied when comparing 1 L data in condition 3 (0.2 U enz/g prot), with lower content of peptides but a higher antioxidant activity, to those of condition 2 (0.5 U enz/g prot) in which the higher amount of enzyme led to an increased peptide content with a lower antioxidant activity.

In view of a possible future exploitation of the obtained extracts, condition 5, showing the highest content of peptides in 2 L scale, was selected for further investigation.

### Peptide fractions characterization

The liquid TD supernatants (120 min samples) obtained from both alc and pro processes with condition 5 (Figure 1), were fractionated by cross-flow filtration leading to obtain peptide fractions with different MW, with concentrated peptides and, therefore, enhanced bioactivities (Figure 2).

The analysis of lyophilized and resuspended samples showed that fraction powder weight corresponded to protein weight (Figures 2A,B, red and gray bars comparison) demonstrating that fractions contained almost totally peptides with the exception of P1 from both alc and pro. This exception was probably due to the presence in the final permeate (below 1 kDa) of salts deriving from several process steps from industrial starch extraction to enzymatic digestion.

After both enzyme treatments, fractions R8 and P1 showed the highest protein concentrations (Figures 2A,B), antioxidant activities (Figures 2C,D), and anti-tyrosinase capacity (Figures 2E,F). These results seem to demonstrate a more efficient digestion occurring in bioreactor with respect to previous flask-scale data where most of the peptides and of the bioactivities were in high MW fractions (alc R8, pro R8, and R5) (Ferri et al., 2017). Moreover, the present fractions had higher antioxidant activity (up to 12-fold in P1 fractions, with respect to flask scale). The present results were in agreement with studies performed on brown rice peptides in which it was observed that peptides with MW lower than 1.5 kDa strongly contributed to antioxidant activity (Selamassakul et al., 2018).

The results of *in vitro* anti-tyrosinase capacity (Figures 2E,F) demonstrated that small peptides needed to be isolated and concentrated to display a significant anti-tyrosinase activity. A recent study reported that the most active tyrosinase-inhibitor rice bran peptides derived from albumin hydrolyzed by papain, while globulin, glutelin, and prolamin had lower inhibitory capacities (Kubglomsong et al., 2018).

According to anti-inflammatory activity results, alc R8 appeared to be the most promising fraction (Figures 2G,H). The present results confirmed previous preliminary evidences (Ferri et al., 2017) showing that alc digestion of rice starch processing by-product, provides the release of anti-inflammatory peptides that may find a suitable application in the nutraceutical and pharmaceutical sectors.

Overall, the bioactivities data (Figure 2) showed that lyophilization preserved or even increased peptide bioactivity. This observation could be very valuable for potential industrial exploitation demonstrating that the obtained peptide fractions could be more stable and easily stored in dry powder form in comparison to liquid form. Lyophilization is in fact one of the most used methods to prepare pharmaceutical formulations containing proteins or peptides, to impart long term stability during storage (Jain et al., 2019). The drying methods could also affect bioactivities. For example, lyophilization of edible asparagus was proved to preserve tyrosinase inhibition activity more than other drying techniques (Yu et al., 2019). All fractions showed different levels of one or more biological activities that varied depending on the enzymatic treatment and on the fraction MW range. In case of both antioxidant and anti-tyrosinase capacities, P1 and R8 were the most active fractions (Figures 2C–F), possibly due to the high peptide concentration (Figures 2A,B) or to the presence of peptides exhibiting multiple biological functionalities, as recently reported by Taniguchi et al. (2020) in low protein rice by-product. In general, powders showed to have equal or higher activity with respect to corresponding liquid fractions (Figures 2C–H). These results seem to be very promising in view of a future industrial application as ingredients of the fractions that showed high stability and easy and cheap storage possibilities.

### Amino acid profile and *in silico* nutritional characteristics and peptide fractions predicted taste

The free amino acid profile can have possible effects on nutritional, biological activities and flavor characteristics of peptide fractions, therefore information concerning amino acid composition can be useful for exploitation evaluation.

As expected, free amino acids were largely released during hydrolysis process and they were concentrated in P1 fractions (Supplementary Table 1). It was previously observed that free amino acids, together with peptide chain length up to 10 amino acids, could easily pass through the ultrafiltration membrane with 1 kDa cut-off (Selamassakul et al., 2020) accordingly with the present results (Supplementary Tables 1, 2).

The present rice peptide fractions can be considered good sources of essential amino acids (EAA) (Supplementary Table 1; Figures 3A–D), besides these samples could also act as potential free radical scavengers (Selamassakul et al., 2020), when used as ingredients in food and nutraceutical product formulations. In this context, the presence of amino acids with particular structural characteristics was also considered (Supplementary Table 1; Figures 3A–D). Branched-chain amino acids (BCAA) seem to promote muscle protein synthesis, while hydrophobic amino acid (HAA) were correlated with peptide antioxidant activity (Selamassakul et al., 2020). These compositions are in accordance with literature data on rice peptides. For example, the highest content of EAA and of HAA (respectively, 50.5 and 36.1% of TOT) was obtained in glutelin-derived peptides after bromelain digestion (Selamassakul et al., 2018). In that case, the authors considered such amino acid composition as well-balanced and promoted the use of the fractions as nutritional supplements, functional ingredients and flavor enhancers in foods (Selamassakul et al., 2018).

The presence of free amino acids can contribute to flavor characteristics: an important aspect in case of use as food or nutraceutical ingredients. After an *in silico* analysis, the flavor predicted most frequently was bitter taste (Supplementary Table 1; Figures 3E–H), consistently with previous rice peptides observations, both in prediction mode and by trained panelists (Selamassakul et al., 2020). Conversely to literature data (Selamassakul et al., 2020), umami taste amino acid contribution was lower that bitter one (Supplementary Table 1; Figures 3E–H). Previous reports proved that bitter taste may be caused by the presence of HAA (Selamassakul et al., 2016), suggesting that amino acids with hydrophobic side chains offer a binding site for a bitter taste receptor (Selamassakul et al., 2020).

### Sub-fractionation and anti-tyrosinase activity analysis

R1 and P1 fractions, containing the smallest MW peptides, were further sub-fractionated (Figure 4) and protein content and anti-tyrosinase activity were evaluated (Table 2). On the contrary to Kubglomsong et al. (2018) that observed lower tyrosinase inhibition and copper chelation activities in the smallest MW rice bran-derived peptide fractions, the present data showed increased anti-tyrosinase capacity with decreasing MW of the peptide sub-fractions. At least 15 sub-fractions had a promising activity, suggesting a putative application of these peptides as a natural tyrosinase inhibitors in the food and cosmetic industries.

### Peptide identification

A total of 365 peptides were identified (Table 3; Supplementary Table 2) from the 15 most active sub-fractions, according to anti-tyrosinase results (Table 2, data in bold). Several peptides were detected both in alc and pro samples, confirming the wide range of specificity of both enzymes. The rice protein sources of these peptides were mainly those expected for this type of by-product: starch synthase, glucose-1-phosphate adenylyl transferase and glutelin. The peptide MW was consistent with that of other hydrolysates with anti-tyrosinase capacity and obtained from processing by-products, e.g., silk (Wu et al., 2008). Most of the identified peptides contained amino acid residues with a hydroxyl group (SER, THR, or TYR), which could interact *via* hydrogen bonds with the active site of the enzyme, thus showing anti-tyrosinase activity. The interaction with the active site (and therefore the inhibiting activity) can also be ascribed to hydrophobic interactions (aliphatic moiety of VAL, ALA, or LEU) and π-π interactions (aromatic moiety of PHE, TYR, or TRP) (Hariri et al., 2021), confirming previous data (Figure 3; Supplementary Table 1). Most of these peptides have been here identified for the first time in rice. The peptide LDWYKGPT, found in the pro R1 hydrolysate (sub-fractions 16, 18, and 19) and in the pro P1 hydrolysate (sub-fraction 13), was previously identified as a potential ACE inhibitor peptide only in broad beans hydrolysates obtained with Neutrase and Protamex (Tang et al., 2017).

## Conclusion

The present study demonstrated the feasibility of bioactive peptide production from an industrial rice starch processing by-product. Alcalase and Protamex optimized enzymatic processes proved to be able to generate total digestates containing peptides exerting an antioxidant activity. Different obtained peptide fractions, according to amino acid composition, showed positive nutritional characteristics with a 50–55% content of essential amino acids, but a predicted bitter taste. That limitation could possibly be overcome by flavoring or masking agents in food and nutraceutical industrial uses. Fractions showed different bioactivities depending on the enzymatic treatment and on the MW range: P1 and R8 were the most antioxidant fractions; R8 from Alcalase treatment was promising for anti-inflammatory potential; R1 and P1 were more active in tyrosinase inhibition with 365 potentially bioactive peptides sequenced. Both enzymes demonstrated to be a good tool for peptide production from such rice by-product, but peptide sequences seemed to depend more on feedstock proteins than on the used enzyme.

Optimized enzymatic processes were composed of few steps and no solvents or harsh conditions were used. Therefore, they are economic and environment-friendly and can be easily scaled-up. Moreover, lyophilization preserved or even increased peptide bioactivities and being this finding very promising for potential industrial exploitation of these fractions as ingredients (high stability and easy storage). The present study demonstrated the possibility to produce added value peptides of potential interest for a further exploitation into food, nutraceutical, pharmaceutical, and cosmetic application fields.

## Data availability statement

The original contributions presented in this study are included in the article/Supplementary Material, further inquiries can be directed to the corresponding author.

## Author contributions

MF performed most of the experiment and wrote the manuscript original draft. TT and BP performed peptide identification. EM and MMC performed biological activity assessment in cell-based systems. EB and AG performed FPLC sub-fractionation processes. JG-H and KB performed membrane fractionation activities. NR contributed to bioreactor processes. ML contributed to conceptualization and supplied the by-product. AT conceptualized the study and contributed to write the manuscript. All authors contributed to revise the manuscript and approved the submitted version.

## Conflict of interest

ML was employed by Carminia snc. The remaining authors declare that the research was conducted in the absence of any commercial or financial relationships that could be construed as a potential conflict of interest.

## Publisher’s note

All claims expressed in this article are solely those of the authors and do not necessarily represent those of their affiliated organizations, or those of the publisher, the editors and the reviewers. Any product that may be evaluated in this article, or claim that may be made by its manufacturer, is not guaranteed or endorsed by the publisher.
