# Supplementary material for: Looking for peptides from rice starch processing by-product: Bioreactor production, anti-tyrosinase and anti-inflammatory activity, and in silico putative taste assessment
Source: Front Plant Sci. 2022 Jul 15;13:929918. doi: 10.3389/fpls.2022.929918 (PMC9335147; doi:10.3389/fpls.2022.929918)
Supplement: Supplementary file 1 [file Data_Sheet_1.PDF]

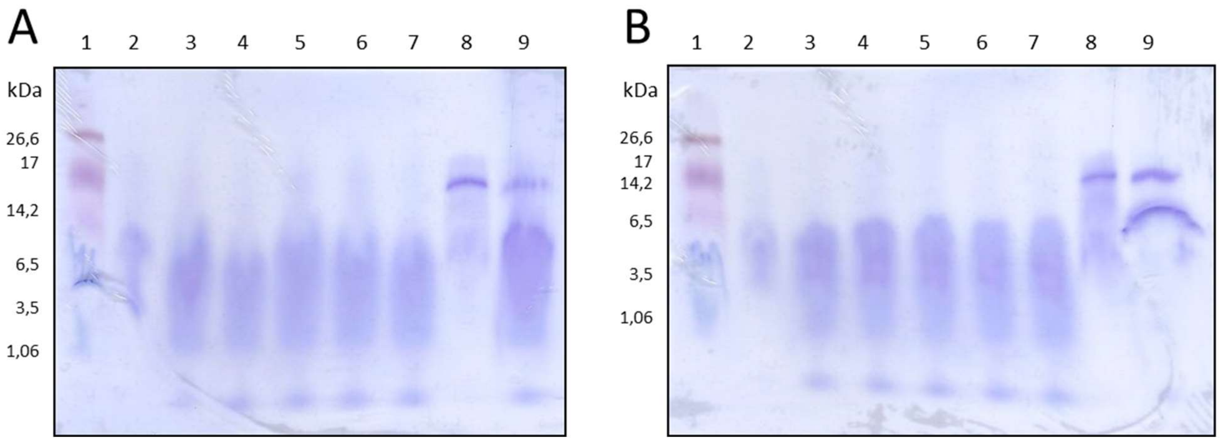

Supplementary Figure S1. Gel electrophoresis (20% w/v of acrylamide) protein profile of rice by-product total digestates and supernatant after 0.5 U/g (A) Alcalase and (B) Protamex enzyme hydrolysis at 55°C in 2 L bioreactor (condition 5). Loading scheme: lane 1: molecular mass markers; lane 2: not digested (ND); lane 3: 0 min; lane 4: 30 min; lane 5: 60 min; lane 6: 90 min; lane 7: 120 min; lane 8: ND, not centrifuged; lane 9: 120 min, not centrifuged. Lanes 2-7 were loaded with 25 µL of supernatant and lanes 8-9 with 3 µL of not centrifuged sample.

Supplementary Table S1. Quantification of free amino acids in not digested (ND) by-product, total digestate (TD condition 5) and peptide fraction samples from Alcalase and Protamex treatments, in liquid and lyophilised forms. Amino acids content (µmol/L) was measured by HPLC-fluorimeter after AccQ Tag kit derivatisation. Different letters indicate a statistically significant difference (one-way ANOVA test followed by post-hoc corrected two-tailed Tukey test, $p < 0.05$), from the lowest (a) to the highest (m). Data are the mean of two independent analyses (n = 3) ± SD.

		SER	ASP	HIS	GLU	GLY	ARG	THR	ALA	PRO	CYS	TYR	VAL	MET	LYS	ILE	LEU	PHE	Total
	**ND**	1.4^{a} ±0.6	3.6^{a} ±0.4	3.7^{a} ±0.3	4.0^{a} ±1.5	2.5^{a} ±0.9	3.0^{a} ±0.9	3.6^{a} ±0.9	9.9^{a} ±1.1	1.9^{a} ±0.2	7.4^{a} ±2.2	5.9^{a} ±0.9	4.1^{a} ±0.6	1.3^{a} ±0.5	5.0^{a} ±0.9	2.8^{a} ±0.7	6.0^{a} ±1.1	4.6^{a} ±0.9	**70.7^{b} ±10.6**
**Alcalase, liquid**	**TD**	32.2^{b} ±8.7	221.7^{c} ±9.9	40.6^{b} ±2.3	189.9^{c} ±4.9	193.9^{c} ±32.4	126.9^{b,c} ±38.1	128.9^{c} ±13.4	503.8^{d,e} ±160.7	27.5^{b} ±6.8	906.7^{e,f} ±159.2	326.3^{d} ±72.8	282.9^{c} ±54.0	127.4^{b} ±27.7	367.6^{c,d} ±84.2	161.6^{c} ±36.9	417.3^{d} ±81.1	254.9^{c,d} ±66.2	**4310.0^{h} ±646.5**
	**R0.2 µm**	36.5^{b} ±7.2	509.7^{d,e} ±94.1	107.3^{b,c} ±26.5	62.0^{b} ±2.9	482.0^{d} 58.5	457.3^{d} ±44.4	203.6^{c} ±19.0	578.4^{d,e} ±103.1	23.0^{b} ±1.9	906.8^{e,f} ±272.0	450.1^{d} ±142.6	353.3^{c,d} ±100.2	296.6^{c,d} ±108.5	436.2^{c,d} ±159.7	250.2^{c,d} ±72.9	738.3^{f} ±110.7	454.5^{d} ±68.2	**6345.7^{i} ±951.9**
	**R8**	176.3^{c} ±81.8	1810.5^{f,g} ±406.1	595.7^{d,e} ±101.6	1381.9^{f,g} ±910.0	1760.5^{f,g} ±616.1	3046.8^{gh} ±583.6	1126.0^{e,f} ±230.4	2490.9^{g} ±494.1	162.2^{b,c} ±81.8	1631.3^{e,f,g} ±850.9	1832.4^{f,g} ±370.0	1812.7^{f,g} ±271.9	882.2^{e,f} ±235.9	1646.0^{f,g} ±572.1	1199.6^{f} ±177.9	2767.0^{g,h} ±415.1	2185.0^{f,g} ±327.8	**26506.9^{l} ±3976.0**
	**R5**	50.7^{b,c} ±7.6	219.2^{c,d} ±32.9	83.8^{b} ±12.6	69.6^{b} ±10.4	421.0^{c} ±63.2	729.0^{e} ±109.4	198.7^{c} ±29.8	477.3^{d} ±71.6	12.7^{b} ±1.9	451.4^{d} ±67.7	657.7^{d,e} ±98.7	413.7^{d} ±62.1	391.3^{d} ±58.7	673.2^{d} ±101.0	313.6^{c} ±47.0	663.0^{d} ±99.5	445.8^{d} ±66.9	**6271.6^{i} ±940.7**
	**R1**	16.3^{b} ±2.4	166.8^{c} ±25.0	39.0^{b} ±5.9	142.3^{c} ±21.3	198.0^{c} ±29.7	367.7^{d} ±55.2	126.0^{c} ±18.9	321.4^{c} ±48.2	0.0^{a}	389.4^{d} ±58.4	442.3^{d} ±66.3	295.0^{c,d} ±44.3	300.2^{c,d} ±45.0	470.3^{d} ±70.5	241.8^{c} ±36.3	454.1^{d} ±68.1	330.9^{c,d} ±49.6	**4301.4^{h} ±645.2**
	**P1**	171.0^{c} ±51.3	1586.3^{f} ±374.5	518.0^{d,e} ±141.6	289.8^{c,d} ±106.6	1411.8^{f} ±205.5	2039.0^{f,g} ±353.2	1122.6^{f} ±26.9	2933.1^{g,h} ±286.0	140.9^{c} ±60.9	5585.7^{h,i} ±1675.7	2509.2^{g} ±165.8	2134.3^{f,g} ±320.1	1860.2^{f,g} ±248.7	2970.1^{g,h} ±678.8	1969.2^{f,g} ±295.4	3814.8^{h} ±572.2	2433.4^{g} ±365.0	**33489.1^{lm} ±5023.4**
**Alcalase, lyophilised**	**R0.2 µm**	49.9^{b} ±7.5	547.7^{d,e} ±82.2	103.3^{b} ±15.5	68.5^{b} ±10.3	512.8^{d} ±76.9	634.5^{d,e} ±95.2	214.3^{c} ±32.1	612.1^{d,e} ±91.8	19.9^{b} ±3.0	747.6^{d,e} ±112.1	571.6^{d,e} ±85.7	420.1^{d} ±63.0	381.7^{d} ±57.3	625.0^{d,e} ±93.8	325.4^{c} ±48.8	722.1^{e} ±108.3	483.8^{d} ±72.6	**7040.5^{i} ±1056.1**
	**R8**	208.3^{c} ±42.3	1592.9^{f} ±276.0	507.0^{d} ±50.3	1096.0^{e,f} ±328.8	1322.6^{f} ±282.3	2573.1^{fgh} ±660.4	851.2^{d,e,f} ±382.1	2087.1^{f,g} ±98.4	107.2^{b} ±54.9	2233.0^{f,g} ±812.0	1982.9^{f,g} ±263.7	1835.1^{f,g} ±55.9	798.1^{e} ±10.5	1572.5^{f} ±101.6	1022.8^{f} ±13.4	2729.3^{g,h} ±409.4	1498.8^{f} ±224.8	**24017.8^{l} ±3602.7**
	**R5**	22.9^{b} ±3.4	224.1^{c} ±33.6	65.2^{b} ±9.8	180.7^{c} ±27.1	324.0^{c} ±48.6	681.7^{d,e} ±102.3	173.9^{c} ±26.1	549.3^{d,e} ±82.4	108.6^{b} ±16.3	1517.1^{f} ±227.6	658.0^{d,e} ±98.7	435.1^{d} ±65.3	472.9^{d} ±70.9	716.6^{d,e} ±107.5	416.7^{d} ±62.5	821.1^{e} ±123.2	444.9^{d} ±66.7	**7812.7^{i,j} ±1171.9**
	**R1**	54.6^{b} ±8.2	213.9^{c} ±32.1	111.4^{b} ±16.7	54.6^{b} ±8.2	406.9^{d} ±61.0	458.1^{d} ±68.7	163.3^{c} ±24.5	438.3^{d} ±65.7	29.7^{b} ±4.5	1318.0^{f} ±197.7	559.0^{d,e} ±83.9	409.9^{d} ±61.5	423.2^{d} ±63.5	521.3^{d} ±78.2	372.9^{d} ±55.9	573.8^{d,e} ±86.1	399.9^{d} ±60.0	**6508.8^{i} ±976.3**
	**P1**	279.0^{c,d} ±97.2	1885.3^{f,g} ±315.5	679.3^{d,e} ±93.0	1707.9^{f} ±296.3	2072.8^{f,g} ±582.9	2513.0^{g} ±470.7	1339.1^{f} ±171.7	3826.8^{h} ±703.3	189.6^{c} ±29.9	9572.2^{j} ±1636.3	3031.0^{g,h} ±589.1	2599.4^{g} ±203.6	2344.4^{f,g} ±444.0	3998.7^{h} ±813.2	2259.7^{f,g} ±338.9	3583.9^{h} ±591.6	2687.9^{g} ±403.2	**44570.2^{m} ±6685.5**
**Protamex, liquid**	**TD**	28.9^{b} ±4.3	375.6^{d} ±56.3	41.7^{b} ±6.3	97.9^{b} ±14.7	264.9^{c,d} ±39.7	280.6^{c} ±42.1	107.6^{b} ±16.1	422.3^{d} ±63.3	26.7^{b} ±4.0	143.7^{c} ±21.6	275.2^{c} ±41.3	335.1^{c} ±50.3	162.7^{c} ±24.4	263.7^{c} ±39.6	294.4^{c,d} ±44.2	597.2^{d,e} ±89.6	229.3^{c,d} ±34.4	**3947.6^{h} ±592.1**
	**R0.2 µm**	57.0^{b} ±8.6	377.9^{d} ±56.7	75.8^{b} ±11.4	454.1^{d} ±68.1	335.1^{d} ±50.3	342.6^{c} ±51.4	192.1^{c} ±28.8	626.0^{d,e} ±93.9	27.7^{b} ±4.2	677.4^{d,e} ±101.6	478.7^{d} ±71.8	543.1^{d,e} ±81.5	240.8^{c} ±36.1	314.1^{c,d} ±47.1	435.7^{d} ±65.4	880.3^{e} ±132.0	400.5^{d} ±60.1	**6459.0^{i} ±968.9**
	**R8**	207.7^{c} ±31.2	589.0^{d,e} ±88.4	174.3^{c} ±26.1	945.9^{e,f} ±141.9	635.7^{d,e} ±95.4	814.1^{e} ±122.1	266.9^{c} ±40.0	1043.8^{e,f} ±156.6	18.1^{b} ±2.7	1217.0^{f} ±182.6	1163.1^{f} ±174.5	1111.0^{e,f} ±166.7	467.8^{d} ±70.2	719.0^{e} ±107.9	893.0^{e,f} ±134.0	1622.9^{f} ±243.4	838.2^{e} ±125.7	**12727.5^{k} ±1909.1**

	**R5**	18.0^{b} ±2.7	211.6^{c} ±31.7	24.5^{b} ±3.7	51.5^{b} ±7.7	184.6^{c} ±27.7	504.7^{d} ±75.7	93.4^{b} ±14.0	365.1^{d} ±54.8	42.6^{b} ±6.4	929.4^{e,f} ±139.4	494.6^{d} ±74.2	360.8^{d} ±54.1	280.1^{c,d} ±42.0	533.3^{d} ±80.0	411.8^{d} ±61.8	626.6^{d,e} ±94.0	299.4^{c,d} ±44.9	**5431.8^{h,i} ±814.6**
	**R1**	26.0^{b} ±3.9	279.6^{c,d} ±41.9	54.3^{b} ±8.1	85.1^{b} ±12.8	257.5^{c} ±38.6	506.3^{d} ±75.9	121.9^{c} ±18.3	490.6^{d} ±73.6	41.0^{b} ±6.2	650.0^{d,e} ±97.5	446.6^{d} ±67.0	500.4^{d} ±75.1	269.2^{c,d} ±40.4	473.7^{d} ±71.1	463.0^{d} ±69.5	847.2^{e} ±127.1	321.7^{c,d} ±48.3	**5834.0^{i} ±875.1**
	**P1**	406.0^{d} ±60.9	3863.4^{h} ±579.5	926.0^{e,f} ±138.9	2039.6^{f,g} ±305.9	3643.2^{h} ±546.5	4621.3^{h} ±693.2	1512.8^{f} ±226.9	9945.6^{j} ±1491.8	523.4^{d} ±78.5	7717.1^{j} ±1157.6	4654.0^{h,i} ±698.1	7657.2^{i,j} ±1148.6	3063.6^{gh} ±455.5	5035.2^{h,i} ±755.28	7005.8^{i} ±1050.9	13650.7^{k} ±2047.6	5127.6^{h,i} ±769.1	**81392.6^{n} ±12208.9**
**Protamex, lyophilised**	**R0.2 µm**	43.0^{b} ±6.5	453.2^{d} ±68.0	71.2^{b} ±10.7	447.7^{d} ±67.2	410.7^{d} ±61.6	574.5^{d,e} ±86.2	227.5^{c} ±34.1	719.6^{e} ±107.9	39.3^{b} ±5.9	467.9^{d} ±70.2	525.1^{d} ±78.8	656.1^{d,e} ±98.4	321.1^{c} ±48.2	419.5^{d} ±62.9	506.7^{d} ±76.0	954.8^{e,f} ±143.2	419.5^{d} ±62.9	**7257.2^{i,j} ±1088.6**
	**R8**	169.3^{c} ±25.4	737.7^{d,e} ±110.7	233.0^{c} ±35.0	297.0^{c,d} ±44.6	786.3^{d,e} ±117.9	1390.7^{f} ±208.6	451.0^{d} ±67.7	1115.8^{e,f} ±167.4	77.1^{b} ±11.6	1150.3^{e,f} ±172.5	1174.8^{f} ±176.2	1218.3^{f} ±182.7	489.2^{d} ±73.4	1072.9^{e,f} ±160.9	1016.3^{e,f} ±152.4	1834.8^{f,g} ±275.2	888.5^{e} ±133.3	**14102.9^{k} ±2115.4**
	**R5**	40.4^{b} ±6.1	349.2^{c,d} ±52.4	104.7^{b} ±15.7	345.4^{c,d} ±51.8	389.9^{d} ±58.5	685.3^{d,e} ±102.8	143.0^{c} ±21.5	748.1^{e} ±112.2	71.5^{b} ±10.7	1452.6^{f} ±217.9	916.7^{e,f} ±137.5	691.3^{d,e} ±103.7	539.0^{d,e} ±80.9	1112.0^{e,f} ±166.8	914.4^{e,f} ±137.2	1312.1^{f} ±196.8	484.3^{d} ±72.6	**10299.9^{j,k} ±1545.0**
	**R1**	48.2^{b} ±7.2	344.0^{c,d} ±51.6	65.0^{b} ±9.8	93.1^{b} ±14.0	283.0^{c} ±42.5	513.3^{d,e} ±77.0	140.8^{c} ±21.1	643.1^{d,e} ±96.5	56.9^{b} ±8.5	560.8^{d,e} ±84.1	540.5^{d,e} ±81.1	504.8^{d} ±75.7	417.8^{d} ±62.7	589.4^{d,e} ±88.4	555.5^{d,e} ±83.3	1011.1^{e,f} ±151.7	367.5^{d} ±55.1	**6734.7^{i} ±1010.2**
	**P1**	452.7^{d} ±178.2	3218.2^{gh} ±529.7	599.3^{d,e} ±108.1	1822.6^{d,e,f,g} ±1750.0	3068.1^{gh} ±853.0	3796.6^{h} ±483.3	1453.5^{f} ±192.5	7346.3^{i,j} ±1822.2	472.6^{d} ±100.4	7081.8^{i,j} ±2845.7	3943.8^{g,h} ±1193.2	6291.0^{i} ±1114.6	2528.8^{f,g} ±713.2	4320.9^{h,i} ±1273.4	5850.9^{h,i} ±1494.4	13505.5^{k} ±2025.8	4085.8^{h} ±1022.2	**69838.4^{n} ±10475.8**

Supplementary Table S2. Amino acid sequences of the identified peptides with rice protein accession, from 15 sub-fractions after Alcalase (alc) or Protamex (pro) hydrolysis, cross-flow filtration (retentate R1, 5 kDa > MW > 1 kDa and permeate P1, 1 kDa > MW) and sub-fractionation via size exclusion chromatography. Data processing was performed using the software Peaks Studio (Bioinformatics Solutions Inc, Waterloo, ON, Canada).

ALCALASE fraction R1

Sub-fraction n. 12. Total: 20 identified peptides, 14 peptides with rice protein accession. RT, retention time.

<table>
<tr><th>Peptide</th><th>-10lgP</th><th>Mass (Da)</th><th>Length (amino acid n.)</th><th>m/z</th><th>RT (min)</th><th>Protein</th><th>Accession</th></tr>
<tr><td>NLNNNPYFKGT</td><td>53.76</td><td>1280.6150</td><td>11</td><td>641.3151</td><td>14.18</td><td>Granule-bound starch synthase I</td><td>tr|A0A0H4BM25|A0A0H4BM25_ORYSI:tr|A0A0E0DW79|A0A0E0DW79_9ORYZ:tr|A8QXE7|A8QXE7_ORYSI:tr|V5NEJ7|V5NEJ7_ORYSA:tr|A0A0E0DW80|A0A0E0DW80_9ORYZ:tr|A0EQH2|A0EQH2_ORYSJ:tr|A0EQK7|A0EQK7_ORYRU:tr|A0EQD4|A0EQD4_ORYSI:tr|A0EQK4|A0EQK4_ORYRU:tr|A0EQE0|A0EQE0_ORYSA:tr|A0EQK5|A0EQK5_ORYRU:tr|A0EQK6|A0EQK6_ORYRU:P0C585|SSG1_ORYSA:tr|B8XEK3|B8XEK3_ORYSA:tr|D0TZY6|D0TZY6_ORYSI:tr|A0A3Q9T378|A0A3Q9T378_ORYSA:tr|D3U2H9|D3U2H9_ORYSA:tr|B8XEJ7|B8XEJ7_ORYSA:tr|B8XEK7|B8XEK7_ORYSA:tr|A0A3Q9T3Z7|A0A3Q9T3Z7_ORYSA:tr|B8XEJ2|B8XEJ2_ORYSA:tr|B8XEK2|B8XEK2_ORYSA:tr|A0A076FRI5|A0A076FRI5_ORYSJ:Q42968|SSG1_ORYGL:A2Y8X2|SSG1_ORYSI:tr|C8CBL1|C8CBL1_ORYSJ:tr|B8XEJ8|B8XEJ8_ORYSA:tr|B1B5Z0|B1B5Z0_ORYSI:tr|B1B5Z1|B1B5Z1_ORYSI:tr|A0A0D9WLF6|A0A0D9WLF6_9ORYZ:tr|A0A0E0A4K1|A0A0E0A4K1_9ORYZ</td></tr>
<tr><td>SAPIYTQPRH</td><td>43.00</td><td>1168.5989</td><td>10</td><td>585.3069</td><td>6.21</td><td>Glucose-1-phosphate adenylyltransferase</td><td>tr|D4AIA3|D4AIA3_ORYSI:tr|B7EVB8|B7EVB8_ORYSJ:P15280-2|GLGS2_ORYSJ:tr|A0A0D3GZB2|A0A0D3GZB2_9ORYZ:tr|A0A0E0H7V9|A0A0E0H7V9_ORYNI:tr|A0A0E0LU24|A0A0E0LU24_ORYPU:tr|A0A0E0QHR8|A0A0E0QHR8_ORYRU:tr|B8XED8|B8XED8_ORYSA:tr|B8XED9|B8XED9_ORYSI:tr|B8XED7|B8XED7_ORYSI:tr|B8XEE8|B8XEE8_ORYSA:tr|B8XEF0|B8XEF0_ORYSI:tr|B8XEF2|B8XEF2_ORYSI:tr|B8XEE9|B8XEE9_ORYSI:tr|B8XEE1|B8XEE1_ORYSI:tr|B8XEE2|B8XEE2_ORYSJ:tr|B8XEE5|B8XEE5_ORYSJ:tr|B8XEE6|B8XEE6_ORYSA:tr|A2YU91|A2YU91_ORYSI:P15280|GLGS2_ORYSJ:tr|A0A0E0AUC9|A0A0E0AUC9_9ORYZ:tr|I1QHZ2|I1QHZ2_ORYGL:tr|A0A0D3GZB1|A0A0D3GZB1_9ORYZ:tr|J3MSE0|J3MSE0_ORYBR:tr|A0A0D9X6Z4|A0A0D9X6Z4_9ORYZ:tr|A0A0D9X6Z3|A0A0D9X6Z3_9ORYZ:tr|A0A0E0H7V8|A0A0E0H7V8_ORYNI:tr|A0A0E0QHR7|A0A0E0QHR7_ORYRU:tr|A0A0E0LU23|A0A0E0LU23_ORYPU</td></tr>
</table>

<table>
<tr><td>TGKSPYFSN</td><td>40.57</td><td>999.4661</td><td>9</td><td>500.7407</td><td>8.56</td><td>Globulin 2</td><td>tr|O65043|O65043_ORYSA:tr|Q9ZRH1|Q9ZRH1_ORYSA:tr|Q9ZRH0|Q9ZRH0_ORYSA:tr|A0A0D3FQ29|A0A0D3FQ29_9ORYZ:tr|A3ANJ6|A3ANJ6_ORYSJ:B8AL97|CUCIN_ORYSI:tr|A0A0E0P2F5|A0A0E0P2F5_ORYRU:tr|A0A0E0GUU5|A0A0E0GUU5_ORYNI:Q852L2|CUCIN_ORYSJ:tr|A0A0E0GUU4|A0A0E0GUU4_ORYNI</td></tr>
<tr><td>KGGIPIGIGK</td><td>38.27</td><td>938.5912</td><td>10</td><td>470.3038</td><td>11.95</td><td>Glucose-1-phosphate adenylyltransferase</td><td>tr|D4AIA3|D4AIA3_ORYSI:tr|B7EVB8|B7EVB8_ORYSJ:P15280-2|GLGS2_ORYSJ:tr|A0A0D3GZB2|A0A0D3GZB2_9ORYZ:tr|A0A0E0H7V9|A0A0E0H7V9_ORYNI:tr|A0A0E0LU24|A0A0E0LU24_ORYPU:tr|A0A0E0QHR8|A0A0E0QHR8_ORYRU:tr|B8XED8|B8XED8_ORYSA:tr|B8XED9|B8XED9_ORYSI:tr|B8XED7|B8XED7_ORYSI:tr|B8XEE8|B8XEE8_ORYSA:tr|B8XEF0|B8XEF0_ORYSI:tr|B8XEF2|B8XEF2_ORYSI:tr|B8XEE9|B8XEE9_ORYSI:tr|B8XEE1|B8XEE1_ORYSI:tr|B8XEE2|B8XEE2_ORYSJ:tr|B8XEE5|B8XEE5_ORYSJ:tr|B8XEE6|B8XEE6_ORYSA:tr|A2YU91|A2YU91_ORYSI:P15280|GLGS2_ORYSJ:tr|A0A0E0AUC9|A0A0E0AUC9_9ORYZ:tr|I1QHZ2|I1QHZ2_ORYGL:tr|A0A0D3GZB1|A0A0D3GZB1_9ORYZ:tr|J3MSE0|J3MSE0_ORYBR:tr|A0A0D9X6Z4|A0A0D9X6Z4_9ORYZ:tr|A0A0D9X6Z3|A0A0D9X6Z3_9ORYZ:tr|A0A0E0H7V8|A0A0E0H7V8_ORYNI:tr|A0A0E0QHR7|A0A0E0QHR7_ORYRU:tr|A0A0E0LU23|A0A0E0LU23_ORYPU</td></tr>
<tr><td>SQNFPILR(sub N)</td><td>35.63</td><td>973.5345</td><td>8</td><td>487.7740</td><td>17.77</td><td>Glutelin</td><td>tr|T1T4Y4|T1T4Y4_ORYSI:tr|A1YQG3|A1YQG3_ORYSJ:tr|A2Z708|A2Z708_ORYSI:tr|I1QU95|I1QU95_ORYGL:tr|A0A0E0M7E8|A0A0E0M7E8_ORYPU:tr|A0A0E0BA63|A0A0E0BA63_9ORYZ:tr|A0A0E0IRV1|A0A0E0IRV1_ORYNI:tr|A0A0E0QYR6|A0A0E0QYR6_ORYRU:tr|A0A0D3HDD5|A0A0D3HDD5_9ORYZ:tr|A0A0E0IRV2|A0A0E0IRV2_ORYNI</td></tr>
<tr><td>AFEPIRSVR</td><td>32.77</td><td>1073.5981</td><td>9</td><td>537.8065</td><td>11.12</td><td>Glutelin</td><td>tr|T1T4Y4|T1T4Y4_ORYSI:tr|A1YQG3|A1YQG3_ORYSJ:tr|A2Z708|A2Z708_ORYSI:tr|I1QU95|I1QU95_ORYGL:tr|A0A0E0M7E8|A0A0E0M7E8_ORYPU:tr|A0A0E0BA63|A0A0E0BA63_9ORYZ:tr|A0A0E0IRV1|A0A0E0IRV1_ORYNI:tr|A0A0E0QYR6|A0A0E0QYR6_ORYRU:tr|A0A0D3HDD5|A0A0D3HDD5_9ORYZ:tr|A0A0E0IRV2|A0A0E0IRV2_ORYNI</td></tr>
<tr><td>SDKGRFF</td><td>32.32</td><td>855.4238</td><td>7</td><td>428.7198</td><td>9.98</td><td>Os02g0519900 protein</td><td>tr|A0A0P0VJL8|A0A0P0VJL8_ORYSJ:tr|A0A0P0W6A1|A0A0P0W6A1_ORYSJ:tr|A0A0D9VGF7|A0A0D9VGF7_9ORYZ:tr|A0A0D3FS13|A0A0D3FS13_9ORYZ:tr|Q01MK8|Q01MK8_ORYSA:tr|A0A0E0DCG7|A0A0E0DCG7_9ORYZ:tr|A0A0D9YSX8|A0A0D9YSX8_9ORYZ:tr|A0A0D9ZGI8|A0A0D9ZGI8_9ORYZ:tr|A0A0E0GX73|A0A0E0GX73_ORYNI:tr|A2X5F3|A2X5F3_ORYSI:tr|A0A0E0NFL3|A0A0E0NFL3_ORYRU:tr|A0A0E0KM97|A0A0E0KM97_ORYPU:tr|A0A0D3F5Q7|A0A0D3F5Q7_9ORYZ:tr|A0A0E0P4M4|A0A0E0P4M4_ORYRU:tr|A0A0E0G7F6|A0A0E0G7F6_ORYNI:tr|Q7XTK1|Q7XTK1_ORYSJ:tr|Q6H4L2|Q6H4L2_ORYSJ:tr|A0A0D9YEC3|A0A0D9YEC3_9ORYZ:tr|A0A0D3ETH6|A0A0D3ETH6_9ORYZ:tr|A2WUL5|A2WUL5_ORYSI:tr|A0A0E0C7D6|A0A0E0C7D6_9ORYZ:tr|A0A0E0FSG8|A0A0E0FSG8_ORYNI:tr|A0A0E0N1V6|A0A0E0N1V6_ORYRU:tr|Q8W0C4|Q8W0C4_ORYSJ:tr|A0A0E0JNF3|A0A0E0JNF3_ORYPU:tr|A2ZXD6|A2ZXD6_ORYSJ:tr|A0A0E0DA54|A0A0E0DA54_9ORYZ:tr|A0A0E0DA52|A0A0E0DA52_9ORYZ:tr|A0A0E0DA53|A0A0E0DA53_9ORYZ</td></tr>
<tr><td>TNFNNRPNSF</td><td>32.27</td><td>1209.5526</td><td>10</td><td>605.7851</td><td>11.09</td><td>Starch branching enzyme 1</td><td>tr|A0A0E0E6K5|A0A0E0E6K5_9ORYZ:Q01401-2|GLGB_ORYSJ:tr|A0A2S0T039|A0A2S0T039_ORYSA:tr|A0A2S0T029|A0A2S0T029_ORYSA:Q01401|GLGB_ORYSJ:tr|A0A0E0Q2U7|A0A0E0Q2U7_ORYRU:tr|A0A0E0Q2U8|A0A0E0Q2U8_ORYRU:tr|A0A0E0Q2U9|A0A0E0Q2U9_ORYRU</td></tr>
</table>

<table>
<tr><td>AFEPIRTVR</td><td>31.19</td><td>1087.6138</td><td>9</td><td>544.8149</td><td>11.44</td><td>Gt3</td><td>tr|A0A0E0D2D4|A0A0E0D2D4_9ORYZ:tr|A0A0E0NWT6|A0A0E0NWT6_ORYRU:tr|B7U2J6|B7U2J6_ORYSJ:tr|T1T5D8|T1T5D8_ORYSI:tr|J3M4W6|J3M4W6_ORYBR:tr|I1PCG0|I1PCG0_ORYGL:tr|A0A0E0KFA5|A0A0E0KFA5_ORYPU:tr|A0A0E0ITU1|A0A0E0ITU1_ORYNI:tr|A0A0D3FK24|A0A0D3FK24_9ORYZ:tr|Q10JA8|Q10JA8_ORYSJ:Q09151|GLUA3_ORYSJ:tr|A0A0D9Z962|A0A0D9Z962_9ORYZ:tr|A0A0D9XVA4|A0A0D9XVA4_9ORYZ:tr|B9F952|B9F952_ORYSJ:tr|B8AKE2|B8AKE2_ORYSI</td></tr>
<tr><td>GRLDSGKQPPRQL</td><td>31.08</td><td>1450.8004</td><td>13</td><td>726.4082</td><td>6.15</td><td>Uncharacterized protein</td><td>tr|A0A0E0E959|A0A0E0E959_9ORYZ:tr|A2YJG7|A2YJG7_ORYSI:tr|A0A0E0IR31|A0A0E0IR31_ORYNI:Q7X8H9|AI172_ORYSJ:tr|A0A0E0AHB5|A0A0E0AHB5_9ORYZ:tr|A0A0E0Q5N0|A0A0E0Q5N0_ORYRU</td></tr>
<tr><td>HRDFFLA</td><td>30.52</td><td>904.4555</td><td>7</td><td>453.2349</td><td>16.23</td><td>Gt3</td><td>tr|A0A0E0D2D4|A0A0E0D2D4_9ORYZ:tr|A0A0E0NWT6|A0A0E0NWT6_ORYRU:tr|B7U2J6|B7U2J6_ORYSJ:tr|T1T5D8|T1T5D8_ORYSI:tr|J3M4W6|J3M4W6_ORYBR:tr|I1PCG0|I1PCG0_ORYGL:tr|A0A0E0KFA5|A0A0E0KFA5_ORYPU:tr|A0A0E0ITU1|A0A0E0ITU1_ORYNI:tr|A0A0D3FK24|A0A0D3FK24_9ORYZ:tr|Q10JA8|Q10JA8_ORYSJ:Q09151|GLUA3_ORYSJ:tr|A0A0D9Z962|A0A0D9Z962_9ORYZ:tr|A0A0D9XVA4|A0A0D9XVA4_9ORYZ:tr|B9F952|B9F952_ORYSJ:tr|B8AKE2|B8AKE2_ORYSI</td></tr>
<tr><td>YSNTPGLR(sub V)</td><td>30.35</td><td>906.4559</td><td>8</td><td>454.2348</td><td>7.00</td><td>Glutelin</td><td>tr|B9F4T1|B9F4T1_ORYSJ:tr|A1YQH4|A1YQH4_ORYSJ:tr|A1YQH6|A1YQH6_ORYSJ:tr|I1NZ08|I1NZ08_ORYGL:tr|A0A0D9VE70|A0A0D9VE70_9ORYZ:tr|M1G571|M1G571_ORYSJ:tr|M1G2E3|M1G2E3_ORYSI:tr|Q0E2G5|Q0E2G5_ORYSJ:tr|C0L8H1|C0L8H1_ORYSJ:tr|A0A0D9YLS5|A0A0D9YLS5_9ORYZ:tr|Q84X94|Q84X94_ORYSJ:tr|A0A0E0G459|A0A0E0G459_ORYNI:tr|Q0E2D5|Q0E2D5_ORYSJ:tr|A1YQH5|A1YQH5_ORYSJ:tr|C0L8H2|C0L8H2_ORYSJ:tr|Q84X93|Q84X93_ORYSJ:tr|Q6ESW6|Q6ESW6_ORYSJ</td></tr>
<tr><td>RYVLEPR</td><td>29.67</td><td>931.5239</td><td>7</td><td>466.7700</td><td>6.92</td><td>Uncharacterized protein</td><td>tr|J3L3K2|J3L3K2_ORYBR</td></tr>
<tr><td>KDKNKPIIF</td><td>27.82</td><td>1101.6545</td><td>9</td><td>551.8352</td><td>10.25</td><td>Sucrose synthase</td><td>tr|A0A0P0WTZ1|A0A0P0WTZ1_ORYSJ:tr|A0A0D9WMW0|A0A0D9WMW0_9ORYZ:tr|A0A0E0HM40|A0A0E0HM40_ORYNI:tr|A2YA91|A2YA91_ORYSI:tr|A0A0D3GDS1|A0A0D3GDS1_9ORYZ:tr|I1Q096|I1Q096_ORYGL:tr|A0A0E0A682|A0A0E0A682_9ORYZ:tr|I3QD82|I3QD82_ORYSJ:tr|A0A0E0PUM1|A0A0E0PUM1_ORYRU:P30298|SUS2_ORYSJ:tr|A0A0E0L8X0|A0A0E0L8X0_ORYPU:tr|A0A0E0HM39|A0A0E0HM39_ORYNI:tr|A0A0E0HM38|A0A0E0HM38_ORYNI</td></tr>
</table>

Sub-fraction n. 13. Total: 12 identified peptides, 7 peptides with rice protein accession. RT, retention time.

<table>
<tr><th>Peptide</th><th>-10lgP</th><th>Mass (Da)</th><th>Length (amino acid n.)</th><th>m/z</th><th>RT (min)</th><th>Protein</th><th>Accession</th></tr>
<tr><td>NLNNNPYFKGT</td><td>55.52</td><td>1280.6150</td><td>11</td><td>641.3151</td><td>14.10</td><td>Granule-bound starch</td><td>tr|A0A0H4BM25|A0A0H4BM25_ORYSI:tr|A0A0E0DW79|A0A0E0DW79_9ORYZ:tr|A8QXE7|A8QXE7_ORYSI:tr|V5NEJ7|V5NEJ7_ORYSA:tr|A0A0E0DW80|A0A0E0DW80_9ORYZ:tr|A0EQH2|A0EQH2_ORYSJ:tr|A0EQK7|A0EQK7_ORYRU:tr|A0EQD4|A0EQD4_ORYSI:tr|A0EQK4|A0EQK4_ORY</td></tr>
</table>

						synthase I	RU:tr|A0EQE0|A0EQE0_ORYSA:tr|A0EQK5|A0EQK5_ORYRU:tr|A0EQK6|A0EQK6_ORYRU:P0C585|SSG1_ORYSA:tr|B8XEK3|B8XEK3_ORYSA:tr|D0TZY6|D0TZY6_ORYSI:tr|A0A3Q9T378|A0A3Q9T378_ORYSA:tr|D3U2H9|D3U2H9_ORYSA:tr|B8XEJ7|B8XEJ7_ORYSA:tr|A0A3Q9T3Z7|A0A3Q9T3Z7_ORYSA:tr|B8XEJ2|B8XEJ2_ORYSA:tr|B8XEK2|B8XEK2_ORYSA:tr|A0A076FRI5|A0A076FRI5_ORYSJ:Q42968|SSG1_ORYGL:A2Y8X2|SSG1_ORYSI:tr|C8CBL1|C8CBL1_ORYSJ:tr|B8XEJ8|B8XEJ8_ORYSA:tr|B1B5Z0|B1B5Z0_ORYSI:tr|B1B5Z1|B1B5Z1_ORYSI:tr|A0A0D9WLF6|A0A0D9WLF6_9ORYZ:tr|A0A0E0A4K1|A0A0E0A4K1_9ORYZ
SAPIYTQPRH	40.04	1168.5989	10	585.3069	5.73	Glucose-1-phosphate adenylyltransferase	tr|D4AIA3|D4AIA3_ORYSI:tr|B7EVB8|B7EVB8_ORYSJ:P15280-2|GLGS2_ORYSJ:tr|A0A0D3GZB2|A0A0D3GZB2_9ORYZ:tr|A0A0E0H7V9|A0A0E0H7V9_ORYNI:tr|A0A0E0LU24|A0A0E0LU24_ORYPU:tr|A0A0E0QHR8|A0A0E0QHR8_ORYRU:tr|B8XED8|B8XED8_ORYSA:tr|B8XED9|B8XED9_ORYSI:tr|B8XED7|B8XED7_ORYSI:tr|B8XEE8|B8XEE8_ORYSA:tr|B8XEF0|B8XEF0_ORYSI:tr|B8XEF2|B8XEF2_ORYSI:tr|B8XEE9|B8XEE9_ORYSI:tr|B8XEE1|B8XEE1_ORYSI:tr|B8XEE2|B8XEE2_ORYSJ:tr|B8XEE5|B8XEE5_ORYSJ:tr|B8XEE6|B8XEE6_ORYSA:tr|A0A0E0AUC9|A0A0E0AUC9_9ORYZ:tr|I1QHZ2|I1QHZ2_ORYGL:tr|A2YU91|A2YU91_ORYSI:P15280|GLGS2_ORYSJ:tr|A0A0D3GZB1|A0A0D3GZB1_9ORYZ:tr|J3MSE0|J3MSE0_ORYBR:tr|A0A0D9X6Z4|A0A0D9X6Z4_9ORYZ:tr|A0A0D9X6Z3|A0A0D9X6Z3_9ORYZ:tr|A0A0E0H7V8|A0A0E0H7V8_ORYNI:tr|A0A0E0QHR7|A0A0E0QHR7_ORYRU:tr|A0A0E0LU23|A0A0E0LU23_ORYPU
KGGIPIGIGK	38.46	938.5912	10	470.3026	11.78	Glucose-1-phosphate adenylyltransferase	tr|D4AIA3|D4AIA3_ORYSI:tr|B7EVB8|B7EVB8_ORYSJ:P15280-2|GLGS2_ORYSJ:tr|A0A0D3GZB2|A0A0D3GZB2_9ORYZ:tr|A0A0E0H7V9|A0A0E0H7V9_ORYNI:tr|A0A0E0LU24|A0A0E0LU24_ORYPU:tr|A0A0E0QHR8|A0A0E0QHR8_ORYRU:tr|B8XED8|B8XED8_ORYSA:tr|B8XED9|B8XED9_ORYSI:tr|B8XED7|B8XED7_ORYSI:tr|B8XEE8|B8XEE8_ORYSA:tr|B8XEF0|B8XEF0_ORYSI:tr|B8XEF2|B8XEF2_ORYSI:tr|B8XEE9|B8XEE9_ORYSI:tr|B8XEE1|B8XEE1_ORYSI:tr|B8XEE2|B8XEE2_ORYSJ:tr|B8XEE5|B8XEE5_ORYSJ:tr|B8XEE6|B8XEE6_ORYSA:tr|A0A0E0AUC9|A0A0E0AUC9_9ORYZ:tr|I1QHZ2|I1QHZ2_ORYGL:tr|A2YU91|A2YU91_ORYSI:P15280|GLGS2_ORYSJ:tr|A0A0D3GZB1|A0A0D3GZB1_9ORYZ:tr|J3MSE0|J3MSE0_ORYBR:tr|A0A0D9X6Z4|A0A0D9X6Z4_9ORYZ:tr|A0A0D9X6Z3|A0A0D9X6Z3_9ORYZ:tr|A0A0E0H7V8|A0A0E0H7V8_ORYNI:tr|A0A0E0QHR7|A0A0E0QHR7_ORYRU:tr|A0A0E0LU23|A0A0E0LU23_ORYPU
AFEPIRSVR	35.81	1073.5981	9	537.8065	10.95	Glutelin	tr|T1T4G3|T1T4G3_ORYSI:tr|T1T4Y4|T1T4Y4_ORYSI:tr|J3L4C0|J3L4C0_ORYBR:tr|A0A0E0JP14|A0A0E0JP14_ORYPU:tr|A1YQG5|A1YQG5_ORYSJ:tr|I1NRU9|I1NRU9_ORYGL:tr|A1YQG3|A1YQG3_ORYSJ:tr|A0A0E0N2T5|A0A0E0N2T5_ORYRU:P07730|GLUA2_ORYSJ:tr|A2WVB9|A2WVB9_ORYSI:tr|A2Z708|A2Z708_ORYSI:P07728|GLUA1_ORYSJ:tr|I1QU95|I1QU95_ORYGL:tr|A0A0E0M7E8|A0A0E0M7E8_ORYPU:tr|A0A0E0FTI2|A0A0E0FTI2_ORYNI:tr|A0A0D9YFB1|A0A0D9YFB1_9ORYZ:tr|A0A0E0C

							821|A0A0E0C821_9ORYZ:tr|A0A0D3EUB5|A0A0D3EUB5_9ORYZ:tr|Q0JJ36|Q0JJ36_ORYSJ:tr|Q40689|Q40689_ORYSA:tr|A0A0E0IRV1|A0A0E0IRV1_ORYNI:tr|A0A0E0QYR5|A0A0E0QYR5_ORYRU:tr|A0A0E0EXG8|A0A0E0EXG8_9ORYZ:tr|A0A0E0QYR6|A0A0E0QYR6_ORYRU:tr|A0A0D3HDD5|A0A0D3HDD5_9ORYZ:tr|A0A0E0IRV2|A0A0E0IRV2_ORYNI:tr|A0A0E0BA63|A0A0E0BA63_9ORYZ:tr|A0A0E0BA64|A0A0E0BA64_9ORYZ
KDKNKPIIF	28.76	1101.6545	9	551.8353	9.92	Sucrose synthase	tr|A0A0P0WTZ1|A0A0P0WTZ1_ORYSJ:tr|A0A0D9WMW0|A0A0D9WMW0_9ORYZ:tr|A0A0E0HM40|A0A0E0HM40_ORYNI:tr|A2YA91|A2YA91_ORYSI:tr|A0A0D3GDS1|A0A0D3GDS1_9ORYZ:tr|I1Q096|I1Q096_ORYGL:tr|A0A0E0A682|A0A0E0A682_9ORYZ:tr|I3QD82|I3QD82_ORYSJ:tr|A0A0E0PUM1|A0A0E0PUM1_ORYRU:P30298|SUS2_ORYSJ:tr|A0A0E0L8X0|A0A0E0L8X0_ORYPU:tr|A0A0E0HM39|A0A0E0HM39_ORYNI:tr|A0A0E0HM38|A0A0E0HM38_ORYNI
SPFRVPIA	28.22	885.5072	8	443.7605	19.96	Pyruvate, phosphate dikinase	tr|J3M716|J3M716_ORYBR:tr|B9FPJ4|B9FPJ4_ORYSJ:tr|A0A0E0PMB6|A0A0E0PMB6_ORYRU:tr|B8AYC1|B8AYC1_ORYSI:tr|A0A0D9ZZ05|A0A0D9ZZ05_9ORYZ:Q6AVA8-2|PPDK1_ORYSJ:tr|A0A0E0DR93|A0A0E0DR93_9ORYZ:tr|A0A0D9VU47|A0A0D9VU47_9ORYZ:tr|J3LQ10|J3LQ10_ORYBR:tr|O82032|O82032_ORYSI:tr|A0A0D9Z998|A0A0D9Z998_9ORYZ:Q75KR1|PPDK2_ORYSJ:tr|A0A0E0NWX2|A0A0E0NWX2_ORYRU:tr|A0A0D3FK58|A0A0D3FK58_9ORYZ:tr|I1PCI9|I1PCI9_ORYGL:tr|A2XIA2|A2XIA2_ORYSI:tr|A0A0D3G7H5|A0A0D3G7H5_9ORYZ:tr|A0A0D9WGC7|A0A0D9WGC7_9ORYZ:tr|A0A0D9WGC6|A0A0D9WGC6_9ORYZ:Q6AVA8|PPDK1_ORYSJ:tr|I1PVJ3|I1PVJ3_ORYGL:tr|A0A0E0ITX7|A0A0E0ITX7_ORYNI:tr|A0A0E0ITX8|A0A0E0ITX8_ORYNI
SDKGRFF	26.51	855.4238	7	428.7193	9.56	Os02g0519900 protein	tr|A0A0P0VJL8|A0A0P0VJL8_ORYSJ:tr|A0A0P0W6A1|A0A0P0W6A1_ORYSJ:tr|A0A0D9VGF7|A0A0D9VGF7_9ORYZ:tr|A0A0D3FS13|A0A0D3FS13_9ORYZ:tr|Q01MK8|Q01MK8_ORYSA:tr|A0A0E0DCG7|A0A0E0DCG7_9ORYZ:tr|A0A0D9YSX8|A0A0D9YSX8_9ORYZ:tr|A0A0D9ZGI8|A0A0D9ZGI8_9ORYZ:tr|A0A0E0GX73|A0A0E0GX73_ORYNI:tr|A2X5F3|A2X5F3_ORYSI:tr|A0A0E0NFL3|A0A0E0NFL3_ORYRU:tr|A0A0E0KM97|A0A0E0KM97_ORYPU:tr|A0A0D3F5Q7|A0A0D3F5Q7_9ORYZ:tr|A0A0E0P4M4|A0A0E0P4M4_ORYRU:tr|A0A0E0G7F6|A0A0E0G7F6_ORYNI:tr|Q7XTK1|Q7XTK1_ORYSJ:tr|Q6H4L2|Q6H4L2_ORYSJ:tr|A0A0D9YEC3|A0A0D9YEC3_9ORYZ:tr|A0A0D3ETH6|A0A0D3ETH6_9ORYZ:tr|A2WUL5|A2WUL5_ORYSI:tr|A0A0E0C7D6|A0A0E0C7D6_9ORYZ:tr|A0A0E0FSG8|A0A0E0FSG8_ORYNI:tr|A0A0E0N1V6|A0A0E0N1V6_ORYRU:tr|Q8W0C4|Q8W0C4_ORYSJ:tr|A0A0E0JNF3|A0A0E0JNF3_ORYPU:tr|A2ZXD6|A2ZXD6_ORYSJ:tr|A0A0E0DA54|A0A0E0DA54_9ORYZ:tr|A0A0E0DA52|A0A0E0DA52_9ORYZ:tr|A0A0E0DA53|A0A0E0DA53_9ORYZ

Sub-fraction n. 14. Total: 29 identified peptides, 25 peptides with rice protein accession. RT, retention time.

<table>
<tr><th>Peptide</th><th>-10lgP</th><th>Mass (Da)</th><th>Length (amino acid n.)</th><th>m/z</th><th>RT (min)</th><th>Protein</th><th>Accession</th></tr>
<tr><td>HGAFTPR</td><td>46.59</td><td>784.3980</td><td>7</td><td>393.2062</td><td>5.39</td><td>Glutelin</td><td>tr|B8AEZ5|B8AEZ5_ORYSI:tr|T1T6C4|T1T6C4_ORYSI:tr|A0A0E0CIL1|A0A0E0CIL1_9ORYZ:tr|A0A0E0G6R1|A0A0E0G6R1_ORYNI:tr|A0A0D9YPX0|A0A0D9YPX0_9ORYZ:tr|A0A0D3F336|A0A0D3F336_9ORYZ:tr|I1NZ10|I1NZ10_ORYGL:tr|T1T4F0|T1T4F0_ORYSI:tr|A0A0E0CIL2|A0A0E0CIL2_9ORYZ:tr|B9F4T3|B9F4T3_ORYSJ:tr|A0A0E0NCF3|A0A0E0NCF3_ORYRU:P14323|GLUB1_ORYSJ:tr|Q0E2D2|Q0E2D2_ORYSJ</td></tr>
<tr><td>NLNNNPYFKGT</td><td>45.59</td><td>1280.6150</td><td>11</td><td>641.3158</td><td>14.19</td><td>Granule-bound starch synthase I</td><td>tr|A0A0H4BM25|A0A0H4BM25_ORYSI:tr|A0A0H4BFT9|A0A0H4BFT9_ORYSI:tr|A0A0E0DW79|A0A0E0DW79_9ORYZ:tr|A8QXE7|A8QXE7_ORYSI:tr|V5NEJ7|V5NEJ7_ORYSA:tr|A0A0E0DW80|A0A0E0DW80_9ORYZ:tr|A0EQH2|A0EQH2_ORYSJ:tr|A0EQK7|A0EQK7_ORYRU:tr|A0EQD4|A0EQD4_ORYSI:tr|A0EQK4|A0EQK4_ORYRU:tr|A0EQG8|A0EQG8_ORYSJ:tr|A0EQE0|A0EQE0_ORYSA:tr|A0EQK5|A0EQK5_ORYRU:tr|A0EQK6|A0EQK6_ORYRU:P0C585|SSG1_ORYSA:tr|B8XEK3|B8XEK3_ORYSA:tr|D0TZY6|D0TZY6_ORYSI:tr|A0A3Q9T378|A0A3Q9T378_ORYSA:tr|D3U2H9|D3U2H9_ORYSA:tr|B8XEJ7|B8XEJ7_ORYSA:tr|A0A3Q9T3Z7|A0A3Q9T3Z7_ORYSA:tr|B8XEJ2|B8XEJ2_ORYSA:tr|B8XEK2|B8XEK2_ORYSA:tr|A0A076FRI5|A0A076FRI5_ORYSJ:Q42968|SSG1_ORYGL:A2Y8X2|SSG1_ORYSI:tr|C8CBL1|C8CBL1_ORYSJ:tr|B8XEJ8|B8XEJ8_ORYSA:tr|B1B5Z0|B1B5Z0_ORYSI:tr|B1B5Z1|B1B5Z1_ORYSI:tr|A0A0D9WLF6|A0A0D9WLF6_9ORYZ:tr|A0A0E0A4K1|A0A0E0A4K1_9ORYZ</td></tr>
<tr><td>APIYTQPR</td><td>41.19</td><td>944.5079</td><td>8</td><td>473.2611</td><td>7.57</td><td>Glucose-1-phosphate adenylyltransferase</td><td>tr|D4AIA3|D4AIA3_ORYSI:tr|B7EVB8|B7EVB8_ORYSJ:P15280-2|GLGS2_ORYSJ:tr|A0A0D3GZB2|A0A0D3GZB2_9ORYZ:tr|A0A0E0H7V9|A0A0E0H7V9_ORYNI:tr|A0A0E0LU24|A0A0E0LU24_ORYPU:tr|A0A0E0QHR8|A0A0E0QHR8_ORYRU:tr|B8XED8|B8XED8_ORYSA:tr|B8XED9|B8XED9_ORYSI:tr|B8XED7|B8XED7_ORYSI:tr|B8XEE8|B8XEE8_ORYSA:tr|B8XEF0|B8XEF0_ORYSI:tr|B8XEF2|B8XEF2_ORYSI:tr|B8XEE9|B8XEE9_ORYSI:tr|B8XEE1|B8XEE1_ORYSI:tr|B8XEE2|B8XEE2_ORYSJ:tr|B8XEE5|B8XEE5_ORYSJ:tr|B8XEE6|B8XEE6_ORYSA:tr|A2YU91|A2YU91_ORYSI:P15280|GLGS2_ORYSJ:tr|A0A0E0AUC9|A0A0E0AUC9_9ORYZ:tr|I1QHZ2|I1QHZ2_ORYGL:tr|A0A0D3GZB1|A0A0D3GZB1_9ORYZ:tr|J3MSE0|J3MSE0_ORYBR:tr|A0A0D9X6Z4|A0A0D9X6Z4_9ORYZ:tr|A0A0D9X6Z3|A0A0D9X6Z3_9ORYZ:tr|A0A0E0H7V8|A0A0E0H7V8_ORYNI:tr|A0A0E0QHR7|A0A0E0QHR7_ORYRU:tr|A0A0E0LU23|A0A0E0LU23_ORYPU</td></tr>
<tr><td>SAPIYTQPRH</td><td>40.52</td><td>1168.5989</td><td>10</td><td>585.3063</td><td>6.07</td><td>Glucose-1-phosphate adenylyltransferase</td><td>tr|D4AIA3|D4AIA3_ORYSI:tr|B7EVB8|B7EVB8_ORYSJ:P15280-2|GLGS2_ORYSJ:tr|A0A0D3GZB2|A0A0D3GZB2_9ORYZ:tr|A0A0E0H7V9|A0A0E0H7V9_ORYNI:tr|A0A0E0LU24|A0A0E0LU24_ORYPU:tr|A0A0E0QHR8|A0A0E0QHR8_ORYRU:tr|B8XED8|B8XED8_ORYSA:tr|B8XED9|B8XED9_ORYSI:tr|B8XED7|B8XED7_ORYSI:tr|B8XEE8|B8XEE8_ORYSA:tr|</td></tr>
</table>

<table>
<tr><td></td><td></td><td></td><td></td><td></td><td></td><td></td><td>B8XEF0|B8XEF0_ORYSI:tr|B8XEF2|B8XEF2_ORYSI:tr|B8XEE9|B8XEE9_ORYSI:tr|B8XEE1|B8XEE1_ORYSI:tr|B8XEE2|B8XEE2_ORYSJ:tr|B8XEE5|B8XEE5_ORYSJ:tr|B8XEE6|B8XEE6_ORYSA:tr|A2YU91|A2YU91_ORYSI:P15280|GLGS2_ORYSJ:tr|A0A0E0AUC9|A0A0E0AUC9_9ORYZ:tr|I1QHZ2|I1QHZ2_ORYGL:tr|A0A0D3GZB1|A0A0D3GZB1_9ORYZ:tr|J3MSE0|J3MSE0_ORYBR:tr|A0A0D9X6Z4|A0A0D9X6Z4_9ORYZ:tr|A0A0D9X6Z3|A0A0D9X6Z3_9ORYZ:tr|A0A0E0H7V8|A0A0E0H7V8_ORYNI:tr|A0A0E0QHR7|A0A0E0QHR7_ORYRU:tr|A0A0E0LU23|A0A0E0LU23_ORYPU</td></tr>
<tr><td>AFEPIRSVR</td><td>40.11</td><td>1073.5981</td><td>9</td><td>537.8064</td><td>11.12</td><td>Glutelin</td><td>tr|T1T4G3|T1T4G3_ORYSI:tr|T1T4Y4|T1T4Y4_ORYSI:tr|J3L4C0|J3L4C0_ORYBR:tr|A0A0E0JP14|A0A0E0JP14_ORYPU:tr|A1YQG5|A1YQG5_ORYSJ:tr|I1NRU9|I1NRU9_ORYGL:tr|A1YQG3|A1YQG3_ORYSJ:tr|A0A0E0N2T5|A0A0E0N2T5_ORYRU:P07730|GLUA2_ORYSJ:tr|A2WVB9|A2WVB9_ORYSI:tr|A2Z708|A2Z708_ORYSI:P07728|GLUA1_ORYSJ:tr|I1QU95|I1QU95_ORYGL:tr|A0A0E0M7E8|A0A0E0M7E8_ORYPU:tr|A0A0E0FTI2|A0A0E0FTI2_ORYNI:tr|A0A0D9YFB1|A0A0D9YFB1_9ORYZ:tr|A0A0E0C821|A0A0E0C821_9ORYZ:tr|A0A0D3EUB5|A0A0D3EUB5_9ORYZ:tr|Q0JJ36|Q0JJ36_ORYSJ:tr|Q40689|Q40689_ORYSA:tr|A0A0E0IRV1|A0A0E0IRV1_ORYNI:tr|A0A0E0QYR5|A0A0E0QYR5_ORYRU:tr|A0A0E0EXG8|A0A0E0EXG8_9ORYZ:tr|A0A0E0QYR6|A0A0E0QYR6_ORYRU:tr|A0A0D3HDD5|A0A0D3HDD5_9ORYZ:tr|A0A0E0IRV2|A0A0E0IRV2_ORYNI:tr|A0A0E0BA63|A0A0E0BA63_9ORYZ:tr|A0A0E0BA64|A0A0E0BA64_9ORYZ</td></tr>
<tr><td>KGGIPIGIGK</td><td>38.70</td><td>938.5912</td><td>10</td><td>470.3036</td><td>11.92</td><td>Glucose-1-phosphate adenylyltransferase</td><td>tr|D4AIA3|D4AIA3_ORYSI:tr|B7EVB8|B7EVB8_ORYSJ:P15280-2|GLGS2_ORYSJ:tr|A0A0D3GZB2|A0A0D3GZB2_9ORYZ:tr|A0A0E0H7V9|A0A0E0H7V9_ORYNI:tr|A0A0E0LU24|A0A0E0LU24_ORYPU:tr|A0A0E0QHR8|A0A0E0QHR8_ORYRU:tr|B8XED8|B8XED8_ORYSA:tr|B8XED9|B8XED9_ORYSI:tr|B8XED7|B8XED7_ORYSI:tr|B8XEE8|B8XEE8_ORYSA:tr|B8XEF0|B8XEF0_ORYSI:tr|B8XEF2|B8XEF2_ORYSI:tr|B8XEE9|B8XEE9_ORYSI:tr|B8XEE1|B8XEE1_ORYSI:tr|B8XEE2|B8XEE2_ORYSJ:tr|B8XEE5|B8XEE5_ORYSJ:tr|B8XEE6|B8XEE6_ORYSA:tr|A2YU91|A2YU91_ORYSI:P15280|GLGS2_ORYSJ:tr|A0A0E0AUC9|A0A0E0AUC9_9ORYZ:tr|I1QHZ2|I1QHZ2_ORYGL:tr|A0A0D3GZB1|A0A0D3GZB1_9ORYZ:tr|J3MSE0|J3MSE0_ORYBR:tr|A0A0D9X6Z4|A0A0D9X6Z4_9ORYZ:tr|A0A0D9X6Z3|A0A0D9X6Z3_9ORYZ:tr|A0A0E0H7V8|A0A0E0H7V8_ORYNI:tr|A0A0E0QHR7|A0A0E0QHR7_ORYRU:tr|A0A0E0LU23|A0A0E0LU23_ORYPU</td></tr>
<tr><td>RGVIGGGGY</td><td>33.85</td><td>834.4348</td><td>9</td><td>418.2255</td><td>9.78</td><td>Oleosin</td><td>tr|S4U0K5|S4U0K5_ORYSJ:tr|I1PNK0|I1PNK0_ORYGL:tr|A0A0E0PCA2|A0A0E0PCA2_ORYRU:tr|Q01J14|Q01J14_ORYSA:tr|A0A0D9ZNU6|A0A0D9ZNU6_9ORYZ:tr|A0A0E0DGQ5|A0A0E0DGQ5_9ORYZ:tr|A0A0D3FYL2|A0A0D3FYL2_9ORYZ:tr|A2XW46|A2XW46_ORYSI:tr|A0A0E0H3U9|A0A0E0H3U9_ORYNI:Q42980|OLEO1_ORYSJ</td></tr>
<tr><td>TVFNGVLRPG</td><td>33.70</td><td>1058.5873</td><td>10</td><td>530.3011</td><td>18.18</td><td>Glutelin</td><td>tr|A2X399|A2X399_ORYSI:P14614|GLUB4_ORYSJ:tr|Q0E261|Q0E261_ORYSJ:tr|A0A0D9YQ79|A0A0D9YQ79_9ORYZ:tr|D6BV14|D6BV14_ORYSJ:tr|A0A0D3F3E6|A0A0D3F3E6_9ORYZ:tr|A0A0E0JY90|A0A0E0JY90_ORYPU:Q6ERU3|GLUB5_ORYSJ:tr|I1NZ94|I1NZ94_ORYGL</td></tr>
</table>

KDKNKPIIF	31.51	1101.6545	9	551.8352	10.28	Sucrose synthase	tr|A0A0P0WTZ1|A0A0P0WTZ1_ORYSJ:tr|A0A0D9WMW0|A0A0D9WMW0_9ORYZ:tr|A0A0E0HM40|A0A0E0HM40_ORYNI:tr|A2YA91|A2YA91_ORYSI:tr|A0A0D3GDS1|A0A0D3GDS1_9ORYZ:tr|I1Q096|I1Q096_ORYGL:tr|A0A0E0A682|A0A0E0A682_9ORYZ:tr|I3QD82|I3QD82_ORYSJ:tr|A0A0E0PUM1|A0A0E0PUM1_ORYRU:P30298|SUS2_ORYSJ:tr|A0A0E0L8X0|A0A0E0L8X0_ORYPU:tr|A0A0E0HM39|A0A0E0HM39_ORYNI:tr|A0A0E0HM38|A0A0E0HM38_ORYNI
YTNTPGVVY(+15.01)I	31.27	1140.5815	10	571.2977	10.20	Glutelin	tr|B8AEZ5|B8AEZ5_ORYSI:tr|T1T6C4|T1T6C4_ORYSI:tr|A0A0E0CIL1|A0A0E0CIL1_9ORYZ:tr|A0A0E0G6R1|A0A0E0G6R1_ORYNI:tr|A0A0D9YPX0|A0A0D9YPX0_9ORYZ:tr|A0A0D3F336|A0A0D3F336_9ORYZ:tr|I1NZ10|I1NZ10_ORYGL:tr|T1T4F0|T1T4F0_ORYSI:tr|A0A0E0CIL2|A0A0E0CIL2_9ORYZ
GIPHLRPA	31.08	859.5028	8	430.7582	9.47	Os08g0156800 protein	tr|A0A0D9W5T2|A0A0D9W5T2_9ORYZ:tr|A0A0E0AR03|A0A0E0AR03_9ORYZ:tr|A0A0E0AVT1|A0A0E0AVT1_9ORYZ:tr|A0A0E0QEJ1|A0A0E0QEJ1_ORYRU:tr|J3MQI5|J3MQI5_ORYBR:tr|A0A0E0EI29|A0A0E0EI29_9ORYZ:tr|A0A0E0QJG8|A0A0E0QJG8_ORYRU:tr|A2YVK0|A2YVK0_ORYSI:tr|A0A0E0LV84|A0A0E0LV84_ORYPU:tr|I1QFS9|I1QFS9_ORYGL:tr|A0A0E0LRE5|A0A0E0LRE5_ORYPU:tr|A0A0D3H0M8|A0A0D3H0M8_9ORYZ:tr|Q6ZD96|Q6ZD96_ORYSJ:tr|A0A0E0ICC0|A0A0E0ICC0_ORYNI:tr|Q6Z537|Q6Z537_ORYSJ:tr|A0A0D3GWH8|A0A0D3GWH8_9ORYZ:tr|A0A0E0IJH7|A0A0E0IJH7_ORYNI:tr|A0A0E0M164|A0A0E0M164_ORYPU:tr|A0A0E0QRD4|A0A0E0QRD4_ORYRU:tr|A3BYS9|A3BYS9_ORYSJ:tr|A0A0E0I7D7|A0A0E0I7D7_ORYNI:tr|A0A0E0ES62|A0A0E0ES62_9ORYZ:tr|J3LYK0|J3LYK0_ORYBR:tr|A2Z177|A2Z177_ORYSI:tr|A0A0D9X4K7|A0A0D9X4K7_9ORYZ:tr|A0A0E0KRG3|A0A0E0KRG3_ORYPU:tr|A0A0D3H6U3|A0A0D3H6U3_9ORYZ:tr|J3MXE6|J3MXE6_ORYBR:tr|A0A0E0B2W8|A0A0E0B2W8_9ORYZ:tr|A0A0E0ELY5|A0A0E0ELY5_9ORYZ
H(+154.10)GAFTPR	30.62	938.4974	7	470.2563	16.74	Glutelin	tr|B8AEZ5|B8AEZ5_ORYSI:tr|T1T6C4|T1T6C4_ORYSI:tr|A0A0E0CIL1|A0A0E0CIL1_9ORYZ:tr|A0A0E0G6R1|A0A0E0G6R1_ORYNI:tr|A0A0D9YPX0|A0A0D9YPX0_9ORYZ:tr|A0A0D3F336|A0A0D3F336_9ORYZ:tr|I1NZ10|I1NZ10_ORYGL:tr|T1T4F0|T1T4F0_ORYSI:tr|A0A0E0CIL2|A0A0E0CIL2_9ORYZ:tr|B9F4T3|B9F4T3_ORYSJ:tr|A0A0E0NCF3|A0A0E0NCF3_ORYRU:P14323|GLUB1_ORYSJ:tr|Q0E2D2|Q0E2D2_ORYSJ
RNNQVWQQ	29.97	1071.5210	8	536.7670	7.67	Os05g0328333 protein	tr|A0A0P0WKT5|A0A0P0WKT5_ORYSJ:tr|Q5W6A6|Q5W6A6_ORYSJ:tr|E5D3L5|E5D3L5_ORYSJ:tr|Q40730|Q40730_ORYSA:tr|E5D3L6|E5D3L6_ORYSJ:tr|C7J346|C7J346_ORYSJ:tr|A0A0P0WKY1|A0A0P0WKY1_ORYSJ:tr|A0A0P0WKV9|A0A0P0WKV9_ORYSJ:tr|Q5W6A1|Q5W6A1_ORYSJ:tr|A0A0D3G667|A0A0D3G667_9ORYZ:tr|Q5W743|Q5W743_ORYSJ:tr|Q5W695|Q5W695_ORYSJ:tr|Q43603|Q43603_ORYSA:tr|P93412|P93412_ORYSJ:tr|Q0DJ44|Q0DJ44_ORYSJ:tr|A0A0P0WKT6|A0A0P0WKT6_ORYSJ:tr|E0X6Y1|E0X6Y1_ORYSJ:tr|Q5W755|Q5W755_ORYSJ:P0C5E5|PRO7_ORYSI:tr|A1YQE8|A1YQE8_ORYSJ:tr|Q5W6A3|Q5W6A3_ORYSJ:tr|A0A0E0PKU8|A0A0E0PKU8_ORYRU:Q0DJ45|PRO7_ORYSJ:tr|A0A0E0DQ09|A0A0E0DQ09_9ORYZ:tr|A0A0E0PKV4|A0A0E0PKV4_ORYRU:tr|A0A0N7

							KKJ8|A0A0N7KKJ8_ORYSJ:tr|Q0DJ38|Q0DJ38_ORYSJ:tr|A1YQF0|A1YQF0_ORYSJ:tr|A0A0D3G661|A0A0D3G661_9ORYZ
TNPWHSPR	29.95	993.4780	8	497.7459	6.08	Glutelin	tr|B8AEZ5|B8AEZ5_ORYSI:tr|T1T6C4|T1T6C4_ORYSI:tr|A0A0E0CIL1|A0A0E0CIL1_9ORYZ:tr|A0A0E0G6R1|A0A0E0G6R1_ORYNI:tr|A0A0D9YPX0|A0A0D9YPX0_9ORYZ:tr|A0A0D3F336|A0A0D3F336_9ORYZ:tr|I1NZ10|I1NZ10_ORYGL:tr|T1T4F0|T1T4F0_ORYSI:tr|A0A0E0CIL2|A0A0E0CIL2_9ORYZ:tr|B9F4T3|B9F4T3_ORYSJ:tr|A0A0E0NCF3|A0A0E0NCF3_ORYRU:P14323|GLUB1_ORYSJ:tr|Q0E2D2|Q0E2D2_ORYSJ
IGNPHLR	29.25	805.4559	7	403.7346	5.53	Putative mitochondrial energy transfer protein	tr|J3LAP3|J3LAP3_ORYBR:tr|A0A0D3F250|A0A0D3F250_9ORYZ:tr|A0A0E0JX14|A0A0E0JX14_ORYPU:tr|A2X256|A2X256_ORYSI:tr|A3A4A0|A3A4A0_ORYSJ:tr|A0A0E0NY14|A0A0E0NY14_ORYRU:tr|A0A0E0G3F4|A0A0E0G3F4_ORYNI:tr|A0A0D9YNX3|A0A0D9YNX3_9ORYZ:tr|A2YER7|A2YER7_ORYSI:tr|Q69XJ8|Q69XJ8_ORYSJ:tr|A0A0E0ABK1|A0A0E0ABK1_9ORYZ:tr|A0A0P0WYD6|A0A0P0WYD6_ORYSJ:tr|A0A0D9WRV1|A0A0D9WRV1_9ORYZ:tr|A0A0E0E3L8|A0A0E0E3L8_9ORYZ:tr|A0A0E0LD95|A0A0E0LD95_ORYPU:tr|I1Q3G0|I1Q3G0_ORYGL:tr|A0A0E0PZV6|A0A0E0PZV6_ORYRU:tr|A0A0E0HT75|A0A0E0HT75_ORYNI:tr|J3MFK2|J3MFK2_ORYBR:tr|A0A0D3GIF4|A0A0D3GIF4_9ORYZ:tr|Q6Z782|Q6Z782_ORYSJ:tr|I1NYC8|I1NYC8_ORYGL
SDKGRFF	28.69	855.4238	7	428.7198	9.78	Os02g0519900 protein	tr|A0A0P0VJL8|A0A0P0VJL8_ORYSJ:tr|A0A0P0W6A1|A0A0P0W6A1_ORYSJ:tr|A0A0D9VGF7|A0A0D9VGF7_9ORYZ:tr|A0A0D3FS13|A0A0D3FS13_9ORYZ:tr|Q01MK8|Q01MK8_ORYSA:tr|A0A0E0DCG7|A0A0E0DCG7_9ORYZ:tr|A0A0D9YSX8|A0A0D9YSX8_9ORYZ:tr|A0A0D9ZGI8|A0A0D9ZGI8_9ORYZ:tr|A0A0E0GX73|A0A0E0GX73_ORYNI:tr|A2X5F3|A2X5F3_ORYSI:tr|A0A0E0NFL3|A0A0E0NFL3_ORYRU:tr|A0A0E0KM97|A0A0E0KM97_ORYPU:tr|A0A0D3F5Q7|A0A0D3F5Q7_9ORYZ:tr|A0A0E0P4M4|A0A0E0P4M4_ORYRU:tr|A0A0E0G7F6|A0A0E0G7F6_ORYNI:tr|Q7XTK1|Q7XTK1_ORYSJ:tr|Q6H4L2|Q6H4L2_ORYSJ:tr|A0A0E0DA54|A0A0E0DA54_9ORYZ:tr|A0A0E0DA52|A0A0E0DA52_9ORYZ:tr|A0A0E0DA53|A0A0E0DA53_9ORYZ
FNGVLRPG	28.00	858.4711	8	430.2435	12.56	Glutelin	tr|A2X399|A2X399_ORYSI:P14614|GLUB4_ORYSJ:tr|Q0E261|Q0E261_ORYSJ:tr|A0A0D9YQ79|A0A0D9YQ79_9ORYZ:tr|D6BV14|D6BV14_ORYSJ:tr|A0A0D3F3E6|A0A0D3F3E6_9ORYZ:tr|A0A0E0JY90|A0A0E0JY90_ORYPU:Q6ERU3|GLUB5_ORYSJ:tr|I1NZ94|I1NZ94_ORYGL
KQLQPTW	27.75	899.4865	7	450.7509	13.62	Glutelin	tr|B9EZT3|B9EZT3_ORYSJ:tr|B8AH66|B8AH66_ORYSI:tr|A0A0E0JZG5|A0A0E0JZG5_ORYPU:tr|A0A0E0G669|A0A0E0G669_ORYNI:tr|I1P037|I1P037_ORYGL:tr|Q6K7K6|Q6K7K6_ORYSJ:tr|A0A0D9YRU4|A0A0D9YRU4_9ORYZ:tr|Q9ZWJ8|Q9ZWJ8_ORYSA:tr|A0A0E0NED3|A0A0E0NED3_ORYRU
YSNTPGMVYH(sub I)	27.19	1167.5020	10	584.7568	11.40	Glutelin	tr|A2X399|A2X399_ORYSI:P14614|GLUB4_ORYSJ:tr|Q0E261|Q0E261_ORYSJ:tr|A0A0D9YQ79|A0A0D9YQ79_9ORYZ:tr|D6BV14|D6BV14_ORY

<table>
<tr><td></td><td></td><td></td><td></td><td></td><td></td><td></td><td>SJ:tr|A0A0D3F3E6|A0A0D3F3E6_9ORYZ:tr|A0A0E0JY90|A0A0E0JY90_ORYPU:Q6ERU3|GLUB5_ORYSJ:tr|I1NZ94|I1NZ94_ORYGL</td></tr>
<tr><td>SPFRVPIA</td><td>27.05</td><td>885.5072</td><td>8</td><td>443.7615</td><td>19.99</td><td>Pyruvate, phosphate dikinase</td><td>tr|J3M716|J3M716_ORYBR:tr|B9FPJ4|B9FPJ4_ORYSJ:tr|A0A0E0PMB6|A0A0E0PMB6_ORYRU:tr|B8AYC1|B8AYC1_ORYSI:tr|A0A0D9ZZ05|A0A0D9ZZ05_9ORYZ:Q6AVA8-2|PPDK1_ORYSJ:tr|A0A0E0DR93|A0A0E0DR93_9ORYZ:tr|A0A0D9VU47|A0A0D9VU47_9ORYZ:tr|J3LQ10|J3LQ10_ORYBR:tr|O82032|O82032_ORYSI:tr|A0A0D9Z998|A0A0D9Z998_9ORYZ:Q75KR1|PPDK2_ORYSJ:tr|A0A0E0NWX2|A0A0E0NWX2_ORYRU:tr|A0A0D3FK58|A0A0D3FK58_9ORYZ:tr|I1PCI9|I1PCI9_ORYGL:tr|A2XIA2|A2XIA2_ORYSI:tr|A0A0D3G7H5|A0A0D3G7H5_9ORYZ:tr|A0A0D9WGC7|A0A0D9WGC7_9ORYZ:tr|A0A0D9WGC6|A0A0D9WGC6_9ORYZ:Q6AVA8|PPDK1_ORYSJ:tr|I1PVJ3|I1PVJ3_ORYGL:tr|A0A0E0ITX7|A0A0E0ITX7_ORYNI:tr|A0A0E0ITX8|A0A0E0ITX8_ORYNI</td></tr>
<tr><td>SLKPLVPR</td><td>27.00</td><td>908.5807</td><td>8</td><td>455.2976</td><td>10.07</td><td>Phosphoglycerate kinase</td><td>tr|A0A0E0GWR2|A0A0E0GWR2_ORYNI:tr|A2YG06|A2YG06_ORYSI:tr|A0A0D3GJQ6|A0A0D3GJQ6_9ORYZ:tr|A0A0E0E560|A0A0E0E560_9ORYZ:tr|A0A0E0AD06|A0A0E0AD06_9ORYZ:tr|Q655T1|Q655T1_ORYSJ:tr|A0A0D9WT19|A0A0D9WT19_9ORYZ:tr|A0A0E0Q1D7|A0A0E0Q1D7_ORYRU:tr|J3MGP8|J3MGP8_ORYBR:tr|A0A0E0LEG8|A0A0E0LEG8_ORYPU:tr|I1Q4G7|I1Q4G7_ORYGL:tr|Q09HR1|Q09HR1_ORYSI:tr|A0A0E0HQB3|A0A0E0HQB3_ORYNI:tr|B8AIH2|B8AIH2_ORYSI:tr|A0A0E0CGA6|A0A0E0CGA6_9ORYZ:tr|A0A0E0NAE9|A0A0E0NAE9_ORYRU:tr|Q09HR2|Q09HR2_ORYSI:tr|J3L9Z3|J3L9Z3_ORYBR:tr|A0A0D9YMV6|A0A0D9YMV6_9ORYZ:tr|I1NXN0|I1NXN0_ORYGL:tr|Q6H6C7|Q6H6C7_ORYSJ:tr|A0A0E0CGA5|A0A0E0CGA5_9ORYZ:tr|A0A0D3F185|A0A0D3F185_9ORYZ:tr|A0A0D9VCN8|A0A0D9VCN8_9ORYZ:tr|A0A0E0JW27|A0A0E0JW27_ORYPU</td></tr>
<tr><td>TNIPGVVYR(sub I)</td><td>26.64</td><td>1017.5607</td><td>9</td><td>509.7878</td><td>15.00</td><td>Glutelin</td><td>tr|B9F4T3|B9F4T3_ORYSJ:tr|A0A0E0NCF3|A0A0E0NCF3_ORYRU:P14323|GLUB1_ORYSJ:tr|Q0E2D2|Q0E2D2_ORYSJ</td></tr>
<tr><td>NNNPYFK</td><td>26.14</td><td>895.4188</td><td>7</td><td>448.7166</td><td>8.48</td><td>Granule-bound starch synthase I</td><td>tr|A0A0H4BM25|A0A0H4BM25_ORYSI:tr|A0A0H4BFT9|A0A0H4BFT9_ORYSI:tr|A0A0E0DW79|A0A0E0DW79_9ORYZ:tr|A8QXE7|A8QXE7_ORYSI:tr|V5NEJ7|V5NEJ7_ORYSA:tr|A0A0E0DW80|A0A0E0DW80_9ORYZ:tr|A0EQH2|A0EQH2_ORYSJ:tr|A0EQK7|A0EQK7_ORYRU:tr|A0EQD4|A0EQD4_ORYSI:tr|A0EQK4|A0EQK4_ORYRU:tr|A0EQG8|A0EQG8_ORYSJ:tr|A0EQE0|A0EQE0_ORYSA:tr|A0EQK5|A0EQK5_ORYRU:tr|A0EQK6|A0EQK6_ORYRU:P0C585|SSG1_ORYSA:tr|B8XEK3|B8XEK3_ORYSA:tr|D0TZY6|D0TZY6_ORYSI:tr|A0A3Q9T378|A0A3Q9T378_ORYSA:tr|D3U2H9|D3U2H9_ORYSA:tr|B8XEJ7|B8XEJ7_ORYSA:tr|A0A3Q9T3Z7|A0A3Q9T3Z7_ORYSA:tr|B8XEJ2|B8XEJ2_ORYSA:tr|B8XEK2|B8XEK2_ORYSA:tr|A0A076FRI5|A0A076FRI5_ORYSJ:Q42968|SSG1_ORYGL:A2Y8X2|SSG1_ORYSI:tr|C8CBL1|C8CBL1_ORYSJ:tr|B8XEJ8|B8XEJ8_ORYSA:tr|B1B5Z0|B1B5Z0_ORYSI:tr|B1B5Z1|B1B5Z1_ORYSI:tr|A0A0D9WLF6|A0A0D9WLF6_9ORYZ:tr|A0A0E0A4K1|A0A0E0A4K1_9ORYZ</td></tr>
</table>

FEPIRSVR	23.77	1002.5610	8	502.2881	11.13	Glutelin	tr|T1T4G3|T1T4G3_ORYSI:tr|T1T4Y4|T1T4Y4_ORYSI:tr|J3L4C0|J3L4C0_ORYBR:tr|A0A0E0JP14|A0A0E0JP14_ORYPU:tr|A1YQG5|A1YQG5_ORYSJ:tr|I1NRU9|I1NRU9_ORYGL:tr|A1YQG3|A1YQG3_ORYSJ:tr|A0A0E0N2T5|A0A0E0N2T5_ORYRU:P07730|GLUA2_ORYSJ:tr|A2WVB9|A2WVB9_ORYSI:tr|A2Z708|A2Z708_ORYSI:P07728|GLUA1_ORYSJ:tr|I1QU95|I1QU95_ORYGL:tr|A0A0E0M7E8|A0A0E0M7E8_ORYPU:tr|A0A0E0FTI2|A0A0E0FTI2_ORYNI:tr|A0A0D9YFB1|A0A0D9YFB1_9ORYZ:tr|A0A0E0C821|A0A0E0C821_9ORYZ:tr|A0A0D3EUB5|A0A0D3EUB5_9ORYZ:tr|Q0JJ36|Q0JJ36_ORYSJ:tr|Q40689|Q40689_ORYSA:tr|A0A0E0IRV1|A0A0E0IRV1_ORYNI:tr|A0A0E0QYR5|A0A0E0QYR5_ORYRU:tr|A0A0E0EXG8|A0A0E0EXG8_9ORYZ:tr|A0A0E0QYR6|A0A0E0QYR6_ORYRU:tr|A0A0D3HDD5|A0A0D3HDD5_9ORYZ:tr|A0A0E0IRV2|A0A0E0IRV2_ORYNI:tr|A0A0E0BA63|A0A0E0BA63_9ORYZ:tr|A0A0E0BA64|A0A0E0BA64_9ORYZ
MQRPGTPLY	22.06	1061.5328	9	531.7740	13.29	Os02g0519900 protein	tr|A0A0P0VJL8|A0A0P0VJL8_ORYSJ:tr|A0A0P0W6A1|A0A0P0W6A1_ORYSJ:tr|A0A0D9VGF7|A0A0D9VGF7_9ORYZ:tr|A0A0D3FS13|A0A0D3FS13_9ORYZ:tr|Q01MK8|Q01MK8_ORYSA:tr|A0A0E0DCG7|A0A0E0DCG7_9ORYZ:tr|A0A0D9YSX8|A0A0D9YSX8_9ORYZ:tr|A0A0D9ZGI8|A0A0D9ZGI8_9ORYZ:tr|A0A0E0GX73|A0A0E0GX73_ORYNI:tr|A2X5F3|A2X5F3_ORYSI:tr|A0A0E0NFL3|A0A0E0NFL3_ORYRU:tr|A0A0E0KM97|A0A0E0KM97_ORYPU:tr|A0A0D3F5Q7|A0A0D3F5Q7_9ORYZ:tr|A0A0E0P4M4|A0A0E0P4M4_ORYRU:tr|A0A0E0G7F6|A0A0E0G7F6_ORYNI:tr|Q7XTK1|Q7XTK1_ORYSJ:tr|Q6H4L2|Q6H4L2_ORYSJ:tr|A0A0E0DA54|A0A0E0DA54_9ORYZ:tr|A0A0E0DA52|A0A0E0DA52_9ORYZ:tr|A0A0E0DA53|A0A0E0DA53_9ORYZ

Sub-fraction n. 16. Total: 33 identified peptides, 27 peptides with rice protein accession. RT, retention time.

Peptide	-10lgP	Mass (Da)	Length (amino acid n.)	m/z	RT (min)	Protein	Accession
GNKRNPQAY	44.31	1046.5258	9	524.2699	5.37	Glutelin	tr|T1T4G3|T1T4G3_ORYSI:tr|T1T4Y4|T1T4Y4_ORYSI:tr|A0A0E0JP14|A0A0E0JP14_ORYPU:tr|A1YQG5|A1YQG5_ORYSJ:tr|I1NRU9|I1NRU9_ORYGL:tr|A1YQG3|A1YQG3_ORYSJ:tr|A0A0E0N2T5|A0A0E0N2T5_ORYRU:P07730|GLUA2_ORYSJ:tr|A2WVB9|A2WVB9_ORYSI:tr|A2Z708|A2Z708_ORYSI:P07728|GLUA1_ORYSJ:tr|I1QU95|I1QU95_ORYGL:tr|A0A0E0M7E8|A0A0E0M7E8_ORYPU:tr|A0A0E0FTI2|A0A0E0FTI2_ORYNI:tr|A0A0E0C821|A0A0E0C821_9ORYZ:tr|A0A0D3EUB5|A0A0D3EUB5_9ORYZ:tr|Q0JJ36|Q0JJ36_ORYSJ:tr|Q40689|Q40689_ORYSA:tr|A0A0E0IRV1|A0A0E0IRV1_ORYNI:tr|A0A0E0QYR5|A0A0E0QYR5_ORYRU:tr|A0A0E0QYR6|A0A0E0QYR6_ORYRU:tr|A0A0D3HDD5|A0A0D3HDD5_9ORYZ:tr|A0A0E0IRV2|A0A0E0IRV2_ORYNI:tr|A0A0E0M7E9|A0A0E0M7E9_ORYPU:tr|A0A0E0BA65|A0A0E0BA65_9ORYZ:tr|A0A0E0IRV3|A0A0E0IRV3_ORYNI:tr

							|A0A0D3HDD6|A0A0D3HDD6_9ORYZ:tr|A0A0E0QYR7|A0A0E0QYR7_ORYRU:tr|A0A0E0BA63|A0A0E0BA63_9ORYZ:tr|A0A0E0BA64|A0A0E0BA64_9ORYZ
HGAFTPR	43.68	784.3980	7	393.2063	5.45	Glutelin	tr|A0A0D3F334|A0A0D3F334_9ORYZ:tr|B9F4T1|B9F4T1_ORYSJ:tr|T1T6C4|T1T6C4_ORYSI:tr|I1NZ08|I1NZ08_ORYGL:tr|A0A0D9YPX0|A0A0D9YPX0_9ORYZ:tr|I1NZ10|I1NZ10_ORYGL:tr|T1T4F0|T1T4F0_ORYSI
SWKGPAKNWE	43.60	1201.5880	10	601.8016	10.83	Starch synthase, chloroplastic/amyloplastic	tr|V5NEJ7|V5NEJ7_ORYSA:tr|A0EQD4|A0EQD4_ORYSI:tr|A0EQE0|A0EQE0_ORYSA:tr|A0EQK6|A0EQK6_ORYRU:tr|B8XEK0|B8XEK0_ORYSI:tr|B8XEK3|B8XEK3_ORYSA:tr|D0TZY6|D0TZY6_ORYSI:tr|A0A3Q9T378|A0A3Q9T378_ORYSA:tr|D3U2H9|D3U2H9_ORYSA:tr|A0A3Q9T3Z7|A0A3Q9T3Z7_ORYSA:tr|B8XEK2|B8XEK2_ORYSA:tr|B8XEJ8|B8XEJ8_ORYSA:tr|B1B5Z0|B1B5Z0_ORYSI:tr|B1B5Z1|B1B5Z1_ORYSI:tr|A0A0D9WLF6|A0A0D9WLF6_9ORYZ:tr|A0A0E0A4K1|A0A0E0A4K1_9ORYZ
RSPAPPKGF	39.86	955.5239	9	478.7687	7.61	Uncharacterized protein	tr|A0A0E0GD25|A0A0E0GD25_ORYNI:tr|A0A0E0Q1K4|A0A0E0Q1K4_ORYRU:tr|A0A0E0Q1K2|A0A0E0Q1K2_ORYRU:tr|A0A0E0AD77|A0A0E0AD77_9ORYZ:tr|A0A0E0Q1K3|A0A0E0Q1K3_ORYRU:tr|A0A0E0AD76|A0A0E0AD76_9ORYZ:tr|I1Q4J4|I1Q4J4_ORYGL:tr|B8B1F4|B8B1F4_ORYSI:tr|A0A0E0AD79|A0A0E0AD79_9ORYZ:tr|A0A0E0AD78|A0A0E0AD78_9ORYZ:tr|A0A0E0AD80|A0A0E0AD80_9ORYZ
KGGIPIGIGK	38.94	938.5912	10	470.3035	12.21	Glucose-1-phosphate adenylyltransferase	tr|D4AIA3|D4AIA3_ORYSI:tr|B7EVB8|B7EVB8_ORYSJ:tr|A0A0D3GZB2|A0A0D3GZB2_9ORYZ:tr|A0A0E0H7V9|A0A0E0H7V9_ORYNI:tr|A0A0E0LU24|A0A0E0LU24_ORYPU:tr|A0A0E0QHR8|A0A0E0QHR8_ORYRU:tr|B8XED8|B8XED8_ORYSA:tr|B8XED9|B8XED9_ORYSI:tr|B8XED7|B8XED7_ORYSI:tr|B8XEE8|B8XEE8_ORYSA:tr|B8XEF0|B8XEF0_ORYSI:tr|B8XEF2|B8XEF2_ORYSI:tr|B8XEE9|B8XEE9_ORYSI:tr|B8XEE1|B8XEE1_ORYSI:tr|B8XEE2|B8XEE2_ORYSJ:tr|B8XEE5|B8XEE5_ORYSJ:tr|B8XEE6|B8XEE6_ORYSA:tr|A2YU91|A2YU91_ORYSI:tr|A0A0D3GZB1|A0A0D3GZB1_9ORYZ:tr|A0A0D9X6Z4|A0A0D9X6Z4_9ORYZ:tr|A0A0D9X6Z3|A0A0D9X6Z3_9ORYZ:tr|A0A0E0H7V8|A0A0E0H7V8_ORYNI:tr|A0A0E0QHR7|A0A0E0QHR7_ORYRU:tr|A0A0E0LU23|A0A0E0LU23_ORYPU
APIYTQPR	36.86	944.5079	8	473.2617	7.93	Glucose-1-phosphate adenylyltransferase	tr|D4AIA3|D4AIA3_ORYSI:tr|B7EVB8|B7EVB8_ORYSJ:tr|A0A0D3GZB2|A0A0D3GZB2_9ORYZ:tr|A0A0E0H7V9|A0A0E0H7V9_ORYNI:tr|A0A0E0LU24|A0A0E0LU24_ORYPU:tr|A0A0E0QHR8|A0A0E0QHR8_ORYRU:tr|B8XED8|B8XED8_ORYSA:tr|B8XED9|B8XED9_ORYSI:tr|B8XED7|B8XED7_ORYSI:tr|B8XEE8|B8XEE8_ORYSA:tr|B8XEF0|B8XEF0_ORYSI:tr|B8XEF2|B8XEF2_ORYSI:tr|B8XEE9|B8XEE9_ORYSI:tr|B8XEE1|B8XEE1_ORYSI:tr|B8XEE2|B8XEE2_ORYSJ:tr|B8XEE5|B8XEE5_ORYSJ:tr|B8XEE6|B8XEE6_ORYSA:tr|A2YU91|A2YU91_ORYSI:tr|A0A0D3GZB1|A0A0D3GZB1_9ORYZ:tr|A0A0D9X6Z4|A0A0D9X6Z4_9ORYZ:tr|A0A0D9X6Z3|A0A0D9X6Z3_9ORYZ:tr|A0A0E0H7V8|A0A0E0H7V8_ORYNI:tr|A0A0E0QHR7|A0A0E0QHR7_ORYRU:tr|A0A0E0LU23|A0A0E0LU23_ORYPU

<table>
<tr><td>YIGTPGKGIL</td><td>35.65</td><td>1017.5858</td><td>10</td><td>509.8009</td><td>18.45</td><td>Os01g0905800 protein</td><td>tr|B8A7T3|B8A7T3_ORYSI:tr|Q5N726|Q5N726_ORYSJ:tr|A0A0D3GVV0|A0A0D3GVV0_9ORYZ:tr|A0A0E0L2C4|A0A0E0L2C4_ORYPU:tr|B8AY35|B8AY35_ORYSI:Q10A30|ALFC2_ORYSJ:tr|A0A0E0CBT2|A0A0E0CBT2_9ORYZ:tr|A0A345YV68|A0A345YV68_ORYSJ:tr|A0A0D3G7F4|A0A0D3G7F4_9ORYZ:tr|A0A0E0EW41|A0A0E0EW41_9ORYZ:tr|A0A0E0HE79|A0A0E0HE79_ORYNI:tr|A0A0E0FX88|A0A0E0FX88_ORYNI:tr|A0A0D3HBH5|A0A0D3HBH5_9ORYZ:tr|A0A0D3EXK7|A0A0D3EXK7_9ORYZ:tr|A2XUB6|A2XUB6_ORYSI:tr|A0A0E0JSG1|A0A0E0JSG1_ORYPU:tr|A0A0E0M5T2|A0A0E0M5T2_ORYPU:tr|A0A0E0N6G4|A0A0E0N6G4_ORYRU:tr|A0A0E0DR72|A0A0E0DR72_9ORYZ:P17784|ALFC1_ORYSJ:tr|A0A1L2JKJ5|A0A1L2JKJ5_ORYSA:Q5N725|ALFC3_ORYSJ:tr|A0A0D9WIC8|A0A0D9WIC8_9ORYZ:tr|A0A0E0LQS0|A0A0E0LQS0_ORYPU:tr|Q6YPF1|Q6YPF1_ORYSJ:tr|A0A0E0QDQ9|A0A0E0QDQ9_ORYRU:tr|A2YQR2|A2YQR2_ORYSI:tr|A0A0E0I6R0|A0A0E0I6R0_ORYNI:tr|A0A0E0EH98|A0A0E0EH98_9ORYZ:tr|A0A0E0QWN8|A0A0E0QWN8_ORYRU</td></tr>
<tr><td>WDPSKDKYITR(sub A)</td><td>33.30</td><td>1407.7146</td><td>11</td><td>704.8655</td><td>10.44</td><td>Starch synthase, chloroplastic/amyloplastic</td><td>tr|V5NEJ7|V5NEJ7_ORYSA:tr|A0EQD4|A0EQD4_ORYSI:tr|A0EQE0|A0EQE0_ORYSA:tr|A0EQK6|A0EQK6_ORYRU:tr|B8XEK0|B8XEK0_ORYSI:tr|B8XEK3|B8XEK3_ORYSA:tr|D0TZY6|D0TZY6_ORYSI:tr|A0A3Q9T378|A0A3Q9T378_ORYSA:tr|D3U2H9|D3U2H9_ORYSA:tr|A0A3Q9T3Z7|A0A3Q9T3Z7_ORYSA:tr|B8XEK2|B8XEK2_ORYSA:tr|B8XEJ8|B8XEJ8_ORYSA:tr|B1B5Z0|B1B5Z0_ORYSI:tr|B1B5Z1|B1B5Z1_ORYSI:tr|A0A0E0A4K1|A0A0E0A4K1_9ORYZ</td></tr>
<tr><td>WDPSKDKYITR(sub V)</td><td>33.30</td><td>1407.7146</td><td>11</td><td>704.8655</td><td>10.44</td><td>Starch synthase, chloroplastic/amyloplastic</td><td>tr|A0A0D9WLF6|A0A0D9WLF6_9ORYZ</td></tr>
<tr><td>WIDFPRAPQ</td><td>30.81</td><td>1128.5715</td><td>9</td><td>565.2931</td><td>21.74</td><td>Os02g0528200 protein</td><td>tr|A0A0N7KFE7|A0A0N7KFE7_ORYSJ:tr|A0A0D3F5W2|A0A0D3F5W2_9ORYZ:tr|A0A0E0K0K5|A0A0E0K0K5_ORYPU:tr|A0A0E0G7M7|A0A0E0G7M7_ORYNI:tr|A0A0E0NFS6|A0A0E0NFS6_ORYRU:tr|A0A0D9YT34|A0A0D9YT34_9ORYZ:tr|A0A0E0K0K4|A0A0E0K0K4_ORYPU:tr|A0A0E0CLF1|A0A0E0CLF1_9ORYZ:tr|Q6H6P8|Q6H6P8_ORYSJ:tr|Q40663|Q40663_ORYSA:tr|A2X5K0|A2X5K0_ORYSI:tr|D0TZK1|D0TZK1_ORYSI:tr|I1P0X2|I1P0X2_ORYGL:tr|B3VDJ4|B3VDJ4_ORYSJ:tr|I6VRB8|I6VRB8_ORYSJ:tr|A0A0D9VGL1|A0A0D9VGL1_9ORYZ</td></tr>
<tr><td>TPRFPQKS</td><td>30.58</td><td>959.5188</td><td>8</td><td>480.7661</td><td>5.76</td><td>Glutelin</td><td>tr|T1T4G5|T1T4G5_ORYSI:tr|M1G949|M1G949_ORYSI:Q6K508|GLUD1_ORYSJ:tr|I1NZ02|I1NZ02_ORYGL:tr|A2X2Z1|A2X2Z1_ORYSI:tr|A0A0D3F331|A0A0D3F331_9ORYZ:tr|A0A0E0FMV5|A0A0E0FMV5_ORYNI:tr|A0A0E0NCE4|A0A0E0NCE4_ORYRU:tr|A0A0E0JXZ6|A0A0E0JXZ6_ORYPU:tr|A0A0D9YPW1|A0A0D9YPW1_9ORYZ</td></tr>
</table>

<table>
<tr><td>IDFPRAPQ</td><td>29.73</td><td>942.4922</td><td>8</td><td>472.2540</td><td>15.91</td><td>Os02g0528200 protein</td><td>tr|A0A0N7KFE7|A0A0N7KFE7_ORYSJ:tr|A0A0D3F5W2|A0A0D3F5W2_9ORYZ:tr|A0A0E0K0K5|A0A0E0K0K5_ORYPU:tr|A0A0E0G7M7|A0A0E0G7M7_ORYNI:tr|A0A0E0NFS6|A0A0E0NFS6_ORYRU:tr|A0A0D9YT34|A0A0D9YT34_9ORYZ:tr|A0A0E0K0K4|A0A0E0K0K4_ORYPU:tr|A0A0E0CLF1|A0A0E0CLF1_9ORYZ:tr|Q6H6P8|Q6H6P8_ORYSJ:tr|Q40663|Q40663_ORYSA:tr|A2X5K0|A2X5K0_ORYSI:tr|D0TZK1|D0TZK1_ORYSI:tr|I1P0X2|I1P0X2_ORYGL:tr|B3VDJ4|B3VDJ4_ORYSJ:tr|I6VRB8|I6VRB8_ORYSJ:tr|A0A0D9VGL1|A0A0D9VGL1_9ORYZ</td></tr>
<tr><td>NSQKFPILY(sub N)</td><td>29.67</td><td>1108.5917</td><td>9</td><td>555.3035</td><td>21.79</td><td>Glutelin</td><td>tr|A0A0D3F334|A0A0D3F334_9ORYZ:tr|B9F4T1|B9F4T1_ORYSJ:tr|T1T6C4|T1T6C4_ORYSI:tr|I1NZ08|I1NZ08_ORYGL:tr|A0A0D9YPX0|A0A0D9YPX0_9ORYZ:tr|I1NZ10|I1NZ10_ORYGL:tr|T1T4F0|T1T4F0_ORYSI</td></tr>
<tr><td>RPPKPDAPRIY</td><td>29.26</td><td>1308.7302</td><td>11</td><td>437.2517</td><td>8.29</td><td>Uncharacterized protein</td><td>tr|A0A0E0HVR8|A0A0E0HVR8_ORYNI</td></tr>
<tr><td>RNNQVWQQ</td><td>29.11</td><td>1071.5210</td><td>8</td><td>536.7670</td><td>8.07</td><td>Prolamin PPROL 14E</td><td>P0C5E5|PRO7_ORYSI:tr|A0A0E0HCS0|A0A0E0HCS0_ORYNI</td></tr>
<tr><td>GGATKASPAR</td><td>28.53</td><td>914.4933</td><td>10</td><td>458.2545</td><td>14.85</td><td>Uncharacterized protein</td><td>tr|A0A0E0P725|A0A0E0P725_ORYRU</td></tr>
<tr><td>FGWDKDLAKK</td><td>28.08</td><td>1206.6396</td><td>10</td><td>403.2210</td><td>11.65</td><td>Os02g0519900 protein</td><td>tr|A0A0P0VJL8|A0A0P0VJL8_ORYSJ:tr|A0A0P0W6A1|A0A0P0W6A1_ORYSJ:tr|A0A0D9VGF7|A0A0D9VGF7_9ORYZ:tr|A0A0D3FS13|A0A0D3FS13_9ORYZ:tr|Q01MK8|Q01MK8_ORYSA:tr|A0A0E0DCG7|A0A0E0DCG7_9ORYZ:tr|A0A0D9YSX8|A0A0D9YSX8_9ORYZ:tr|I1P0T4|I1P0T4_ORYGL:tr|A0A0D9ZGI8|A0A0D9ZGI8_9ORYZ:tr|A0A0E0GX73|A0A0E0GX73_ORYNI:tr|A2X5F3|A2X5F3_ORYSI:tr|A0A0E0NFL3|A0A0E0NFL3_ORYRU:tr|J3LDA2|J3LDA2_ORYBR:tr|A0A0E0KM97|A0A0E0KM97_ORYPU:tr|A0A0D3F5Q7|A0A0D3F5Q7_9ORYZ:tr|A0A0E0P4M4|A0A0E0P4M4_ORYRU:tr|A0A0D9V5H0|A0A0D9V5H0_9ORYZ:tr|J3LVA1|J3LVA1_ORYBR:tr|A0A0E0G7F6|A0A0E0G7F6_ORYNI:tr|Q7XTK1|Q7XTK1_ORYSJ:tr|Q6H4L2|Q6H4L2_ORYSJ:tr|I1PIQ9|I1PIQ9_ORYGL:tr|A0A0E0DA54|A0A0E0DA54_9ORYZ:tr|A0A0E0DA52|A0A0E0DA52_9ORYZ:tr|A0A0E0DA53|A0A0E0DA53_9ORYZ</td></tr>
<tr><td>NLNNNPYFK</td><td>26.58</td><td>1122.5458</td><td>9</td><td>562.2805</td><td>13.84</td><td>Starch synthase, chloroplastic/amyloplastic</td><td>tr|V5NEJ7|V5NEJ7_ORYSA:tr|A0EQD4|A0EQD4_ORYSI:tr|A0EQE0|A0EQE0_ORYSA:tr|A0EQK6|A0EQK6_ORYRU:tr|B8XEK0|B8XEK0_ORYSI:tr|B8XEK3|B8XEK3_ORYSA:tr|D0TZY6|D0TZY6_ORYSI:tr|A0A3Q9T378|A0A3Q9T378_ORYSA:tr|D3U2H9|D3U2H9_ORYSA:tr|A0A3Q9T3Z7|A0A3Q9T3Z7_ORYSA:tr|B8XEK2|B8XEK2_ORYSA:tr|B8XEJ8|B8XEJ8_ORYSA:tr|B1B5Z0|B1B5Z0_ORYSI:tr|B1B5Z1|B1B5Z1_ORYSI:tr|A0A0D9WLF6|A0A0D9WLF6_9ORYZ:tr|A0A0E0A4K1|A0A0E0A4K1_9ORYZ</td></tr>
</table>

IGNPHLR	26.34	805.4559	7	403.7355	5.60	Putative mitochondrial energy transfer protein	tr|J3LAP3|J3LAP3_ORYBR:tr|A0A0D3F250|A0A0D3F250_9ORYZ:tr|A0A0E0JX14|A0A0E0JX14_ORYPU:tr|A2X256|A2X256_ORYSI:tr|A3A4A0|A3A4A0_ORYSJ:tr|A0A0E0NY14|A0A0E0NY14_ORYRU:tr|A0A0E0G3F4|A0A0E0G3F4_ORYNI:tr|A0A0D9YNX3|A0A0D9YNX3_9ORYZ:tr|A2YER7|A2YER7_ORYSI:tr|Q69XJ8|Q69XJ8_ORYSJ:tr|A0A0E0ABK1|A0A0E0ABK1_9ORYZ:tr|A0A0P0WYD6|A0A0P0WYD6_ORYSJ:tr|A0A0D9WRV1|A0A0D9WRV1_9ORYZ:tr|A0A0E0E3L8|A0A0E0E3L8_9ORYZ:tr|A0A0E0LD95|A0A0E0LD95_ORYPU:tr|I1Q3G0|I1Q3G0_ORYGL:tr|A0A0E0PZV6|A0A0E0PZV6_ORYRU:tr|A0A0E0HT75|A0A0E0HT75_ORYNI:tr|J3MFK2|J3MFK2_ORYBR:tr|A0A0D3GIF4|A0A0D3GIF4_9ORYZ:tr|Q6Z782|Q6Z782_ORYSJ:tr|I1NYC8|I1NYC8_ORYGL
GDWFNKL	26.06	878.4286	7	440.2219	22.27	Pullulanase	tr|A0A0U1X1L2|A0A0U1X1L2_ORYPU:tr|A0A0U1WXM7|A0A0U1WXM7_ORYNI:tr|A0A0P0W7K6|A0A0P0W7K6_ORYSJ:tr|I1PIZ4|I1PIZ4_ORYGL:tr|Q0JF44|Q0JF44_ORYSJ:tr|I1Q5M1|I1Q5M1_ORYGL:tr|B9FDM0|B9FDM0_ORYSJ:tr|D0TZH2|D0TZH2_ORYSI:tr|D0TZG6|D0TZG6_ORYSJ:tr|D0TZH0|D0TZH0_ORYSI:tr|D0TZH3|D0TZH3_ORYSI:tr|A0A0E0KMN0|A0A0E0KMN0_ORYPU:tr|Q7X834|Q7X834_ORYSJ:tr|I7HIL3|I7HIL3_ORYSA:tr|A0A0E0DAM7|A0A0E0DAM7_9ORYZ:tr|A0A0E0ICV6|A0A0E0ICV6_ORYNI:tr|D0TZG4|D0TZG4_ORYSJ:tr|D0TZH1|D0TZH1_ORYSI:tr|A0A0D3FSF6|A0A0D3FSF6_9ORYZ:tr|O64454|O64454_ORYSA:tr|B8AV01|B8AV01_ORYSI:tr|A0A0E0P590|A0A0E0P590_ORYRU
GDWFNKI	26.06	878.4286	7	440.2219	22.27	Uncharacterized protein	tr|A0A0E0ICV7|A0A0E0ICV7_ORYNI:tr|A0A0E0DAM9|A0A0E0DAM9_9ORYZ:tr|A0A0D9ZGW7|A0A0D9ZGW7_9ORYZ:tr|A0A0E0ICV8|A0A0E0ICV8_ORYNI:tr|A0A0E0DAM8|A0A0E0DAM8_9ORYZ:tr|A0A0D9ZGW5|A0A0D9ZGW5_9ORYZ
SPFRVPIA	24.66	885.5072	8	443.7605	19.95	Pyruvate, phosphate dikinase	tr|J3M716|J3M716_ORYBR:tr|B9FPJ4|B9FPJ4_ORYSJ:tr|A0A0E0PMB6|A0A0E0PMB6_ORYRU:tr|B8AYC1|B8AYC1_ORYSI:tr|A0A0D9ZZ05|A0A0D9ZZ05_9ORYZ:Q6AVA8-2|PPDK1_ORYSJ:tr|A0A0E0DR93|A0A0E0DR93_9ORYZ:tr|A0A0D3G7H5|A0A0D3G7H5_9ORYZ:tr|A0A0D9WGC7|A0A0D9WGC7_9ORYZ:tr|A0A0D9WGC6|A0A0D9WGC6_9ORYZ:Q6AVA8|PPDK1_ORYSJ:tr|I1PVJ3|I1PVJ3_ORYGL
KDKLWPM	24.29	916.4840	7	459.2496	16.79	Os02g0519900 protein	tr|A0A0P0VJL8|A0A0P0VJL8_ORYSJ:tr|A0A0P0W6A1|A0A0P0W6A1_ORYSJ:tr|A0A0D9VGF7|A0A0D9VGF7_9ORYZ:tr|A0A0D3FS13|A0A0D3FS13_9ORYZ:tr|Q01MK8|Q01MK8_ORYSA:tr|A0A0E0DCG7|A0A0E0DCG7_9ORYZ:tr|A0A0D9YSX8|A0A0D9YSX8_9ORYZ:tr|I1P0T4|I1P0T4_ORYGL:tr|A0A0D9ZGI8|A0A0D9ZGI8_9ORYZ:tr|A0A0E0GX73|A0A0E0GX73_ORYNI:tr|A2X5F3|A2X5F3_ORYSI:tr|A0A0E0NFL3|A0A0E0NFL3_ORYRU:tr|J3LDA2|J3LDA2_ORYBR:tr|A0A0E0KM97|A0A0E0KM97_ORYPU:tr|A0A0D3F5Q7|A0A0D3F5Q7_9ORYZ:tr|A0A0E0P4M4|A0A0E0P4M4_ORYRU:tr|A0A0D9V5H0|A0A0D9V5H0_9ORYZ:tr|J3LVA1|J3LVA1_ORYBR:tr|A0A0E0G7F6|A0A0E0G7F6_ORYNI:tr|Q7XTK1|Q7XTK1_ORYSJ:tr|Q6H4L2|Q6H4L2_ORYSJ:tr|I1PIQ9|I1PIQ9_ORYGL:tr|A0A0E0DA54|A0A0E0DA54_

							9ORYZ:tr|A0A0E0DA52|A0A0E0DA52_9ORYZ:tr|A0A0E0DA53|A0A0E0DA53_9ORYZ
VGKFLPI	23.05	772.4847	7	387.2499	22.01	Pyruvate, phosphate dikinase	tr|J3M716|J3M716_ORYBR:tr|B9FPJ4|B9FPJ4_ORYSJ:tr|A0A0E0PMB6|A0A0E0PMB6_ORYRU:tr|B8AYC1|B8AYC1_ORYSI:tr|A0A0D9ZZ05|A0A0D9ZZ05_9ORYZ:Q6AVA8-2|PPDK1_ORYSJ:tr|A0A0E0DR93|A0A0E0DR93_9ORYZ:tr|A0A0D3G7H5|A0A0D3G7H5_9ORYZ:tr|A0A0D9WGC7|A0A0D9WGC7_9ORYZ:tr|A0A0D9WGC6|A0A0D9WGC6_9ORYZ:Q6AVA8|PPDK1_ORYSJ:tr|I1PVJ3|I1PVJ3_ORYGL
GKGYVGL	21.57	692.3857	7	347.2007	12.06	Glutelin	tr|T1T4G5|T1T4G5_ORYSI:tr|M1G949|M1G949_ORYSI:Q6K508|GLUD1_ORYSJ:tr|I1NZ02|I1NZ02_ORYGL:tr|A2X2Z1|A2X2Z1_ORYSI:tr|A0A0D3F331|A0A0D3F331_9ORYZ:tr|A0A0E0FMV5|A0A0E0FMV5_ORYNI:tr|A0A0E0NCE4|A0A0E0NCE4_ORYRU:tr|A0A0E0JXZ6|A0A0E0JXZ6_ORYPU:tr|A0A0D9YPW1|A0A0D9YPW1_9ORYZ
TNPWHSPR	21.51	993.4780	8	497.7458	6.28	Glutelin	tr|A0A0D3F334|A0A0D3F334_9ORYZ:tr|B9F4T1|B9F4T1_ORYSJ:tr|T1T6C4|T1T6C4_ORYSI:tr|I1NZ08|I1NZ08_ORYGL:tr|A0A0D9YPX0|A0A0D9YPX0_9ORYZ:tr|I1NZ10|I1NZ10_ORYGL:tr|T1T4F0|T1T4F0_ORYSI
TRPGGGRVL	20.82	911.5301	9	304.8509	5.92	Uncharacterized protein	tr|A0A0E0GD25|A0A0E0GD25_ORYNI:tr|A0A0E0Q1K4|A0A0E0Q1K4_ORYRU:tr|A0A0E0Q1K2|A0A0E0Q1K2_ORYRU:tr|A0A0E0AD77|A0A0E0AD77_9ORYZ:tr|A0A0E0Q1K3|A0A0E0Q1K3_ORYRU:tr|A0A0E0AD76|A0A0E0AD76_9ORYZ:tr|I1Q4J4|I1Q4J4_ORYGL:tr|B8B1F4|B8B1F4_ORYSI:tr|A0A0E0AD79|A0A0E0AD79_9ORYZ:tr|A0A0E0AD78|A0A0E0AD78_9ORYZ:tr|A0A0E0AD80|A0A0E0AD80_9ORYZ

Sub-fraction n. 18. Total: 26 identified peptides, all with rice protein accession. RT, retention time.

Peptide	-10lgP	Mass (Da)	Length (amino acid n.)	m/z	RT	Protein	Accession
SWKGPAKNWE	52.73	1201.5880	10	601.8016	10.72	Starch synthase, chloroplastic/amyloplastic	tr|V5NEJ7|V5NEJ7_ORYSA:tr|A0EQH2|A0EQH2_ORYSJ:tr|A0EQK7|A0EQK7_ORYRU:tr|A0EQD4|A0EQD4_ORYSI:tr|A0EQK4|A0EQK4_ORYRU:tr|A0EQE0|A0EQE0_ORYSA:tr|A0EQK5|A0EQK5_ORYRU:tr|A0EQK6|A0EQK6_ORYRU:P0C585|SSG1_ORYSA:tr|B8XEK3|B8XEK3_ORYSA:tr|D0TZY6|D0TZY6_ORYSI:tr|A0A3Q9T378|A0A3Q9T378_ORYSA:tr|D3U2H9|D3U2H9_ORYSA:tr|B8XEJ7|B8XEJ7_ORYSA:tr|A0A3Q9T3Z7|A0A3Q9T3Z7_ORYSA:tr|B8XEK2|B8XEK2_ORYSA:tr|A0A076FRI5|A0A076FRI5_ORYSJ:Q42968|SSG1_ORYGL:A2Y8X2|SSG1_ORYSI:tr|C8CBL1|C8CBL1_ORYSJ:tr|B8XEJ8|B8XEJ8_ORYSA:tr|B1B5Z0|B1B5Z0_ORYSI:tr|B1B5Z1|B1B5Z1_ORYSI:tr|A0A0D9WLF6|A0A0D9WLF6_9ORYZ:tr|A0A0E0A4K1|A0A0E0A4K1_9ORYZ

<table>
<tr><td>VFNGVLRPG</td><td>49.93</td><td>957.5396</td><td>9</td><td>479.7774</td><td>15.90</td><td>GluB-5 short variant</td><td>tr|A0A0E0JY91|A0A0E0JY91_ORYPU:tr|A0A0D3F3E5|A0A0D3F3E5_9ORYZ:tr|A2X3A0|A2X3A0_ORYSI:tr|C0L8H2|C0L8H2_ORYSJ:tr|A3A5D6|A3A5D6_ORYSJ:tr|A0A0E0G4Q4|A0A0E0G4Q4_ORYNI:tr|A0A0E0NCT0|A0A0E0NCT0_ORYRU:tr|M1G571|M1G571_ORYSJ:tr|M1G2E3|M1G2E3_ORYSI:tr|A0A0D9VG85|A0A0D9VG85_9ORYZ:tr|Q0E2G5|Q0E2G5_ORYSJ:tr|J3LBL3|J3LBL3_ORYBR:tr|C0L8H1|C0L8H1_ORYSJ:tr|A0A0D9YLS5|A0A0D9YLS5_9ORYZ:tr|Q84X94|Q84X94_ORYSJ:tr|A0A0E0NCA6|A0A0E0NCA6_ORYRU:tr|Q84X93|Q84X93_ORYSJ:tr|A0A0E0G459|A0A0E0G459_ORYNI:tr|Q6ESW6|Q6ESW6_ORYSJ:tr|A2X2V1|A2X2V1_ORYSI:tr|A2X399|A2X399_ORYSI:P14614|GLUB4_ORYSJ:tr|Q0E261|Q0E261_ORYSJ:tr|A0A0D9YQ79|A0A0D9YQ79_9ORYZ:tr|D6BV14|D6BV14_ORYSJ:tr|A0A0D3F3E6|A0A0D3F3E6_9ORYZ:tr|A0A0E0JY90|A0A0E0JY90_ORYPU:Q6ERU3|GLUB5_ORYSJ:tr|I1NZ94|I1NZ94_ORYGL:tr|A0A0D3F3E3|A0A0D3F3E3_9ORYZ</td></tr>
<tr><td>TPIQYKSY</td><td>47.29</td><td>998.5073</td><td>8</td><td>500.2612</td><td>10.31</td><td>Glutelin</td><td>tr|T1T4G3|T1T4G3_ORYSI:tr|A1YQG5|A1YQG5_ORYSJ:tr|I1NRU9|I1NRU9_ORYGL:tr|A2WVB9|A2WVB9_ORYSI:tr|A0A0E0M7E8|A0A0E0M7E8_ORYPU:tr|A0A0E0FTI2|A0A0E0FTI2_ORYNI:tr|A0A0D9YFB1|A0A0D9YFB1_9ORYZ:tr|A0A0D3EUB5|A0A0D3EUB5_9ORYZ:tr|Q0JJ36|Q0JJ36_ORYSJ:tr|Q40689|Q40689_ORYSA</td></tr>
<tr><td>TPLQYKSY</td><td>47.29</td><td>998.5073</td><td>8</td><td>500.2612</td><td>10.31</td><td>Glutelin</td><td>tr|T1T4Y4|T1T4Y4_ORYSI:tr|A1YQG3|A1YQG3_ORYSJ:tr|A2Z708|A2Z708_ORYSI:tr|I1QU95|I1QU95_ORYGL:tr|A0A0E0BA63|A0A0E0BA63_9ORYZ:tr|A0A0E0IRV1|A0A0E0IRV1_ORYNI:tr|A0A0E0QYR6|A0A0E0QYR6_ORYRU:tr|A0A0D3HDD5|A0A0D3HDD5_9ORYZ:tr|A0A0E0IRV2|A0A0E0IRV2_ORYNI</td></tr>
<tr><td>KKPVPDFSFY</td><td>44.81</td><td>1226.6335</td><td>10</td><td>614.3240</td><td>19.19</td><td>ADP-glucose pyrophosphorylase small subunit</td><td>tr|A7IZE4|A7IZE4_ORYSI:tr|D4AIA3|D4AIA3_ORYSI:tr|B7EVB8|B7EVB8_ORYSJ:tr|A0A0D3GZB2|A0A0D3GZB2_9ORYZ:tr|A0A0E0H7V9|A0A0E0H7V9_ORYNI:tr|A0A0E0QHR8|A0A0E0QHR8_ORYRU:tr|B8XED8|B8XED8_ORYSA:tr|B8XED9|B8XED9_ORYSI:tr|B8XED7|B8XED7_ORYSI:tr|B8XEE8|B8XEE8_ORYSA:tr|B8XEF0|B8XEF0_ORYSI:tr|B8XEF2|B8XEF2_ORYSI:tr|B8XEE9|B8XEE9_ORYSI:tr|B8XEE1|B8XEE1_ORYSI:tr|B8XEE2|B8XEE2_ORYSJ:tr|Q9ARH9|Q9ARH9_ORYSA:tr|D3U2H7|D3U2H7_ORYSA:tr|D0TZC9|D0TZC9_ORYSI:Q69T99|GLGS1_ORYSJ:tr|B8XEE5|B8XEE5_ORYSJ:tr|B8XEE6|B8XEE6_ORYSA:tr|D0TZC6|D0TZC6_ORYSJ:tr|A0A0E0EQI0|A0A0E0EQI0_9ORYZ:tr|B8BE16|B8BE16_ORYSI:tr|A2YU91|A2YU91_ORYSI:tr|A0A0D3GZB1|A0A0D3GZB1_9ORYZ:tr|A0A0E0B114|A0A0E0B114_9ORYZ:tr|A0A0D3H4W0|A0A0D3H4W0_9ORYZ:tr|A0A0E0LZH2|A0A0E0LZH2_ORYPU:tr|A0A0E0QHR7|A0A0E0QHR7_ORYRU</td></tr>
<tr><td>RPPKPDAPRIY</td><td>41.00</td><td>1308.7302</td><td>11</td><td>437.2507</td><td>8.11</td><td>Starch-branching enzyme</td><td>tr|E2GHS5|E2GHS5_ORYSI:tr|E2GHS2|E2GHS2_ORYSI:tr|E2GHS6|E2GHS6_ORYSI:tr|A0SHA2|A0SHA2_ORYNI:tr|A0PIS0|A0PIS0_ORYLO:tr|A0PIR9|A0PIR9_9ORYZ:tr|A0PIS1|A0PIS1_9ORYZ:tr|A0PIR8|A0PIR8_9ORYZ:tr|A0PIT1|A0PIT1_ORYSI:tr|A0A0E0AEM3|A0A0E0AEM3_9ORYZ:tr|A0A0E0E6K5|A0A0E0E6K5_9ORYZ:Q01401-2|GLGB_ORYSJ:tr|B7EAH2|B7EAH2_ORYSJ:tr|A0A0E0LFU9|A0A0E0LFU</td></tr>
</table>

							9_ORYPU:tr|A0A2S0T044|A0A2S0T044_ORYSA:tr|A0A2S0T039|A0A2S0T039_ORYSA:tr|A0A0E0LFV0|A0A0E0LFV0_ORYPU:tr|A0A0E0LFU8|A0A0E0LFU8_ORYPU:tr|A0A0E0AEM0|A0A0E0AEM0_9ORYZ:tr|A0A0E0AEL9|A0A0E0AEL9_9ORYZ:tr|A0A0E0LFU7|A0A0E0LFU7_ORYPU:tr|A0A2S0T029|A0A2S0T029_ORYSA:tr|Q0D9D0|Q0D9D0_ORYSJ:Q01401|GLGB_ORYSJ:tr|A0A2S0T020|A0A2S0T020_ORYSA:tr|D0TZI4|D0TZI4_ORYSI:tr|A0A0E0Q2U7|A0A0E0Q2U7_ORYRU:tr|B8B2L2|B8B2L2_ORYSI:tr|A0A0E0Q2U8|A0A0E0Q2U8_ORYRU:tr|A0A0E0Q2U9|A0A0E0Q2U9_ORYRU:tr|A0A0E0E6K3|A0A0E0E6K3_9ORYZ
IGRPAPMPY	39.27	1000.5164	9	501.2657	14.14	Uncharacterized protein	tr|A0A0D3GJV3|A0A0D3GJV3_9ORYZ:tr|A0A0E0GD25|A0A0E0GD25_ORYNI:tr|A0A0E0E5A4|A0A0E0E5A4_9ORYZ:tr|A0A0E0Q1K4|A0A0E0Q1K4_ORYRU:tr|A0A0E0Q1K2|A0A0E0Q1K2_ORYRU:tr|A0A0E0AD77|A0A0E0AD77_9ORYZ:tr|A0A0E0Q1K3|A0A0E0Q1K3_ORYRU:tr|A0A0E0AD76|A0A0E0AD76_9ORYZ:tr|I1Q4J9|I1Q4J9_ORYGL:tr|A0A0D3GJV2|A0A0D3GJV2_9ORYZ:tr|I1Q4J4|I1Q4J4_ORYGL:tr|A2YG59|A2YG59_ORYSI:tr|A0A0E0AD89|A0A0E0AD89_9ORYZ:tr|B8B1F4|B8B1F4_ORYSI:tr|A0A0E0AD79|A0A0E0AD79_9ORYZ:tr|A0A0E0AD78|A0A0E0AD78_9ORYZ:tr|A0A0E0AD80|A0A0E0AD80_9ORYZ:tr|A0A0E0GD31|A0A0E0GD31_ORYNI:tr|A0A0E0E598|A0A0E0E598_9ORYZ
VANPKKPF	38.94	899.5228	8	450.7680	6.53	Phosphoglycerate kinase	tr|A6N1P1|A6N1P1_ORYSI:tr|A0A0E0GWR2|A0A0E0GWR2_ORYNI:tr|A2YG06|A2YG06_ORYSI:tr|A0A0D3GJQ6|A0A0D3GJQ6_9ORYZ:tr|A0A0E0E560|A0A0E0E560_9ORYZ:tr|A0A0E0AD06|A0A0E0AD06_9ORYZ:tr|Q655T1|Q655T1_ORYSJ:tr|A0A0D9WT19|A0A0D9WT19_9ORYZ:tr|A0A0E0Q1D7|A0A0E0Q1D7_ORYRU:tr|J3MGP8|J3MGP8_ORYBR:tr|A0A0E0LEG8|A0A0E0LEG8_ORYPU:tr|I1Q4G7|I1Q4G7_ORYGL:tr|Q09HR1|Q09HR1_ORYSI:tr|A0A0E0HQB3|A0A0E0HQB3_ORYNI:tr|B8AIH2|B8AIH2_ORYSI:tr|A0A0E0CGA6|A0A0E0CGA6_9ORYZ:tr|A0A0E0NAE9|A0A0E0NAE9_ORYRU:tr|Q09HR2|Q09HR2_ORYSI:tr|J3L9Z3|J3L9Z3_ORYBR:tr|A0A0D9YMV6|A0A0D9YMV6_9ORYZ:tr|I1NXN0|I1NXN0_ORYGL:tr|Q6H6C7|Q6H6C7_ORYSJ:tr|A0A0E0CGA5|A0A0E0CGA5_9ORYZ:tr|A0A0D3F185|A0A0D3F185_9ORYZ:tr|A0A0D9VCN8|A0A0D9VCN8_9ORYZ:tr|A0A0E0JW27|A0A0E0JW27_ORYPU
HGAFTPR	37.61	784.3980	7	393.2062	5.45	Glutelin	tr|B9F4T3|B9F4T3_ORYSJ:tr|A0A0E0NCF3|A0A0E0NCF3_ORYRU:P14323|GLUB1_ORYSJ:tr|Q0E2D2|Q0E2D2_ORYSJ
NLNNNPYFK	35.91	1122.5458	9	562.2805	13.74	Starch synthase, chloroplastic/amyloplastic	tr|V5NEJ7|V5NEJ7_ORYSA:tr|A0EQH2|A0EQH2_ORYSJ:tr|A0EQK7|A0EQK7_ORYRU:tr|A0EQD4|A0EQD4_ORYSI:tr|A0EQK4|A0EQK4_ORYRU:tr|A0EQE0|A0EQE0_ORYSA:tr|A0EQK5|A0EQK5_ORYRU:tr|A0EQK6|A0EQK6_ORYRU:P0C585|SSG1_ORYSA:tr|B8XEK3|B8XEK3_ORYSA:tr|D0TZY6|D0TZY6_ORYSI:tr|A0A3Q9T378|A0A3Q9T378_ORYSA:tr|D3U2H9|D3U2H9_ORYSA:tr|B8XEJ7|B8XEJ7_ORYSA:tr|A0A3Q9T3Z7|A0A3Q9T3Z7_ORYSA:tr|B8XEK2|B8XEK2_ORYSA:tr|A0A076FRI5|A0A076FRI5_ORYSJ:Q42968|SSG1_ORYGL:A2Y8X2|SSG1_ORYSI:tr|C8CBL1|C8CBL1_ORYSJ:tr|B8XEJ8|B8XEJ8_ORYSA:tr|B1B5Z0|B1B5Z0_ORYSI:tr|B1B5Z1|B1

<table>
<tr><td></td><td></td><td></td><td></td><td></td><td></td><td></td><td>B5Z1_ORYSI:tr|A0A0D9WLF6|A0A0D9WLF6_9ORYZ:tr|A0A0E0A4K1|A0A0E0A4K1_9ORYZ</td></tr>
<tr><td>GNKRNPQAY</td><td>35.68</td><td>1046.5258</td><td>9</td><td>524.2706</td><td>5.34</td><td>Glutelin</td><td>tr|T1T4G3|T1T4G3_ORYSI:tr|T1T4Y4|T1T4Y4_ORYSI:tr|A1YQG5|A1YQG5_ORYSJ:tr|I1NRU9|I1NRU9_ORYGL:tr|A1YQG3|A1YQG3_ORYSJ:tr|A2WVB9|A2WVB9_ORYSI:tr|A2Z708|A2Z708_ORYSI:tr|I1QU95|I1QU95_ORYGL:tr|A0A0E0M7E8|A0A0E0M7E8_ORYPU:tr|A0A0E0FTI2|A0A0E0FTI2_ORYNI:tr|A0A0D9YFB1|A0A0D9YFB1_9ORYZ:tr|A0A0D3EUB5|A0A0D3EUB5_9ORYZ:tr|Q0JJ36|Q0JJ36_ORYSJ:tr|Q40689|Q40689_ORYSA:tr|A0A0E0BA63|A0A0E0BA63_9ORYZ:tr|A0A0E0IRV1|A0A0E0IRV1_ORYNI:tr|A0A0E0QYR6|A0A0E0QYR6_ORYRU:tr|A0A0D3HDD5|A0A0D3HDD5_9ORYZ:tr|A0A0E0IRV2|A0A0E0IRV2_ORYNI</td></tr>
<tr><td>DNKSNWKF</td><td>34.83</td><td>1037.4930</td><td>8</td><td>519.7539</td><td>13.02</td><td>Starch debranching enzyme</td><td>tr|B3F2P6|B3F2P6_9ORYZ:tr|B3F2P7|B3F2P7_9ORYZ:tr|B3F2P5|B3F2P5_ORYPU:tr|A0A0P0W7K6|A0A0P0W7K6_ORYSJ:tr|I1PIZ4|I1PIZ4_ORYGL:tr|Q0JF44|Q0JF44_ORYSJ:tr|A0A0E0ICV7|A0A0E0ICV7_ORYNI:tr|I1Q5M1|I1Q5M1_ORYGL:tr|B9FDM0|B9FDM0_ORYSJ:tr|A0A0E0DAM9|A0A0E0DAM9_9ORYZ:tr|A0A0D9ZGW7|A0A0D9ZGW7_9ORYZ:tr|A0A0E0ICV8|A0A0E0ICV8_ORYNI:tr|A0A0D9ZGW6|A0A0D9ZGW6_9ORYZ:tr|D0TZH0|D0TZH0_ORYSI:tr|A0A0E0KMN0|A0A0E0KMN0_ORYPU:tr|A0A0E0DAM8|A0A0E0DAM8_9ORYZ:tr|A0A0D9ZGW5|A0A0D9ZGW5_9ORYZ:tr|Q7X834|Q7X834_ORYSJ:tr|I7HIL3|I7HIL3_ORYSA:tr|A0A0E0DAM7|A0A0E0DAM7_9ORYZ:tr|A0A0E0ICV6|A0A0E0ICV6_ORYNI:tr|D0TZG4|D0TZG4_ORYSJ:tr|D0TZH1|D0TZH1_ORYSI:tr|A0A0D3FSF6|A0A0D3FSF6_9ORYZ:tr|P93416|P93416_ORYSA:tr|O64454|O64454_ORYSA</td></tr>
<tr><td>VVVGTPGRVF</td><td>33.46</td><td>1029.5970</td><td>10</td><td>515.8058</td><td>18.03</td><td>Os06g0701100 protein</td><td>tr|A0A0D9WTS3|A0A0D9WTS3_9ORYZ:tr|A0A0D9VC68|A0A0D9VC68_9ORYZ:tr|A0A0N7KMN9|A0A0N7KMN9_ORYSJ:P41376|IF4A1_ARATH:P41377|IF4A2_ARATH:tr|A0A0E0N9T3|A0A0E0N9T3_ORYRU:P35683|IF4A1_ORYSJ:Q6Z2Z4|IF4A3_ORYSJ:tr|J3MHA8|J3MHA8_ORYBR:tr|I1NX84|I1NX84_ORYGL:tr|J3L9J6|J3L9J6_ORYBR:Q9CAI7|IF4A3_ARATH:tr|A0A0E0Q284|A0A0E0Q284_ORYRU:tr|A0A0E0LFA0|A0A0E0LFA0_ORYPU:tr|I1Q4Z1|I1Q4Z1_ORYGL:tr|A2X0V4|A2X0V4_ORYSI:tr|A0A0D9YM91|A0A0D9YM91_9ORYZ:tr|A0A0E0JVG9|A0A0E0JVG9_ORYPU:tr|A0A0D3GKG6|A0A0D3GKG6_9ORYZ:tr|A0A0E0G162|A0A0E0G162_ORYNI:tr|A0A0E0CFK9|A0A0E0CFK9_9ORYZ:tr|A0A0E0ADY5|A0A0E0ADY5_9ORYZ:tr|A0A0E0HUY2|A0A0E0HUY2_ORYNI:tr|A0A0E0HUY1|A0A0E0HUY1_ORYNI:tr|A2YGP5|A2YGP5_ORYSI:tr|A0A0D3F0N3|A0A0D3F0N3_9ORYZ:tr|A0A0E0HUY0|A0A0E0HUY0_ORYNI:tr|A0A0D3F0N2|A0A0D3F0N2_9ORYZ</td></tr>
<tr><td>SSKPFFGGL</td><td>32.95</td><td>938.4861</td><td>9</td><td>470.2505</td><td>21.96</td><td>Nucleoside diphosphate kinase</td><td>tr|A2ZAA7|A2ZAA7_ORYSI:tr|A0A0E0EGM8|A0A0E0EGM8_9ORYZ:tr|A0A0E0MAU0|A0A0E0MAU0_ORYPU</td></tr>
</table>

IGRPAPM(+15.99)PY	32.16	1016.5113	9	509.2630	9.85	Uncharacterized protein	tr|A0A0D3GJV3|A0A0D3GJV3_9ORYZ:tr|A0A0E0GD25|A0A0E0GD25_ORYNI:tr|A0A0E0E5A4|A0A0E0E5A4_9ORYZ:tr|A0A0E0Q1K4|A0A0E0Q1K4_ORYRU:tr|A0A0E0Q1K2|A0A0E0Q1K2_ORYRU:tr|A0A0E0AD77|A0A0E0AD77_9ORYZ:tr|A0A0E0Q1K3|A0A0E0Q1K3_ORYRU:tr|A0A0E0AD76|A0A0E0AD76_9ORYZ:tr|I1Q4J9|I1Q4J9_ORYGL:tr|A0A0D3GJV2|A0A0D3GJV2_9ORYZ:tr|I1Q4J4|I1Q4J4_ORYGL:tr|A2YG59|A2YG59_ORYSI:tr|A0A0E0AD89|A0A0E0AD89_9ORYZ:tr|B8B1F4|B8B1F4_ORYSI:tr|A0A0E0AD79|A0A0E0AD79_9ORYZ:tr|A0A0E0AD78|A0A0E0AD78_9ORYZ:tr|A0A0E0AD80|A0A0E0AD80_9ORYZ:tr|A0A0E0GD31|A0A0E0GD31_ORYNI:tr|A0A0E0E598|A0A0E0E598_9ORYZ
WIDFPRAPQ	31.86	1128.5715	9	565.2937	21.79	Os02g0528200 protein	tr|A0A0N7KFE7|A0A0N7KFE7_ORYSJ:tr|A0A0D3F5W2|A0A0D3F5W2_9ORYZ:tr|A0A0E0K0K5|A0A0E0K0K5_ORYPU:tr|A0A0E0G7M7|A0A0E0G7M7_ORYNI:tr|A0A0E0NFS6|A0A0E0NFS6_ORYRU:tr|A0A0D9YT34|A0A0D9YT34_9ORYZ:tr|A0A0E0K0K4|A0A0E0K0K4_ORYPU:tr|A0A0E0CLF1|A0A0E0CLF1_9ORYZ:tr|Q6H6P8|Q6H6P8_ORYSJ:tr|Q40663|Q40663_ORYSA:tr|A2X5K0|A2X5K0_ORYSI:tr|D0TZK1|D0TZK1_ORYSI:tr|I1P0X2|I1P0X2_ORYGL:tr|B3VDJ4|B3VDJ4_ORYSJ:tr|I6VRB8|I6VRB8_ORYSJ:tr|A0A0D9VGL1|A0A0D9VGL1_9ORYZ
RSPAPPKGF	31.00	955.5239	9	478.7692	7.42	Uncharacterized protein	tr|A0A0D3GJV3|A0A0D3GJV3_9ORYZ:tr|A0A0E0GD25|A0A0E0GD25_ORYNI:tr|A0A0E0E5A4|A0A0E0E5A4_9ORYZ:tr|A0A0E0Q1K4|A0A0E0Q1K4_ORYRU:tr|A0A0E0Q1K2|A0A0E0Q1K2_ORYRU:tr|A0A0E0AD77|A0A0E0AD77_9ORYZ:tr|A0A0E0Q1K3|A0A0E0Q1K3_ORYRU:tr|A0A0E0AD76|A0A0E0AD76_9ORYZ:tr|I1Q4J9|I1Q4J9_ORYGL:tr|A0A0D3GJV2|A0A0D3GJV2_9ORYZ:tr|I1Q4J4|I1Q4J4_ORYGL:tr|A2YG59|A2YG59_ORYSI:tr|A0A0E0AD89|A0A0E0AD89_9ORYZ:tr|B8B1F4|B8B1F4_ORYSI:tr|A0A0E0AD79|A0A0E0AD79_9ORYZ:tr|A0A0E0AD78|A0A0E0AD78_9ORYZ:tr|A0A0E0AD80|A0A0E0AD80_9ORYZ:tr|A0A0E0GD31|A0A0E0GD31_ORYNI:tr|A0A0E0E598|A0A0E0E598_9ORYZ
IDFPRAPQ	28.36	942.4922	8	472.2540	15.91	Os02g0528200 protein	tr|A0A0N7KFE7|A0A0N7KFE7_ORYSJ:tr|A0A0D3F5W2|A0A0D3F5W2_9ORYZ:tr|A0A0E0K0K5|A0A0E0K0K5_ORYPU:tr|A0A0E0G7M7|A0A0E0G7M7_ORYNI:tr|A0A0E0NFS6|A0A0E0NFS6_ORYRU:tr|A0A0D9YT34|A0A0D9YT34_9ORYZ:tr|A0A0E0K0K4|A0A0E0K0K4_ORYPU:tr|A0A0E0CLF1|A0A0E0CLF1_9ORYZ:tr|Q6H6P8|Q6H6P8_ORYSJ:tr|Q40663|Q40663_ORYSA:tr|A2X5K0|A2X5K0_ORYSI:tr|D0TZK1|D0TZK1_ORYSI:tr|I1P0X2|I1P0X2_ORYGL:tr|B3VDJ4|B3VDJ4_ORYSJ:tr|I6VRB8|I6VRB8_ORYSJ:tr|A0A0D9VGL1|A0A0D9VGL1_9ORYZ
YIGTPGKGIL	28.05	1017.5858	10	509.8004	18.47	Os01g0905800 protein	tr|B8A7T3|B8A7T3_ORYSI:tr|Q5N726|Q5N726_ORYSJ:tr|A0A0D3GVV0|A0A0D3GVV0_9ORYZ:tr|A0A0E0L2C4|A0A0E0L2C4_ORYPU:tr|B8AY35|B8AY35_ORYSI:Q10A30|ALFC2_ORYSJ:tr|A0A0E0CBT2|A0A0E0CBT2_9ORYZ:tr|A0A345YV68|A0A345YV68_ORYSJ:tr|A0A0D3G7F4|A0A0D3G7F4_9ORYZ:tr|A0A0E0EW41|A0A0E0EW41_9ORYZ:tr|A0A0E0HE79|A0A0E0HE79_ORYNI:tr|A0A0D3HBH5|A0A0D3HBH5_9ORYZ:tr|A0A0D3EXK7|A0A0D3EXK7_9ORYZ:tr|A2XUB6|A2XUB6_ORYSI:tr|A0A0E0JSG1|A0A0E0JSG1_ORYPU:tr|A0A0E0M5T2|A0A0E0M5T2_ORYPU:tr|A0A0E0N6

							G4|A0A0E0N6G4_ORYRU:tr|A0A0E0DR72|A0A0E0DR72_9ORYZ:P17784|ALFC1_ORYSJ:tr|A0A1L2JKJ5|A0A1L2JKJ5_ORYSA:Q5N725|ALFC3_ORYSJ:tr|A0A0D9WIC8|A0A0D9WIC8_9ORYZ:tr|A0A0E0LQS0|A0A0E0LQS0_ORYPU:tr|Q6YPF1|Q6YPF1_ORYSJ:tr|A0A0E0QDQ9|A0A0E0QDQ9_ORYRU:tr|A2YQR2|A2YQR2_ORYSI:tr|A0A0E0I6R0|A0A0E0I6R0_ORYNI:tr|A0A0E0EH98|A0A0E0EH98_9ORYZ:tr|A0A0E0QWN8|A0A0E0QWN8_ORYRU
AFEPIRSW(sub V)	26.98	1004.5079	8	503.2613	20.94	Glutelin	tr|T1T4G3|T1T4G3_ORYSI:tr|T1T4Y4|T1T4Y4_ORYSI:tr|A1YQG5|A1YQG5_ORYSJ:tr|I1NRU9|I1NRU9_ORYGL:tr|A1YQG3|A1YQG3_ORYSJ:tr|A2WVB9|A2WVB9_ORYSI:tr|A2Z708|A2Z708_ORYSI:tr|I1QU95|I1QU95_ORYGL:tr|A0A0E0M7E8|A0A0E0M7E8_ORYPU:tr|A0A0E0FTI2|A0A0E0FTI2_ORYNI:tr|A0A0D9YFB1|A0A0D9YFB1_9ORYZ:tr|A0A0D3EUB5|A0A0D3EUB5_9ORYZ:tr|Q0JJ36|Q0JJ36_ORYSJ:tr|Q40689|Q40689_ORYSA:tr|A0A0E0BA63|A0A0E0BA63_9ORYZ:tr|A0A0E0IRV1|A0A0E0IRV1_ORYNI:tr|A0A0E0QYR6|A0A0E0QYR6_ORYRU:tr|A0A0D3HDD5|A0A0D3HDD5_9ORYZ:tr|A0A0E0IRV2|A0A0E0IRV2_ORYNI
SRPDFRF	26.63	923.4613	7	462.7385	13.72	Os10g0189100 protein	tr|A0A0E0EWF5|A0A0E0EWF5_9ORYZ:tr|A0A0E0EWF4|A0A0E0EWF4_9ORYZ:tr|A0A0E0EWF3|A0A0E0EWF3_9ORYZ:tr|A0A0E0IQA4|A0A0E0IQA4_ORYNI:tr|A0A0D3HBW4|A0A0D3HBW4_9ORYZ:tr|B8BG13|B8BG13_ORYSI:tr|A0A0E0B8J4|A0A0E0B8J4_9ORYZ:tr|Q33AE4|Q33AE4_ORYSJ:tr|I1QT43|I1QT43_ORYGL:tr|Q53QR8|Q53QR8_ORYSJ:tr|J3N1F5|J3N1F5_ORYBR:tr|A0A0D9XIC3|A0A0D9XIC3_9ORYZ:tr|A0A0E0QX42|A0A0E0QX42_ORYRU:tr|A0A0E0M659|A0A0E0M659_ORYPU
DWYKGPTLL	25.66	1091.5651	9	546.7900	26.93	Os03g0177900 protein	tr|A0A0P0VTT8|A0A0P0VTT8_ORYSJ:O64937|EF1A_ORYSJ
RLPAVGSF	23.36	845.4759	8	423.7451	15.97	Auxin response factor	tr|A0A0E0DT15|A0A0E0DT15_9ORYZ:tr|A0A0D3GN20|A0A0D3GN20_9ORYZ
YTNIPGVVYH(sub I)	23.35	1161.5818	10	581.7996	17.22	Glutelin	tr|B9F4T3|B9F4T3_ORYSJ:tr|A0A0E0NCF3|A0A0E0NCF3_ORYRU:P14323|GLUB1_ORYSJ:tr|Q0E2D2|Q0E2D2_ORYSJ
KAGKFPTL	22.82	860.5120	8	431.2638	12.23	Ribosomal protein	tr|Q5QL84|Q5QL84_ORYSJ:tr|Q6YZI6|Q6YZI6_ORYSJ:tr|A6N0A3|A6N0A3_ORYSI:tr|J3MV98|J3MV98_ORYBR:tr|J3L624|J3L624_ORYBR:B8B9K6|R10A_ORYSI:tr|A0A0E0AYW9|A0A0E0AYW9_9ORYZ:tr|I1QLR2|I1QLR2_ORYGL:tr|Q6ER67|Q6ER67_ORYSJ:B7F845|R10A_ORYSJ:tr|Q6ZJ04|Q6ZJ04_ORYSJ:tr|J3LCA4|J3LCA4_ORYBR:tr|B7F9R7|B7F9R7_ORYSJ:tr|I1QKE1|I1QKE1_ORYGL:tr|J3MV97|J3MV97_ORYBR
LSPFW(+15.99)NINAH	21.11	1213.5880	10	607.8016	19.85	Glutelin	tr|T1T4G3|T1T4G3_ORYSI:tr|T1T4Y4|T1T4Y4_ORYSI:tr|A1YQG5|A1YQG5_ORYSJ:tr|I1NRU9|I1NRU9_ORYGL:tr|A1YQG3|A1YQG3_ORYSJ:tr|A2WVB9|A2WVB9_ORYSI:tr|A2Z708|A2Z708_ORYSI:tr|I1QU95|I1QU95_ORYGL:tr|A0A0E0M7E8|A0A0E0M7E8_ORYPU:tr|A0A0E0FTI2|A0A0E0F

							TI2_ORYNI:tr|A0A0D9YFB1|A0A0D9YFB1_9ORYZ:tr|A0A0D3EUB5|A0A0D3EUB5_9ORYZ:tr|Q0JJ36|Q0JJ36_ORYSJ:tr|Q40689|Q40689_ORYSA:tr|A0A0E0BA63|A0A0E0BA63_9ORYZ:tr|A0A0E0IRV1|A0A0E0IRV1_ORYNI:tr|A0A0E0QYR6|A0A0E0QYR6_ORYRU:tr|A0A0D3HDD5|A0A0D3HDD5_9ORYZ:tr|A0A0E0IRV2|A0A0E0IRV2_ORYNI

Sub-fraction n. 19. Total: 22 identified peptides, 21 peptides with rice protein accession. RT, retention time.

Peptide	-10lgP	Mass (Da)	Length (amino acid n.)	m/z	RT (min)	Protein	Accession
TPIQYKSY	51.44	998.5073	8	500.2612	10.80	Glutelin	tr|A0A0E0M7E9|A0A0E0M7E9_ORYPU:tr|T1T4G3|T1T4G3_ORYSI:tr|A0A0E0JP14|A0A0E0JP14_ORYPU:tr|A1YQG5|A1YQG5_ORYSJ:tr|I1NRU9|I1NRU9_ORYGL:tr|A0A0E0N2T5|A0A0E0N2T5_ORYRU:tr|A2WVB9|A2WVB9_ORYSI:P07728|GLUA1_ORYSJ:tr|A0A0E0M7E8|A0A0E0M7E8_ORYPU:tr|A0A0E0FTI2|A0A0E0FTI2_ORYNI:tr|A0A0E0C821|A0A0E0C821_9ORYZ:tr|A0A0D3EUB5|A0A0D3EUB5_9ORYZ:tr|Q0JJ36|Q0JJ36_ORYSJ:tr|Q40689|Q40689_ORYSA
TPLQYKSY	51.44	998.5073	8	500.2612	10.80	Glutelin	tr|T1T4Y4|T1T4Y4_ORYSI:tr|A1YQG3|A1YQG3_ORYSJ:P07730|GLUA2_ORYSJ:tr|A2Z708|A2Z708_ORYSI:tr|I1QU95|I1QU95_ORYGL:tr|A0A0E0BA65|A0A0E0BA65_9ORYZ:tr|A0A0E0IRV3|A0A0E0IRV3_ORYNI:tr|A0A0D3HDD6|A0A0D3HDD6_9ORYZ:tr|A0A0E0QYR7|A0A0E0QYR7_ORYRU:tr|A0A0E0BA63|A0A0E0BA63_9ORYZ:tr|A0A0E0BA64|A0A0E0BA64_9ORYZ:tr|A0A0E0IRV1|A0A0E0IRV1_ORYNI:tr|A0A0E0QYR5|A0A0E0QYR5_ORYRU:tr|A0A0E0QYR6|A0A0E0QYR6_ORYRU:tr|A0A0D3HDD5|A0A0D3HDD5_9ORYZ:tr|A0A0E0IRV2|A0A0E0IRV2_ORYNI
KKPVPDFSFY	50.89	1226.6335	10	614.3219	19.36	ADP-glucose pyrophosphorylase small subunit	tr|A7IZE4|A7IZE4_ORYSI:tr|D4AIA3|D4AIA3_ORYSI:tr|B7EVB8|B7EVB8_ORYSJ:tr|A0A0D3GZB2|A0A0D3GZB2_9ORYZ:tr|A0A0E0H7V9|A0A0E0H7V9_ORYNI:tr|A0A0E0QHR8|A0A0E0QHR8_ORYRU:tr|B8XED8|B8XED8_ORYSA:tr|B8XED9|B8XED9_ORYSI:tr|B8XED7|B8XED7_ORYSI:tr|B8XEE8|B8XEE8_ORYSA:tr|B8XEF0|B8XEF0_ORYSI:tr|B8XEF2|B8XEF2_ORYSI:tr|B8XEE9|B8XEE9_ORYSI:tr|B8XEE1|B8XEE1_ORYSI:tr|B8XEE2|B8XEE2_ORYSJ:tr|Q9ARH9|Q9ARH9_ORYSA:tr|D3U2H7|D3U2H7_ORYSA:tr|D0TZC9|D0TZC9_ORYSI:Q69T99|GLGS1_ORYSJ:tr|B8XEE5|B8XEE5_ORYSJ:tr|B8XEE6|B8XEE6_ORYSA:tr|D0TZC6|D0TZC6_ORYSJ:tr|A0A0E0EQI0|A0A0E0EQI0_9ORYZ:tr|B8BE16|B8BE16_ORYSI:tr|A2YU91|A2YU91_ORYSI:tr|A0A0D3GZB1|A0A0D3GZB1_9ORYZ:tr|A0A0E0B114|A0A0E0B114_9ORYZ:tr|A0A0D3H4W0|A0A0D3H4W0_9ORYZ:tr|A0A0E0LZH2|A0A0E0LZH2_ORYPU:tr|A0A0E0QHR7|A0A0E0QHR7_ORYRU
SWKGPAKNWE	49.54	1201.5880	10	601.8023	11.07	Granule-bound	tr|A5XE09|A5XE09_9ORYZ:tr|A5XE24|A5XE24_ORYPU:tr|A5XDZ6|A5XDZ6_9ORYZ:tr|S4S218|S4S218_9ORYZ:tr|S4S221|S4S221_ORYGR:tr|S4S1Y5|S4S1Y5_9ORYZ:tr|V5NEJ7|V5NEJ7_ORYSA:tr|A0EQH2|A0EQH

<table>
<tr><td></td><td></td><td></td><td></td><td></td><td></td><td>starch synthase</td><td>2_ORYSJ:tr|A0EQD4|A0EQD4_ORYSI:tr|A0EQK4|A0EQK4_ORYRU:tr|A0EQE0|A0EQE0_ORYSA:tr|A0EQK5|A0EQK5_ORYRU:tr|A0EQK6|A0EQK6_ORYRU:P0C585|SSG1_ORYSA:tr|B8XEK3|B8XEK3_ORYSA:tr|D0TZY6|D0TZY6_ORYSI:tr|A0A3Q9T378|A0A3Q9T378_ORYSA:tr|D3U2H9|D3U2H9_ORYSA:tr|A0A3Q9T3Z7|A0A3Q9T3Z7_ORYSA:tr|B8XEK2|B8XEK2_ORYSA:tr|A0A076FRI5|A0A076FRI5_ORYSJ:Q42968|SSG1_ORYGL:A2Y8X2|SSG1_ORYSI:tr|C8CBL1|C8CBL1_ORYSJ:tr|B8XEJ8|B8XEJ8_ORYSA:tr|B1B5Z0|B1B5Z0_ORYSI:tr|B1B5Z1|B1B5Z1_ORYSI:tr|A0A0D9WLF6|A0A0D9WLF6_9ORYZ:tr|A0A0E0A4K1|A0A0E0A4K1_9ORYZ:tr|I1VW86|I1VW86_ORYPU:tr|I1VWC3|I1VWC3_9ORYZ:tr|I1VWB8|I1VWB8_9ORYZ:tr|A5Y217|A5Y217_ORYNI:tr|A5Y253|A5Y253_ORYRU:tr|A5Y213|A5Y213_ORYNI</td></tr>
<tr><td>VFNGVLRPG</td><td>46.07</td><td>957.5396</td><td>9</td><td>479.7774</td><td>15.94</td><td>GluB-5 short variant</td><td>tr|A0A0E0JY91|A0A0E0JY91_ORYPU:tr|A0A0D3F3E5|A0A0D3F3E5_9ORYZ:tr|A2X3A0|A2X3A0_ORYSI:tr|C0L8H2|C0L8H2_ORYSJ:tr|A3A5D6|A3A5D6_ORYSJ:tr|A0A0E0G4Q4|A0A0E0G4Q4_ORYNI:tr|A0A0E0NCT0|A0A0E0NCT0_ORYRU:tr|M1G571|M1G571_ORYSJ:tr|M1G2E3|M1G2E3_ORYSI:tr|A0A0D9VG85|A0A0D9VG85_9ORYZ:tr|Q0E2G5|Q0E2G5_ORYSJ:tr|J3LBL3|J3LBL3_ORYBR:tr|C0L8H1|C0L8H1_ORYSJ:tr|A0A0D9YLS5|A0A0D9YLS5_9ORYZ:tr|Q84X94|Q84X94_ORYSJ:tr|A0A0E0NCA6|A0A0E0NCA6_ORYRU:tr|Q84X93|Q84X93_ORYSJ:tr|A0A0E0G459|A0A0E0G459_ORYNI:tr|Q6ESW6|Q6ESW6_ORYSJ:tr|A2X2V1|A2X2V1_ORYSI:tr|A2X399|A2X399_ORYSI:P14614|GLUB4_ORYSJ:tr|Q0E261|Q0E261_ORYSJ:tr|A0A0D9YQ79|A0A0D9YQ79_9ORYZ:tr|D6BV14|D6BV14_ORYSJ:tr|A0A0D3F3E6|A0A0D3F3E6_9ORYZ:tr|A0A0E0JY90|A0A0E0JY90_ORYPU:Q6ERU3|GLUB5_ORYSJ:tr|I1NZ94|I1NZ94_ORYGL:tr|A0A0D3F3E3|A0A0D3F3E3_9ORYZ</td></tr>
<tr><td>IGRPAPMPY</td><td>43.77</td><td>1000.5164</td><td>9</td><td>501.2657</td><td>14.28</td><td>Os06g0676700 protein</td><td>tr|A0A0E0GD25|A0A0E0GD25_ORYNI:tr|A0A0E0Q1K4|A0A0E0Q1K4_ORYRU:tr|A0A0E0Q1K2|A0A0E0Q1K2_ORYRU:tr|A0A0E0AD77|A0A0E0AD77_9ORYZ:tr|A0A0E0Q1K3|A0A0E0Q1K3_ORYRU:tr|A0A0E0AD76|A0A0E0AD76_9ORYZ:Q653V7|AGLU_ORYSJ:tr|B8B1F4|B8B1F4_ORYSI:tr|A0A0E0LEM2|A0A0E0LEM2_ORYPU:tr|A0A0E0AD80|A0A0E0AD80_9ORYZ:tr|A0A0E0Q1L2|A0A0E0Q1L2_ORYRU:tr|A0A0E0LEN1|A0A0E0LEN1_ORYPU:tr|A0A0D3GJV3|A0A0D3GJV3_9ORYZ:tr|A0A0E0E5A4|A0A0E0E5A4_9ORYZ:tr|A3BEL8|A3BEL8_ORYSJ:tr|A0A0D3GJV2|A0A0D3GJV2_9ORYZ:tr|Q653V4|Q653V4_ORYSJ:tr|A2YG59|A2YG59_ORYSI:tr|A0A0E0AD89|A0A0E0AD89_9ORYZ:tr|B8AD31|B8AD31_ORYSI:tr|Q9LGC6|Q9LGC6_ORYSJ:tr|A0A0D9UWE3|A0A0D9UWE3_9ORYZ:tr|A0A0E0BWI1|A0A0E0BWI1_9ORYZ:tr|Q0JQZ2|Q0JQZ2_ORYSJ:tr|A0A0E0MQY3|A0A0E0MQY3_ORYRU:tr|A0A0E0FFR9|A0A0E0FFR9_ORYNI:tr|A0A0D3EJ82|A0A0D3EJ82_9ORYZ:tr|A0A0E0JDN4|A0A0E0JDN4_ORYPU:tr|A0A0E0GD31|A0A0E0GD31_ORYNI:tr|A0A0E0E598|A0A0E0E598_9ORYZ</td></tr>
</table>

<table>
<tr><td>KGQTPVFPR</td><td>41.83</td><td>1028.5767</td><td>9</td><td>515.2948</td><td>8.76</td><td>Alcohol dehydrogenase 1</td><td>tr|A0A0E0MDS3|A0A0E0MDS3_ORYPU:tr|A0A0E0F3I6|A0A0E0F3I6_9ORYZ:tr|Q75ZX2|Q75ZX2_9ORYZ:tr|Q4R1G8|Q4R1G8_ORYRU:tr|B6F2D3|B6F2D3_9ORYZ:tr|B9V0Q8|B9V0Q8_9ORYZ:tr|B6F2D0|B6F2D0_ORYPU:tr|Q75ZX3|Q75ZX3_9ORYZ:Q2R8Z5|ADH1_ORYSJ:tr|B6F2B9|B6F2B9_ORYSI:tr|Q4R1F4|Q4R1F4_ORYRU:tr|Q75ZX1|Q75ZX1_9ORYZ:tr|D7PPK7|D7PPK7_ORYSJ:tr|Q4R1F0|Q4R1F0_ORYRU:Q0ITW7|ADH2_ORYSJ:tr|Q75ZX6|Q75ZX6_ORYRU:tr|B6F2D1|B6F2D1_ORYMI:tr|B9V0R7|B9V0R7_ORYPU:tr|B6F2D5|B6F2D5_ORYBR:tr|D7PPG9|D7PPG9_ORYRU:tr|B9V0N7|B9V0N7_ORYNI:Q75ZX4|ADH1_ORYSI:Q4R1E8|ADH2_ORYSI:tr|B9V0R8|B9V0R8_ORYPU:tr|B9V0Q7|B9V0Q7_9ORYZ:tr|A0A0E0F3I5|A0A0E0F3I5_9ORYZ:tr|A2ZCK1|A2ZCK1_ORYSI:tr|A0A0E0F3I4|A0A0E0F3I4_9ORYZ:tr|A0A0E0F3I3|A0A0E0F3I3_9ORYZ:tr|A0A0E0F3I0|A0A0E0F3I0_9ORYZ</td></tr>
<tr><td>HGAFTPR</td><td>39.54</td><td>784.3980</td><td>7</td><td>393.2062</td><td>5.63</td><td>Glutelin</td><td>tr|A0A0D9VE94|A0A0D9VE94_9ORYZ:tr|J3LBT5|J3LBT5_ORYBR:tr|B9F4T2|B9F4T2_ORYSJ:tr|A0A0D3F337|A0A0D3F337_9ORYZ:tr|A0A0E0G6R3|A0A0E0G6R3_ORYNI:tr|A2X301|A2X301_ORYSI:tr|J3LB95|J3LB95_ORYBR:tr|A0A0E0NCF1|A0A0E0NCF1_ORYRU:tr|A0A0D3F334|A0A0D3F334_9ORYZ:tr|B9F4T1|B9F4T1_ORYSJ:tr|B9F4T3|B9F4T3_ORYSJ:tr|B8AEZ5|B8AEZ5_ORYSI:tr|T1T6C4|T1T6C4_ORYSI:tr|A1YQH4|A1YQH4_ORYSJ:Q02897|GLUB2_ORYSJ:tr|A1YQH6|A1YQH6_ORYSJ:tr|I1NZ08|I1NZ08_ORYGL:tr|Q0E2D5|Q0E2D5_ORYSJ:tr|A1YQH5|A1YQH5_ORYSJ:tr|A0A0E0CIL1|A0A0E0CIL1_9ORYZ:tr|A0A0E0JY04|A0A0E0JY04_ORYPU:tr|A0A0E0NCF3|A0A0E0NCF3_ORYRU:tr|A0A0E0G6R1|A0A0E0G6R1_ORYNI:P14323|GLUB1_ORYSJ:tr|A0A0D9YPX0|A0A0D9YPX0_9ORYZ:tr|A0A0D3F336|A0A0D3F336_9ORYZ:tr|I1NZ10|I1NZ10_ORYGL:tr|Q0E2D2|Q0E2D2_ORYSJ:tr|T1T4F0|T1T4F0_ORYSI:tr|A0A0E0CIL2|A0A0E0CIL2_9ORYZ</td></tr>
<tr><td>VANPKKPF</td><td>36.68</td><td>899.5228</td><td>8</td><td>450.7681</td><td>7.00</td><td>Phosphoglycerate kinase</td><td>tr|A6N1P1|A6N1P1_ORYSI:tr|A0A0E0GWR2|A0A0E0GWR2_ORYNI:tr|A2YG06|A2YG06_ORYSI:tr|A0A0D3GJQ6|A0A0D3GJQ6_9ORYZ:tr|A0A0E0E560|A0A0E0E560_9ORYZ:tr|A0A0E0AD06|A0A0E0AD06_9ORYZ:tr|Q655T1|Q655T1_ORYSJ:tr|A0A0D9WT19|A0A0D9WT19_9ORYZ:tr|A0A0E0Q1D7|A0A0E0Q1D7_ORYRU:tr|J3MGP8|J3MGP8_ORYBR:tr|A0A0E0LEG8|A0A0E0LEG8_ORYPU:tr|I1Q4G7|I1Q4G7_ORYGL:tr|Q09HR1|Q09HR1_ORYSI:tr|A0A0E0HQB3|A0A0E0HQB3_ORYNI:tr|B8AIH2|B8AIH2_ORYSI:tr|A0A0E0CGA6|A0A0E0CGA6_9ORYZ:tr|A0A0E0NAE9|A0A0E0NAE9_ORYRU:tr|Q09HR2|Q09HR2_ORYSI:tr|J3L9Z3|J3L9Z3_ORYBR:tr|A0A0D9YMV6|A0A0D9YMV6_9ORYZ:tr|I1NXN0|I1NXN0_ORYGL:tr|Q6H6C7|Q6H6C7_ORYSJ:tr|A0A0E0CGA5|A0A0E0CGA5_9ORYZ:tr|A0A0D3F185|A0A0D3F185_9ORYZ:tr|A0A0D9VCN8|A0A0D9VCN8_9ORYZ:tr|A0A0E0JW27|A0A0E0JW27_ORYPU</td></tr>
<tr><td>KKPVPDFS</td><td>36.67</td><td>916.5018</td><td>8</td><td>459.2580</td><td>8.23</td><td>ADP-glucose pyrophosphorylase</td><td>tr|A7IZE4|A7IZE4_ORYSI:tr|D4AIA3|D4AIA3_ORYSI:tr|B7EVB8|B7EVB8_ORYSJ:tr|A0A0D3GZB2|A0A0D3GZB2_9ORYZ:tr|A0A0E0H7V9|A0A0E0H7V9_ORYNI:tr|A0A0E0QHR8|A0A0E0QHR8_ORYRU:tr|B8XED8|B8XED8_ORYSA:tr|B8XED9|B8XED9_ORYSI:tr|B8XED7|B8XED7_ORYSI:tr|B</td></tr>
</table>

<table>
<tr><td></td><td></td><td></td><td></td><td></td><td></td><td>small subunit</td><td>8XEE8|B8XEE8_ORYSA:tr|B8XEF0|B8XEF0_ORYSI:tr|B8XEF2|B8XEF2_ORYSI:tr|B8XEE9|B8XEE9_ORYSI:tr|B8XEE1|B8XEE1_ORYSI:tr|B8XEE2|B8XEE2_ORYSJ:tr|Q9ARH9|Q9ARH9_ORYSA:tr|D3U2H7|D3U2H7_ORYSA:tr|D0TZC9|D0TZC9_ORYSI:Q69T99|GLGS1_ORYSJ:tr|B8XEE5|B8XEE5_ORYSJ:tr|B8XEE6|B8XEE6_ORYSA:tr|D0TZC6|D0TZC6_ORYSJ:tr|A0A0E0EQI0|A0A0E0EQI0_9ORYZ:tr|B8BE16|B8BE16_ORYSI:tr|A2YU91|A2YU91_ORYSI:tr|A0A0D3GZB1|A0A0D3GZB1_9ORYZ:tr|A0A0E0B114|A0A0E0B114_9ORYZ:tr|A0A0D3H4W0|A0A0D3H4W0_9ORYZ:tr|A0A0E0LZH2|A0A0E0LZH2_ORYPU:tr|A0A0E0QHR7|A0A0E0QHR7_ORYRU</td></tr>
<tr><td>RPPKPDAPRIY</td><td>35.56</td><td>1308.7302</td><td>11</td><td>437.2517</td><td>8.77</td><td>Starch-branching enzyme</td><td>tr|E2GHS5|E2GHS5_ORYSI:tr|E2GHS2|E2GHS2_ORYSI:tr|E2GHS6|E2GHS6_ORYSI:tr|A0SHA2|A0SHA2_ORYNI:tr|A0PIS0|A0PIS0_ORYLO:tr|A0PIR9|A0PIR9_9ORYZ:tr|A0PIS1|A0PIS1_9ORYZ:tr|A0PIR8|A0PIR8_9ORYZ:tr|A0PIT1|A0PIT1_ORYSI:tr|A0A0E0E6K5|A0A0E0E6K5_9ORYZ:Q01401-2|GLGB_ORYSJ:tr|B7EAH2|B7EAH2_ORYSJ:tr|A0A0E0LFU9|A0A0E0LFU9_ORYPU:tr|A0A2S0T044|A0A2S0T044_ORYSA:tr|A0A2S0T039|A0A2S0T039_ORYSA:tr|A0A0E0LFV0|A0A0E0LFV0_ORYPU:tr|A0A0E0LFU8|A0A0E0LFU8_ORYPU:tr|A0A0E0AEM0|A0A0E0AEM0_9ORYZ:tr|A0A0E0AEL9|A0A0E0AEL9_9ORYZ:tr|A0A0E0LFU7|A0A0E0LFU7_ORYPU:tr|A0A2S0T029|A0A2S0T029_ORYSA:tr|Q0D9D0|Q0D9D0_ORYSJ:Q01401|GLGB_ORYSJ:tr|A0A2S0T020|A0A2S0T020_ORYSA:tr|D0TZI4|D0TZI4_ORYSI:tr|A0A0E0Q2U7|A0A0E0Q2U7_ORYRU:tr|B8B2L2|B8B2L2_ORYSI:tr|A0A0E0Q2U8|A0A0E0Q2U8_ORYRU:tr|A0A0E0Q2U9|A0A0E0Q2U9_ORYRU:tr|A0A0E0E6K3|A0A0E0E6K3_9ORYZ</td></tr>
<tr><td>SKNPFFN</td><td>34.36</td><td>852.4130</td><td>7</td><td>427.2142</td><td>15.66</td><td>Os03g0271200 protein</td><td>tr|A0A0E0MBQ7|A0A0E0MBQ7_ORYPU:tr|A0A0P0VVX9|A0A0P0VVX9_ORYSJ:tr|A0A0E0F1W6|A0A0E0F1W6_9ORYZ:tr|A0A0E0F1W3|A0A0E0F1W3_9ORYZ:tr|A0A0E0F1W0|A0A0E0F1W0_9ORYZ:tr|A0A0E0F1V9|A0A0E0F1V9_9ORYZ:tr|B9F795|B9F795_ORYSJ:tr|A0A0E0F8Y7|A0A0E0F8Y7_9ORYZ:tr|B9G922|B9G922_ORYSJ:tr|A0A0E0F8Y5|A0A0E0F8Y5_9ORYZ:tr|I1R2M2|I1R2M2_ORYGL:tr|Q2RBC5|Q2RBC5_ORYSJ:tr|A0A0E0F1W8|A0A0E0F1W8_9ORYZ:tr|Q0IV31|Q0IV31_ORYSJ:tr|B8BIR0|B8BIR0_ORYSI:tr|A0A0D9XW90|A0A0D9XW90_9ORYZ:tr|A0A0E0NUS4|A0A0E0NUS4_ORYRU:tr|A0A0E0BN08|A0A0E0BN08_9ORYZ:tr|A0A0E0F8Y6|A0A0E0F8Y6_9ORYZ:tr|A0A0D9VSF4|A0A0D9VSF4_9ORYZ:tr|A0A0E0KBV6|A0A0E0KBV6_ORYPU:tr|I1P9X3|I1P9X3_ORYGL:tr|B8AKQ1|B8AKQ1_ORYSI:Q84Q83|TOC75_ORYSJ:tr|A0A0E0CYM6|A0A0E0CYM6_9ORYZ:tr|A0A0E0R3F3|A0A0E0R3F3_ORYRU:tr|A0A0D3FGS0|A0A0D3FGS0_9ORYZ:tr|A0A0E0GKR3|A0A0E0GKR3_ORYNI:tr|A0A0D3HHG9|A0A0D3HHG9_9ORYZ:tr|A0A0E0IXC4|A0A0E0IXC4_ORYNI</td></tr>
<tr><td>GNKRNPQAY</td><td>30.54</td><td>1046.5258</td><td>9</td><td>524.2700</td><td>5.37</td><td>Glutelin</td><td>tr|A0A0E0M7E9|A0A0E0M7E9_ORYPU:tr|T1T4G3|T1T4G3_ORYSI:tr|T1T4Y4|T1T4Y4_ORYSI:tr|A0A0E0JP14|A0A0E0JP14_ORYPU:tr|A1YQG5|A1YQG5_ORYSJ:tr|I1NRU9|I1NRU9_ORYGL:tr|A1YQG3|A1YQG3_ORYSJ:tr|A0A0E0N2T5|A0A0E0N2T5_ORYRU:P07730|GLUA2_ORYSJ:tr|A2WVB9|A2WVB9_ORYSI:tr|A2Z708|A2Z708_ORYSI:P07728|GLUA1_ORYSJ:tr</td></tr>
</table>

<table>
<tr><td></td><td></td><td></td><td></td><td></td><td></td><td></td><td>|I1QU95|I1QU95_ORYGL:tr|A0A0E0M7E8|A0A0E0M7E8_ORYPU:tr|A0A0E0FTI2|A0A0E0FTI2_ORYNI:tr|A0A0E0C821|A0A0E0C821_9ORYZ:tr|A0A0D3EUB5|A0A0D3EUB5_9ORYZ:tr|Q0JJ36|Q0JJ36_ORYSJ:tr|Q40689|Q40689_ORYSA:tr|A0A0E0BA65|A0A0E0BA65_9ORYZ:tr|A0A0E0IRV3|A0A0E0IRV3_ORYNI:tr|A0A0D3HDD6|A0A0D3HDD6_9ORYZ:tr|A0A0E0QYR7|A0A0E0QYR7_ORYRU:tr|A0A0E0BA63|A0A0E0BA63_9ORYZ:tr|A0A0E0BA64|A0A0E0BA64_9ORYZ:tr|A0A0E0IRV1|A0A0E0IRV1_ORYNI:tr|A0A0E0QYR5|A0A0E0QYR5_ORYRU:tr|A0A0E0QYR6|A0A0E0QYR6_ORYRU:tr|A0A0D3HDD5|A0A0D3HDD5_9ORYZ:tr|A0A0E0IRV2|A0A0E0IRV2_ORYNI</td></tr>
<tr><td>PAGTGPEHSQPAAA</td><td>30.51</td><td>1289.6000</td><td>14</td><td>645.8035</td><td>10.92</td><td>Serine phosphatase RsbU (Regulator of sigma subunit)</td><td>tr|A0A3N4U1Y9|A0A3N4U1Y9_9ACTN</td></tr>
<tr><td>VVVGTPGRVF</td><td>30.45</td><td>1029.5970</td><td>10</td><td>515.8063</td><td>18.09</td><td>Uncharacterized protein</td><td>tr|A0A0D9WTS3|A0A0D9WTS3_9ORYZ:P41376|IF4A1_ARATH:P41377|IF4A2_ARATH:tr|A0A0E0N9T3|A0A0E0N9T3_ORYRU:P35683|IF4A1_ORYSJ:Q6Z2Z4|IF4A3_ORYSJ:Q9CAI7|IF4A3_ARATH:tr|A0A0E0Q284|A0A0E0Q284_ORYRU:tr|A0A0E0LFA0|A0A0E0LFA0_ORYPU:tr|A2X0V4|A2X0V4_ORYSI:tr|A0A0E0JVG9|A0A0E0JVG9_ORYPU:tr|A0A0D3GKG6|A0A0D3GKG6_9ORYZ:tr|A0A0E0CFK9|A0A0E0CFK9_9ORYZ:tr|A2YGP5|A2YGP5_ORYSI</td></tr>
<tr><td>GQKPVTFF</td><td>30.17</td><td>922.4912</td><td>8</td><td>462.2526</td><td>19.39</td><td>Os10g0390500 protein</td><td>tr|A0A0P0XUE4|A0A0P0XUE4_ORYSJ:tr|A0A0E0IRN2|A0A0E0IRN2_ORYNI:tr|A0A0D3HD77|A0A0D3HD77_9ORYZ:tr|Q9S768|Q9S768_ORYSA:tr|I1QU38|I1QU38_ORYGL:tr|B8BGM4|B8BGM4_ORYSI:tr|Q338N8|Q338N8_ORYSJ:tr|A0A0E0QYK7|A0A0E0QYK7_ORYRU:tr|A0A0D3HD76|A0A0D3HD76_9ORYZ:tr|Q94HC5|Q94HC5_ORYSJ:tr|A0A0E0IRN1|A0A0E0IRN1_ORYNI:tr|A0A0E0QYK6|A0A0E0QYK6_ORYRU:tr|A0A0E0B9Z8|A0A0E0B9Z8_9ORYZ:tr|A0A0E0B9Z7|A0A0E0B9Z7_9ORYZ:tr|A0A0E0B9Z9|A0A0E0B9Z9_9ORYZ</td></tr>
<tr><td>SRPDFRF</td><td>27.12</td><td>923.4613</td><td>7</td><td>462.7385</td><td>13.96</td><td>Os10g0189100 protein</td><td>tr|A0A0E0EWF3|A0A0E0EWF3_9ORYZ:tr|A0A0E0EWF5|A0A0E0EWF5_9ORYZ:tr|A0A0E0EWF4|A0A0E0EWF4_9ORYZ:tr|J3N1F5|J3N1F5_ORYBR:tr|A0A0E0IQA4|A0A0E0IQA4_ORYNI:tr|A0A0D3HBW4|A0A0D3HBW4_9ORYZ:tr|A0A0D9XIC3|A0A0D9XIC3_9ORYZ:tr|B8BG13|B8BG13_ORYSI:tr|A0A0E0QX42|A0A0E0QX42_ORYRU:tr|A0A0E0B8J4|A0A0E0B8J4_9ORYZ:tr|Q33AE4|Q33AE4_ORYSJ:tr|I1QT43|I1QT43_ORYGL:tr|Q53QR8|Q53QR8_ORYSJ:tr|A0A0E0M659|A0A0E0M659_ORYPU</td></tr>
<tr><td>DWYKGPTLL</td><td>26.20</td><td>1091.5651</td><td>9</td><td>546.7906</td><td>26.90</td><td>Uncharacterized protein</td><td>tr|A0A0E0CW26|A0A0E0CW26_9ORYZ</td></tr>
</table>

SPESPRWLF	25.12	1117.5557	9	559.7860	25.25	Putative hexose transporter	tr|A0A0E0MR08|A0A0E0MR08_ORYRU:tr|B8AD38|B8AD38_ORYSI:tr|B9EZD0|B9EZD0_ORYSJ:tr|Q5ZCN2|Q5ZCN2_ORYSJ:tr|Q0JQX6|Q0JQX6_ORYSJ:tr|A0A0D3EJA4|A0A0D3EJA4_9ORYZ:tr|A0A0E0MR07|A0A0E0MR07_ORYRU:tr|A0A0E0JDQ5|A0A0E0JDQ5_ORYPU:tr|A0A0D3EJA3|A0A0D3EJA3_9ORYZ:tr|A0A0D9Y2U9|A0A0D9Y2U9_9ORYZ:tr|A0A0E0BWK8|A0A0E0BWK8_9ORYZ:tr|A0A0E0FFU0|A0A0E0FFU0_ORYNI:tr|A0A0E0BWK7|A0A0E0BWK7_9ORYZ
DTSKPFF	23.96	840.4017	7	421.2052	15.66	Uncharacterized protein	tr|A0A0E0Q5Q5|A0A0E0Q5Q5_ORYRU:tr|A0A0E0LIL6|A0A0E0LIL6_ORYPU
SSKPFFGGL	22.69	938.4861	9	470.2505	22.07	Nucleoside diphosphate kinase	tr|A2ZAA7|A2ZAA7_ORYSI:tr|A0A0E0EGM8|A0A0E0EGM8_9ORYZ:tr|A0A0E0MAU0|A0A0E0MAU0_ORYPU

Sub-fraction n. 24. Total: 13 identified peptides, all with rice protein accession. RT, retention time.

Peptide	-10lgP	Mass (Da)	Length (amino acid n.)	m/z	RT (min)	Accession	Protein
SIPTVGGVWY	57.33	1077.5494	10	539.7828	28.99	Prolamin	tr|Q9SAY8|Q9SAY8_ORYSA:tr|I1Q8Y6|I1Q8Y6_ORYGL:tr|Q6ZIX4|Q6ZIX4_ORYSJ:tr|A0A0E0Q5F7|A0A0E0Q5F7_ORYRU:tr|A0A0E0HLY0|A0A0E0HLY0_ORYNI
GIYPN(+.98)YYIAPR	56.60	1326.6608	11	664.3377	20.25	Prolamin	tr|Q9SAY8|Q9SAY8_ORYSA:tr|I1Q8Y6|I1Q8Y6_ORYGL:tr|Q6ZIX4|Q6ZIX4_ORYSJ:tr|A0A0E0Q5F7|A0A0E0Q5F7_ORYRU:tr|A0A0E0HLY0|A0A0E0HLY0_ORYNI
TNPWHSPRQG	54.99	1178.5581	10	590.2863	6.41	Glutelin	tr|A0A0D9VE94|A0A0D9VE94_9ORYZ:tr|B9F4T2|B9F4T2_ORYSJ:tr|A0A0E0NCF1|A0A0E0NCF1_ORYRU:tr|A0A0D3F334|A0A0D3F334_9ORYZ:tr|B9F4T1|B9F4T1_ORYSJ:tr|B9F4T3|B9F4T3_ORYSJ:tr|B8AEZ5|B8AEZ5_ORYSI:tr|T1T6C4|T1T6C4_ORYSI:Q02897|GLUB2_ORYSJ:tr|A1YQH6|A1YQH6_ORYSJ:tr|I1NZ08|I1NZ08_ORYGL:tr|A1YQH4|A1YQH4_ORYSJ:tr|Q0E2D5|Q0E2D5_ORYSJ:tr|A1YQH5|A1YQH5_ORYSJ:tr|A0A0E0CIL1|A0A0E0CIL1_9ORYZ:tr|A0A0E0G6R1|A0A0E0G6R1_ORYNI:P14323|GLUB1_ORYSJ:tr|A0A0E0NCF3|A0A0E0NCF3_ORYRU:tr|I1NZ10|I1NZ10_ORYGL:tr|Q0E2D2|Q0E2D2_ORYSJ:tr|T1T4F0|T1T4F0_ORYSI:tr|A0A0D9YPX0|A0A0D9YPX0_9ORYZ:tr|A0A0D3F336|A0A0D3F336_9ORYZ:tr|A0A0E0CIL2|A0A0E0CIL2_9ORYZ:tr|A2X2Z8|A2X2Z8_ORYSI
TNPWHSPR	41.01	993.4780	8	497.7463	6.69	Glutelin	tr|A0A0D9VE94|A0A0D9VE94_9ORYZ:tr|B9F4T2|B9F4T2_ORYSJ:tr|A0A0E0NCF1|A0A0E0NCF1_ORYRU:tr|A0A0D3F334|A0A0D3F334_9ORYZ:tr|B9F4T1|B9F4T1_ORYSJ:tr|B9F4T3|B9F4T3_ORYSJ:tr|B8AEZ5|B8AEZ5_ORYSI:tr|T1T6C4|T1T6C4_ORYSI:Q02897|GLUB2_ORYSJ:tr|A1YQH6|

							A1YQH6_ORYSJ:tr|I1NZ08|I1NZ08_ORYGL:tr|A1YQH4|A1YQH4_ORYSJ:tr|Q0E2D5|Q0E2D5_ORYSJ:tr|A1YQH5|A1YQH5_ORYSJ:tr|A0A0E0CIL1|A0A0E0CIL1_9ORYZ:tr|A0A0E0G6R1|A0A0E0G6R1_ORYNI:P14323|GLUB1_ORYSJ:tr|A0A0E0NCF3|A0A0E0NCF3_ORYRU:tr|I1NZ10|I1NZ10_ORYGL:tr|Q0E2D2|Q0E2D2_ORYSJ:tr|T1T4F0|T1T4F0_ORYSI:tr|A0A0D9YPX0|A0A0D9YPX0_9ORYZ:tr|A0A0D3F336|A0A0D3F336_9ORYZ:tr|A0A0E0CIL2|A0A0E0CIL2_9ORYZ:tr|A2X2Z8|A2X2Z8_ORYSI
HGAFTPR	39.11	784.3980	7	393.2070	5.71	Glutelin	tr|A0A0D9VE94|A0A0D9VE94_9ORYZ:tr|B9F4T2|B9F4T2_ORYSJ:tr|A0A0E0NCF1|A0A0E0NCF1_ORYRU:tr|A0A0D3F334|A0A0D3F334_9ORYZ:tr|B9F4T1|B9F4T1_ORYSJ:tr|B9F4T3|B9F4T3_ORYSJ:tr|B8AEZ5|B8AEZ5_ORYSI:tr|T1T6C4|T1T6C4_ORYSI:Q02897|GLUB2_ORYSJ:tr|A1YQH6|A1YQH6_ORYSJ:tr|I1NZ08|I1NZ08_ORYGL:tr|A1YQH4|A1YQH4_ORYSJ:tr|Q0E2D5|Q0E2D5_ORYSJ:tr|A1YQH5|A1YQH5_ORYSJ:tr|A0A0E0CIL1|A0A0E0CIL1_9ORYZ:tr|A0A0E0G6R1|A0A0E0G6R1_ORYNI:P14323|GLUB1_ORYSJ:tr|A0A0E0NCF3|A0A0E0NCF3_ORYRU:tr|I1NZ10|I1NZ10_ORYGL:tr|Q0E2D2|Q0E2D2_ORYSJ:tr|T1T4F0|T1T4F0_ORYSI:tr|A0A0D9YPX0|A0A0D9YPX0_9ORYZ:tr|A0A0D3F336|A0A0D3F336_9ORYZ:tr|A0A0E0CIL2|A0A0E0CIL2_9ORYZ
HS(-18.01)AFTPR	33.83	796.3980	7	399.2061	5.98	Uncharacterized protein	tr|A2X2Z8|A2X2Z8_ORYSI
H(+40.03)GAFTPR	31.31	824.4293	7	413.2221	8.51	Glutelin	tr|A0A0D9VE94|A0A0D9VE94_9ORYZ:tr|B9F4T2|B9F4T2_ORYSJ:tr|A0A0E0NCF1|A0A0E0NCF1_ORYRU:tr|A0A0D3F334|A0A0D3F334_9ORYZ:tr|B9F4T1|B9F4T1_ORYSJ:tr|B9F4T3|B9F4T3_ORYSJ:tr|B8AEZ5|B8AEZ5_ORYSI:tr|T1T6C4|T1T6C4_ORYSI:Q02897|GLUB2_ORYSJ:tr|A1YQH6|A1YQH6_ORYSJ:tr|I1NZ08|I1NZ08_ORYGL:tr|A1YQH4|A1YQH4_ORYSJ:tr|Q0E2D5|Q0E2D5_ORYSJ:tr|A1YQH5|A1YQH5_ORYSJ:tr|A0A0E0CIL1|A0A0E0CIL1_9ORYZ:tr|A0A0E0G6R1|A0A0E0G6R1_ORYNI:P14323|GLUB1_ORYSJ:tr|A0A0E0NCF3|A0A0E0NCF3_ORYRU:tr|I1NZ10|I1NZ10_ORYGL:tr|Q0E2D2|Q0E2D2_ORYSJ:tr|T1T4F0|T1T4F0_ORYSI:tr|A0A0D9YPX0|A0A0D9YPX0_9ORYZ:tr|A0A0D3F336|A0A0D3F336_9ORYZ:tr|A0A0E0CIL2|A0A0E0CIL2_9ORYZ
FNVPSRY	28.94	881.4395	7	441.7273	14.44	Os05g0329200 protein	tr|A0A0E0HCR9|A0A0E0HCR9_ORYNI:tr|C7J346|C7J346_ORYSJ:tr|A0A0P0WKY1|A0A0P0WKY1_ORYSJ:tr|A0A0D9ZXI8|A0A0D9ZXI8_9ORYZ:tr|Q5W6A5|Q5W6A5_ORYSJ:tr|A0A0E0PKU6|A0A0E0PKU6_ORYRU:tr|Q5W695|Q5W695_ORYSJ:tr|Q43603|Q43603_ORYSA:tr|P93412|P93412_ORYSJ:tr|P93413|P93413_ORYSJ:tr|Q0DJ44|Q0DJ44_ORYSJ:P0C5E5|PRO7_ORYSI:tr|A0A0E0PKU8|A0A0E0PKU8_ORYRU:tr|A1YQE8|A1YQE8_ORYSJ:tr|I1PUH1|I1PUH1_ORYGL:tr|Q5W6A3|Q5W6A3_ORYSJ:tr|A0A0P0WKT6|A0A0P0WKT6_ORYSJ:tr|E0X6Y1|E0X6Y1_ORYSJ:tr|Q5W755|Q5W755_ORYSJ:tr|Q5EFA3|Q5EFA3_9ORYZ:tr|Q5EFA4|Q5EFA4_ORYNI:Q0DJ45|PRO7_ORYSJ:tr|A0A0E0PKU9|A0A0E0PKU9_ORYRU:tr|A0A0D3

							G662|A0A0D3G662_9ORYZ:tr|A0A0N7KKJ8|A0A0N7KKJ8_ORYSJ:tr|A0A0D3G661|A0A0D3G661_9ORYZ
GPNVPSRW	25.06	911.4613	8	456.7383	13.78	Uncharacterized protein	tr|A0A0E0CST4|A0A0E0CST4_9ORYZ
HNVVKFR	25.01	898.5137	7	450.2607	7.41	NADH-cytochrome b5 reductase	tr|A0A0D9WHY7|A0A0D9WHY7_9ORYZ
AEAAAAEGGVS	23.18	931.4246	11	466.7231	21.29	F-box protein interaction domain containing protein, expressed	tr|A0A0D3HD85|A0A0D3HD85_9ORYZ:tr|A2Z6V4|A2Z6V4_ORYSI:tr|Q7XEZ8|Q7XEZ8_ORYSJ:tr|A3C4D4|A3C4D4_ORYSJ:tr|A0A0E0QYL5|A0A0E0QYL5_ORYRU:tr|A0A0E0IRP0|A0A0E0IRP0_ORYNI:tr|A0A0E0BA03|A0A0E0BA03_9ORYZ:tr|I1QU45|I1QU45_ORYGL
AIVLPPWVA	22.78	964.5746	9	483.2954	34.28	Sucrose synthase	tr|A0A0D9WMW0|A0A0D9WMW0_9ORYZ:tr|A0A0E0HM40|A0A0E0HM40_ORYNI:tr|A2YA91|A2YA91_ORYSI:tr|A0A0D3GDS1|A0A0D3GDS1_9ORYZ:tr|I1Q096|I1Q096_ORYGL:P30298|SUS2_ORYSJ:tr|A0A0E0A682|A0A0E0A682_9ORYZ:tr|J3MC18|J3MC18_ORYBR:tr|A0A0E0L8X0|A0A0E0L8X0_ORYPU:tr|I3QD82|I3QD82_ORYSJ:tr|A0A0E0HM39|A0A0E0HM39_ORYNI:tr|A0A0E0PUM1|A0A0E0PUM1_ORYRU:tr|A0A0E0HM38|A0A0E0HM38_ORYNI
AIVIPPWVA	22.78	964.5746	9	483.2954	34.28	Sucrose synthase	tr|A0A0D9X1I4|A0A0D9X1I4_9ORYZ:tr|J3MMZ8|J3MMZ8_ORYBR

PROTAMEX fraction R1

Sub-fraction n. 12. Total: 19 identified peptides, all with rice protein accession. RT, retention time.

Peptide	-10lgP	Mass (Da)	Length (amino acid n.)	m/z	RT (min)	Protein	Accession
NLNNNPYFKGT	51.96	1280.6150	11	641.3158	14.50	Granule-bound starch synthase I	tr|A0A0H4BM25|A0A0H4BM25_ORYSI:tr|A0A0E0DW79|A0A0E0DW79_9ORYZ:tr|A8QXE7|A8QXE7_ORYSI:tr|V5NEJ7|V5NEJ7_ORYSA:tr|A0A0E0DW80|A0A0E0DW80_9ORYZ:tr|A0EQH2|A0EQH2_ORYSJ:tr|A0EQE0|A0EQE0_ORYSA:tr|A0EQK7|A0EQK7_ORYRU:tr|A0EQD4|A0EQD4_ORYSI

							:tr|A0EQK5|A0EQK5_ORYRU:tr|A0EQK6|A0EQK6_ORYRU:tr|A0EQK4|A0EQK4_ORYRU:P0C585|SSG1_ORYSA:tr|B8XEJ0|B8XEJ0_ORYSJ:tr|B8XEJ8|B8XEJ8_ORYSA:tr|B8XEK3|B8XEK3_ORYSA:tr|D0TZY6|D0TZY6_ORYSI:tr|A0A3Q9T378|A0A3Q9T378_ORYSA:tr|D3U2H9|D3U2H9_ORYSA:tr|B8XEJ7|B8XEJ7_ORYSA:tr|A0A3Q9T3Z7|A0A3Q9T3Z7_ORYSA:tr|B8XEJ2|B8XEJ2_ORYSA:tr|B8XEK2|B8XEK2_ORYSA:tr|A0A076FRI5|A0A076FRI5_ORYSJ:Q42968|SSG1_ORYGL:A2Y8X2|SSG1_ORYSI:tr|B1B5Z0|B1B5Z0_ORYSI:tr|B1B5Z1|B1B5Z1_ORYSI:tr|C8CBL1|C8CBL1_ORYSJ:tr|B2KNV0|B2KNV0_ORYRU:tr|A0A0D9WLF6|A0A0D9WLF6_9ORYZ:tr|A0A0E0A4K1|A0A0E0A4K1_9ORYZ
VDTGRGPIMYY	44.76	1270.6016	11	636.3087	18.68	Uncharacterized protein	tr|A0A0D9Z6Z6|A0A0D9Z6Z6_9ORYZ:tr|A0A0D3FI73|A0A0D3FI73_9ORYZ:tr|I1PB32|I1PB32_ORYGL:tr|A0A0E0NUP7|A0A0E0NUP7_ORYRU:tr|A0A0E0D051|A0A0E0D051_9ORYZ:tr|A2XGF3|A2XGF3_ORYSI:tr|Q10LT1|Q10LT1_ORYSJ:tr|A0A0E0GM25|A0A0E0GM25_ORYNI
GKTVFDGVLRPG	42.24	1244.6876	12	623.3521	17.49	Glutelin	tr|B9F4T3|B9F4T3_ORYSJ:tr|A0A0E0NCF3|A0A0E0NCF3_ORYRU:tr|Q0E2D2|Q0E2D2_ORYSJ:P14323|GLUB1_ORYSJ:tr|B9F4T2|B9F4T2_ORYSJ:tr|A0A0E0NCF1|A0A0E0NCF1_ORYRU:tr|B9F4T1|B9F4T1_ORYSJ:tr|B8AEZ5|B8AEZ5_ORYSI:tr|T1T6C4|T1T6C4_ORYSI:tr|A1YQH4|A1YQH4_ORYSJ:tr|Q0E2D5|Q0E2D5_ORYSJ:Q02897|GLUB2_ORYSJ:tr|A1YQH6|A1YQH6_ORYSJ:tr|A1YQH5|A1YQH5_ORYSJ:tr|A0A0E0CIL1|A0A0E0CIL1_9ORYZ:tr|I1NZ10|I1NZ10_ORYGL:tr|T1T4F0|T1T4F0_ORYSI:tr|A0A0E0G6R1|A0A0E0G6R1_ORYNI:tr|A0A0D9YPX0|A0A0D9YPX0_9ORYZ:tr|A0A0D3F336|A0A0D3F336_9ORYZ:tr|A0A0E0CIL2|A0A0E0CIL2_9ORYZ:tr|A0A0D3F337|A0A0D3F337_9ORYZ:tr|A2X301|A2X301_ORYSI:tr|A0A0E0JY04|A0A0E0JY04_ORYPU:tr|A0A0D3F334|A0A0D3F334_9ORYZ:tr|I1NZ08|I1NZ08_ORYGL
APIYTQPR	41.23	944.5079	8	473.2617	7.88	Glucose-1-phosphate adenylyltransferase	tr|D4AIA3|D4AIA3_ORYSI:tr|B7EVB8|B7EVB8_ORYSJ:tr|A0A0E0LU24|A0A0E0LU24_ORYPU:tr|A0A0D3GZB2|A0A0D3GZB2_9ORYZ:tr|A0A0E0H7V9|A0A0E0H7V9_ORYNI:tr|A0A0E0QHR8|A0A0E0QHR8_ORYRU:tr|B8XEF2|B8XEF2_ORYSI:tr|B8XEE9|B8XEE9_ORYSI:tr|B8XED8|B8XED8_ORYSA:tr|B8XEE1|B8XEE1_ORYSI:tr|B8XED9|B8XED9_ORYSI:tr|B8XED7|B8XED7_ORYSI:tr|B8XEE8|B8XEE8_ORYSA:tr|B8XEE2|B8XEE2_ORYSJ:tr|B8XEF0|B8XEF0_ORYSI:tr|B8XEE5|B8XEE5_ORYSJ:tr|B8XEE6|B8XEE6_ORYSA:tr|A2YU91|A2YU91_ORYSI:tr|A0A0D3GZB1|A0A0D3GZB1_9ORYZ:tr|A0A0D9X6Z4|A0A0D9X6Z4_9ORYZ:tr|A0A0D9X6Z3|A0A0D9X6Z3_9ORYZ:tr|A0A0E0QHR7|A0A0E0QHR7_ORYRU:tr|A0A0E0H7V8|A0A0E0H7V8_ORYNI:tr|A0A0E0LU23|A0A0E0LU23_ORYPU
KGGIPIGIGK	37.79	938.5912	10	470.3041	12.29	Glucose-1-phosphate adenylyltransferase	tr|D4AIA3|D4AIA3_ORYSI:tr|B7EVB8|B7EVB8_ORYSJ:tr|A0A0E0LU24|A0A0E0LU24_ORYPU:tr|A0A0D3GZB2|A0A0D3GZB2_9ORYZ:tr|A0A0E0H7V9|A0A0E0H7V9_ORYNI:tr|A0A0E0QHR8|A0A0E0QHR8_ORYRU:tr|B8XEF2|B8XEF2_ORYSI:tr|B8XEE9|B8XEE9_ORYSI:tr|B8XED8|B8XED8_ORYSA:tr|B8XEE1|B8XEE1_ORYSI:tr|B8XED9|B8XED9_ORYSI:tr|B8XED7|B8XED7_ORYSI:tr|B8XEE8|B8XEE8_ORYSA:tr|B8XEE2|B8XEE2_ORYSJ:tr|B8XEF0|B8XEF0_ORYSI:tr|B8XEE5|B8XEE5_ORYSJ:tr|B8XEE6|B

<table>
<tr><td></td><td></td><td></td><td></td><td></td><td></td><td></td><td>8XEE6_ORYSA:tr|A2YU91|A2YU91_ORYSI:tr|A0A0D3GZB1|A0A0D3GZB1_9ORYZ:tr|A0A0D9X6Z4|A0A0D9X6Z4_9ORYZ:tr|A0A0D9X6Z3|A0A0D9X6Z3_9ORYZ:tr|A0A0E0QHR7|A0A0E0QHR7_ORYRU:tr|A0A0E0H7V8|A0A0E0H7V8_ORYNI:tr|A0A0E0LU23|A0A0E0LU23_ORYPU</td></tr>
<tr><td>HGAFTPR</td><td>37.68</td><td>784.3980</td><td>7</td><td>393.2070</td><td>5.46</td><td>Glutelin</td><td>tr|B9F4T3|B9F4T3_ORYSJ:tr|A0A0E0NCF3|A0A0E0NCF3_ORYRU:tr|Q0E2D2|Q0E2D2_ORYSJ:P14323|GLUB1_ORYSJ:tr|B9F4T2|B9F4T2_ORYSJ:tr|A0A0E0NCF1|A0A0E0NCF1_ORYRU:tr|B9F4T1|B9F4T1_ORYSJ:tr|B8AEZ5|B8AEZ5_ORYSI:tr|T1T6C4|T1T6C4_ORYSI:tr|A1YQH4|A1YQH4_ORYSJ:tr|Q0E2D5|Q0E2D5_ORYSJ:Q02897|GLUB2_ORYSJ:tr|A1YQH6|A1YQH6_ORYSJ:tr|A1YQH5|A1YQH5_ORYSJ:tr|A0A0E0CIL1|A0A0E0CIL1_9ORYZ:tr|I1NZ10|I1NZ10_ORYGL:tr|T1T4F0|T1T4F0_ORYSI:tr|A0A0E0G6R1|A0A0E0G6R1_ORYNI:tr|A0A0D9YPX0|A0A0D9YPX0_9ORYZ:tr|A0A0D3F336|A0A0D3F336_9ORYZ:tr|A0A0E0CIL2|A0A0E0CIL2_9ORYZ:tr|A0A0D3F337|A0A0D3F337_9ORYZ:tr|A2X301|A2X301_ORYSI:tr|A0A0E0JY04|A0A0E0JY04_ORYPU:tr|A0A0D3F334|A0A0D3F334_9ORYZ:tr|I1NZ08|I1NZ08_ORYGL</td></tr>
<tr><td>KLPPVGPY</td><td>37.23</td><td>869.5010</td><td>8</td><td>435.7585</td><td>15.37</td><td>Os08g0545200 protein</td><td>tr|A0A0E0LXL4|A0A0E0LXL4_ORYPU:tr|B8B9C4|B8B9C4_ORYSI:tr|A0A0E0IFD3|A0A0E0IFD3_ORYNI:tr|B8B9C5|B8B9C5_ORYSI:tr|I1QLA2|I1QLA2_ORYGL:tr|Q6ZBH2|Q6ZBH2_ORYSJ:tr|A0A0E0QLZ3|A0A0E0QLZ3_ORYRU:tr|A0A0E0AYG5|A0A0E0AYG5_9ORYZ:tr|A0A0D3H2T7|A0A0D3H2T7_9ORYZ:tr|A6N1S7|A6N1S7_ORYSI</td></tr>
<tr><td>GYVGANPRL</td><td>35.82</td><td>945.5032</td><td>9</td><td>473.7595</td><td>13.01</td><td>Os04g0404400 protein</td><td>tr|Q0JDG9|Q0JDG9_ORYSJ:tr|A0A0P0WA63|A0A0P0WA63_ORYSJ:tr|A0A0D3FVG1|A0A0D3FVG1_9ORYZ:tr|A2XT28|A2XT28_ORYSI:tr|Q7X6I8|Q7X6I8_ORYSJ:tr|Q01L47|Q01L47_ORYSA:tr|I1PKX3|I1PKX3_ORYGL:tr|A0A0D9ZKB8|A0A0D9ZKB8_9ORYZ:tr|A0A0E0H0B3|A0A0E0H0B3_ORYNI:tr|A0A0E0P8P0|A0A0E0P8P0_ORYRU</td></tr>
<tr><td>HRDFFLA</td><td>28.71</td><td>904.4555</td><td>7</td><td>453.2358</td><td>16.56</td><td>Gt3</td><td>tr|A0A0E0D2D4|A0A0E0D2D4_9ORYZ:tr|A0A0E0NWT6|A0A0E0NWT6_ORYRU:tr|B7U2J6|B7U2J6_ORYSJ:tr|T1T5D8|T1T5D8_ORYSI:tr|J3M4W6|J3M4W6_ORYBR:tr|A0A0D3FK24|A0A0D3FK24_9ORYZ:tr|I1PCG0|I1PCG0_ORYGL:tr|Q10JA8|Q10JA8_ORYSJ:tr|A0A0E0KFA5|A0A0E0KFA5_ORYPU:Q09151|GLUA3_ORYSJ:tr|A0A0E0ITU1|A0A0E0ITU1_ORYNI:tr|A0A0D9Z962|A0A0D9Z962_9ORYZ:tr|B9F952|B9F952_ORYSJ:tr|B8AKE2|B8AKE2_ORYSI:tr|C7DQE9|C7DQE9_ORYSJ:tr|A0A0D9XVA4|A0A0D9XVA4_9ORYZ:tr|A0A0D9V5Z5|A0A0D9V5Z5_9ORYZ:tr|A0A0D9V5Z4|A0A0D9V5Z4_9ORYZ</td></tr>
<tr><td>NNNPYFKG</td><td>27.33</td><td>952.4402</td><td>8</td><td>477.2284</td><td>9.14</td><td>Granule-bound starch synthase I</td><td>tr|A0A0H4BM25|A0A0H4BM25_ORYSI:tr|A0A0E0DW79|A0A0E0DW79_9ORYZ:tr|A8QXE7|A8QXE7_ORYSI:tr|V5NEJ7|V5NEJ7_ORYSA:tr|A0A0E0DW80|A0A0E0DW80_9ORYZ:tr|A0EQH2|A0EQH2_ORYSJ:tr|A0EQE0|A0EQE0_ORYSA:tr|A0EQK7|A0EQK7_ORYRU:tr|A0EQD4|A0EQD4_ORYSI:tr|A0EQK5|A0EQK5_ORYRU:tr|A0EQK6|A0EQK6_ORYRU:tr|A0EQK4|A0EQK4_ORYRU:P0C585|SSG1_ORYSA:tr|B8XEJ0|B8XEJ0_ORYSJ:tr|B8XEJ8|B8XEJ8_ORYSA:tr|B8XEK3|B8XEK3_ORYSA:tr|D0TZY6|D0TZY6_OR</td></tr>
</table>

<table>
<tr><td></td><td></td><td></td><td></td><td></td><td></td><td></td><td>YSI:tr|A0A3Q9T378|A0A3Q9T378_ORYSA:tr|D3U2H9|D3U2H9_ORYSA:tr|B8XEJ7|B8XEJ7_ORYSA:tr|A0A3Q9T3Z7|A0A3Q9T3Z7_ORYSA:tr|B8XEJ2|B8XEJ2_ORYSA:tr|B8XEK2|B8XEK2_ORYSA:tr|A0A076FRI5|A0A076FRI5_ORYSJ:Q42968|SSG1_ORYGL:A2Y8X2|SSG1_ORYSI:tr|B1B5Z0|B1B5Z0_ORYSI:tr|B1B5Z1|B1B5Z1_ORYSI:tr|C8CBL1|C8CBL1_ORYSJ:tr|B2KNV0|B2KNV0_ORYRU:tr|A0A0D9WLF6|A0A0D9WLF6_9ORYZ:tr|A0A0E0A4K1|A0A0E0A4K1_9ORYZ</td></tr>
<tr><td>LENVRFY</td><td>27.31</td><td>939.4814</td><td>7</td><td>470.7484</td><td>17.50</td><td>Phosphoglycerate kinase</td><td>tr|A2Y650|A2Y650_ORYSI:tr|A6N1P1|A6N1P1_ORYSI:tr|Q75K90|Q75K90_ORYSJ:tr|A0A0N7KL10|A0A0N7KL10_ORYSJ:tr|C7IXG8|C7IXG8_ORYSJ:tr|Q655T1|Q655T1_ORYSJ:tr|A0A0D9WT19|A0A0D9WT19_9ORYZ:tr|A2YG06|A2YG06_ORYSI:tr|A0A0E0Q1D7|A0A0E0Q1D7_ORYRU:tr|A0A0D3GJQ6|A0A0D3GJQ6_9ORYZ:tr|A0A0E0E560|A0A0E0E560_9ORYZ:tr|A0A0E0LEG8|A0A0E0LEG8_ORYPU:tr|A0A0E0NAE9|A0A0E0NAE9_ORYRU:tr|Q09HR1|Q09HR1_ORYSI:tr|Q09HR2|Q09HR2_ORYSI:tr|A0A0D9YMV6|A0A0D9YMV6_9ORYZ:tr|Q6H6C7|Q6H6C7_ORYSJ:tr|B8AIH2|B8AIH2_ORYSI:tr|A0A0E0CGA6|A0A0E0CGA6_9ORYZ:tr|A0A0D3G9D3|A0A0D3G9D3_9ORYZ:tr|B9ETR7|B9ETR7_ORYSJ:tr|A0A0E0CGA5|A0A0E0CGA5_9ORYZ:tr|A0A0E0N3P8|A0A0E0N3P8_ORYRU:tr|B8AAV9|B8AAV9_ORYSI:tr|A0A0E0C8V8|A0A0E0C8V8_9ORYZ:tr|A0A0E0DTH3|A0A0E0DTH3_9ORYZ:tr|A0A0P0WP33|A0A0P0WP33_ORYSJ:tr|A0A0E0PPF6|A0A0E0PPF6_ORYRU:tr|A0A0E0L498|A0A0E0L498_ORYPU:tr|A0A0E0JW27|A0A0E0JW27_ORYPU</td></tr>
<tr><td>GKGYVGL</td><td>23.86</td><td>692.3857</td><td>7</td><td>347.2008</td><td>12.20</td><td>Glutelin</td><td>tr|A0A0D3F331|A0A0D3F331_9ORYZ:tr|A0A0E0NCE4|A0A0E0NCE4_ORYRU:tr|A0A0D9YPW1|A0A0D9YPW1_9ORYZ:tr|A0A0E0FMV5|A0A0E0FMV5_ORYNI:tr|A0A0E0JXZ6|A0A0E0JXZ6_ORYPU:Q6K508|GLUD1_ORYSJ:tr|I1NZ02|I1NZ02_ORYGL:tr|A0A0E0NCE6|A0A0E0NCE6_ORYRU:tr|T1T4G5|T1T4G5_ORYSI:tr|M1G949|M1G949_ORYSI:tr|A2X2Z1|A2X2Z1_ORYSI</td></tr>
<tr><td>SPFRVPIA</td><td>22.60</td><td>885.5072</td><td>8</td><td>443.7615</td><td>20.18</td><td>Pyruvate, phosphate dikinase</td><td>tr|J3M716|J3M716_ORYBR:tr|B9FPJ4|B9FPJ4_ORYSJ:tr|A0A0E0PMB6|A0A0E0PMB6_ORYRU:tr|B8AYC1|B8AYC1_ORYSI:tr|A0A0D9ZZ05|A0A0D9ZZ05_9ORYZ:Q6AVA8-2|PPDK1_ORYSJ:tr|A0A0E0DR93|A0A0E0DR93_9ORYZ:tr|A0A0D9VU47|A0A0D9VU47_9ORYZ:tr|J3LQ10|J3LQ10_ORYBR:tr|A0A0E0NWX2|A0A0E0NWX2_ORYRU:tr|O82032|O82032_ORYSI:tr|A0A0D3FK58|A0A0D3FK58_9ORYZ:tr|A0A0D9Z998|A0A0D9Z998_9ORYZ:tr|I1PCI9|I1PCI9_ORYGL:Q75KR1|PPDK2_ORYSJ:tr|A2XIA2|A2XIA2_ORYSI:tr|A0A0D3G7H5|A0A0D3G7H5_9ORYZ:tr|A0A0D9WGC7|A0A0D9WGC7_9ORYZ:tr|A0A0D9WGC6|A0A0D9WGC6_9ORYZ:Q6AVA8|PPDK1_ORYSJ:tr|I1PVJ3|I1PVJ3_ORYGL:tr|A0A0E0ITX7|A0A0E0ITX7_ORYNI:tr|A0A0E0ITX8|A0A0E0ITX8_ORYNI</td></tr>
<tr><td>FGGAAVPP</td><td>21.74</td><td>714.3701</td><td>8</td><td>358.1929</td><td>11.12</td><td>Uncharacterized protein</td><td>tr|A0A0E0FCL2|A0A0E0FCL2_9ORYZ</td></tr>
</table>

RNAFGGVL	21.06	832.4555	8	417.2357	11.83	Uncharacterized protein	tr|A0A0E0L4R3|A0A0E0L4R3_ORYPU:tr|A0A0D9WIS4|A0A0D9WIS4_9ORYZ:tr|J3M8Z0|J3M8Z0_ORYBR
NLNNNPYFKG	20.61	1179.5673	10	590.7914	14.13	Granule-bound starch synthase I	tr|A0A0H4BM25|A0A0H4BM25_ORYSI:tr|A0A0E0DW79|A0A0E0DW79_9ORYZ:tr|A8QXE7|A8QXE7_ORYSI:tr|V5NEJ7|V5NEJ7_ORYSA:tr|A0A0E0DW80|A0A0E0DW80_9ORYZ:tr|A0EQH2|A0EQH2_ORYSJ:tr|A0EQE0|A0EQE0_ORYSA:tr|A0EQK7|A0EQK7_ORYRU:tr|A0EQD4|A0EQD4_ORYSI:tr|A0EQK5|A0EQK5_ORYRU:tr|A0EQK6|A0EQK6_ORYRU:tr|A0EQK4|A0EQK4_ORYRU:P0C585|SSG1_ORYSA:tr|B8XEJ0|B8XEJ0_ORYSJ:tr|B8XEJ8|B8XEJ8_ORYSA:tr|B8XEK3|B8XEK3_ORYSA:tr|D0TZY6|D0TZY6_ORYSI:tr|A0A3Q9T378|A0A3Q9T378_ORYSA:tr|D3U2H9|D3U2H9_ORYSA:tr|B8XEJ7|B8XEJ7_ORYSA:tr|A0A3Q9T3Z7|A0A3Q9T3Z7_ORYSA:tr|B8XEJ2|B8XEJ2_ORYSA:tr|B8XEK2|B8XEK2_ORYSA:tr|A0A076FRI5|A0A076FRI5_ORYSJ:Q42968|SSG1_ORYGL:A2Y8X2|SSG1_ORYSI:tr|B1B5Z0|B1B5Z0_ORYSI:tr|B1B5Z1|B1B5Z1_ORYSI:tr|C8CBL1|C8CBL1_ORYSJ:tr|B2KNV0|B2KNV0_ORYRU:tr|A0A0D9WLF6|A0A0D9WLF6_9ORYZ:tr|A0A0E0A4K1|A0A0E0A4K1_9ORYZ
LGSGGKFP	20.32	761.4072	8	381.7117	11.29	Uncharacterized protein	tr|B8AHJ6|B8AHJ6_ORYSI:tr|B9EZY1|B9EZY1_ORYSJ
DWYKGPT	20.21	865.3970	7	433.7066	14.23	Elongation factor	tr|E7BJ60|E7BJ60_ORYSI:tr|A0A0E0NQG9|A0A0E0NQG9_ORYRU:tr|A0A0P0VTT8|A0A0P0VTT8_ORYSJ:tr|A0A0E0CW33|A0A0E0CW33_9ORYZ:tr|A0A0E0CW26|A0A0E0CW26_9ORYZ:tr|Q10QZ5|Q10QZ5_ORYSJ:tr|A0A0E0GHL8|A0A0E0GHL8_ORYNI:tr|A0A0N7KGP4|A0A0N7KGP4_ORYSJ:tr|B9FBM7|B9FBM7_ORYSJ:tr|A0A0D9VSS7|A0A0D9VSS7_9ORYZ:tr|A0A0D3FEJ0|A0A0D3FEJ0_9ORYZ:O64937|EF1A_ORYSJ:tr|A0A0E0GHL3|A0A0E0GHL3_ORYNI:tr|J3LKK4|J3LKK4_ORYBR:tr|A0A0E0K9J4|A0A0E0K9J4_ORYPU:tr|A0A1L2JKK1|A0A1L2JKK1_ORYSA:tr|Q10QZ6|Q10QZ6_ORYSJ:tr|A0A0D3FEJ3|A0A0D3FEJ3_9ORYZ:tr|B8APM5|B8APM5_ORYSI:tr|I1P851|I1P851_ORYGL:tr|A0A0E0K9J0|A0A0E0K9J0_ORYPU:tr|Q10QZ4|Q10QZ4_ORYSJ:tr|A0A0D3FEJ2|A0A0D3FEJ2_9ORYZ:tr|A0A0D3FEJ4|A0A0D3FEJ4_9ORYZ:tr|A0A0D3FEJ1|A0A0D3FEJ1_9ORYZ
FYDPKTPF	20.04	1013.4858	8	507.7507	19.35	ADP-glucose pyrophosphorylase large subunit	tr|B8XEC2|B8XEC2_ORYSI

Sub-fraction n. 14. Total: 21 identified peptides, all with rice protein accession. RT, retention time.

Peptide	-10lgP	Mass (Da)	Length (amino acid n.)	m/z	RT (min)	Protein	Accession
NLNNNPYFKGT	51.56	1280.6150	11	641.3151	14.30	Granule-bound starch synthase I	tr|A0A0H4BM25|A0A0H4BM25_ORYSI:tr|A0A0E0DW79|A0A0E0DW79_9ORYZ:tr|A8QXE7|A8QXE7_ORYSI:tr|V5NEJ7|V5NEJ7_ORYSA:tr|A0A0E0DW80|A0A0E0DW80_9ORYZ:tr|A0EQH2|A0EQH2_ORYSJ:tr|A0EQE0|A0EQE0_ORYSA:tr|A0EQK7|A0EQK7_ORYRU:tr|A0EQD4|A0EQD4_ORYSI:tr|A0EQK5|A0EQK5_ORYRU:tr|A0EQK6|A0EQK6_ORYRU:tr|A0EQK4|A0EQK4_ORYRU:P0C585|SSG1_ORYSA:tr|B8XEJ8|B8XEJ8_ORYSA:tr|B8XEK3|B8XEK3_ORYSA:tr|D0TZY6|D0TZY6_ORYSI:tr|A0A3Q9T378|A0A3Q9T378_ORYSA:tr|D3U2H9|D3U2H9_ORYSA:tr|B8XEJ7|B8XEJ7_ORYSA:tr|A0A3Q9T3Z7|A0A3Q9T3Z7_ORYSA:tr|B8XEJ2|B8XEJ2_ORYSA:tr|B8XEK2|B8XEK2_ORYSA:tr|A0A076FRI5|A0A076FRI5_ORYSJ:Q42968|SSG1_ORYGL:A2Y8X2|SSG1_ORYSI:tr|B1B5Z0|B1B5Z0_ORYSI:tr|B1B5Z1|B1B5Z1_ORYSI:tr|C8CBL1|C8CBL1_ORYSJ:tr|A0A0D9WLF6|A0A0D9WLF6_9ORYZ:tr|A0A0E0A4K1|A0A0E0A4K1_9ORYZ
NLNNNPYFKG	42.87	1179.5673	10	590.7920	13.89	Granule-bound starch synthase I	tr|A0A0H4BM25|A0A0H4BM25_ORYSI:tr|A0A0E0DW79|A0A0E0DW79_9ORYZ:tr|A8QXE7|A8QXE7_ORYSI:tr|V5NEJ7|V5NEJ7_ORYSA:tr|A0A0E0DW80|A0A0E0DW80_9ORYZ:tr|A0EQH2|A0EQH2_ORYSJ:tr|A0EQE0|A0EQE0_ORYSA:tr|A0EQK7|A0EQK7_ORYRU:tr|A0EQD4|A0EQD4_ORYSI:tr|A0EQK5|A0EQK5_ORYRU:tr|A0EQK6|A0EQK6_ORYRU:tr|A0EQK4|A0EQK4_ORYRU:P0C585|SSG1_ORYSA:tr|B8XEJ8|B8XEJ8_ORYSA:tr|B8XEK3|B8XEK3_ORYSA:tr|D0TZY6|D0TZY6_ORYSI:tr|A0A3Q9T378|A0A3Q9T378_ORYSA:tr|D3U2H9|D3U2H9_ORYSA:tr|B8XEJ7|B8XEJ7_ORYSA:tr|A0A3Q9T3Z7|A0A3Q9T3Z7_ORYSA:tr|B8XEJ2|B8XEJ2_ORYSA:tr|B8XEK2|B8XEK2_ORYSA:tr|A0A076FRI5|A0A076FRI5_ORYSJ:Q42968|SSG1_ORYGL:A2Y8X2|SSG1_ORYSI:tr|B1B5Z0|B1B5Z0_ORYSI:tr|B1B5Z1|B1B5Z1_ORYSI:tr|C8CBL1|C8CBL1_ORYSJ:tr|A0A0D9WLF6|A0A0D9WLF6_9ORYZ:tr|A0A0E0A4K1|A0A0E0A4K1_9ORYZ
FGKAPGVPH	41.86	908.4868	9	455.2517	7.11	60S ribosomal protein l18	tr|A2Y0K0|A2Y0K0_ORYSI:tr|A0A0D9ZUF2|A0A0D9ZUF2_9ORYZ:tr|A0A0E0KYN0|A0A0E0KYN0_ORYPU:tr|A0A0D9WD00|A0A0D9WD00_9ORYZ:tr|A0A0E0DM93|A0A0E0DM93_9ORYZ:tr|A0A0E0H9T4|A0A0E0H9T4_ORYNI:tr|Q5WMY3|Q5WMY3_ORYSJ:tr|A0A0E0PHR1|A0A0E0PHR1_ORYRU:tr|A0A0D3G3E7|A0A0D3G3E7_9ORYZ:tr|J3M436|J3M436_ORYBR:tr|I1PSL1|I1PSL1_ORYGL
HGAFTPR	41.47	784.3980	7	393.2062	5.43	Glutelin	tr|B9F4T1|B9F4T1_ORYSJ:tr|I1NZ08|I1NZ08_ORYGL:tr|A1YQH4|A1YQH4_ORYSJ:tr|Q0E2D5|Q0E2D5_ORYSJ:Q02897|GLUB2_ORYSJ:tr|A1YQH5|A1YQH5_ORYSJ:tr|A0A0D9VE94|A0A0D9VE94_9ORYZ:tr|B9F4T2|B9F4T2_ORYSJ:tr|J3LB95|J3LB95_ORYBR:tr|A0A0E0NCF1|A0A0E0NCF1_ORYRU:tr|A0A0D3F334|A0A0D3F334_9ORYZ:tr|B8AEZ5|B8AEZ5_ORYSI:tr|B9F4T3|B9F4T3_ORYSJ:tr|T1T6C4|T1T6C4_ORYSI:tr|A1YQH6|A1Y

<table>
<tr><td></td><td></td><td></td><td></td><td></td><td></td><td></td><td>QH6_ORYSJ:tr|A0A0E0CIL1|A0A0E0CIL1_9ORYZ:tr|A0A0E0JY04|A0A0E0JY04_ORYPU:tr|A0A0E0NCF3|A0A0E0NCF3_ORYRU:tr|I1NZ10|I1NZ10_ORYGL:tr|Q0E2D2|Q0E2D2_ORYSJ:tr|T1T4F0|T1T4F0_ORYSI:tr|A0A0E0G6R1|A0A0E0G6R1_ORYNI:P14323|GLUB1_ORYSJ:tr|A0A0D9YPX0|A0A0D9YPX0_9ORYZ:tr|A0A0D3F336|A0A0D3F336_9ORYZ:tr|A0A0E0CIL2|A0A0E0CIL2_9ORYZ:tr|J3LBT5|J3LBT5_ORYBR:tr|A0A0D3F337|A0A0D3F337_9ORYZ:tr|A0A0E0G6R3|A0A0E0G6R3_ORYNI:tr|A2X301|A2X301_ORYSI</td></tr>
<tr><td>KGGIPIGIGK</td><td>39.99</td><td>938.5912</td><td>10</td><td>470.3026</td><td>12.12</td><td>Glucose-1-phosphate adenylyltransferase</td><td>tr|D4AIA3|D4AIA3_ORYSI:tr|B7EVB8|B7EVB8_ORYSJ:tr|A0A0E0LU24|A0A0E0LU24_ORYPU:tr|A0A0D3GZB2|A0A0D3GZB2_9ORYZ:tr|A0A0E0H7V9|A0A0E0H7V9_ORYNI:tr|A0A0E0QHR8|A0A0E0QHR8_ORYRU:tr|B8XEF2|B8XEF2_ORYSI:tr|B8XEE9|B8XEE9_ORYSI:tr|B8XED8|B8XED8_ORYSA:tr|B8XEE1|B8XEE1_ORYSI:tr|B8XED9|B8XED9_ORYSI:tr|B8XED7|B8XED7_ORYSI:tr|B8XEE8|B8XEE8_ORYSA:tr|B8XEE2|B8XEE2_ORYSJ:tr|B8XEF0|B8XEF0_ORYSI:tr|B8XEE5|B8XEE5_ORYSJ:tr|B8XEE6|B8XEE6_ORYSA:tr|A2YU91|A2YU91_ORYSI:tr|A0A0D3GZB1|A0A0D3GZB1_9ORYZ:tr|A0A0D9X6Z4|A0A0D9X6Z4_9ORYZ:tr|A0A0D9X6Z3|A0A0D9X6Z3_9ORYZ:tr|A0A0E0QHR7|A0A0E0QHR7_ORYRU:tr|A0A0E0H7V8|A0A0E0H7V8_ORYNI:tr|A0A0E0LU23|A0A0E0LU23_ORYPU</td></tr>
<tr><td>GYVGANPRL</td><td>39.81</td><td>945.5032</td><td>9</td><td>473.7594</td><td>12.69</td><td>Os04g0404400 protein</td><td>tr|Q0JDG9|Q0JDG9_ORYSJ:tr|A0A0P0WA63|A0A0P0WA63_ORYSJ:tr|A0A0D3FVG1|A0A0D3FVG1_9ORYZ:tr|A2XT28|A2XT28_ORYSI:tr|Q7X6I8|Q7X6I8_ORYSJ:tr|Q01L47|Q01L47_ORYSA:tr|I1PKX3|I1PKX3_ORYGL:tr|A0A0D9ZKB8|A0A0D9ZKB8_9ORYZ:tr|A0A0E0H0B3|A0A0E0H0B3_ORYNI:tr|A0A0E0P8P0|A0A0E0P8P0_ORYRU</td></tr>
<tr><td>APLYTAGPR</td><td>38.98</td><td>944.5079</td><td>9</td><td>473.2610</td><td>7.77</td><td>Uracil-DNA glycosylase</td><td>tr|A0A397LW25|A0A397LW25_9MICO</td></tr>
<tr><td>YVGANPRL</td><td>35.12</td><td>888.4817</td><td>8</td><td>445.2488</td><td>11.34</td><td>Os04g0404400 protein</td><td>tr|Q0JDG9|Q0JDG9_ORYSJ:tr|A0A0P0WA63|A0A0P0WA63_ORYSJ:tr|A0A0D3FVG1|A0A0D3FVG1_9ORYZ:tr|A2XT28|A2XT28_ORYSI:tr|Q7X6I8|Q7X6I8_ORYSJ:tr|Q01L47|Q01L47_ORYSA:tr|I1PKX3|I1PKX3_ORYGL:tr|A0A0D9ZKB8|A0A0D9ZKB8_9ORYZ:tr|A0A0E0H0B3|A0A0E0H0B3_ORYNI:tr|A0A0E0P8P0|A0A0E0P8P0_ORYRU</td></tr>
<tr><td>FNGVLRPG</td><td>33.39</td><td>858.4711</td><td>8</td><td>430.2441</td><td>12.65</td><td>Glutelin</td><td>tr|A2X399|A2X399_ORYSI:tr|A0A0E0JY90|A0A0E0JY90_ORYPU:P14614|GLUB4_ORYSJ:Q6ERU3|GLUB5_ORYSJ:tr|Q0E261|Q0E261_ORYSJ:tr|A0A0D9YQ79|A0A0D9YQ79_9ORYZ:tr|D6BV14|D6BV14_ORYSJ:tr|A0A0D3F3E6|A0A0D3F3E6_9ORYZ:tr|I1NZ94|I1NZ94_ORYGL:tr|A0A0D3F3E3|A0A0D3F3E3_9ORYZ:tr|A2X3A0|A2X3A0_ORYSI</td></tr>
<tr><td>SPFRVPIA</td><td>33.33</td><td>885.5072</td><td>8</td><td>443.7615</td><td>20.08</td><td>Pyruvate, phosphate dikinase</td><td>tr|J3M716|J3M716_ORYBR:tr|B9FPJ4|B9FPJ4_ORYSJ:tr|A0A0E0PMB6|A0A0E0PMB6_ORYRU:tr|B8AYC1|B8AYC1_ORYSI:tr|A0A0D9ZZ05|A0A0D9ZZ05_9ORYZ:Q6AVA8-2|PPDK1_ORYSJ:tr|A0A0E0DR93|A0A0E0DR93_9ORYZ:tr|A0A0D9VU47</td></tr>
</table>

							|A0A0D9VU47_9ORYZ:tr|J3LQ10|J3LQ10_ORYBR:tr|A0A0E0NWX2|A0A0E0NWX2_ORYRU:tr|O82032|O82032_ORYSI:tr|A0A0D3FK58|A0A0D3FK58_9ORYZ:tr|A0A0D9Z998|A0A0D9Z998_9ORYZ:tr|I1PCI9|I1PCI9_ORYGL:Q75KR1|PPDK2_ORYSJ:tr|A2XIA2|A2XIA2_ORYSI:tr|A0A0D3G7H5|A0A0D3G7H5_9ORYZ:tr|A0A0D9WGC7|A0A0D9WGC7_9ORYZ:tr|A0A0D9WGC6|A0A0D9WGC6_9ORYZ:Q6AVA8|PPDK1_ORYSJ:tr|I1PVJ3|I1PVJ3_ORYGL:tr|A0A0E0ITX7|A0A0E0ITX7_ORYNI:tr|A0A0E0ITX8|A0A0E0ITX8_ORYNI
APIYTQPR	32.25	944.5079	8	473.2610	7.77	Glucose-1-phosphate adenylyltransferase	tr|D4AIA3|D4AIA3_ORYSI:tr|B7EVB8|B7EVB8_ORYSJ:tr|A0A0E0LU24|A0A0E0LU24_ORYPU:tr|A0A0D3GZB2|A0A0D3GZB2_9ORYZ:tr|A0A0E0H7V9|A0A0E0H7V9_ORYNI:tr|A0A0E0QHR8|A0A0E0QHR8_ORYRU:tr|B8XEF2|B8XEF2_ORYSI:tr|B8XEE9|B8XEE9_ORYSI:tr|B8XED8|B8XED8_ORYSA:tr|B8XEE1|B8XEE1_ORYSI:tr|B8XED9|B8XED9_ORYSI:tr|B8XED7|B8XED7_ORYSI:tr|B8XEE8|B8XEE8_ORYSA:tr|B8XEE2|B8XEE2_ORYSJ:tr|B8XEF0|B8XEF0_ORYSI:tr|B8XEE5|B8XEE5_ORYSJ:tr|B8XEE6|B8XEE6_ORYSA:tr|A2YU91|A2YU91_ORYSI:tr|A0A0D3GZB1|A0A0D3GZB1_9ORYZ:tr|A0A0D9X6Z4|A0A0D9X6Z4_9ORYZ:tr|A0A0D9X6Z3|A0A0D9X6Z3_9ORYZ:tr|A0A0E0QHR7|A0A0E0QHR7_ORYRU:tr|A0A0E0H7V8|A0A0E0H7V8_ORYNI:tr|A0A0E0LU23|A0A0E0LU23_ORYPU
DWYKGPT	27.77	865.3970	7	433.7059	14.10	Elongation factor	tr|E7BJ60|E7BJ60_ORYSI:tr|A0A0E0NQG9|A0A0E0NQG9_ORYRU:tr|A0A0E0CW33|A0A0E0CW33_9ORYZ:tr|A0A0E0CW26|A0A0E0CW26_9ORYZ:tr|Q10QZ5|Q10QZ5_ORYSJ:tr|A0A0E0GHL8|A0A0E0GHL8_ORYNI:tr|A0A0N7KGP4|A0A0N7KGP4_ORYSJ:tr|B9FBM7|B9FBM7_ORYSJ:tr|A0A0D9VSS7|A0A0D9VSS7_9ORYZ:tr|A0A0D3FEJ0|A0A0D3FEJ0_9ORYZ:O64937|EF1A_ORYSJ:tr|A0A0E0GHL3|A0A0E0GHL3_ORYNI:tr|J3LKK4|J3LKK4_ORYBR:tr|A0A0E0K9J4|A0A0E0K9J4_ORYPU:tr|A0A1L2JKK1|A0A1L2JKK1_ORYSA:tr|Q10QZ6|Q10QZ6_ORYSJ:tr|A0A0D3FEJ3|A0A0D3FEJ3_9ORYZ:tr|B8APM5|B8APM5_ORYSI:tr|I1P851|I1P851_ORYGL:tr|A0A0E0K9J0|A0A0E0K9J0_ORYPU:tr|Q10QZ4|Q10QZ4_ORYSJ:tr|A0A0D3FEJ2|A0A0D3FEJ2_9ORYZ:tr|A0A0D3FEJ4|A0A0D3FEJ4_9ORYZ:tr|A0A0D3FEJ1|A0A0D3FEJ1_9ORYZ
NNPYFKGT	25.94	939.4450	8	470.7296	9.74	Granule-bound starch synthase I	tr|A0A0H4BM25|A0A0H4BM25_ORYSI:tr|A0A0E0DW79|A0A0E0DW79_9ORYZ:tr|A8QXE7|A8QXE7_ORYSI:tr|V5NEJ7|V5NEJ7_ORYSA:tr|A0A0E0DW80|A0A0E0DW80_9ORYZ:tr|A0EQH2|A0EQH2_ORYSJ:tr|A0EQE0|A0EQE0_ORYSA:tr|A0EQK7|A0EQK7_ORYRU:tr|A0EQD4|A0EQD4_ORYSI:tr|A0EQK5|A0EQK5_ORYRU:tr|A0EQK6|A0EQK6_ORYRU:tr|A0EQK4|A0EQK4_ORYRU:P0C585|SSG1_ORYSA:tr|B8XEJ8|B8XEJ8_ORYSA:tr|B8XEK3|B8XEK3_ORYSA:tr|D0TZY6|D0TZY6_ORYSI:tr|A0A3Q9T378|A0A3Q9T378_ORYSA:tr|D3U2H9|D3U2H9_ORYSA:tr|B8XEJ7|B8XEJ7_ORYSA:tr|A0A3Q9T3Z7|A0A3Q9T3Z7_ORYSA:tr|B8XEJ2|B8XEJ2_ORYSA:tr|B8XEK2|B8XEK2_ORYSA:tr|A0A076FRI5|A0A076FRI5_ORYSJ:Q42968|SSG1_ORYGL:A2Y8X2|SSG1_ORYSI:tr|B1B5Z0|B1B5Z0_ORYSI:tr|B1B5Z1|B1B5Z1_ORYSI:tr|C8CBL1|C8CBL1_ORYSJ:tr|A0A0D9WLF6|A0A0D9WLF6_9ORYZ:tr|A0A0E0A4K1|A0A0E0A4K1_9ORYZ

LEKVWGK	25.67	858.4963	7	430.2550	8.05	Granule-bound starch synthase I	tr|A0A0H4BM25|A0A0H4BM25_ORYSI:tr|A0A0E0DW79|A0A0E0DW79_9ORYZ:tr|A8QXE7|A8QXE7_ORYSI:tr|V5NEJ7|V5NEJ7_ORYSA:tr|A0A0E0DW80|A0A0E0DW80_9ORYZ:tr|A0EQH2|A0EQH2_ORYSJ:tr|A0EQE0|A0EQE0_ORYSA:tr|A0EQK7|A0EQK7_ORYRU:tr|A0EQD4|A0EQD4_ORYSI:tr|A0EQK5|A0EQK5_ORYRU:tr|A0EQK6|A0EQK6_ORYRU:tr|A0EQK4|A0EQK4_ORYRU:P0C585|SSG1_ORYSA:tr|B8XEJ8|B8XEJ8_ORYSA:tr|B8XEK3|B8XEK3_ORYSA:tr|D0TZY6|D0TZY6_ORYSI:tr|A0A3Q9T378|A0A3Q9T378_ORYSA:tr|D3U2H9|D3U2H9_ORYSA:tr|B8XEJ7|B8XEJ7_ORYSA:tr|A0A3Q9T3Z7|A0A3Q9T3Z7_ORYSA:tr|B8XEJ2|B8XEJ2_ORYSA:tr|B8XEK2|B8XEK2_ORYSA:tr|A0A076FRI5|A0A076FRI5_ORYSJ:Q42968|SSG1_ORYGL:A2Y8X2|SSG1_ORYSI:tr|B1B5Z0|B1B5Z0_ORYSI:tr|B1B5Z1|B1B5Z1_ORYSI:tr|C8CBL1|C8CBL1_ORYSJ:tr|A0A0D9WLF6|A0A0D9WLF6_9ORYZ:tr|A0A0E0A4K1|A0A0E0A4K1_9ORYZ
NNNPYFK	24.59	895.4188	7	448.7166	8.73	Granule-bound starch synthase I	tr|A0A0H4BM25|A0A0H4BM25_ORYSI:tr|A0A0E0DW79|A0A0E0DW79_9ORYZ:tr|A8QXE7|A8QXE7_ORYSI:tr|V5NEJ7|V5NEJ7_ORYSA:tr|A0A0E0DW80|A0A0E0DW80_9ORYZ:tr|A0EQH2|A0EQH2_ORYSJ:tr|A0EQE0|A0EQE0_ORYSA:tr|A0EQK7|A0EQK7_ORYRU:tr|A0EQD4|A0EQD4_ORYSI:tr|A0EQK5|A0EQK5_ORYRU:tr|A0EQK6|A0EQK6_ORYRU:tr|A0EQK4|A0EQK4_ORYRU:P0C585|SSG1_ORYSA:tr|B8XEJ8|B8XEJ8_ORYSA:tr|B8XEK3|B8XEK3_ORYSA:tr|D0TZY6|D0TZY6_ORYSI:tr|A0A3Q9T378|A0A3Q9T378_ORYSA:tr|D3U2H9|D3U2H9_ORYSA:tr|B8XEJ7|B8XEJ7_ORYSA:tr|A0A3Q9T3Z7|A0A3Q9T3Z7_ORYSA:tr|B8XEJ2|B8XEJ2_ORYSA:tr|B8XEK2|B8XEK2_ORYSA:tr|A0A076FRI5|A0A076FRI5_ORYSJ:Q42968|SSG1_ORYGL:A2Y8X2|SSG1_ORYSI:tr|B1B5Z0|B1B5Z0_ORYSI:tr|B1B5Z1|B1B5Z1_ORYSI:tr|C8CBL1|C8CBL1_ORYSJ:tr|A0A0D9WLF6|A0A0D9WLF6_9ORYZ:tr|A0A0E0A4K1|A0A0E0A4K1_9ORYZ
AFEPIRSVR	24.24	1073.5981	9	537.8064	11.34	Glutelin	tr|T1T4Y4|T1T4Y4_ORYSI:tr|J3L4C0|J3L4C0_ORYBR:tr|A0A0E0JP14|A0A0E0JP14_ORYPU:tr|A1YQG5|A1YQG5_ORYSJ:tr|I1NRU9|I1NRU9_ORYGL:tr|A1YQG3|A1YQG3_ORYSJ:tr|A2Z708|A2Z708_ORYSI:P07728|GLUA1_ORYSJ:tr|A0A0E0N2T5|A0A0E0N2T5_ORYRU:tr|I1QU95|I1QU95_ORYGL:P07730|GLUA2_ORYSJ:tr|A0A0E0M7E8|A0A0E0M7E8_ORYPU:tr|A0A0E0FTI2|A0A0E0FTI2_ORYNI:tr|A2WVB9|A2WVB9_ORYSI:tr|A0A0D9YFB1|A0A0D9YFB1_9ORYZ:tr|A0A0E0C821|A0A0E0C821_9ORYZ:tr|A0A0D3EUB5|A0A0D3EUB5_9ORYZ:tr|Q0JJ36|Q0JJ36_ORYSJ:tr|Q40689|Q40689_ORYSA:tr|A0A0E0QYR5|A0A0E0QYR5_ORYRU:tr|A0A0E0IRV1|A0A0E0IRV1_ORYNI:tr|A0A0E0EXG8|A0A0E0EXG8_9ORYZ:tr|A0A0E0QYR6|A0A0E0QYR6_ORYRU:tr|A0A0D3HDD5|A0A0D3HDD5_9ORYZ:tr|A0A0E0IRV2|A0A0E0IRV2_ORYNI:tr|T1T4G3|T1T4G3_ORYSI:tr|A0A0E0BA63|A0A0E0BA63_9ORYZ:tr|A0A0E0BA64|A0A0E0BA64_9ORYZ
HNPRQGGF	24.11	911.4362	8	456.7263	5.36	Glutelin	tr|A2X399|A2X399_ORYSI:tr|A0A0E0JY90|A0A0E0JY90_ORYPU:P14614|GLUB4_ORYSJ:Q6ERU3|GLUB5_ORYSJ:tr|Q0E261|Q0E261_ORYSJ:tr|A0A0D9YQ79|A0A0D9YQ79_9ORYZ:tr|D6BV14|D6BV14_ORYSJ:tr|A0A0D3F3E6|A0A0D3F3E6_9ORYZ:tr|I1NZ94|I1NZ94_ORYGL:tr|A0A0D3F3E3|A0A0D3F3E3_9ORYZ:tr|A2X3A0|A2X3A0_ORYSI

IGNPHLR	23.26	805.4559	7	403.7347	5.58	Putative mitochondrial energy transfer protein	tr|J3LAP3|J3LAP3_ORYBR:tr|A0A0D3F250|A0A0D3F250_9ORYZ:tr|A0A0E0JX14|A0A0E0JX14_ORYPU:tr|A2X256|A2X256_ORYSI:tr|A3A4A0|A3A4A0_ORYSJ:tr|A0A0E0G3F4|A0A0E0G3F4_ORYNI:tr|A0A0E0NY14|A0A0E0NY14_ORYRU:tr|A0A0D9YNX3|A0A0D9YNX3_9ORYZ:tr|A2YER7|A2YER7_ORYSI:tr|Q69XJ8|Q69XJ8_ORYSJ:tr|A0A0E0ABK1|A0A0E0ABK1_9ORYZ:tr|A0A0P0WYD6|A0A0P0WYD6_ORYSJ:tr|A0A0E0HT75|A0A0E0HT75_ORYNI:tr|J3MFK2|J3MFK2_ORYBR:tr|A0A0D9WRV1|A0A0D9WRV1_9ORYZ:tr|A0A0E0E3L8|A0A0E0E3L8_9ORYZ:tr|A0A0E0LD95|A0A0E0LD95_ORYPU:tr|I1Q3G0|I1Q3G0_ORYGL:tr|A0A0E0PZV6|A0A0E0PZV6_ORYRU:tr|A0A0D3GIF4|A0A0D3GIF4_9ORYZ:tr|Q6Z782|Q6Z782_ORYSJ:tr|I1NYC8|I1NYC8_ORYGL
HRDFFLA	21.86	904.4555	7	453.2358	16.33	Gt3	tr|A0A0E0D2D4|A0A0E0D2D4_9ORYZ:tr|A0A0E0NWT6|A0A0E0NWT6_ORYRU:tr|B7U2J6|B7U2J6_ORYSJ:tr|T1T5D8|T1T5D8_ORYSI:tr|J3M4W6|J3M4W6_ORYBR:tr|A0A0D3FK24|A0A0D3FK24_9ORYZ:tr|I1PCG0|I1PCG0_ORYGL:tr|Q10JA8|Q10JA8_ORYSJ:tr|A0A0E0KFA5|A0A0E0KFA5_ORYPU:Q09151|GLUA3_ORYSJ:tr|A0A0E0ITU1|A0A0E0ITU1_ORYNI:tr|A0A0D9Z962|A0A0D9Z962_9ORYZ:tr|B9F952|B9F952_ORYSJ:tr|B8AKE2|B8AKE2_ORYSI:tr|C7DQE9|C7DQE9_ORYSJ:tr|A0A0D9XVA4|A0A0D9XVA4_9ORYZ:tr|A0A0D9V5Z5|A0A0D9V5Z5_9ORYZ:tr|A0A0D9V5Z4|A0A0D9V5Z4_9ORYZ
WEDRFYK	20.44	1042.4872	7	522.2515	11.60	Acetolactate synthase	tr|B9F068|B9F068_ORYSJ:tr|E5FWX8|E5FWX8_ORYSI
KDKLWPM	20.34	916.4840	7	459.2496	16.79	H0613H07.5 protein	tr|A0A0D9VGF7|A0A0D9VGF7_9ORYZ:tr|Q01MK8|Q01MK8_ORYSA:tr|A0A0E0P4M4|A0A0E0P4M4_ORYRU:tr|A0A0E0DCG7|A0A0E0DCG7_9ORYZ:tr|A0A0D9ZGI8|A0A0D9ZGI8_9ORYZ:tr|A0A0E0GX73|A0A0E0GX73_ORYNI:tr|A2X5F3|A2X5F3_ORYSI:tr|Q7XTK1|Q7XTK1_ORYSJ:tr|Q6H4L2|Q6H4L2_ORYSJ:tr|A0A0E0KM97|A0A0E0KM97_ORYPU:tr|A0A0E0DA54|A0A0E0DA54_9ORYZ:tr|A0A0E0DA52|A0A0E0DA52_9ORYZ:tr|A0A0E0DA53|A0A0E0DA53_9ORYZ

Sub-fraction n. 16. Total: 24 identified peptides, all with rice protein accession. RT, retention time.

Peptide	-10lgP	Mass (Da)	Length (amino acid n.)	m/z	RT (min)	Protein	Accession
FGWDKDLAKK	51.68	1206.6396	10	604.3276	11.73	Os02g0519900 protein	tr|A0A0H4BM25|A0A0H4BM25_ORYSI:tr|A0A0E0DW79|A0A0E0DW79_9ORYZ:tr|A8QXE7|A8QXE7_ORYSI:tr|V5NEJ7|V5NEJ7_ORYSA:tr|A0A0E0DW80|A0A0E0DW80_9ORYZ:tr|A0EQH2|A0EQH2_ORYSJ:tr|A0EQE0|A0EQE0_ORYSA:tr|A0EQK7|A0EQK7_ORYRU:tr|A0EQD4|A0EQD4_ORYSI:tr|A0EQK5|A0EQK5_ORYRU:tr|A0EQK6|A0EQK6_ORYRU:tr|A0EQK4|A0EQK4_ORYRU:P0C585|SSG1_ORYSA:tr|B8XEJ8|B8XEJ8_ORYSA:tr|B8XEK3|B8XEK3_ORYSA:tr|D0TZY6|D0TZY6_ORYSI:tr|A0A3Q9T378|A0A

<table>
<tr><td></td><td></td><td></td><td></td><td></td><td></td><td></td><td>3Q9T378_ORYSA:tr|D3U2H9|D3U2H9_ORYSA:tr|B8XEJ7|B8XEJ7_ORYSA:tr|A0A3Q9T3Z7|A0A3Q9T3Z7_ORYSA:tr|B8XEJ2|B8XEJ2_ORYSA:tr|B8XEK2|B8XEK2_ORYSA:tr|A0A076FRI5|A0A076FRI5_ORYSJ:Q42968|SSG1_ORYGL:A2Y8X2|SSG1_ORYSI:tr|B1B5Z0|B1B5Z0_ORYSI:tr|B1B5Z1|B1B5Z1_ORYSI:tr|C8CBL1|C8CBL1_ORYSJ:tr|A0A0D9WLF6|A0A0D9WLF6_9ORYZ:tr|A0A0E0A4K1|A0A0E0A4K1_9ORYZ</td></tr>
<tr><td>GYVGANPRL</td><td>45.79</td><td>945.5032</td><td>9</td><td>473.7594</td><td>12.87</td><td>Os04g0404400 protein</td><td>tr|A0A0H4BM25|A0A0H4BM25_ORYSI:tr|A0A0E0DW79|A0A0E0DW79_9ORYZ:tr|A8QXE7|A8QXE7_ORYSI:tr|V5NEJ7|V5NEJ7_ORYSA:tr|A0A0E0DW80|A0A0E0DW80_9ORYZ:tr|A0EQH2|A0EQH2_ORYSJ:tr|A0EQE0|A0EQE0_ORYSA:tr|A0EQK7|A0EQK7_ORYRU:tr|A0EQD4|A0EQD4_ORYSI:tr|A0EQK5|A0EQK5_ORYRU:tr|A0EQK6|A0EQK6_ORYRU:tr|A0EQK4|A0EQK4_ORYRU:P0C585|SSG1_ORYSA:tr|B8XEJ8|B8XEJ8_ORYSA:tr|B8XEK3|B8XEK3_ORYSA:tr|D0TZY6|D0TZY6_ORYSI:tr|A0A3Q9T378|A0A3Q9T378_ORYSA:tr|D3U2H9|D3U2H9_ORYSA:tr|B8XEJ7|B8XEJ7_ORYSA:tr|A0A3Q9T3Z7|A0A3Q9T3Z7_ORYSA:tr|B8XEJ2|B8XEJ2_ORYSA:tr|B8XEK2|B8XEK2_ORYSA:tr|A0A076FRI5|A0A076FRI5_ORYSJ:Q42968|SSG1_ORYGL:A2Y8X2|SSG1_ORYSI:tr|B1B5Z0|B1B5Z0_ORYSI:tr|B1B5Z1|B1B5Z1_ORYSI:tr|C8CBL1|C8CBL1_ORYSJ:tr|A0A0D9WLF6|A0A0D9WLF6_9ORYZ:tr|A0A0E0A4K1|A0A0E0A4K1_9ORYZ</td></tr>
<tr><td>APIYTQPR</td><td>42.94</td><td>944.5079</td><td>8</td><td>473.2617</td><td>7.86</td><td>Glucose-1-phosphate adenylyltransferase</td><td>tr|A2Y0K0|A2Y0K0_ORYSI:tr|A0A0D9ZUF2|A0A0D9ZUF2_9ORYZ:tr|A0A0E0KYN0|A0A0E0KYN0_ORYPU:tr|A0A0D9WD00|A0A0D9WD00_9ORYZ:tr|A0A0E0DM93|A0A0E0DM93_9ORYZ:tr|A0A0E0H9T4|A0A0E0H9T4_ORYNI:tr|Q5WMY3|Q5WMY3_ORYSJ:tr|A0A0E0PHR1|A0A0E0PHR1_ORYRU:tr|A0A0D3G3E7|A0A0D3G3E7_9ORYZ:tr|J3M436|J3M436_ORYBR:tr|I1PSL1|I1PSL1_ORYGL</td></tr>
<tr><td>LDWYKGPT</td><td>42.26</td><td>978.4811</td><td>8</td><td>490.2484</td><td>17.93</td><td>Elongation factor</td><td>tr|B9F4T1|B9F4T1_ORYSJ:tr|I1NZ08|I1NZ08_ORYGL:tr|A1YQH4|A1YQH4_ORYSJ:tr|Q0E2D5|Q0E2D5_ORYSJ:Q02897|GLUB2_ORYSJ:tr|A1YQH5|A1YQH5_ORYSJ:tr|A0A0D9VE94|A0A0D9VE94_9ORYZ:tr|B9F4T2|B9F4T2_ORYSJ:tr|J3LB95|J3LB95_ORYBR:tr|A0A0E0NCF1|A0A0E0NCF1_ORYRU:tr|A0A0D3F334|A0A0D3F334_9ORYZ:tr|B8AEZ5|B8AEZ5_ORYSI:tr|B9F4T3|B9F4T3_ORYSJ:tr|T1T6C4|T1T6C4_ORYSI:tr|A1YQH6|A1YQH6_ORYSJ:tr|A0A0E0CIL1|A0A0E0CIL1_9ORYZ:tr|A0A0E0JY04|A0A0E0JY04_ORYPU:tr|A0A0E0NCF3|A0A0E0NCF3_ORYRU:tr|I1NZ10|I1NZ10_ORYGL:tr|Q0E2D2|Q0E2D2_ORYSJ:tr|T1T4F0|T1T4F0_ORYSI:tr|A0A0E0G6R1|A0A0E0G6R1_ORYNI:P14323|GLUB1_ORYSJ:tr|A0A0D9YPX0|A0A0D9YPX0_9ORYZ:tr|A0A0D3F336|A0A0D3F336_9ORYZ:tr|A0A0E0CIL2|A0A0E0CIL2_9ORYZ:tr|J3LBT5|J3LBT5_ORYBR:tr|A0A0D3F337|A0A0D3F337_9ORYZ:tr|A0A0E0G6R3|A0A0E0G6R3_ORYNI:tr|A2X301|A2X301_ORYSI</td></tr>
<tr><td>KGGIPIGIGK</td><td>40.26</td><td>938.5912</td><td>10</td><td>470.3035</td><td>12.31</td><td>Glucose-1-phosphate adenylyltransferase</td><td>tr|D4AIA3|D4AIA3_ORYSI:tr|B7EVB8|B7EVB8_ORYSJ:tr|A0A0E0LU24|A0A0E0LU24_ORYPU:tr|A0A0D3GZB2|A0A0D3GZB2_9ORYZ:tr|A0A0E0H7V9|A0A0E0H7V9_ORYNI:tr|A0A0E0QHR8|A0A0E0QHR8_ORYRU:tr|B8XEF2|B8XEF2_ORYSI:tr|B8XEE9|B8XEE9_ORYSI:tr|B8XED8|B8XED8_</td></tr>
</table>

							ORYSA:tr|B8XEE1|B8XEE1_ORYSI:tr|B8XED9|B8XED9_ORYSI:tr|B8XED7|B8XED7_ORYSI:tr|B8XEE8|B8XEE8_ORYSA:tr|B8XEE2|B8XEE2_ORYSJ:tr|B8XEF0|B8XEF0_ORYSI:tr|B8XEE5|B8XEE5_ORYSJ:tr|B8XEE6|B8XEE6_ORYSA:tr|A2YU91|A2YU91_ORYSI:tr|A0A0D3GZB1|A0A0D3GZB1_9ORYZ:tr|A0A0D9X6Z4|A0A0D9X6Z4_9ORYZ:tr|A0A0D9X6Z3|A0A0D9X6Z3_9ORYZ:tr|A0A0E0QHR7|A0A0E0QHR7_ORYRU:tr|A0A0E0H7V8|A0A0E0H7V8_ORYNI:tr|A0A0E0LU23|A0A0E0LU23_ORYPU
HGAFTPR	38.93	784.3980	7	393.2070	5.47	Glutelin	tr|Q0JDG9|Q0JDG9_ORYSJ:tr|A0A0P0WA63|A0A0P0WA63_ORYSJ:tr|A0A0D3FVG1|A0A0D3FVG1_9ORYZ:tr|A2XT28|A2XT28_ORYSI:tr|Q7X6I8|Q7X6I8_ORYSJ:tr|Q01L47|Q01L47_ORYSA:tr|I1PKX3|I1PKX3_ORYGL:tr|A0A0D9ZKB8|A0A0D9ZKB8_9ORYZ:tr|A0A0E0H0B3|A0A0E0H0B3_ORYNI:tr|A0A0E0P8P0|A0A0E0P8P0_ORYRU
NLNNNPYFK	35.93	1122.5458	9	562.2812	13.89	Granule-bound starch synthase I	tr|A0A397LW25|A0A397LW25_9MICO
KDKLWPM(+15.99)	30.44	932.4789	7	467.2475	16.96	Os02g0519900 protein	tr|Q0JDG9|Q0JDG9_ORYSJ:tr|A0A0P0WA63|A0A0P0WA63_ORYSJ:tr|A0A0D3FVG1|A0A0D3FVG1_9ORYZ:tr|A2XT28|A2XT28_ORYSI:tr|Q7X6I8|Q7X6I8_ORYSJ:tr|Q01L47|Q01L47_ORYSA:tr|I1PKX3|I1PKX3_ORYGL:tr|A0A0D9ZKB8|A0A0D9ZKB8_9ORYZ:tr|A0A0E0H0B3|A0A0E0H0B3_ORYNI:tr|A0A0E0P8P0|A0A0E0P8P0_ORYRU
H(+154.10)GAFTPR	30.09	938.4974	7	470.2562	16.99	Glutelin	tr|A2X399|A2X399_ORYSI:tr|A0A0E0JY90|A0A0E0JY90_ORYPU:P14614|GLUB4_ORYSJ:Q6ERU3|GLUB5_ORYSJ:tr|Q0E261|Q0E261_ORYSJ:tr|A0A0D9YQ79|A0A0D9YQ79_9ORYZ:tr|D6BV14|D6BV14_ORYSJ:tr|A0A0D3F3E6|A0A0D3F3E6_9ORYZ:tr|I1NZ94|I1NZ94_ORYGL:tr|A0A0D3F3E3|A0A0D3F3E3_9ORYZ:tr|A2X3A0|A2X3A0_ORYSI
IGNPHLR	29.31	805.4559	7	403.7355	5.68	Putative mitochondrial energy transfer protein	tr|J3M716|J3M716_ORYBR:tr|B9FPJ4|B9FPJ4_ORYSJ:tr|A0A0E0PMB6|A0A0E0PMB6_ORYRU:tr|B8AYC1|B8AYC1_ORYSI:tr|A0A0D9ZZ05|A0A0D9ZZ05_9ORYZ:Q6AVA8-2|PPDK1_ORYSJ:tr|A0A0E0DR93|A0A0E0DR93_9ORYZ:tr|A0A0D9VU47|A0A0D9VU47_9ORYZ:tr|J3LQ10|J3LQ10_ORYBR:tr|A0A0E0NWX2|A0A0E0NWX2_ORYRU:tr|O82032|O82032_ORYSI:tr|A0A0D3FK58|A0A0D3FK58_9ORYZ:tr|A0A0D9Z998|A0A0D9Z998_9ORYZ:tr|I1PCI9|I1PCI9_ORYGL:Q75KR1|PPDK2_ORYSJ:tr|A2XIA2|A2XIA2_ORYSI:tr|A0A0D3G7H5|A0A0D3G7H5_9ORYZ:tr|A0A0D9WGC7|A0A0D9WGC7_9ORYZ:tr|A0A0D9WGC6|A0A0D9WGC6_9ORYZ:Q6AVA8|PPDK1_ORYSJ:tr|I1PVJ3|I1PVJ3_ORYGL:tr|A0A0E0ITX7|A0A0E0ITX7_ORYNI:tr|A0A0E0ITX8|A0A0E0ITX8_ORYNI
WIDFPRAPQ	27.82	1128.5715	9	565.2937	21.83	Os02g0528200 protein	tr|D4AIA3|D4AIA3_ORYSI:tr|B7EVB8|B7EVB8_ORYSJ:tr|A0A0E0LU24|A0A0E0LU24_ORYPU:tr|A0A0D3GZB2|A0A0D3GZB2_9ORYZ:tr|A0A0E0H7V9|A0A0E0H7V9_ORYNI:tr|A0A0E0QHR8|A0A0E0QHR8_ORYRU:tr|B8XEF2|B8XEF2_ORYSI:tr|B8XEE9|B8XEE9_ORYSI:tr|B8XED8|B8XED8_

<table>
<tr><td></td><td></td><td></td><td></td><td></td><td></td><td></td><td>ORYSA:tr|B8XEE1|B8XEE1_ORYSI:tr|B8XED9|B8XED9_ORYSI:tr|B8XED7|B8XED7_ORYSI:tr|B8XEE8|B8XEE8_ORYSA:tr|B8XEE2|B8XEE2_ORYSJ:tr|B8XEF0|B8XEF0_ORYSI:tr|B8XEE5|B8XEE5_ORYSJ:tr|B8XEE6|B8XEE6_ORYSA:tr|A2YU91|A2YU91_ORYSI:tr|A0A0D3GZB1|A0A0D3GZB1_9ORYZ:tr|A0A0D9X6Z4|A0A0D9X6Z4_9ORYZ:tr|A0A0D9X6Z3|A0A0D9X6Z3_9ORYZ:tr|A0A0E0QHR7|A0A0E0QHR7_ORYRU:tr|A0A0E0H7V8|A0A0E0H7V8_ORYNI:tr|A0A0E0LU23|A0A0E0LU23_ORYPU</td></tr>
<tr><td>GDWFNKL</td><td>25.42</td><td>878.4286</td><td>7</td><td>440.2225</td><td>22.35</td><td>Pullulanase</td><td>tr|E7BJ60|E7BJ60_ORYSI:tr|A0A0E0NQG9|A0A0E0NQG9_ORYRU:tr|A0A0E0CW33|A0A0E0CW33_9ORYZ:tr|A0A0E0CW26|A0A0E0CW26_9ORYZ:tr|Q10QZ5|Q10QZ5_ORYSJ:tr|A0A0E0GHL8|A0A0E0GHL8_ORYNI:tr|A0A0N7KGP4|A0A0N7KGP4_ORYSJ:tr|B9FBM7|B9FBM7_ORYSJ:tr|A0A0D9VSS7|A0A0D9VSS7_9ORYZ:tr|A0A0D3FEJ0|A0A0D3FEJ0_9ORYZ:O64937|EF1A_ORYSJ:tr|A0A0E0GHL3|A0A0E0GHL3_ORYNI:tr|J3LKK4|J3LKK4_ORYBR:tr|A0A0E0K9J4|A0A0E0K9J4_ORYPU:tr|A0A1L2JKK1|A0A1L2JKK1_ORYSA:tr|Q10QZ6|Q10QZ6_ORYSJ:tr|A0A0D3FEJ3|A0A0D3FEJ3_9ORYZ:tr|B8APM5|B8APM5_ORYSI:tr|I1P851|I1P851_ORYGL:tr|A0A0E0K9J0|A0A0E0K9J0_ORYPU:tr|Q10QZ4|Q10QZ4_ORYSJ:tr|A0A0D3FEJ2|A0A0D3FEJ2_9ORYZ:tr|A0A0D3FEJ4|A0A0D3FEJ4_9ORYZ:tr|A0A0D3FEJ1|A0A0D3FEJ1_9ORYZ</td></tr>
<tr><td>GDWFNKI</td><td>25.42</td><td>878.4286</td><td>7</td><td>440.2225</td><td>22.35</td><td>Uncharacterized protein</td><td>tr|A0A0H4BM25|A0A0H4BM25_ORYSI:tr|A0A0E0DW79|A0A0E0DW79_9ORYZ:tr|A8QXE7|A8QXE7_ORYSI:tr|V5NEJ7|V5NEJ7_ORYSA:tr|A0A0E0DW80|A0A0E0DW80_9ORYZ:tr|A0EQH2|A0EQH2_ORYSJ:tr|A0EQE0|A0EQE0_ORYSA:tr|A0EQK7|A0EQK7_ORYRU:tr|A0EQD4|A0EQD4_ORYSI:tr|A0EQK5|A0EQK5_ORYRU:tr|A0EQK6|A0EQK6_ORYRU:tr|A0EQK4|A0EQK4_ORYRU:P0C585|SSG1_ORYSA:tr|B8XEJ8|B8XEJ8_ORYSA:tr|B8XEK3|B8XEK3_ORYSA:tr|D0TZY6|D0TZY6_ORYSI:tr|A0A3Q9T378|A0A3Q9T378_ORYSA:tr|D3U2H9|D3U2H9_ORYSA:tr|B8XEJ7|B8XEJ7_ORYSA:tr|A0A3Q9T3Z7|A0A3Q9T3Z7_ORYSA:tr|B8XEJ2|B8XEJ2_ORYSA:tr|B8XEK2|B8XEK2_ORYSA:tr|A0A076FRI5|A0A076FRI5_ORYSJ:Q42968|SSG1_ORYGL:A2Y8X2|SSG1_ORYSI:tr|B1B5Z0|B1B5Z0_ORYSI:tr|B1B5Z1|B1B5Z1_ORYSI:tr|C8CBL1|C8CBL1_ORYSJ:tr|A0A0D9WLF6|A0A0D9WLF6_9ORYZ:tr|A0A0E0A4K1|A0A0E0A4K1_9ORYZ</td></tr>
<tr><td>VVFGPLPKT</td><td>23.71</td><td>956.5695</td><td>9</td><td>479.2927</td><td>18.87</td><td>Os03g0305000 protein</td><td>tr|A0A0H4BM25|A0A0H4BM25_ORYSI:tr|A0A0E0DW79|A0A0E0DW79_9ORYZ:tr|A8QXE7|A8QXE7_ORYSI:tr|V5NEJ7|V5NEJ7_ORYSA:tr|A0A0E0DW80|A0A0E0DW80_9ORYZ:tr|A0EQH2|A0EQH2_ORYSJ:tr|A0EQE0|A0EQE0_ORYSA:tr|A0EQK7|A0EQK7_ORYRU:tr|A0EQD4|A0EQD4_ORYSI:tr|A0EQK5|A0EQK5_ORYRU:tr|A0EQK6|A0EQK6_ORYRU:tr|A0EQK4|A0EQK4_ORYRU:P0C585|SSG1_ORYSA:tr|B8XEJ8|B8XEJ8_ORYSA:tr|B8XEK3|B8XEK3_ORYSA:tr|D0TZY6|D0TZY6_ORYSI:tr|A0A3Q9T378|A0A3Q9T378_ORYSA:tr|D3U2H9|D3U2H9_ORYSA:tr|B8XEJ7|B8XEJ7_ORYSA:tr|A0A3Q9T3Z7|A0A3Q9T3Z7_ORYSA:tr|B8XEJ2|B8XEJ2_ORYSA:tr|B8XEK2|B8XEK2_ORYSA:tr|A0A076FRI5|A0A076FRI5_ORYSJ:Q42968|SSG1_ORYGL:A2Y8X2|SSG1_ORYSI:tr|B1B5Z0|B1B5Z0_ORYSI:tr|B1B5</td></tr>
</table>

							Z1|B1B5Z1_ORYSI:tr|C8CBL1|C8CBL1_ORYSJ:tr|A0A0D9WLF6|A0A0D9WLF6_9ORYZ:tr|A0A0E0A4K1|A0A0E0A4K1_9ORYZ
TGKSPYF	23.69	798.3912	7	400.2034	11.43	Globulin 2	tr|A0A0H4BM25|A0A0H4BM25_ORYSI:tr|A0A0E0DW79|A0A0E0DW79_9ORYZ:tr|A8QXE7|A8QXE7_ORYSI:tr|V5NEJ7|V5NEJ7_ORYSA:tr|A0A0E0DW80|A0A0E0DW80_9ORYZ:tr|A0EQH2|A0EQH2_ORYSJ:tr|A0EQE0|A0EQE0_ORYSA:tr|A0EQK7|A0EQK7_ORYRU:tr|A0EQD4|A0EQD4_ORYSI:tr|A0EQK5|A0EQK5_ORYRU:tr|A0EQK6|A0EQK6_ORYRU:tr|A0EQK4|A0EQK4_ORYRU:P0C585|SSG1_ORYSA:tr|B8XEJ8|B8XEJ8_ORYSA:tr|B8XEK3|B8XEK3_ORYSA:tr|D0TZY6|D0TZY6_ORYSI:tr|A0A3Q9T378|A0A3Q9T378_ORYSA:tr|D3U2H9|D3U2H9_ORYSA:tr|B8XEJ7|B8XEJ7_ORYSA:tr|A0A3Q9T3Z7|A0A3Q9T3Z7_ORYSA:tr|B8XEJ2|B8XEJ2_ORYSA:tr|B8XEK2|B8XEK2_ORYSA:tr|A0A076FRI5|A0A076FRI5_ORYSJ:Q42968|SSG1_ORYGL:A2Y8X2|SSG1_ORYSI:tr|B1B5Z0|B1B5Z0_ORYSI:tr|B1B5Z1|B1B5Z1_ORYSI:tr|C8CBL1|C8CBL1_ORYSJ:tr|A0A0D9WLF6|A0A0D9WLF6_9ORYZ:tr|A0A0E0A4K1|A0A0E0A4K1_9ORYZ
DWYKGPTL	23.48	978.4811	8	490.2484	22.19	Elongation factor	tr|T1T4Y4|T1T4Y4_ORYSI:tr|J3L4C0|J3L4C0_ORYBR:tr|A0A0E0JP14|A0A0E0JP14_ORYPU:tr|A1YQG5|A1YQG5_ORYSJ:tr|I1NRU9|I1NRU9_ORYGL:tr|A1YQG3|A1YQG3_ORYSJ:tr|A2Z708|A2Z708_ORYSI:P07728|GLUA1_ORYSJ:tr|A0A0E0N2T5|A0A0E0N2T5_ORYRU:tr|I1QU95|I1QU95_ORYGL:P07730|GLUA2_ORYSJ:tr|A0A0E0M7E8|A0A0E0M7E8_ORYPU:tr|A0A0E0FTI2|A0A0E0FTI2_ORYNI:tr|A2WVB9|A2WVB9_ORYSI:tr|A0A0D9YFB1|A0A0D9YFB1_9ORYZ:tr|A0A0E0C821|A0A0E0C821_9ORYZ:tr|A0A0D3EUB5|A0A0D3EUB5_9ORYZ:tr|Q0JJ36|Q0JJ36_ORYSJ:tr|Q40689|Q40689_ORYSA:tr|A0A0E0QYR5|A0A0E0QYR5_ORYRU:tr|A0A0E0IRV1|A0A0E0IRV1_ORYNI:tr|A0A0E0EXG8|A0A0E0EXG8_9ORYZ:tr|A0A0E0QYR6|A0A0E0QYR6_ORYRU:tr|A0A0D3HDD5|A0A0D3HDD5_9ORYZ:tr|A0A0E0IRV2|A0A0E0IRV2_ORYNI:tr|T1T4G3|T1T4G3_ORYSI:tr|A0A0E0BA63|A0A0E0BA63_9ORYZ:tr|A0A0E0BA64|A0A0E0BA64_9ORYZ
NPSTNPWHSPRQ	23.44	1419.6643	12	710.8401	7.20	Glutelin	tr|A2X399|A2X399_ORYSI:tr|A0A0E0JY90|A0A0E0JY90_ORYPU:P14614|GLUB4_ORYSJ:Q6ERU3|GLUB5_ORYSJ:tr|Q0E261|Q0E261_ORYSJ:tr|A0A0D9YQ79|A0A0D9YQ79_9ORYZ:tr|D6BV14|D6BV14_ORYSJ:tr|A0A0D3F3E6|A0A0D3F3E6_9ORYZ:tr|I1NZ94|I1NZ94_ORYGL:tr|A0A0D3F3E3|A0A0D3F3E3_9ORYZ:tr|A2X3A0|A2X3A0_ORYSI
NLNNNPYFKG	21.50	1179.5673	10	590.7913	14.04	Granule-bound starch synthase I	tr|J3LAP3|J3LAP3_ORYBR:tr|A0A0D3F250|A0A0D3F250_9ORYZ:tr|A0A0E0JX14|A0A0E0JX14_ORYPU:tr|A2X256|A2X256_ORYSI:tr|A3A4A0|A3A4A0_ORYSJ:tr|A0A0E0G3F4|A0A0E0G3F4_ORYNI:tr|A0A0E0NY14|A0A0E0NY14_ORYRU:tr|A0A0D9YNX3|A0A0D9YNX3_9ORYZ:tr|A2YER7|A2YER7_ORYSI:tr|Q69XJ8|Q69XJ8_ORYSJ:tr|A0A0E0ABK1|A0A0E0ABK1_9ORYZ:tr|A0A0P0WYD6|A0A0P0WYD6_ORYSJ:tr|A0A0E0HT75|A0A0E0HT75_ORYNI:tr|J3MFK2|J3MFK2_ORYBR:tr|A0A0D9WRV1|A0A0D9WRV1_9ORYZ:tr|A0A0E0E3L8|A0A0E0E3L8_9ORYZ:tr|A0A0E0LD95|A0A0E0LD95_ORYPU:tr|I1Q3G0|I1Q3G0_ORYGL:tr|A0A0E0PZV6|A0A0E0PZV6_ORYRU:tr|A0A0D3GIF4|A0A0D3GIF4_9ORYZ:tr|Q6Z782|Q6Z782_ORYSJ:tr|I1NYC8|I1NYC8_ORYGL

<table>
<tr><td>IPPPRGPLW</td><td>20.89</td><td>1031.5916</td><td>9</td><td>516.8027</td><td>20.95</td><td>Uncharacterized protein</td><td>tr|A0A0E0D2D4|A0A0E0D2D4_9ORYZ:tr|A0A0E0NWT6|A0A0E0NWT6_ORYRU:tr|B7U2J6|B7U2J6_ORYSJ:tr|T1T5D8|T1T5D8_ORYSI:tr|J3M4W6|J3M4W6_ORYBR:tr|A0A0D3FK24|A0A0D3FK24_9ORYZ:tr|I1PCG0|I1PCG0_ORYGL:tr|Q10JA8|Q10JA8_ORYSJ:tr|A0A0E0KFA5|A0A0E0KFA5_ORYPU:Q09151|GLUA3_ORYSJ:tr|A0A0E0ITU1|A0A0E0ITU1_ORYNI:tr|A0A0D9Z962|A0A0D9Z962_9ORYZ:tr|B9F952|B9F952_ORYSJ:tr|B8AKE2|B8AKE2_ORYSI:tr|C7DQE9|C7DQE9_ORYSJ:tr|A0A0D9XVA4|A0A0D9XVA4_9ORYZ:tr|A0A0D9V5Z5|A0A0D9V5Z5_9ORYZ:tr|A0A0D9V5Z4|A0A0D9V5Z4_9ORYZ</td></tr>
<tr><td>LDW(+15.99)YKGPT</td><td>20.83</td><td>994.4760</td><td>8</td><td>498.2450</td><td>17.95</td><td>Elongation factor</td><td>tr|B9F068|B9F068_ORYSJ:tr|E5FWX8|E5FWX8_ORYSI</td></tr>
<tr><td>KDKLWPM</td><td>20.82</td><td>916.4840</td><td>7</td><td>459.2496</td><td>16.86</td><td>Os02g0519900 protein</td><td>tr|A0A0D9VGF7|A0A0D9VGF7_9ORYZ:tr|Q01MK8|Q01MK8_ORYSA:tr|A0A0E0P4M4|A0A0E0P4M4_ORYRU:tr|A0A0E0DCG7|A0A0E0DCG7_9ORYZ:tr|A0A0D9ZGI8|A0A0D9ZGI8_9ORYZ:tr|A0A0E0GX73|A0A0E0GX73_ORYNI:tr|A2X5F3|A2X5F3_ORYSI:tr|Q7XTK1|Q7XTK1_ORYSJ:tr|Q6H4L2|Q6H4L2_ORYSJ:tr|A0A0E0KM97|A0A0E0KM97_ORYPU:tr|A0A0E0DA54|A0A0E0DA54_9ORYZ:tr|A0A0E0DA52|A0A0E0DA52_9ORYZ:tr|A0A0E0DA53|A0A0E0DA53_9ORYZ</td></tr>
<tr><td>GKGYVGL</td><td>20.59</td><td>692.3857</td><td>7</td><td>347.2007</td><td>12.12</td><td>Glutelin</td><td>tr|A0A0E0NCE6|A0A0E0NCE6_ORYRU:tr|T1T4G5|T1T4G5_ORYSI:tr|M1G949|M1G949_ORYSI:Q6K508|GLUD1_ORYSJ:tr|I1NZ02|I1NZ02_ORYGL:tr|A2X2Z1|A2X2Z1_ORYSI:tr|A0A0D3F331|A0A0D3F331_9ORYZ:tr|A0A0E0FMV5|A0A0E0FMV5_ORYNI:tr|A0A0E0NCE4|A0A0E0NCE4_ORYRU:tr|A0A0E0JXZ6|A0A0E0JXZ6_ORYPU:tr|A0A0D9YPW1|A0A0D9YPW1_9ORYZ</td></tr>
<tr><td>ARGGGGGGGGGVAPPG</td><td>20.26</td><td>1179.5745</td><td>16</td><td>590.7913</td><td>14.04</td><td>Uncharacterized protein</td><td>tr|A0A0E0PQU4|A0A0E0PQU4_ORYRU</td></tr>
<tr><td>GTDGRMHW</td><td>20.06</td><td>958.4080</td><td>8</td><td>480.2126</td><td>9.11</td><td>Alpha-1,4 glucan phosphorylase</td><td>tr|A0A0E0D717|A0A0E0D717_9ORYZ</td></tr>
</table>

Sub-fraction n. 18. Total: 29 identified peptides, 21 peptides with rice protein accession. RT, retention time.

<table>
<tr><th>Peptide</th><th>-10lgP</th><th>Mass (Da)</th><th>Length (amino acid n.)</th><th>m/z</th><th>RT (min)</th><th>Protein</th><th>Accession</th></tr>
<tr><td>LDWYKGPT</td><td>48.22</td><td>978.4811</td><td>8</td><td>490.2474</td><td>17.88</td><td>Elongation factor</td><td>tr|E7BJ60|E7BJ60_ORYSI:tr|A0A0E0NQG9|A0A0E0NQG9_ORYRU:tr|A0A0P0VTT8|A0A0P0VTT8_ORYSJ:tr|A0A0E0CW33|A0A0E0CW33_9ORYZ:</td></tr>
</table>

<table>
<tr><td></td><td></td><td></td><td></td><td></td><td></td><td></td><td>tr|A0A0E0CW26|A0A0E0CW26_9ORYZ:tr|Q10QZ5|Q10QZ5_ORYSJ:tr|A0A0E0GHL8|A0A0E0GHL8_ORYNI:tr|A0A0N7KGP4|A0A0N7KGP4_ORYSJ:tr|I1P848|I1P848_ORYGL:tr|B9FBM7|B9FBM7_ORYSJ:tr|J3LKK4|J3LKK4_ORYBR:tr|A0A0E0K9J4|A0A0E0K9J4_ORYPU:tr|A0A1L2JKK1|A0A1L2JKK1_ORYSA:tr|Q10QZ6|Q10QZ6_ORYSJ:tr|A0A0D3FEJ3|A0A0D3FEJ3_9ORYZ:tr|A0A0D9VSS7|A0A0D9VSS7_9ORYZ:tr|A0A0D3FEJ0|A0A0D3FEJ0_9ORYZ:O64937|EF1A_ORYSJ:tr|A0A0E0GHL3|A0A0E0GHL3_ORYNI:tr|J3L9Z7|J3L9Z7_ORYBR:tr|B8APM5|B8APM5_ORYSI:tr|I1P851|I1P851_ORYGL:tr|A0A0E0K9J0|A0A0E0K9J0_ORYPU:tr|Q10QZ4|Q10QZ4_ORYSJ:tr|A0A0D3FEJ2|A0A0D3FEJ2_9ORYZ:tr|I1P850|I1P850_ORYGL:tr|A0A0D3FEJ4|A0A0D3FEJ4_9ORYZ:tr|A0A0D9Z2R9|A0A0D9Z2R9_9ORYZ:tr|A0A0D3FEJ1|A0A0D3FEJ1_9ORYZ</td></tr>
<tr><td>VVVGTPGRVF</td><td>48.10</td><td>1029.5970</td><td>10</td><td>515.8058</td><td>17.99</td><td>Eukaryotic initiation factor 4A-1</td><td>P41376|IF4A1_ARATH</td></tr>
<tr><td>VFNGVLRPG</td><td>47.26</td><td>957.5396</td><td>9</td><td>479.7779</td><td>15.84</td><td>Glutelin</td><td>tr|A2X3A0|A2X3A0_ORYSI:tr|A2X399|A2X399_ORYSI:P14614|GLUB4_ORYSJ:tr|Q0E261|Q0E261_ORYSJ:tr|A0A0D9YQ79|A0A0D9YQ79_9ORYZ:tr|D6BV14|D6BV14_ORYSJ:tr|A0A0D3F3E6|A0A0D3F3E6_9ORYZ:tr|A0A0E0JY90|A0A0E0JY90_ORYPU:Q6ERU3|GLUB5_ORYSJ:tr|I1NZ94|I1NZ94_ORYGL:tr|A0A0D3F3E3|A0A0D3F3E3_9ORYZ:tr|A0A0E0JY91|A0A0E0JY91_ORYPU:tr|A0A0D3F3E5|A0A0D3F3E5_9ORYZ:tr|C0L8H2|C0L8H2_ORYSJ:tr|A3A5D6|A3A5D6_ORYSJ:tr|A0A0E0G4Q4|A0A0E0G4Q4_ORYNI:tr|A0A0E0NCT0|A0A0E0NCT0_ORYRU:tr|M1G571|M1G571_ORYSJ:tr|M1G2E3|M1G2E3_ORYSI:tr|A0A0D9VG85|A0A0D9VG85_9ORYZ:tr|Q0E2G5|Q0E2G5_ORYSJ:tr|J3LBL3|J3LBL3_ORYBR:tr|C0L8H1|C0L8H1_ORYSJ:tr|A0A0D9YLS5|A0A0D9YLS5_9ORYZ:tr|Q84X94|Q84X94_ORYSJ:tr|A0A0E0NCA6|A0A0E0NCA6_ORYRU:tr|Q84X93|Q84X93_ORYSJ:tr|A0A0E0G459|A0A0E0G459_ORYNI:tr|Q6ESW6|Q6ESW6_ORYSJ:tr|A2X2V1|A2X2V1_ORYSI</td></tr>
<tr><td>SSKPFFGGL</td><td>44.30</td><td>938.4861</td><td>9</td><td>470.2505</td><td>21.93</td><td>Nucleoside diphosphate kinase</td><td>tr|A0A0E0IWK3|A0A0E0IWK3_ORYNI:tr|A2ZAA7|A2ZAA7_ORYSI:tr|A0A0E0R2J8|A0A0E0R2J8_ORYRU:tr|Q7XC37|Q7XC37_ORYSJ:tr|I1QW04|I1QW04_ORYGL:tr|A0A0D9XMI7|A0A0D9XMI7_9ORYZ:tr|A0A0E0EGM8|A0A0E0EGM8_9ORYZ:tr|A0A0E0BDU7|A0A0E0BDU7_9ORYZ:tr|A0A0D3HGP0|A0A0D3HGP0_9ORYZ:tr|A0A0E0MAU0|A0A0E0MAU0_ORYPU:tr|J3N4Y6|J3N4Y6_ORYBR:tr|A6N077|A6N077_ORYSI</td></tr>
<tr><td>KKPVPDFSFY</td><td>43.99</td><td>1226.6335</td><td>10</td><td>614.3234</td><td>19.18</td><td>ADP-glucose pyrophosphorylase small subunit</td><td>tr|A7IZE4|A7IZE4_ORYSI:tr|D4AIA3|D4AIA3_ORYSI:tr|B7EVB8|B7EVB8_ORYSJ:tr|A0A0D3GZB2|A0A0D3GZB2_9ORYZ:tr|A0A0E0QHR8|A0A0E0QHR8_ORYRU:tr|B8XED8|B8XED8_ORYSA:tr|B8XED9|B8XED9_ORYSI:tr|B8XED7|B8XED7_ORYSI:tr|B8XEE8|B8XEE8_ORYSA:tr|B8XEF0|B8XEF0_ORYSI:tr|B8XEF2|B8XEF2_ORYSI:tr|B8XEE9|B8XEE9_ORYSI:tr|B8XEE1|B8XEE1_ORYSI:tr|B8XEE2|B8XEE2_ORYSJ:tr|Q9ARH9|Q9ARH9_ORYSA:tr|D3U2H7|D3U2H7_ORYSA:tr|D0TZC9|D0TZC9_ORYSI:Q69T9</td></tr>
</table>

							9|GLGS1_ORYSJ:tr|B8XEE5|B8XEE5_ORYSJ:tr|B8XEE6|B8XEE6_ORYSA:tr|D0TZC6|D0TZC6_ORYSJ:tr|A0A0E0EQI0|A0A0E0EQI0_9ORYZ:tr|B8BE16|B8BE16_ORYSI:tr|A2YU91|A2YU91_ORYSI:tr|A0A0D3GZB1|A0A0D3GZB1_9ORYZ:tr|A0A0E0B114|A0A0E0B114_9ORYZ:tr|A0A0D3H4W0|A0A0D3H4W0_9ORYZ:tr|A0A0E0LZH2|A0A0E0LZH2_ORYPU:tr|A0A0E0QHR7|A0A0E0QHR7_ORYRU
TPIQYKSY	43.91	998.5073	8	500.2612	8.83	Glutelin	tr|A0A0E0M7E9|A0A0E0M7E9_ORYPU:tr|T1T4G3|T1T4G3_ORYSI:tr|A0A0E0JP14|A0A0E0JP14_ORYPU:tr|A1YQG5|A1YQG5_ORYSJ:tr|I1NRU9|I1NRU9_ORYGL:tr|A0A0E0N2T5|A0A0E0N2T5_ORYRU:tr|A2WVB9|A2WVB9_ORYSI:P07728|GLUA1_ORYSJ:tr|A0A0E0M7E8|A0A0E0M7E8_ORYPU:tr|A0A0E0FTI2|A0A0E0FTI2_ORYNI:tr|A0A0D9YFB1|A0A0D9YFB1_9ORYZ:tr|A0A0E0C821|A0A0E0C821_9ORYZ:tr|A0A0D3EUB5|A0A0D3EUB5_9ORYZ:tr|Q0JJ36|Q0JJ36_ORYSJ:tr|Q40689|Q40689_ORYSA
TPLQYKSY	43.91	998.5073	8	500.2612	8.83	Glutelin	tr|T1T4Y4|T1T4Y4_ORYSI:tr|A1YQG3|A1YQG3_ORYSJ:P07730|GLUA2_ORYSJ:tr|A2Z708|A2Z708_ORYSI:tr|A0A0E0BA65|A0A0E0BA65_9ORYZ:tr|A0A0E0IRV3|A0A0E0IRV3_ORYNI:tr|A0A0D3HDD6|A0A0D3HDD6_9ORYZ:tr|A0A0E0QYR7|A0A0E0QYR7_ORYRU:tr|A0A0E0EXG9|A0A0E0EXG9_9ORYZ:tr|A0A0E0BA63|A0A0E0BA63_9ORYZ:tr|A0A0E0BA64|A0A0E0BA64_9ORYZ:tr|A0A0E0IRV1|A0A0E0IRV1_ORYNI:tr|A0A0E0QYR5|A0A0E0QYR5_ORYRU:tr|A0A0E0EXG8|A0A0E0EXG8_9ORYZ:tr|A0A0E0QYR6|A0A0E0QYR6_ORYRU:tr|A0A0D3HDD5|A0A0D3HDD5_9ORYZ:tr|A0A0E0IRV2|A0A0E0IRV2_ORYNI
VFDGVLRPG	40.15	958.5236	9	480.2692	16.56	Uncharacterized protein	tr|I1NZ10|I1NZ10_ORYGL:tr|A2X2Z8|A2X2Z8_ORYSI
WIDFPRAPQ	37.36	1128.5715	9	565.2944	21.76	Branching enzyme-3	tr|A0A0D3F5W2|A0A0D3F5W2_9ORYZ:tr|A0A0E0K0K5|A0A0E0K0K5_ORYPU:tr|A0A0E0G7M7|A0A0E0G7M7_ORYNI:tr|A0A0E0NFS6|A0A0E0NFS6_ORYRU:tr|A0A0D9YT34|A0A0D9YT34_9ORYZ:tr|A0A0E0K0K4|A0A0E0K0K4_ORYPU:tr|A0A0E0CLF1|A0A0E0CLF1_9ORYZ:tr|Q6H6P8|Q6H6P8_ORYSJ:tr|Q40663|Q40663_ORYSA:tr|A2X5K0|A2X5K0_ORYSI:tr|D0TZK1|D0TZK1_ORYSI:tr|I1P0X2|I1P0X2_ORYGL:tr|B3VDJ4|B3VDJ4_ORYSJ:tr|I6VRB8|I6VRB8_ORYSJ:tr|A0A0D9VGL1|A0A0D9VGL1_9ORYZ:tr|A0A0N7KFE7|A0A0N7KFE7_ORYSJ
HGAFTPR	37.03	784.3980	7	393.2070	5.44	Uncharacterized protein	tr|I1NZ10|I1NZ10_ORYGL
HS(-18.01)AFTPR	35.92	796.3980	7	399.2061	5.61	Uncharacterized protein	tr|A2X2Z8|A2X2Z8_ORYSI

IGRPAPMPY	35.69	1000.5164	9	501.2663	14.18	Os06g0676700 protein	tr|A0A0E0Q1L2|A0A0E0Q1L2_ORYRU:tr|A0A0E0LEN1|A0A0E0LEN1_ORYPU:tr|A0A0D3GJV3|A0A0D3GJV3_9ORYZ:tr|A0A0E0GD25|A0A0E0GD25_ORYNI:tr|A0A0E0E5A4|A0A0E0E5A4_9ORYZ:tr|A0A0E0Q1K4|A0A0E0Q1K4_ORYRU:tr|A0A0E0Q1K2|A0A0E0Q1K2_ORYRU:tr|A0A0E0AD77|A0A0E0AD77_9ORYZ:tr|A3BEL8|A3BEL8_ORYSJ:tr|A0A0E0Q1K3|A0A0E0Q1K3_ORYRU:tr|A0A0E0AD76|A0A0E0AD76_9ORYZ:tr|A0A0D3GJV2|A0A0D3GJV2_9ORYZ:Q653V7|AGLU_ORYSJ:tr|Q653V4|Q653V4_ORYSJ:tr|A2YG59|A2YG59_ORYSI:tr|A0A0E0AD89|A0A0E0AD89_9ORYZ:tr|B8B1F4|B8B1F4_ORYSI:tr|B8AD31|B8AD31_ORYSI:tr|Q9LGC6|Q9LGC6_ORYSJ:tr|A0A0D9UWE3|A0A0D9UWE3_9ORYZ:tr|A0A0E0BWI1|A0A0E0BWI1_9ORYZ:tr|Q0JQZ2|Q0JQZ2_ORYSJ:tr|A0A0E0MQY3|A0A0E0MQY3_ORYRU:tr|A0A0E0FFR9|A0A0E0FFR9_ORYNI:tr|A0A0D3EJ82|A0A0D3EJ82_9ORYZ:tr|A0A0E0JDN4|A0A0E0JDN4_ORYPU:tr|A0A0E0LEM2|A0A0E0LEM2_ORYPU:tr|A0A0E0AD80|A0A0E0AD80_9ORYZ:tr|A0A0E0GD31|A0A0E0GD31_ORYNI:tr|A0A0E0E598|A0A0E0E598_9ORYZ
NLNNNPYFK	34.84	1122.5458	9	562.2811	13.78	Granule-bound starch synthase I	tr|A0A0H4BM25|A0A0H4BM25_ORYSI:tr|A0A0E0DW79|A0A0E0DW79_9ORYZ:tr|A8QXE7|A8QXE7_ORYSI:tr|V5NEJ7|V5NEJ7_ORYSA:tr|A0A0E0DW80|A0A0E0DW80_9ORYZ:tr|A0EQH2|A0EQH2_ORYSJ:tr|A0EQK7|A0EQK7_ORYRU:tr|A0EQD4|A0EQD4_ORYSI:tr|A0EQK4|A0EQK4_ORYRU:tr|A0EQE0|A0EQE0_ORYSA:tr|A0EQK5|A0EQK5_ORYRU:tr|A0EQK6|A0EQK6_ORYRU:P0C585|SSG1_ORYSA:tr|B8XEK3|B8XEK3_ORYSA:tr|D0TZY6|D0TZY6_ORYSI:tr|A0A3Q9T378|A0A3Q9T378_ORYSA:tr|D3U2H9|D3U2H9_ORYSA:tr|B8XEJ7|B8XEJ7_ORYSA:tr|A0A3Q9T3Z7|A0A3Q9T3Z7_ORYSA:tr|B8XEJ2|B8XEJ2_ORYSA:tr|B8XEK2|B8XEK2_ORYSA:tr|A0A076FRI5|A0A076FRI5_ORYSJ:Q42968|SSG1_ORYGL:A2Y8X2|SSG1_ORYSI:tr|C8CBL1|C8CBL1_ORYSJ:tr|B8XEJ8|B8XEJ8_ORYSA:tr|B8XEI6|B8XEI6_ORYSI:tr|B1B5Z0|B1B5Z0_ORYSI:tr|B1B5Z1|B1B5Z1_ORYSI:tr|A0A0D9WLF6|A0A0D9WLF6_9ORYZ:tr|A0A0E0A4K1|A0A0E0A4K1_9ORYZ
STNPWHSPRQG	33.72	1265.5901	11	633.8027	6.00	Uncharacterized protein	tr|I1NZ10|I1NZ10_ORYGL:tr|A2X2Z8|A2X2Z8_ORYSI
H(+154.10)GAFTPR	33.27	938.4974	7	470.2571	16.91	Uncharacterized protein	tr|I1NZ10|I1NZ10_ORYGL
TNPWHSPRQGS	30.72	1265.5901	11	633.8027	6.00	Uncharacterized protein	tr|I1NZ10|I1NZ10_ORYGL:tr|A2X2Z8|A2X2Z8_ORYSI
KIVDFIKF	29.10	1008.6008	8	505.3086	23.28	40S ribosomal protein S4	tr|A0A0E0N8M2|A0A0E0N8M2_ORYRU:tr|A0A0E0DQN5|A0A0E0DQN5_9ORYZ:tr|A0A0E0L1P5|A0A0E0L1P5_ORYPU:tr|Q6L588|Q6L588_ORYSJ:tr|A0A0E0N8M1|A0A0E0N8M1_ORYRU:tr|A0A0E0JIC6|A0A0E0JIC6_ORYPU:tr|A0A0D3G6S5|A0A0D3G6S5_9ORYZ:tr|A0A0E0L1P4|A0A0E0L1P

							4_ORYPU:tr|A0A0E0HDG9|A0A0E0HDG9_ORYNI:tr|Q0DIR7|Q0DIR7_ORYSJ:tr|A0A0E0DQN3|A0A0E0DQN3_9ORYZ:tr|A0A0E0PLJ2|A0A0E0PLJ2_ORYRU:tr|A2WZV1|A2WZV1_ORYSI:tr|A0A0E0FZW8|A0A0E0FZW8_ORYNI:tr|Q0E4Q0|Q0E4Q0_ORYSJ:tr|A0A0E0C1U8|A0A0E0C1U8_9ORYZ:tr|A0A0D3ENQ1|A0A0D3ENQ1_9ORYZ:tr|A2WPV4|A2WPV4_ORYSI:tr|A0A0E0FZX5|A0A0E0FZX5_ORYNI:tr|I1NN05|I1NN05_ORYGL:tr|A0A0E0MW63|A0A0E0MW63_ORYRU:P49398|RS4_ORYSJ:tr|Q5ZBX1|Q5ZBX1_ORYSJ:tr|A0A0D3EZK6|A0A0D3EZK6_9ORYZ:tr|A0A0E0FLJ5|A0A0E0FLJ5_ORYNI:tr|A0A0D9YL39|A0A0D9YL39_9ORYZ:tr|A0A0D9V0T6|A0A0D9V0T6_9ORYZ:tr|A0A0E0JUG2|A0A0E0JUG2_ORYPU:tr|B9FHI2|B9FHI2_ORYSJ:tr|A0A0D3EZK3|A0A0D3EZK3_9ORYZ
IPPPRGPLW	28.80	1031.5916	9	516.8038	20.92	Uncharacterized protein	tr|A0A0E0PH77|A0A0E0PH77_ORYRU:P42211|ASPRX_ORYSJ:tr|A0A0D3GAX9|A0A0D3GAX9_9ORYZ:Q42456|ASPR1_ORYSJ
PASVAHW	28.01	766.3762	7	384.1961	11.46	Uncharacterized protein	tr|I1NZ10|I1NZ10_ORYGL
VVGTPGRVF	27.73	930.5287	9	466.2719	15.08	P41376|IF4A1_ARATH	P41376|IF4A1_ARATH
IDFPRAPQ	27.42	942.4922	8	472.2540	15.84	Branching enzyme-3	tr|A0A0D3F5W2|A0A0D3F5W2_9ORYZ:tr|A0A0E0K0K5|A0A0E0K0K5_ORYPU:tr|A0A0E0G7M7|A0A0E0G7M7_ORYNI:tr|A0A0E0NFS6|A0A0E0NFS6_ORYRU:tr|A0A0D9YT34|A0A0D9YT34_9ORYZ:tr|A0A0E0K0K4|A0A0E0K0K4_ORYPU:tr|A0A0E0CLF1|A0A0E0CLF1_9ORYZ:tr|Q6H6P8|Q6H6P8_ORYSJ:tr|Q40663|Q40663_ORYSA:tr|A2X5K0|A2X5K0_ORYSI:tr|D0TZK1|D0TZK1_ORYSI:tr|I1P0X2|I1P0X2_ORYGL:tr|B3VDJ4|B3VDJ4_ORYSJ:tr|I6VRB8|I6VRB8_ORYSJ:tr|A0A0D9VGL1|A0A0D9VGL1_9ORYZ:tr|A0A0N7KFE7|A0A0N7KFE7_ORYSJ

Sub-fraction n. 19. Total: 24 identified peptides, all with rice protein accession. RT, retention time.

Peptide	-10lgP	Mass (Da)	Length (amino acid n.)	m/z	RT (min)	Protein	Accession
KKPVPDFSFY	53.64	1226.6335	10	614.3241	19.51	Glucose-1-phosphate adenylyltransferase	tr|D4AIA3|D4AIA3_ORYSI:tr|B7EVB8|B7EVB8_ORYSJ:tr|A0A0D3GZB2|A0A0D3GZB2_9ORYZ:tr|A0A0E0H7V9|A0A0E0H7V9_ORYNI:tr|A0A0E0QHR8|A0A0E0QHR8_ORYRU:tr|B8XED8|B8XED8_ORYSA:tr|B8XED9|B8XED9_ORYSI:tr|B8XED7|B8XED7_ORYSI:tr|B8XEE8|B8XEE8_ORYSA:tr|B8XEF0|B8XEF0_ORYSI:tr|B8XEF2|B8XEF2_ORYSI:tr|B8XEE1|B8XEE1_ORYSI:tr|B8XEE2|B8XEE2_ORYSJ:tr|Q9ARH9|Q9ARH9_ORYSA:tr|D3U2

Peptide	-10lgP	Mass (Da)	Length (amino acid n.)	m/z	RT (min)	Protein	Accession
							H7|D3U2H7_ORYSA:tr|D0TZC9|D0TZC9_ORYSI:Q69T99|GLGS1_ORYSJ:tr|B8XEE5|B8XEE5_ORYSJ:tr|B8XEE6|B8XEE6_ORYSA:tr|D0TZC6|D0TZC6_ORYSJ:tr|A0A0E0EQI0|A0A0E0EQI0_9ORYZ:tr|B8BE16|B8BE16_ORYSI:tr|A2YU91|A2YU91_ORYSI:tr|A0A0D3GZB1|A0A0D3GZB1_9ORYZ:tr|A0A0E0B114|A0A0E0B114_9ORYZ:tr|A0A0D3H4W0|A0A0D3H4W0_9ORYZ:tr|A0A0E0LZH2|A0A0E0LZH2_ORYPU:tr|A0A0E0QHR7|A0A0E0QHR7_ORYRU:tr|A7IZE4|A7IZE4_ORYSI:tr|B8XEE9|B8XEE9_ORYSI
STNPWHSPRQG	42.95	1265.5901	11	633.8027	6.30	Glutelin	tr|A0A0D9VE94|A0A0D9VE94_9ORYZ:tr|B9F4T2|B9F4T2_ORYSJ:tr|A0A0E0NCF1|A0A0E0NCF1_ORYRU:tr|A0A0D3F334|A0A0D3F334_9ORYZ:tr|B9F4T1|B9F4T1_ORYSJ:tr|B9F4T3|B9F4T3_ORYSJ:tr|B8AEZ5|B8AEZ5_ORYSI:tr|T1T6C4|T1T6C4_ORYSI:tr|A1YQH4|A1YQH4_ORYSJ:Q02897|GLUB2_ORYSJ:tr|A1YQH6|A1YQH6_ORYSJ:tr|I1NZ08|I1NZ08_ORYGL:tr|Q0E2D5|Q0E2D5_ORYSJ:tr|A1YQH5|A1YQH5_ORYSJ:tr|A0A0E0CIL1|A0A0E0CIL1_9ORYZ:tr|A0A0E0NCF3|A0A0E0NCF3_ORYRU:tr|A0A0E0G6R1|A0A0E0G6R1_ORYNI:P14323|GLUB1_ORYSJ:tr|A0A0D9YPX0|A0A0D9YPX0_9ORYZ:tr|A0A0D3F336|A0A0D3F336_9ORYZ:tr|I1NZ10|I1NZ10_ORYGL:tr|Q0E2D2|Q0E2D2_ORYSJ:tr|T1T4F0|T1T4F0_ORYSI:tr|A0A0E0CIL2|A0A0E0CIL2_9ORYZ:tr|A2X2Z8|A2X2Z8_ORYSI
FDWVGGR	40.88	835.3976	7	418.7065	16.89	Glucose-6-phosphate isomerase	tr|Q84P59|Q84P59_ORYSJ:tr|A0A0D3H7U8|A0A0D3H7U8_9ORYZ:tr|A0A0E0QSI2|A0A0E0QSI2_ORYRU:tr|A0A0E0IKZ3|A0A0E0IKZ3_ORYNI:tr|A0A0E0B412|A0A0E0B412_9ORYZ:tr|A0A0D9X8Y6|A0A0D9X8Y6_9ORYZ:tr|A0A0E0QKB2|A0A0E0QKB2_ORYRU:tr|A0A0E0E500|A0A0E0E500_9ORYZ:tr|A0A0E0E501|A0A0E0E501_9ORYZ:tr|A0A0E0ET13|A0A0E0ET13_9ORYZ:tr|A0A0D9XEL8|A0A0D9XEL8_9ORYZ:tr|A0A0E0ET14|A0A0E0ET14_9ORYZ:tr|Q6ZB94|Q6ZB94_ORYSJ:tr|A3BU52|A3BU52_ORYSJ:tr|A0A0E0M265|A0A0E0M265_ORYPU:tr|I1QJH8|I1QJH8_ORYGL:tr|J3MY99|J3MY99_ORYBR:tr|A0A0D3H1D9|A0A0D3H1D9_9ORYZ:tr|A2YWA4|A2YWA4_ORYSI:tr|A0A0E0AWN3|A0A0E0AWN3_9ORYZ:tr|A0A0E0IDE9|A0A0E0IDE9_ORYNI:tr|Q76E42|Q76E42_ORYSJ:tr|B8BCM8|B8BCM8_ORYSI:tr|Q6YXI1|Q6YXI1_ORYSJ:tr|A0A0E0LVZ9|A0A0E0LVZ9_ORYPU:tr|A0A0E0M263|A0A0E0M263_ORYPU:tr|A0A0D9X8Y5|A0A0D9X8Y5_9ORYZ:tr|A0A0E0ET12|A0A0E0ET12_9ORYZ:tr|J3MTV1|J3MTV1_ORYBR
VVVGTPGRVF	37.73	1029.5970	10	515.8064	18.03	Eukaryotic initiation factor 4A-2	P41377|IF4A2_ARATH
VFNGVLRPG	37.18	957.5396	9	479.7769	15.86	Glutelin type-B 4	tr|A2X3A0|A2X3A0_ORYSI:tr|A2X399|A2X399_ORYSI:P14614|GLUB4_ORYSJ:tr|Q0E261|Q0E261_ORYSJ:tr|A0A0D9YQ79|A0A0D9YQ79_9ORY

Peptide	-10lgP	Mass (Da)	Length (amino acid n.)	m/z	RT (min)	Protein	Accession
							Z:tr|D6BV14|D6BV14_ORYSJ:tr|A0A0D3F3E6|A0A0D3F3E6_9ORYZ:tr|A0A0E0JY90|A0A0E0JY90_ORYPU:Q6ERU3|GLUB5_ORYSJ:tr|I1NZ94|I1NZ94_ORYGL:tr|A0A0D3F3E3|A0A0D3F3E3_9ORYZ:tr|A0A0E0JY91|A0A0E0JY91_ORYPU:tr|A0A0D3F3E5|A0A0D3F3E5_9ORYZ:tr|C0L8H2|C0L8H2_ORYSJ:tr|A3A5D6|A3A5D6_ORYSJ:tr|A0A0E0G4Q4|A0A0E0G4Q4_ORYNI:tr|A0A0E0NCT0|A0A0E0NCT0_ORYRU:tr|M1G571|M1G571_ORYSJ:tr|M1G2E3|M1G2E3_ORYSI:tr|A0A0D9VG85|A0A0D9VG85_9ORYZ:tr|Q0E2G5|Q0E2G5_ORYSJ:tr|J3LBL3|J3LBL3_ORYBR:tr|C0L8H1|C0L8H1_ORYSJ:tr|A0A0D9YLS5|A0A0D9YLS5_9ORYZ:tr|Q84X94|Q84X94_ORYSJ:tr|A0A0E0NCA6|A0A0E0NCA6_ORYRU:tr|Q84X93|Q84X93_ORYSJ:tr|A0A0E0G459|A0A0E0G459_ORYNI:tr|Q6ESW6|Q6ESW6_ORYSJ:tr|A2X2V1|A2X2V1_ORYSI
LDWYKGPT	36.37	978.4811	8	490.2474	17.89	Elongation factor	tr|E7BJ60|E7BJ60_ORYSI:tr|A0A0E0NQG9|A0A0E0NQG9_ORYRU:tr|A0A0P0VTT8|A0A0P0VTT8_ORYSJ:tr|A0A0E0CW33|A0A0E0CW33_9ORYZ:tr|A0A0E0CW26|A0A0E0CW26_9ORYZ:tr|Q10QZ5|Q10QZ5_ORYSJ:tr|A0A0E0GHL8|A0A0E0GHL8_ORYNI:tr|A0A0N7KGP4|A0A0N7KGP4_ORYSJ:tr|I1P848|I1P848_ORYGL:tr|B9FBM7|B9FBM7_ORYSJ:tr|J3LKK4|J3LKK4_ORYBR:tr|A0A0E0K9J4|A0A0E0K9J4_ORYPU:tr|A0A1L2JKK1|A0A1L2JKK1_ORYSA:tr|Q10QZ6|Q10QZ6_ORYSJ:tr|A0A0D3FEJ3|A0A0D3FEJ3_9ORYZ:tr|A0A0D9VSS7|A0A0D9VSS7_9ORYZ:tr|A0A0D3FEJ0|A0A0D3FEJ0_9ORYZ:O64937|EF1A_ORYSJ:tr|A0A0E0GHL3|A0A0E0GHL3_ORYNI:tr|B8APM5|B8APM5_ORYSI:tr|I1P851|I1P851_ORYGL:tr|A0A0E0K9J0|A0A0E0K9J0_ORYPU:tr|Q10QZ4|Q10QZ4_ORYSJ:tr|A0A0D3FEJ2|A0A0D3FEJ2_9ORYZ:tr|I1P850|I1P850_ORYGL:tr|A0A0D3FEJ4|A0A0D3FEJ4_9ORYZ:tr|A0A0D9Z2R9|A0A0D9Z2R9_9ORYZ:tr|A0A0D3FEJ1|A0A0D3FEJ1_9ORYZ
HGAFTPR	35.93	784.3980	7	393.2067	8.31	Glutelin	tr|A0A0D9VE94|A0A0D9VE94_9ORYZ:tr|B9F4T2|B9F4T2_ORYSJ:tr|A0A0E0NCF1|A0A0E0NCF1_ORYRU:tr|A0A0D3F334|A0A0D3F334_9ORYZ:tr|B9F4T1|B9F4T1_ORYSJ:tr|B9F4T3|B9F4T3_ORYSJ:tr|B8AEZ5|B8AEZ5_ORYSI:tr|T1T6C4|T1T6C4_ORYSI:tr|A1YQH4|A1YQH4_ORYSJ:Q02897|GLUB2_ORYSJ:tr|A1YQH6|A1YQH6_ORYSJ:tr|I1NZ08|I1NZ08_ORYGL:tr|Q0E2D5|Q0E2D5_ORYSJ:tr|A1YQH5|A1YQH5_ORYSJ:tr|A0A0E0CIL1|A0A0E0CIL1_9ORYZ:tr|A0A0E0NCF3|A0A0E0NCF3_ORYRU:tr|A0A0E0G6R1|A0A0E0G6R1_ORYNI:P14323|GLUB1_ORYSJ:tr|A0A0D9YPX0|A0A0D9YPX0_9ORYZ:tr|A0A0D3F336|A0A0D3F336_9ORYZ:tr|I1NZ10|I1NZ10_ORYGL:tr|Q0E2D2|Q0E2D2_ORYSJ:tr|T1T4F0|T1T4F0_ORYSI:tr|A0A0E0CIL2|A0A0E0CIL2_9ORYZ

<table>
<tr><th>Peptide</th><th>-10lgP</th><th>Mass (Da)</th><th>Length (amino acid n.)</th><th>m/z</th><th>RT (min)</th><th>Protein</th><th>Accession</th></tr>
<tr><td>WIDFPRAPQ</td><td>34.87</td><td>1128.5715</td><td>9</td><td>565.2937</td><td>21.65</td><td>Os02g0528200 protein</td><td>tr|A0A0N7KFE7|A0A0N7KFE7_ORYSJ:tr|A0A0D3F5W2|A0A0D3F5W2_9ORYZ:tr|A0A0E0K0K5|A0A0E0K0K5_ORYPU:tr|A0A0E0G7M7|A0A0E0G7M7_ORYNI:tr|A0A0E0NFS6|A0A0E0NFS6_ORYRU:tr|A0A0D9YT34|A0A0D9YT34_9ORYZ:tr|A0A0E0K0K4|A0A0E0K0K4_ORYPU:tr|A0A0E0CLF1|A0A0E0CLF1_9ORYZ:tr|Q6H6P8|Q6H6P8_ORYSJ:tr|Q40663|Q40663_ORYSA:tr|A2X5K0|A2X5K0_ORYSI:tr|D0TZK1|D0TZK1_ORYSI:tr|I1P0X2|I1P0X2_ORYGL:tr|B3VDJ4|B3VDJ4_ORYSJ:tr|I6VRB8|I6VRB8_ORYSJ:tr|A0A0D9VGL1|A0A0D9VGL1_9ORYZ</td></tr>
<tr><td>TNPWHSPR</td><td>34.15</td><td>993.4780</td><td>8</td><td>497.7464</td><td>6.53</td><td>Glutelin</td><td>tr|A0A0D9VE94|A0A0D9VE94_9ORYZ:tr|B9F4T2|B9F4T2_ORYSJ:tr|A0A0E0NCF1|A0A0E0NCF1_ORYRU:tr|A0A0D3F334|A0A0D3F334_9ORYZ:tr|B9F4T1|B9F4T1_ORYSJ:tr|B9F4T3|B9F4T3_ORYSJ:tr|B8AEZ5|B8AEZ5_ORYSI:tr|T1T6C4|T1T6C4_ORYSI:tr|A1YQH4|A1YQH4_ORYSJ:Q02897|GLUB2_ORYSJ:tr|A1YQH6|A1YQH6_ORYSJ:tr|I1NZ08|I1NZ08_ORYGL:tr|Q0E2D5|Q0E2D5_ORYSJ:tr|A1YQH5|A1YQH5_ORYSJ:tr|A0A0E0CIL1|A0A0E0CIL1_9ORYZ:tr|A0A0E0NCF3|A0A0E0NCF3_ORYRU:tr|A0A0E0G6R1|A0A0E0G6R1_ORYNI:P14323|GLUB1_ORYSJ:tr|A0A0D9YPX0|A0A0D9YPX0_9ORYZ:tr|A0A0D3F336|A0A0D3F336_9ORYZ:tr|I1NZ10|I1NZ10_ORYGL:tr|Q0E2D2|Q0E2D2_ORYSJ:tr|T1T4F0|T1T4F0_ORYSI:tr|A0A0E0CIL2|A0A0E0CIL2_9ORYZ:tr|A2X2Z8|A2X2Z8_ORYSI</td></tr>
<tr><td>H(+154.10)GAFTPR</td><td>33.84</td><td>938.4974</td><td>7</td><td>470.2563</td><td>16.84</td><td>Glutelin</td><td>tr|A0A0D9VE94|A0A0D9VE94_9ORYZ:tr|B9F4T2|B9F4T2_ORYSJ:tr|A0A0E0NCF1|A0A0E0NCF1_ORYRU:tr|A0A0D3F334|A0A0D3F334_9ORYZ:tr|B9F4T1|B9F4T1_ORYSJ:tr|B9F4T3|B9F4T3_ORYSJ:tr|B8AEZ5|B8AEZ5_ORYSI:tr|T1T6C4|T1T6C4_ORYSI:tr|A1YQH4|A1YQH4_ORYSJ:Q02897|GLUB2_ORYSJ:tr|A1YQH6|A1YQH6_ORYSJ:tr|I1NZ08|I1NZ08_ORYGL:tr|Q0E2D5|Q0E2D5_ORYSJ:tr|A1YQH5|A1YQH5_ORYSJ:tr|A0A0E0CIL1|A0A0E0CIL1_9ORYZ:tr|A0A0E0NCF3|A0A0E0NCF3_ORYRU:tr|A0A0E0G6R1|A0A0E0G6R1_ORYNI:P14323|GLUB1_ORYSJ:tr|A0A0D9YPX0|A0A0D9YPX0_9ORYZ:tr|A0A0D3F336|A0A0D3F336_9ORYZ:tr|I1NZ10|I1NZ10_ORYGL:tr|Q0E2D2|Q0E2D2_ORYSJ:tr|T1T4F0|T1T4F0_ORYSI:tr|A0A0E0CIL2|A0A0E0CIL2_9ORYZ</td></tr>
<tr><td>IPPPRGPLW</td><td>32.55</td><td>1031.5916</td><td>9</td><td>516.8027</td><td>20.91</td><td>Aspartic protease</td><td>tr|Q93XE4|Q93XE4_ORYSJ:tr|A0A0P0WR25|A0A0P0WR25_ORYSJ:tr|A0A0E0D6E9|A0A0E0D6E9_9ORYZ:tr|A0A0P0V679|A0A0P0V679_ORYSJ:tr|A0A0E0KY87|A0A0E0KY87_ORYPU:tr|A0A0E0PH77|A0A0E0PH77_ORYRU:tr|A0A0E0H931|A0A0E0H931_ORYNI:tr|A0A0D3G2Z6|A0A0D3G2Z6_9ORYZ:P42211|ASPRX_ORYSJ:tr|A0A0D9WCH7|A0A0D9WCH7_9ORYZ:tr|B9FMC0|B9FMC0_ORYSJ:tr|A0A0E0C5N5|A0A0E0C5N5_9ORYZ:tr|A0A0D9WJU6|A0A0D9WJU6_9ORYZ:tr|A0A0E0D6E6|A0A0E0D6E6_9ORYZ:tr|A0A0D3GAX9|A0A0D3GAX9_9ORYZ:tr|A0A0E0HIK1|A0A0E0HIK1_ORYNI:tr|A2Y7J9|A2Y7J9_ORYSI:tr|A0A0E0PRC6|A0A0E0PRC6_ORYRU:tr|A0A0E0A2X2|A0A0E0A2X2_9ORYZ:Q42456|ASPR1_ORYSJ:tr|Q0DFW</td></tr>
</table>

Peptide	-10lgP	Mass (Da)	Length (amino acid n.)	m/z	RT (min)	Protein	Accession
							1|Q0DFW1_ORYSJ:tr|A0A0E0L5U7|A0A0E0L5U7_ORYPU:tr|A0A0E0JM18|A0A0E0JM18_ORYPU:tr|A0A0E0C5N4|A0A0E0C5N4_9ORYZ:tr|A0A0E0N0C1|A0A0E0N0C1_ORYRU:tr|B8A762|B8A762_ORYSI:tr|Q0JKM8|Q0JKM8_ORYSJ:tr|A0A0E0D6E8|A0A0E0D6E8_9ORYZ:tr|A0A0E0A2X1|A0A0E0A2X1_9ORYZ:tr|A0A0E0PRC4|A0A0E0PRC4_ORYRU:tr|A0A0E0L5U6|A0A0E0L5U6_ORYPU
RYGAVGVVF	32.33	966.5287	9	484.2720	23.93	Uncharacterized protein	tr|A0A0E0GUU4|A0A0E0GUU4_ORYNI
HS(-18.01)AFTPR	32.28	796.3980	7	399.2061	5.80	Uncharacterized protein	tr|A2X2Z8|A2X2Z8_ORYSI
TNPWHSPRQGS	32.26	1265.5901	11	633.8027	6.30	Glutelin	tr|A0A0D9VE94|A0A0D9VE94_9ORYZ:tr|B9F4T2|B9F4T2_ORYSJ:tr|A0A0E0NCF1|A0A0E0NCF1_ORYRU:tr|A0A0D3F334|A0A0D3F334_9ORYZ:tr|B9F4T1|B9F4T1_ORYSJ:tr|B9F4T3|B9F4T3_ORYSJ:tr|B8AEZ5|B8AEZ5_ORYSI:tr|T1T6C4|T1T6C4_ORYSI:tr|A1YQH4|A1YQH4_ORYSJ:Q02897|GLUB2_ORYSJ:tr|A1YQH6|A1YQH6_ORYSJ:tr|I1NZ08|I1NZ08_ORYGL:tr|Q0E2D5|Q0E2D5_ORYSJ:tr|A1YQH5|A1YQH5_ORYSJ:tr|A0A0E0CIL1|A0A0E0CIL1_9ORYZ:tr|A0A0E0NCF3|A0A0E0NCF3_ORYRU:tr|A0A0E0G6R1|A0A0E0G6R1_ORYNI:P14323|GLUB1_ORYSJ:tr|A0A0D9YPX0|A0A0D9YPX0_9ORYZ:tr|A0A0D3F336|A0A0D3F336_9ORYZ:tr|I1NZ10|I1NZ10_ORYGL:tr|Q0E2D2|Q0E2D2_ORYSJ:tr|T1T4F0|T1T4F0_ORYSI:tr|A0A0E0CIL2|A0A0E0CIL2_9ORYZ:tr|A2X2Z8|A2X2Z8_ORYSI
GQKPVTFF	31.74	922.4912	8	462.2526	19.27	Alanine aminotransferase	tr|Q9S768|Q9S768_ORYSA:tr|I1QU38|I1QU38_ORYGL:tr|B8BGM4|B8BGM4_ORYSI:tr|Q338N8|Q338N8_ORYSJ:tr|A0A0E0QYK7|A0A0E0QYK7_ORYRU:tr|A0A0D3HD76|A0A0D3HD76_9ORYZ:tr|Q94HC5|Q94HC5_ORYSJ:tr|A0A0E0IRN1|A0A0E0IRN1_ORYNI:tr|A0A0E0QYK6|A0A0E0QYK6_ORYRU:tr|A0A0E0B9Z7|A0A0E0B9Z7_9ORYZ:tr|A0A0E0B9Z9|A0A0E0B9Z9_9ORYZ:tr|A0A0P0XUE4|A0A0P0XUE4_ORYSJ:tr|A0A0E0IRN2|A0A0E0IRN2_ORYNI:tr|A0A0D3HD77|A0A0D3HD77_9ORYZ:tr|A0A0E0B9Z8|A0A0E0B9Z8_9ORYZ
IGRPAPMPY	31.07	1000.5164	9	501.2657	14.18	Uncharacterized protein	tr|A3BEL8|A3BEL8_ORYSJ:tr|A0A0E0AD80|A0A0E0AD80_9ORYZ
GGFPHYG	28.62	733.3184	7	367.6666	11.58	60S ribosomal protein l3	tr|A6MZT4|A6MZT4_ORYSI:tr|Q6XEB7|Q6XEB7_ORYSI:tr|A0A0E0MCV3|A0A0E0MCV3_ORYPU:tr|Q53JG0|Q53JG0_ORYSJ:tr|A0A0E0F8N9|A0A0E0F8N9_9ORYZ:tr|A5D934|A5D934_ORYSA:tr|A0A0E0MK40|A0A0E0M

Peptide	-10lgP	Mass (Da)	Length (amino acid n.)	m/z	RT (min)	Protein	Accession
							K40_ORYPU:tr|J3N6B6|J3N6B6_ORYBR:tr|J7LIN0|J7LIN0_ORYSA:tr|Q0IPU6|Q0IPU6_ORYSJ:tr|A0A0D3HIP3|A0A0D3HIP3_9ORYZ:tr|J3NBP7|J3NBP7_ORYBR:tr|A2ZBV5|A2ZBV5_ORYSI:tr|A0A0E0J781|A0A0E0J781_ORYNI:tr|A0A0D9XX94|A0A0D9XX94_9ORYZ:tr|A0A0E0BPI6|A0A0E0BPI6_9ORYZ:P35684|RL3_ORYSJ:tr|I1QY10|I1QY10_ORYGL:tr|A0A0E0IYP9|A0A0E0IYP9_ORYNI:tr|A0A0E0R4P3|A0A0E0R4P3_ORYRU:tr|A2ZIF8|A2ZIF8_ORYSI:tr|A0A0E0BFS9|A0A0E0BFS9_9ORYZ:tr|A0A0D9XPH0|A0A0D9XPH0_9ORYZ
DALQPPHKYVPW	25.91	1449.7405	12	725.8778	20.02	Gamma interferon inducible lysosomal thiol reductase family protein, expressed	tr|Q10MT7|Q10MT7_ORYSJ
IDFPRAPQ	25.10	942.4922	8	472.2535	15.86	Os02g0528200 protein	tr|A0A0N7KFE7|A0A0N7KFE7_ORYSJ:tr|A0A0D3F5W2|A0A0D3F5W2_9ORYZ:tr|A0A0E0K0K5|A0A0E0K0K5_ORYPU:tr|A0A0E0G7M7|A0A0E0G7M7_ORYNI:tr|A0A0E0NFS6|A0A0E0NFS6_ORYRU:tr|A0A0D9YT34|A0A0D9YT34_9ORYZ:tr|A0A0E0K0K4|A0A0E0K0K4_ORYPU:tr|A0A0E0CLF1|A0A0E0CLF1_9ORYZ:tr|Q6H6P8|Q6H6P8_ORYSJ:tr|Q40663|Q40663_ORYSA:tr|A2X5K0|A2X5K0_ORYSI:tr|D0TZK1|D0TZK1_ORYSI:tr|I1P0X2|I1P0X2_ORYGL:tr|B3VDJ4|B3VDJ4_ORYSJ:tr|I6VRB8|I6VRB8_ORYSJ:tr|A0A0D9VGL1|A0A0D9VGL1_9ORYZ
WIDFPRAP	24.50	1000.5130	8	501.2642	23.44	Os02g0528200 protein	tr|A0A0N7KFE7|A0A0N7KFE7_ORYSJ:tr|A0A0D3F5W2|A0A0D3F5W2_9ORYZ:tr|A0A0E0K0K5|A0A0E0K0K5_ORYPU:tr|A0A0E0G7M7|A0A0E0G7M7_ORYNI:tr|A0A0E0NFS6|A0A0E0NFS6_ORYRU:tr|A0A0D9YT34|A0A0D9YT34_9ORYZ:tr|A0A0E0K0K4|A0A0E0K0K4_ORYPU:tr|A0A0E0CLF1|A0A0E0CLF1_9ORYZ:tr|Q6H6P8|Q6H6P8_ORYSJ:tr|Q40663|Q40663_ORYSA:tr|A2X5K0|A2X5K0_ORYSI:tr|D0TZK1|D0TZK1_ORYSI:tr|I1P0X2|I1P0X2_ORYGL:tr|B3VDJ4|B3VDJ4_ORYSJ:tr|I6VRB8|I6VRB8_ORYSJ:tr|A0A0D9VGL1|A0A0D9VGL1_9ORYZ
GGVSCGEY	23.96	770.2905	8	386.1537	20.72	RBR-type E3 ubiquitin transferase	tr|A0A0D3H4N3|A0A0D3H4N3_9ORYZ:tr|A3BWW9|A3BWW9_ORYSJ:tr|A0A0E0IHE1|A0A0E0IHE1_ORYNI:tr|Q6EPS5|Q6EPS5_ORYSJ:tr|A0A0E0QP44|A0A0E0QP44_ORYRU:tr|A0A0E0B0T0|A0A0E0B0T0_9ORYZ:tr|B8BA89|B8BA89_ORYSI

Peptide	-10lgP	Mass (Da)	Length (amino acid n.)	m/z	RT (min)	Protein	Accession
LRAVTNVGVSLGA VL	20.79	1467.877 3	15	734.9404	21.39	MFS transporter	tr|A0A3N1M4F8|A0A3N1M4F8_9ACTN
SRPDFRF	20.59	923.4613	7	462.7384	13.89	Os10g018 9100 protein	tr|A0A0E0EWF5|A0A0E0EWF5_9ORYZ:tr|A0A0E0EWF4|A0A0E0EWF4_9ORYZ:tr|A0A0E0EWF3|A0A0E0EWF3_9ORYZ:tr|A0A0E0IQA4|A0A0E0IQA4_ORYNI:tr|A0A0D3HBW4|A0A0D3HBW4_9ORYZ:tr|B8BG13|B8BG13_ORYSI:tr|A0A0E0QX42|A0A0E0QX42_ORYRU:tr|A0A0E0B8J4|A0A0E0B8J4_9ORYZ:tr|Q33AE4|Q33AE4_ORYSJ:tr|Q53QR8|Q53QR8_ORYSJ:tr|A0A0E0M659|A0A0E0M659_ORYPU
PTRFPQK	20.51	872.4868	7	437.2507	5.98	Uncharact erized protein	tr|J3NCE6|J3NCE6_ORYBR:Q8GU83|AB41G_ORYSJ:Q2QV81|AB49G_ORYSJ:tr|A0A0E0MKZ6|A0A0E0MKZ6_ORYPU

Sub-fraction n. 24. Total: 17 identified peptides, 11 peptides with rice protein accession. RT, retention time.

Peptide	-10lgP	Mass (Da)	Length (amino acid n.)	m/z	RT (min)	Protein	Accession
TNPWHSPRQG	53.08	1178.558 1	10	590.2863	6.56	Glutelin	tr|A2X2Z8|A2X2Z8_ORYSI:tr|A0A0D9VE94|A0A0D9VE94_9ORYZ:tr|B9F4T2|B9F4T2_ORYSJ:tr|A0A0D3F334|A0A0D3F334_9ORYZ:tr|A0A0E0NCF1|A0A0E0NCF1_ORYRU:tr|B9F4T1|B9F4T1_ORYSJ:tr|B8AEZ5|B8AEZ5_ORYSI:tr|B9F4T3|B9F4T3_ORYSJ:tr|T1T6C4|T1T6C4_ORYSI:tr|A1YQH5|A1YQH5_ORYSJ:tr|Q0E2D5|Q0E2D5_ORYSJ:tr|I1NZ08|I1NZ08_ORYGL:tr|A1YQH6|A1YQH6_ORYSJ:Q02897|GLUB2_ORYSJ:tr|A1YQH4|A1YQH4_ORYSJ:tr|A0A0E0CIL1|A0A0E0CIL1_9ORYZ:tr|T1T4F0|T1T4F0_ORYSI:tr|Q0E2D2|Q0E2D2_ORYSJ:tr|I1NZ10|I1NZ10_ORYGL:tr|A0A0D3F336|A0A0D3F336_9ORYZ:tr|A0A0D9YPX0|A0A0D9YPX0_9ORYZ:P14323|GLUB1_ORYSJ:tr|A0A0E0G6R1|A0A0E0G6R1_ORYNI:tr|A0A0E0NCF3|A0A0E0NCF3_ORYRU:tr|A0A0E0CIL2|A0A0E0CIL2_9ORYZ
TNPWHSPRQ	47.39	1121.536 6	9	561.7757	6.59	Glutelin	tr|A2X2Z8|A2X2Z8_ORYSI:tr|A0A0D9VE94|A0A0D9VE94_9ORYZ:tr|B9F4T2|B9F4T2_ORYSJ:tr|A0A0D3F334|A0A0D3F334_9ORYZ:tr|A0A0E0NCF1|A0A0E0NCF1_ORYRU:tr|B9F4T1|B9F4T1_ORYSJ:tr|B8AEZ5|B8AEZ5_ORYSI:tr|B9F4T3|B9F4T3_ORYSJ:tr|T1T6C4|T1T6C4_ORYSI:tr|A1YQH5|A1YQH5_ORYSJ:tr|Q0E2D5|Q0E2D5_ORYSJ:tr|I1NZ08|I1NZ08_ORYGL:tr|A1YQH6|A1YQH6_ORYSJ:Q02897|GLUB2_ORYSJ:tr|A1YQH4|A1YQH4_ORYSJ:tr|A0A0E0CIL1|A0A0E0CIL1_9ORYZ:tr|T1T4F0|T1T4F0_ORYSI:tr|Q0E2D2|Q0E2D2_ORYSJ:tr|I1NZ10|I1NZ10_ORYGL:tr|A0A0D3F336|A0A0D3F336_9ORYZ:tr|A0A0D9YPX0|A0A0D9YPX0_9ORYZ:P1432

							3|GLUB1_ORYSJ:tr|A0A0E0G6R1|A0A0E0G6R1_ORYNI:tr|A0A0E0NCF3|A0A0E0NCF3_ORYRU:tr|A0A0E0CIL2|A0A0E0CIL2_9ORYZ
HS(-18.01)AFTPR	46.00	796.3980	7	399.2070	6.07	Uncharacterized protein	tr|A2X2Z8|A2X2Z8_ORYSI
TNPWHSPR	44.89	993.4780	8	497.7464	6.82	Glutelin	tr|A2X2Z8|A2X2Z8_ORYSI:tr|A0A0D9VE94|A0A0D9VE94_9ORYZ:tr|B9F4T2|B9F4T2_ORYSJ:tr|A0A0D3F334|A0A0D3F334_9ORYZ:tr|A0A0E0NCF1|A0A0E0NCF1_ORYRU:tr|B9F4T1|B9F4T1_ORYSJ:tr|B8AEZ5|B8AEZ5_ORYSI:tr|B9F4T3|B9F4T3_ORYSJ:tr|T1T6C4|T1T6C4_ORYSI:tr|A1YQH5|A1YQH5_ORYSJ:tr|Q0E2D5|Q0E2D5_ORYSJ:tr|I1NZ08|I1NZ08_ORYGL:tr|A1YQH6|A1YQH6_ORYSJ:Q02897|GLUB2_ORYSJ:tr|A1YQH4|A1YQH4_ORYSJ:tr|A0A0E0CIL1|A0A0E0CIL1_9ORYZ:tr|T1T4F0|T1T4F0_ORYSI:tr|Q0E2D2|Q0E2D2_ORYSJ:tr|I1NZ10|I1NZ10_ORYGL:tr|A0A0D3F336|A0A0D3F336_9ORYZ:tr|A0A0D9YPX0|A0A0D9YPX0_9ORYZ:P14323|GLUB1_ORYSJ:tr|A0A0E0G6R1|A0A0E0G6R1_ORYNI:tr|A0A0E0NCF3|A0A0E0NCF3_ORYRU:tr|A0A0E0CIL2|A0A0E0CIL2_9ORYZ
LNNNPYFK	42.36	1008.5029	8	505.2590	12.12	Starch synthase, chloroplastic/amyloplastic	tr|A0A0E0DW79|A0A0E0DW79_9ORYZ:tr|A8QXE7|A8QXE7_ORYSI:tr|V5NEJ7|V5NEJ7_ORYSA:tr|A0A0E0DW80|A0A0E0DW80_9ORYZ:tr|A0EQH2|A0EQH2_ORYSJ:tr|A0EQK4|A0EQK4_ORYRU:tr|A0EQK6|A0EQK6_ORYRU:tr|A0EQK5|A0EQK5_ORYRU:tr|A0EQD4|A0EQD4_ORYSI:tr|A0EQK7|A0EQK7_ORYRU:tr|A0EQE0|A0EQE0_ORYSA:P0C585|SSG1_ORYSA:tr|C8CBL1|C8CBL1_ORYSJ:tr|B1B5Z1|B1B5Z1_ORYSI:tr|B1B5Z0|B1B5Z0_ORYSI:A2Y8X2|SSG1_ORYSI:Q42968|SSG1_ORYGL:tr|A0A076FRI5|A0A076FRI5_ORYSJ:tr|B8XEK2|B8XEK2_ORYSA:tr|B8XEJ2|B8XEJ2_ORYSA:tr|A0A3Q9T3Z7|A0A3Q9T3Z7_ORYSA:tr|B8XEJ7|B8XEJ7_ORYSA:tr|D3U2H9|D3U2H9_ORYSA:tr|A0A3Q9T378|A0A3Q9T378_ORYSA:tr|D0TZY6|D0TZY6_ORYSI:tr|B8XEK3|B8XEK3_ORYSA:tr|B8XEJ8|B8XEJ8_ORYSA:tr|A0A0D9WLF6|A0A0D9WLF6_9ORYZ:tr|A0A0E0A4K1|A0A0E0A4K1_9ORYZ
HGAFTPR	40.98	784.3980	7	393.2062	5.78	Glutelin	tr|A0A0D9VE94|A0A0D9VE94_9ORYZ:tr|B9F4T2|B9F4T2_ORYSJ:tr|A0A0D3F334|A0A0D3F334_9ORYZ:tr|A0A0E0NCF1|A0A0E0NCF1_ORYRU:tr|B9F4T1|B9F4T1_ORYSJ:tr|B8AEZ5|B8AEZ5_ORYSI:tr|B9F4T3|B9F4T3_ORYSJ:tr|T1T6C4|T1T6C4_ORYSI:tr|A1YQH5|A1YQH5_ORYSJ:tr|Q0E2D5|Q0E2D5_ORYSJ:tr|I1NZ08|I1NZ08_ORYGL:tr|A1YQH6|A1YQH6_ORYSJ:Q02897|GLUB2_ORYSJ:tr|A1YQH4|A1YQH4_ORYSJ:tr|A0A0E0CIL1|A0A0E0CIL1_9ORYZ:tr|T1T4F0|T1T4F0_ORYSI:tr|Q0E2D2|Q0E2D2_ORYSJ:tr|I1NZ10|I1NZ10_ORYGL:tr|A0A0D3F336|A0A0D3F336_9ORYZ:tr|A0A0D9YPX0|A0A0D9YPX0_9ORYZ:P14323|GLUB1_ORYSJ:tr|A0A0E0G6R1|A0A0E0G6R1_ORYNI:tr|A0A0E0NCF3|A0A0E0NCF3_ORYRU:tr|A0A0E0CIL2|A0A0E0CIL2_9ORYZ
APWSKTGGL	37.76	915.4814	9	458.7490	15.81	Starch synthase,	tr|A0A0E0DW79|A0A0E0DW79_9ORYZ:tr|A8QXE7|A8QXE7_ORYSI:tr|V5NEJ7|V5NEJ7_ORYSA:tr|A0A0E0DW80|A0A0E0DW80_9ORYZ:tr|A0EQH2|A0EQH2_ORYSJ:tr|A0EQK4|A0EQK4_ORYRU:tr|A0EQK6|A0EQK6_O

						chloroplastic/amyloplastic	RYRU:tr|A0EQK5|A0EQK5_ORYRU:tr|A0EQD4|A0EQD4_ORYSI:tr|A0EQK7|A0EQK7_ORYRU:tr|A0EQE0|A0EQE0_ORYSA:P0C585|SSG1_ORYSA:tr|C8CBL1|C8CBL1_ORYSJ:tr|B1B5Z1|B1B5Z1_ORYSI:tr|B1B5Z0|B1B5Z0_ORYSI:A2Y8X2|SSG1_ORYSI:Q42968|SSG1_ORYGL:tr|A0A076FRI5|A0A076FRI5_ORYSJ:tr|B8XEK2|B8XEK2_ORYSA:tr|B8XEJ2|B8XEJ2_ORYSA:tr|A0A3Q9T3Z7|A0A3Q9T3Z7_ORYSA:tr|B8XEJ7|B8XEJ7_ORYSA:tr|D3U2H9|D3U2H9_ORYSA:tr|A0A3Q9T378|A0A3Q9T378_ORYSA:tr|D0TZY6|D0TZY6_ORYSI:tr|B8XEK3|B8XEK3_ORYSA:tr|B8XEJ8|B8XEJ8_ORYSA:tr|A0A0D9WLF6|A0A0D9WLF6_9ORYZ:tr|A0A0E0A4K1|A0A0E0A4K1_9ORYZ
RYKDTWPL	35.52	1077.5607	8	539.7880	16.59	Os08g0545200 protein	tr|A0A0D9XAG1|A0A0D9XAG1_9ORYZ:tr|A0A0E0ENL8|A0A0E0ENL8_9ORYZ:tr|A0A0E0LXL4|A0A0E0LXL4_ORYPU:tr|B8B9C4|B8B9C4_ORYSI:tr|A0A0E0IFD3|A0A0E0IFD3_ORYNI:tr|B8B9C5|B8B9C5_ORYSI:tr|Q6ZBH2|Q6ZBH2_ORYSJ:tr|I1QLA2|I1QLA2_ORYGL:tr|A0A0E0QLZ3|A0A0E0QLZ3_ORYRU:tr|A0A0D3H2T7|A0A0D3H2T7_9ORYZ:tr|A0A0E0AYG5|A0A0E0AYG5_9ORYZ
VKGFPRP	34.82	799.4704	7	400.7427	9.13	Os04g0404400 protein	tr|Q0JDG9|Q0JDG9_ORYSJ:tr|A0A0P0WA63|A0A0P0WA63_ORYSJ:tr|A0A0D9W4I7|A0A0D9W4I7_9ORYZ:tr|A0A0E0DDL8|A0A0E0DDL8_9ORYZ:tr|A0A0E0H0B3|A0A0E0H0B3_ORYNI:tr|A0A0D9ZKB8|A0A0D9ZKB8_9ORYZ:tr|A0A0E0KQ59|A0A0E0KQ59_ORYPU:tr|I1PKX3|I1PKX3_ORYGL:tr|Q01L47|Q01L47_ORYSA:tr|Q7X6I8|Q7X6I8_ORYSJ:tr|A2XT28|A2XT28_ORYSI:tr|A0A0D3FVG1|A0A0D3FVG1_9ORYZ:tr|A0A0E0P8P0|A0A0E0P8P0_ORYRU
FYDPKTPFF	31.42	1160.5542	9	581.2841	25.44	Glucose-1-phosphate adenylyltransferase large subunit 2, cytosolic	Q7G065|GLGL2_ORYSJ
FKVPDWF	31.10	937.4697	7	469.7426	28.95	CBL-interacting protein kinase 20	Q60EY8|CIPKK_ORYSJ:tr|A0A0E0PIV5|A0A0E0PIV5_ORYRU

PROTAMEX fraction P1

Sub-fraction n. 10. Total: 26 identified peptides, all with rice protein accession. RT, retention time.

Peptide	-10lgP	Mass (Da)	Length (amino acid n.)	m/z	RT (min)	Protein	Accession
NLNNNPYFKGT	56.61	1280.6150	11	641.3150	14.25	Granule-bound starch synthase I	tr|A0A0H4BM25|A0A0H4BM25_ORYSI:tr|A0A0E0DW79|A0A0E0DW79_9ORYZ:tr|A8QXE7|A8QXE7_ORYSI:tr|V5NEJ7|V5NEJ7_ORYSA:tr|A0A0E0DW80|A0A0E0DW80_9ORYZ:tr|A0EQH2|A0EQH2_ORYSJ:tr|A0EQK4|A0EQK4_ORYRU:tr|A0EQK6|A0EQK6_ORYRU:tr|A0EQK5|A0EQK5_ORYRU:tr|A0EQD4|A0EQD4_ORYSI:tr|A0EQK7|A0EQK7_ORYRU:tr|A0EQE0|A0EQE0_ORYSA:P0C585|SSG1_ORYSA:tr|C8CBL4|C8CBL4_ORYSJ:tr|C8CBL1|C8CBL1_ORYSJ:tr|B1B5Z1|B1B5Z1_ORYSI:tr|B1B5Z0|B1B5Z0_ORYSI:A2Y8X2|SSG1_ORYSI:Q42968|SSG1_ORYGL:tr|A0A076FRI5|A0A076FRI5_ORYSJ:tr|B8XEK2|B8XEK2_ORYSA:tr|B8XEJ2|B8XEJ2_ORYSA:tr|A0A3Q9T3Z7|A0A3Q9T3Z7_ORYSA:tr|B8XEJ7|B8XEJ7_ORYSA:tr|D3U2H9|D3U2H9_ORYSA:tr|A0A3Q9T378|A0A3Q9T378_ORYSA:tr|D0TZY6|D0TZY6_ORYSI:tr|B8XEK3|B8XEK3_ORYSA:tr|B8XEJ8|B8XEJ8_ORYSA:tr|A0A0D9WLF6|A0A0D9WLF6_9ORYZ:tr|A0A0E0A4K1|A0A0E0A4K1_9ORYZ
GKTVFDGVLRPG	51.36	1244.6876	12	623.3521	17.30	Glutelin	tr|B9F4T3|B9F4T3_ORYSJ:tr|Q0E2D2|Q0E2D2_ORYSJ:P14323|GLUB1_ORYSJ:tr|A0A0E0NCF3|A0A0E0NCF3_ORYRU:tr|B9F4T1|B9F4T1_ORYSJ:tr|A1YQH5|A1YQH5_ORYSJ:tr|Q0E2D5|Q0E2D5_ORYSJ:Q02897|GLUB2_ORYSJ:tr|A1YQH4|A1YQH4_ORYSJ:tr|B9F4T2|B9F4T2_ORYSJ:tr|I1NZ08|I1NZ08_ORYGL:tr|A0A0E0NCF1|A0A0E0NCF1_ORYRU:tr|B8AEZ5|B8AEZ5_ORYSI:tr|T1T6C4|T1T6C4_ORYSI:tr|A1YQH6|A1YQH6_ORYSJ:tr|A0A0E0CIL1|A0A0E0CIL1_9ORYZ:tr|T1T4F0|T1T4F0_ORYSI:tr|I1NZ10|I1NZ10_ORYGL:tr|A0A0D3F336|A0A0D3F336_9ORYZ:tr|A0A0D9YPX0|A0A0D9YPX0_9ORYZ:tr|A0A0E0G6R1|A0A0E0G6R1_ORYNI:tr|A0A0E0CIL2|A0A0E0CIL2_9ORYZ:tr|A0A0D3F334|A0A0D3F334_9ORYZ
NN(+.98)NPYFK	46.97	896.4028	7	449.2090	12.34	Granule-bound starch synthase I	tr|A0A0H4BM25|A0A0H4BM25_ORYSI:tr|A0A0E0DW79|A0A0E0DW79_9ORYZ:tr|A8QXE7|A8QXE7_ORYSI:tr|V5NEJ7|V5NEJ7_ORYSA:tr|A0A0E0DW80|A0A0E0DW80_9ORYZ:tr|A0EQH2|A0EQH2_ORYSJ:tr|A0EQK4|A0EQK4_ORYRU:tr|A0EQK6|A0EQK6_ORYRU:tr|A0EQK5|A0EQK5_ORYRU:tr|A0EQD4|A0EQD4_ORYSI:tr|A0EQK7|A0EQK7_ORYRU:tr|A0EQE0|A0EQE0_ORYSA:P0C585|SSG1_ORYSA:tr|C8CBL4|C8CBL4_ORYSJ:tr|C8CBL1|C8CBL1_ORYSJ:tr|B1B5Z1|B1B5Z1_ORYSI:tr|B1B5Z0|B1B5Z0_ORYSI:A2Y8X2|SSG1_ORYSI:Q42968|SSG1_ORYGL:tr|A0A076FRI5|A0A076FRI5_ORYSJ:tr|B8XEK2|B8XEK2_ORYSA:tr|B8XEJ2|B8XEJ2_ORYSA:tr|A0A3Q9T3Z7|A0A3Q9T3Z7_ORYSA:tr|B8XEJ7|B8XEJ7_ORYSA:tr|D3U2H9|D3U2H9_ORYSA:tr|A0A3Q9T378|A0A3Q9T378_ORYSA:tr|D0TZY6|D0TZY6_ORYSI:tr|B8XEK3|B8XEK3_ORYSA:tr|B8XEJ8|B8XEJ8_ORYSA:tr|A0A0D9WLF6|A0A0D9WLF6_9ORYZ:tr|A0A0E0A4K1|A0A0E0A4K1_9ORYZ

H(+154.10)GAFTPR	43.95	938.4974	7	470.2558	16.81	Glutelin	tr|B9F4T3|B9F4T3_ORYSJ:tr|Q0E2D2|Q0E2D2_ORYSJ:P14323|GLUB1_ORYSJ:tr|A0A0E0NCF3|A0A0E0NCF3_ORYRU:tr|B9F4T1|B9F4T1_ORYSJ:tr|A1YQH5|A1YQH5_ORYSJ:tr|Q0E2D5|Q0E2D5_ORYSJ:Q02897|GLUB2_ORYSJ:tr|A1YQH4|A1YQH4_ORYSJ:tr|B9F4T2|B9F4T2_ORYSJ:tr|I1NZ08|I1NZ08_ORYGL:tr|A0A0E0NCF1|A0A0E0NCF1_ORYRU:tr|B8AEZ5|B8AEZ5_ORYSI:tr|T1T6C4|T1T6C4_ORYSI:tr|A1YQH6|A1YQH6_ORYSJ:tr|A0A0E0CIL1|A0A0E0CIL1_9ORYZ:tr|T1T4F0|T1T4F0_ORYSI:tr|I1NZ10|I1NZ10_ORYGL:tr|A0A0D3F336|A0A0D3F336_9ORYZ:tr|A0A0D9YPX0|A0A0D9YPX0_9ORYZ:tr|A0A0E0G6R1|A0A0E0G6R1_ORYNI:tr|A0A0E0CIL2|A0A0E0CIL2_9ORYZ:tr|A0A0D3F334|A0A0D3F334_9ORYZ
HGAFTPR	41.94	784.3980	7	393.2070	5.45	Glutelin	tr|B9F4T3|B9F4T3_ORYSJ:tr|Q0E2D2|Q0E2D2_ORYSJ:P14323|GLUB1_ORYSJ:tr|A0A0E0NCF3|A0A0E0NCF3_ORYRU:tr|B9F4T1|B9F4T1_ORYSJ:tr|A1YQH5|A1YQH5_ORYSJ:tr|Q0E2D5|Q0E2D5_ORYSJ:Q02897|GLUB2_ORYSJ:tr|A1YQH4|A1YQH4_ORYSJ:tr|B9F4T2|B9F4T2_ORYSJ:tr|I1NZ08|I1NZ08_ORYGL:tr|A0A0E0NCF1|A0A0E0NCF1_ORYRU:tr|B8AEZ5|B8AEZ5_ORYSI:tr|T1T6C4|T1T6C4_ORYSI:tr|A1YQH6|A1YQH6_ORYSJ:tr|A0A0E0CIL1|A0A0E0CIL1_9ORYZ:tr|T1T4F0|T1T4F0_ORYSI:tr|I1NZ10|I1NZ10_ORYGL:tr|A0A0D3F336|A0A0D3F336_9ORYZ:tr|A0A0D9YPX0|A0A0D9YPX0_9ORYZ:tr|A0A0E0G6R1|A0A0E0G6R1_ORYNI:tr|A0A0E0CIL2|A0A0E0CIL2_9ORYZ:tr|A0A0D3F334|A0A0D3F334_9ORYZ
NLNNNPYFKG	41.50	1179.5673	10	590.7914	13.81	Granule-bound starch synthase I	tr|A0A0H4BM25|A0A0H4BM25_ORYSI:tr|A0A0E0DW79|A0A0E0DW79_9ORYZ:tr|A8QXE7|A8QXE7_ORYSI:tr|V5NEJ7|V5NEJ7_ORYSA:tr|A0A0E0DW80|A0A0E0DW80_9ORYZ:tr|A0EQH2|A0EQH2_ORYSJ:tr|A0EQK4|A0EQK4_ORYRU:tr|A0EQK6|A0EQK6_ORYRU:tr|A0EQK5|A0EQK5_ORYRU:tr|A0EQD4|A0EQD4_ORYSI:tr|A0EQK7|A0EQK7_ORYRU:tr|A0EQE0|A0EQE0_ORYSA:P0C585|SSG1_ORYSA:tr|C8CBL4|C8CBL4_ORYSJ:tr|C8CBL1|C8CBL1_ORYSJ:tr|B1B5Z1|B1B5Z1_ORYSI:tr|B1B5Z0|B1B5Z0_ORYSI:A2Y8X2|SSG1_ORYSI:Q42968|SSG1_ORYGL:tr|A0A076FRI5|A0A076FRI5_ORYSJ:tr|B8XEK2|B8XEK2_ORYSA:tr|B8XEJ2|B8XEJ2_ORYSA:tr|A0A3Q9T3Z7|A0A3Q9T3Z7_ORYSA:tr|B8XEJ7|B8XEJ7_ORYSA:tr|D3U2H9|D3U2H9_ORYSA:tr|A0A3Q9T378|A0A3Q9T378_ORYSA:tr|D0TZY6|D0TZY6_ORYSI:tr|B8XEK3|B8XEK3_ORYSA:tr|B8XEJ8|B8XEJ8_ORYSA:tr|A0A0D9WLF6|A0A0D9WLF6_9ORYZ:tr|A0A0E0A4K1|A0A0E0A4K1_9ORYZ
NPSTNPWHSPRQG	41.08	1476.6858	13	739.3515	6.74	Glutelin	tr|B9F4T3|B9F4T3_ORYSJ:tr|Q0E2D2|Q0E2D2_ORYSJ:P14323|GLUB1_ORYSJ:tr|A0A0E0NCF3|A0A0E0NCF3_ORYRU:tr|B9F4T1|B9F4T1_ORYSJ:tr|A1YQH5|A1YQH5_ORYSJ:tr|Q0E2D5|Q0E2D5_ORYSJ:Q02897|GLUB2_ORYSJ:tr|A1YQH4|A1YQH4_ORYSJ:tr|B9F4T2|B9F4T2_ORYSJ:tr|I1NZ08|I1NZ08_ORYGL:tr|A0A0E0NCF1|A0A0E0NCF1_ORYRU:tr|B8AEZ5|B8AEZ5_ORYSI:tr|T1T6C4|T1T6C4_ORYSI:tr|A1YQH6|A1YQH6_ORYSJ:tr|A0A0E0CIL1|A0A0E0CIL1_9ORYZ:tr|T1T4F0|T1T4F0_ORYSI:tr|I1NZ10|I1NZ10_ORYGL:tr|A0A0D3F336|A0A0D3F336_9ORYZ:tr|A0A0D9YPX0|A0A0D9YPX0_9ORYZ:tr|A0A0E0G6R1|A0A0E0G6R1_ORYNI:tr|A0A0E0CIL2|A0A0E0CIL2_9ORYZ:tr|A0A0D3F334|A0A0D3F334_9ORYZ

<table>
<tr><td>H(+26.02)GAFTPR</td><td>39.95</td><td>810.4136</td><td>7</td><td>406.2139</td><td>7.04</td><td>Glutelin</td><td>tr|B9F4T3|B9F4T3_ORYSJ:tr|Q0E2D2|Q0E2D2_ORYSJ:P14323|GLUB1_ORYSJ:tr|A0A0E0NCF3|A0A0E0NCF3_ORYRU:tr|B9F4T1|B9F4T1_ORYSJ:tr|A1YQH5|A1YQH5_ORYSJ:tr|Q0E2D5|Q0E2D5_ORYSJ:Q02897|GLUB2_ORYSJ:tr|A1YQH4|A1YQH4_ORYSJ:tr|B9F4T2|B9F4T2_ORYSJ:tr|I1NZ08|I1NZ08_ORYGL:tr|A0A0E0NCF1|A0A0E0NCF1_ORYRU:tr|B8AEZ5|B8AEZ5_ORYSI:tr|T1T6C4|T1T6C4_ORYSI:tr|A1YQH6|A1YQH6_ORYSJ:tr|A0A0E0CIL1|A0A0E0CIL1_9ORYZ:tr|T1T4F0|T1T4F0_ORYSI:tr|I1NZ10|I1NZ10_ORYGL:tr|A0A0D3F336|A0A0D3F336_9ORYZ:tr|A0A0D9YPX0|A0A0D9YPX0_9ORYZ:tr|A0A0E0G6R1|A0A0E0G6R1_ORYNI:tr|A0A0E0CIL2|A0A0E0CIL2_9ORYZ:tr|A0A0D3F334|A0A0D3F334_9ORYZ</td></tr>
<tr><td>VFDGVLRPG</td><td>37.85</td><td>958.5236</td><td>9</td><td>480.2682</td><td>17.44</td><td>Glutelin</td><td>tr|B9F4T3|B9F4T3_ORYSJ:tr|Q0E2D2|Q0E2D2_ORYSJ:P14323|GLUB1_ORYSJ:tr|A0A0E0NCF3|A0A0E0NCF3_ORYRU:tr|B9F4T1|B9F4T1_ORYSJ:tr|A1YQH5|A1YQH5_ORYSJ:tr|Q0E2D5|Q0E2D5_ORYSJ:Q02897|GLUB2_ORYSJ:tr|A1YQH4|A1YQH4_ORYSJ:tr|B9F4T2|B9F4T2_ORYSJ:tr|I1NZ08|I1NZ08_ORYGL:tr|A0A0E0NCF1|A0A0E0NCF1_ORYRU:tr|B8AEZ5|B8AEZ5_ORYSI:tr|T1T6C4|T1T6C4_ORYSI:tr|A1YQH6|A1YQH6_ORYSJ:tr|A0A0E0CIL1|A0A0E0CIL1_9ORYZ:tr|T1T4F0|T1T4F0_ORYSI:tr|I1NZ10|I1NZ10_ORYGL:tr|A0A0D3F336|A0A0D3F336_9ORYZ:tr|A0A0D9YPX0|A0A0D9YPX0_9ORYZ:tr|A0A0E0G6R1|A0A0E0G6R1_ORYNI:tr|A0A0E0CIL2|A0A0E0CIL2_9ORYZ:tr|A0A0D3F334|A0A0D3F334_9ORYZ</td></tr>
<tr><td>HGAFTPRF</td><td>37.54</td><td>931.4664</td><td>8</td><td>466.7402</td><td>12.55</td><td>Glutelin</td><td>tr|B9F4T3|B9F4T3_ORYSJ:tr|Q0E2D2|Q0E2D2_ORYSJ:P14323|GLUB1_ORYSJ:tr|A0A0E0NCF3|A0A0E0NCF3_ORYRU:tr|B9F4T1|B9F4T1_ORYSJ:tr|A1YQH5|A1YQH5_ORYSJ:tr|Q0E2D5|Q0E2D5_ORYSJ:Q02897|GLUB2_ORYSJ:tr|A1YQH4|A1YQH4_ORYSJ:tr|B9F4T2|B9F4T2_ORYSJ:tr|I1NZ08|I1NZ08_ORYGL:tr|A0A0E0NCF1|A0A0E0NCF1_ORYRU:tr|B8AEZ5|B8AEZ5_ORYSI:tr|T1T6C4|T1T6C4_ORYSI:tr|A1YQH6|A1YQH6_ORYSJ:tr|A0A0E0CIL1|A0A0E0CIL1_9ORYZ:tr|T1T4F0|T1T4F0_ORYSI:tr|I1NZ10|I1NZ10_ORYGL:tr|A0A0D3F336|A0A0D3F336_9ORYZ:tr|A0A0D9YPX0|A0A0D9YPX0_9ORYZ:tr|A0A0E0G6R1|A0A0E0G6R1_ORYNI:tr|A0A0E0CIL2|A0A0E0CIL2_9ORYZ:tr|A0A0D3F334|A0A0D3F334_9ORYZ</td></tr>
<tr><td>SPFRVPIA</td><td>36.52</td><td>885.5072</td><td>8</td><td>443.7614</td><td>20.01</td><td>Pyruvate, phosphate dikinase</td><td>tr|J3M716|J3M716_ORYBR:tr|B9FPJ4|B9FPJ4_ORYSJ:tr|A0A0E0PMB6|A0A0E0PMB6_ORYRU:tr|B8AYC1|B8AYC1_ORYSI:tr|A0A0E0DR93|A0A0E0DR93_9ORYZ:Q6AVA8-2|PPDK1_ORYSJ:tr|A0A0D9ZZ05|A0A0D9ZZ05_9ORYZ:tr|A0A0D9VU47|A0A0D9VU47_9ORYZ:tr|J3LQ10|J3LQ10_ORYBR:tr|A2XIA2|A2XIA2_ORYSI:tr|I1PCI9|I1PCI9_ORYGL:tr|A0A0D3FK58|A0A0D3FK58_9ORYZ:tr|A0A0E0NWX2|A0A0E0NWX2_ORYRU:Q75KR1|PPDK2_ORYSJ:tr|A0A0D9Z998|A0A0D9Z998_9ORYZ:tr|O82032|O82032_ORYSI:tr|A0A0D3G7H5|A0A0D3G7H5_9ORYZ:tr|A0A0D9WGC7|A0A0D9WGC7_9ORYZ:tr|A0A0D9WGC6|A0A0D9WGC6_9ORYZ:Q6AVA8|PPDK1_ORYSJ:tr|I1PVJ3|I1PVJ3_ORYGL:tr|A0A0E0ITX7|A0A0E0ITX7_ORYNI:tr|A0A0E0ITX8|A0A0E0ITX8_ORYNI</td></tr>
</table>

YVGANPRL	34.34	888.4817	8	445.2480	11.24	Os04g0404400 protein	tr|Q0JDG9|Q0JDG9_ORYSJ:tr|A0A0P0WA63|A0A0P0WA63_ORYSJ:tr|A0A0E0H0B3|A0A0E0H0B3_ORYNI:tr|A0A0D9ZKB8|A0A0D9ZKB8_9ORYZ:tr|I1PKX3|I1PKX3_ORYGL:tr|Q01L47|Q01L47_ORYSA:tr|Q7X6I8|Q7X6I8_ORYSJ:tr|A2XT28|A2XT28_ORYSI:tr|A0A0D3FVG1|A0A0D3FVG1_9ORYZ:tr|A0A0E0P8P0|A0A0E0P8P0_ORYRU
APIYTQPR	33.32	944.5079	8	473.2621	7.57	glucose-1-phosphate adenylyltransferase	tr|D4AIA3|D4AIA3_ORYSI:tr|B7EVB8|B7EVB8_ORYSJ:tr|A0A0E0QHR8|A0A0E0QHR8_ORYRU:tr|A0A0E0H7V9|A0A0E0H7V9_ORYNI:tr|A0A0D3GZB2|A0A0D3GZB2_9ORYZ:tr|B8XEE2|B8XEE2_ORYSJ:tr|B8XEE1|B8XEE1_ORYSI:tr|B8XEE9|B8XEE9_ORYSI:tr|B8XEF2|B8XEF2_ORYSI:tr|B8XEF0|B8XEF0_ORYSI:tr|B8XEE8|B8XEE8_ORYSA:tr|B8XED7|B8XED7_ORYSI:tr|B8XED9|B8XED9_ORYSI:tr|B8XED8|B8XED8_ORYSA:tr|B8XEE6|B8XEE6_ORYSA:tr|B8XEE5|B8XEE5_ORYSJ:tr|A2YU91|A2YU91_ORYSI:tr|A0A0D3GZB1|A0A0D3GZB1_9ORYZ:tr|A0A0E0H7V8|A0A0E0H7V8_ORYNI:tr|A0A0E0QHR7|A0A0E0QHR7_ORYRU:tr|A0A0E0LU24|A0A0E0LU24_ORYPU:tr|A0A0D9X6Z4|A0A0D9X6Z4_9ORYZ:tr|A0A0D9X6Z3|A0A0D9X6Z3_9ORYZ:tr|A0A0E0LU23|A0A0E0LU23_ORYPU
FGKAPGVPH	33.25	908.4868	9	455.2508	6.90	Cytoplasmic ribosomal protein L18	tr|A0A0D3G3E7|A0A0D3G3E7_9ORYZ:tr|A0A0E0PHR1|A0A0E0PHR1_ORYRU:tr|A0A0E0H9T4|A0A0E0H9T4_ORYNI:tr|A0A0D9ZUF2|A0A0D9ZUF2_9ORYZ:tr|I1PSL1|I1PSL1_ORYGL:tr|J3M436|J3M436_ORYBR:tr|Q5WMY3|Q5WMY3_ORYSJ:tr|A0A0E0DM93|A0A0E0DM93_9ORYZ:tr|A0A0D9WD00|A0A0D9WD00_9ORYZ:tr|A0A0E0KYN0|A0A0E0KYN0_ORYPU:tr|A2Y0K0|A2Y0K0_ORYSI
FGPSQPFKGA	32.75	1034.5184	10	518.2677	14.80	Adenosylhomocysteinase	tr|H2KW56|H2KW56_ORYSJ:tr|Q2R4Y8|Q2R4Y8_ORYSJ:tr|J3N863|J3N863_ORYBR:tr|A0A0D3HLJ3|A0A0D3HLJ3_9ORYZ:tr|Q84VE1|Q84VE1_ORYSJ:tr|I1QZZ3|I1QZZ3_ORYGL:tr|A0A0E0MF96|A0A0E0MF96_ORYPU:tr|A0A0E0R823|A0A0E0R823_ORYRU:tr|A0A0E0J1P0|A0A0E0J1P0_ORYNI:tr|A2ZDY4|A2ZDY4_ORYSI:tr|A0A0E0BIU7|A0A0E0BIU7_9ORYZ:tr|Q0ISV7|Q0ISV7_ORYSJ:tr|A0A0P0Y1Y5|A0A0P0Y1Y5_ORYSJ
SDKGRFF	31.89	855.4238	7	428.7188	10.01	Os02g0519900 protein	tr|A0A0P0VJL8|A0A0P0VJL8_ORYSJ:tr|A0A0P0W6A1|A0A0P0W6A1_ORYSJ:tr|A0A0D9VGF7|A0A0D9VGF7_9ORYZ:tr|I1PIQ9|I1PIQ9_ORYGL:tr|Q6H4L2|Q6H4L2_ORYSJ:tr|Q7XTK1|Q7XTK1_ORYSJ:tr|A0A0E0G7F6|A0A0E0G7F6_ORYNI:tr|J3LVA1|J3LVA1_ORYBR:tr|A0A0E0P4M4|A0A0E0P4M4_ORYRU:tr|A0A0D3F5Q7|A0A0D3F5Q7_9ORYZ:tr|A0A0E0KM97|A0A0E0KM97_ORYPU:tr|J3LDA2|J3LDA2_ORYBR:tr|A0A0E0NFL3|A0A0E0NFL3_ORYRU:tr|A2X5F3|A2X5F3_ORYSI:tr|A0A0E0GX73|A0A0E0GX73_ORYNI:tr|A0A0D9ZGI8|A0A0D9ZGI8_9ORYZ:tr|I1P0T4|I1P0T4_ORYGL:tr|A0A0D9YSX8|A0A0D9YSX8_9ORYZ:tr|A0A0E0DCG7|A0A0E0DCG7_9ORYZ:tr|Q01MK8|Q01MK8_ORYSA:tr|A0A0D3FS13|A0A0D3FS13_9ORYZ:tr|A0A0E0DA54|A0A0E0DA54_9ORYZ:tr|A0A0E0DA53|A0A0E0DA53_9ORYZ:tr|A0A0E0DA52|A0A0E0DA52_9ORYZ
FNGVLRPG	29.88	858.4711	8	430.2426	12.62	Glutelin	Q6ERU3|GLUB5_ORYSJ:tr|A0A0E0JY90|A0A0E0JY90_ORYPU:tr|A0A0D3F3E6|A0A0D3F3E6_9ORYZ:tr|D6BV14|D6BV14_ORYSJ:tr|A0A0D9YQ79

							|A0A0D9YQ79_9ORYZ:tr|Q0E261|Q0E261_ORYSJ:P14614|GLUB4_ORYSJ:tr|A2X399|A2X399_ORYSI:tr|I1NZ94|I1NZ94_ORYGL:tr|A0A0D3F3E3|A0A0D3F3E3_9ORYZ:tr|A0A0D9VG85|A0A0D9VG85_9ORYZ:tr|A2X3A0|A2X3A0_ORYSI:tr|A0A0E0JY91|A0A0E0JY91_ORYPU:tr|A0A0D3F3E5|A0A0D3F3E5_9ORYZ:tr|C0L8H2|C0L8H2_ORYSJ:tr|A3A5D6|A3A5D6_ORYSJ:tr|A0A0E0G4Q4|A0A0E0G4Q4_ORYNI:tr|A0A0E0NCT0|A0A0E0NCT0_ORYRU:tr|M1G2E3|M1G2E3_ORYSI:tr|M1G571|M1G571_ORYSJ:tr|J3LBL3|J3LBL3_ORYBR:tr|Q0E2G5|Q0E2G5_ORYSJ:tr|C0L8H1|C0L8H1_ORYSJ:tr|A2X2V1|A2X2V1_ORYSI:tr|Q6ESW6|Q6ESW6_ORYSJ:tr|A0A0E0G459|A0A0E0G459_ORYNI:tr|Q84X93|Q84X93_ORYSJ:tr|A0A0E0NCA6|A0A0E0NCA6_ORYRU:tr|Q84X94|Q84X94_ORYSJ:tr|A0A0D9YLS5|A0A0D9YLS5_9ORYZ
FGWDKDLAKK	28.31	1206.6396	10	403.2210	11.50	Os02g0519900 protein	tr|A0A0P0VJL8|A0A0P0VJL8_ORYSJ:tr|A0A0P0W6A1|A0A0P0W6A1_ORYSJ:tr|A0A0D9VGF7|A0A0D9VGF7_9ORYZ:tr|I1PIQ9|I1PIQ9_ORYGL:tr|Q6H4L2|Q6H4L2_ORYSJ:tr|Q7XTK1|Q7XTK1_ORYSJ:tr|A0A0E0G7F6|A0A0E0G7F6_ORYNI:tr|J3LVA1|J3LVA1_ORYBR:tr|A0A0E0P4M4|A0A0E0P4M4_ORYRU:tr|A0A0D3F5Q7|A0A0D3F5Q7_9ORYZ:tr|A0A0E0KM97|A0A0E0KM97_ORYPU:tr|J3LDA2|J3LDA2_ORYBR:tr|A0A0E0NFL3|A0A0E0NFL3_ORYRU:tr|A2X5F3|A2X5F3_ORYSI:tr|A0A0E0GX73|A0A0E0GX73_ORYNI:tr|A0A0D9ZGI8|A0A0D9ZGI8_9ORYZ:tr|I1P0T4|I1P0T4_ORYGL:tr|A0A0D9YSX8|A0A0D9YSX8_9ORYZ:tr|A0A0E0DCG7|A0A0E0DCG7_9ORYZ:tr|Q01MK8|Q01MK8_ORYSA:tr|A0A0D3FS13|A0A0D3FS13_9ORYZ:tr|A0A0E0DA54|A0A0E0DA54_9ORYZ:tr|A0A0E0DA53|A0A0E0DA53_9ORYZ:tr|A0A0E0DA52|A0A0E0DA52_9ORYZ
SAPIYTQPRH	27.46	1168.5989	10	585.3062	6.11	glucose-1-phosphate adenylyltransferase	tr|D4AIA3|D4AIA3_ORYSI:tr|B7EVB8|B7EVB8_ORYSJ:tr|A0A0E0QHR8|A0A0E0QHR8_ORYRU:tr|A0A0E0H7V9|A0A0E0H7V9_ORYNI:tr|A0A0D3GZB2|A0A0D3GZB2_9ORYZ:tr|B8XEE2|B8XEE2_ORYSJ:tr|B8XEE1|B8XEE1_ORYSI:tr|B8XEE9|B8XEE9_ORYSI:tr|B8XEF2|B8XEF2_ORYSI:tr|B8XEF0|B8XEF0_ORYSI:tr|B8XEE8|B8XEE8_ORYSA:tr|B8XED7|B8XED7_ORYSI:tr|B8XED9|B8XED9_ORYSI:tr|B8XED8|B8XED8_ORYSA:tr|B8XEE6|B8XEE6_ORYSA:tr|B8XEE5|B8XEE5_ORYSJ:tr|A2YU91|A2YU91_ORYSI:tr|A0A0D3GZB1|A0A0D3GZB1_9ORYZ:tr|A0A0E0H7V8|A0A0E0H7V8_ORYNI:tr|A0A0E0QHR7|A0A0E0QHR7_ORYRU:tr|A0A0E0LU24|A0A0E0LU24_ORYPU:tr|A0A0D9X6Z4|A0A0D9X6Z4_9ORYZ:tr|A0A0D9X6Z3|A0A0D9X6Z3_9ORYZ:tr|A0A0E0LU23|A0A0E0LU23_ORYPU
VWFPQPAPK	26.18	1068.5756	9	535.2947	18.42	Formate dehydrogenase 1, mitochondrial	tr|A0A0E0LBQ9|A0A0E0LBQ9_ORYPU:Q9SXP2|FDH1_ORYSJ:Q67U69|FDH2_ORYSJ
KDKLWPM	26.06	916.4840	7	459.2496	16.79	Os02g0519900 protein	tr|A0A0P0VJL8|A0A0P0VJL8_ORYSJ:tr|A0A0P0W6A1|A0A0P0W6A1_ORYSJ:tr|A0A0D9VGF7|A0A0D9VGF7_9ORYZ:tr|I1PIQ9|I1PIQ9_ORYGL:tr|Q6H4L2|Q6H4L2_ORYSJ:tr|Q7XTK1|Q7XTK1_ORYSJ:tr|A0A0E0G7F6|A

							0A0E0G7F6_ORYNI:tr|J3LVA1|J3LVA1_ORYBR:tr|A0A0E0P4M4|A0A0E0P4M4_ORYRU:tr|A0A0D3F5Q7|A0A0D3F5Q7_9ORYZ:tr|A0A0E0KM97|A0A0E0KM97_ORYPU:tr|J3LDA2|J3LDA2_ORYBR:tr|A0A0E0NFL3|A0A0E0NFL3_ORYRU:tr|A2X5F3|A2X5F3_ORYSI:tr|A0A0E0GX73|A0A0E0GX73_ORYNI:tr|A0A0D9ZGI8|A0A0D9ZGI8_9ORYZ:tr|I1P0T4|I1P0T4_ORYGL:tr|A0A0D9YSX8|A0A0D9YSX8_9ORYZ:tr|A0A0E0DCG7|A0A0E0DCG7_9ORYZ:tr|Q01MK8|Q01MK8_ORYSA:tr|A0A0D3FS13|A0A0D3FS13_9ORYZ:tr|A0A0E0DA54|A0A0E0DA54_9ORYZ:tr|A0A0E0DA53|A0A0E0DA53_9ORYZ:tr|A0A0E0DA52|A0A0E0DA52_9ORYZ
NLNNNPYFK	24.61	1122.5458	9	562.2805	13.64	Granule-bound starch synthase I	tr|A0A0H4BM25|A0A0H4BM25_ORYSI:tr|A0A0E0DW79|A0A0E0DW79_9ORYZ:tr|A8QXE7|A8QXE7_ORYSI:tr|V5NEJ7|V5NEJ7_ORYSA:tr|A0A0E0DW80|A0A0E0DW80_9ORYZ:tr|A0EQH2|A0EQH2_ORYSJ:tr|A0EQK4|A0EQK4_ORYRU:tr|A0EQK6|A0EQK6_ORYRU:tr|A0EQK5|A0EQK5_ORYRU:tr|A0EQD4|A0EQD4_ORYSI:tr|A0EQK7|A0EQK7_ORYRU:tr|A0EQE0|A0EQE0_ORYSA:P0C585|SSG1_ORYSA:tr|C8CBL4|C8CBL4_ORYSJ:tr|C8CBL1|C8CBL1_ORYSJ:tr|B1B5Z1|B1B5Z1_ORYSI:tr|B1B5Z0|B1B5Z0_ORYSI:A2Y8X2|SSG1_ORYSI:Q42968|SSG1_ORYGL:tr|A0A076FRI5|A0A076FRI5_ORYSJ:tr|B8XEK2|B8XEK2_ORYSA:tr|B8XEJ2|B8XEJ2_ORYSA:tr|A0A3Q9T3Z7|A0A3Q9T3Z7_ORYSA:tr|B8XEJ7|B8XEJ7_ORYSA:tr|D3U2H9|D3U2H9_ORYSA:tr|A0A3Q9T378|A0A3Q9T378_ORYSA:tr|D0TZY6|D0TZY6_ORYSI:tr|B8XEK3|B8XEK3_ORYSA:tr|B8XEJ8|B8XEJ8_ORYSA:tr|A0A0D9WLF6|A0A0D9WLF6_9ORYZ:tr|A0A0E0A4K1|A0A0E0A4K1_9ORYZ
NNNPYFK	24.28	895.4188	7	448.7165	8.69	Granule-bound starch synthase I	tr|A0A0H4BM25|A0A0H4BM25_ORYSI:tr|A0A0E0DW79|A0A0E0DW79_9ORYZ:tr|A8QXE7|A8QXE7_ORYSI:tr|V5NEJ7|V5NEJ7_ORYSA:tr|A0A0E0DW80|A0A0E0DW80_9ORYZ:tr|A0EQH2|A0EQH2_ORYSJ:tr|A0EQK4|A0EQK4_ORYRU:tr|A0EQK6|A0EQK6_ORYRU:tr|A0EQK5|A0EQK5_ORYRU:tr|A0EQD4|A0EQD4_ORYSI:tr|A0EQK7|A0EQK7_ORYRU:tr|A0EQE0|A0EQE0_ORYSA:P0C585|SSG1_ORYSA:tr|C8CBL4|C8CBL4_ORYSJ:tr|C8CBL1|C8CBL1_ORYSJ:tr|B1B5Z1|B1B5Z1_ORYSI:tr|B1B5Z0|B1B5Z0_ORYSI:A2Y8X2|SSG1_ORYSI:Q42968|SSG1_ORYGL:tr|A0A076FRI5|A0A076FRI5_ORYSJ:tr|B8XEK2|B8XEK2_ORYSA:tr|B8XEJ2|B8XEJ2_ORYSA:tr|A0A3Q9T3Z7|A0A3Q9T3Z7_ORYSA:tr|B8XEJ7|B8XEJ7_ORYSA:tr|D3U2H9|D3U2H9_ORYSA:tr|A0A3Q9T378|A0A3Q9T378_ORYSA:tr|D0TZY6|D0TZY6_ORYSI:tr|B8XEK3|B8XEK3_ORYSA:tr|B8XEJ8|B8XEJ8_ORYSA:tr|A0A0D9WLF6|A0A0D9WLF6_9ORYZ:tr|A0A0E0A4K1|A0A0E0A4K1_9ORYZ
IPPPRGPLW	23.83	1031.5916	9	516.8033	20.94	Aspartic protease	tr|Q93XE4|Q93XE4_ORYSJ:tr|A0A0P0WR25|A0A0P0WR25_ORYSJ:tr|A0A0E0D6E9|A0A0E0D6E9_9ORYZ:tr|A0A0E0KY87|A0A0E0KY87_ORYPU:P42211|ASPRX_ORYSJ:tr|A0A0D3G2Z6|A0A0D3G2Z6_9ORYZ:tr|A0A0E0H931|A0A0E0H931_ORYNI:tr|A0A0E0PH77|A0A0E0PH77_ORYRU:tr|A0A0D9WCH7|A0A0D9WCH7_9ORYZ:tr|B9FMC0|B9FMC0_ORYSJ:tr|A0A0E0C5N5|A0A0E0C5N5_9ORYZ:tr|A0A0E0L5U7|A0A0E0L5U7_ORYPU:tr|Q0DFW1|Q0DFW1_ORYSJ:Q42456|ASPR1_ORYSJ:tr|A0A0E0A2X2|A0A

							0E0A2X2_9ORYZ:tr|A0A0E0PRC6|A0A0E0PRC6_ORYRU:tr|A2Y7J9|A2Y7J9_ORYSI:tr|A0A0E0HIK1|A0A0E0HIK1_ORYNI:tr|A0A0D3GAX9|A0A0D3GAX9_9ORYZ:tr|A0A0E0D6E6|A0A0E0D6E6_9ORYZ:tr|A0A0D9WJU6|A0A0D9WJU6_9ORYZ:tr|A0A0E0JM18|A0A0E0JM18_ORYPU:tr|A0A0E0C5N4|A0A0E0C5N4_9ORYZ:tr|A0A0E0N0C1|A0A0E0N0C1_ORYRU:tr|Q0JKM8|Q0JKM8_ORYSJ:tr|B8A762|B8A762_ORYSI:tr|A0A0E0D6E8|A0A0E0D6E8_9ORYZ:tr|A0A0E0PRC4|A0A0E0PRC4_ORYRU:tr|A0A0E0L5U6|A0A0E0L5U6_ORYPU
ARVAMTI	21.20	760.4265	7	381.2189	12.92	Uncharacterized protein	tr|A0A0E0L762|A0A0E0L762_ORYPU
FDGVLRPG	20.74	859.4551	8	430.7377	14.07	Glutelin	tr|B9F4T3|B9F4T3_ORYSJ:tr|Q0E2D2|Q0E2D2_ORYSJ:P14323|GLUB1_ORYSJ:tr|A0A0E0NCF3|A0A0E0NCF3_ORYRU:tr|B9F4T1|B9F4T1_ORYSJ:tr|A1YQH5|A1YQH5_ORYSJ:tr|Q0E2D5|Q0E2D5_ORYSJ:Q02897|GLUB2_ORYSJ:tr|A1YQH4|A1YQH4_ORYSJ:tr|B9F4T2|B9F4T2_ORYSJ:tr|I1NZ08|I1NZ08_ORYGL:tr|A0A0E0NCF1|A0A0E0NCF1_ORYRU:tr|B8AEZ5|B8AEZ5_ORYSI:tr|T1T6C4|T1T6C4_ORYSI:tr|A1YQH6|A1YQH6_ORYSJ:tr|A0A0E0CIL1|A0A0E0CIL1_9ORYZ:tr|T1T4F0|T1T4F0_ORYSI:tr|I1NZ10|I1NZ10_ORYGL:tr|A0A0D3F336|A0A0D3F336_9ORYZ:tr|A0A0D9YPX0|A0A0D9YPX0_9ORYZ:tr|A0A0E0G6R1|A0A0E0G6R1_ORYNI:tr|A0A0E0CIL2|A0A0E0CIL2_9ORYZ:tr|A0A0D3F334|A0A0D3F334_9ORYZ

Sub-fraction n. 13. Total: 49 identified peptides, all with rice protein accession. RT, retention time.

Peptide	-10lgP	Mass (Da)	Length (amino acid n.)	m/z	RT (min)	Accession	Protein
NPSTNPWHSPR	54.08	1291.6057	11	646.8109	7.36	Uncharacterized protein	tr|A0A0D9VE94|A0A0D9VE94_9ORYZ:tr|A2X2Z8|A2X2Z8_ORYSI
KKPVPDFSFY	51.04	1226.6335	10	614.3240	19.13	Glucose-1-phosphate adenylyltransferase	tr|A0A0E0LZH2|A0A0E0LZH2_ORYPU:tr|D0TZC6|D0TZC6_ORYSJ:tr|A0A0E0EQI0|A0A0E0EQI0_9ORYZ:tr|B8BE16|B8BE16_ORYSI:tr|A0A0E0B114|A0A0E0B114_9ORYZ:tr|A0A0D3H4W0|A0A0D3H4W0_9ORYZ:tr|A7IZE4|A7IZE4_ORYSI:tr|D4AIA3|D4AIA3_ORYSI:tr|B7EVB8|B7EVB8_ORYSJ:tr|A0A0D3GZB2|A0A0D3GZB2_9ORYZ:tr|A0A0E0H7V9|A0A0E0H7V9_ORYNI:tr|A0A0E0QHR8|A0A0E0QHR8_ORYRU:tr|B8XED8|B8XED8_ORYSA:tr|B8XED9|B8XED9_ORYSI:tr|B8XED7|B8XED7_ORYSI:tr|B8XEE8|B8XEE8_ORYSA:tr|B8XEF0|B8XEF0_ORYSI:tr|B8XEF2|B8XEF2_ORYSI:tr|B8XEE9|B8XEE9_ORYSI:tr|B8XEE1|B8XEE1_ORYSI:tr|B8XEE2|B8XEE2_ORYSJ:tr|Q9ARH9|Q9ARH9_ORYSA:tr|D3U2H7|D3U2H7_ORYSA:tr|D0TZC9|D0TZC9_ORYSI:Q69T99|GLGS1_ORYSJ:tr|B8XEE5|B8XEE5_O

<table>
<tr><td></td><td></td><td></td><td></td><td></td><td></td><td></td><td>RYSJ:tr|B8XEE6|B8XEE6_ORYSA:tr|A2YU91|A2YU91_ORYSI:tr|I1QHZ2|I1QHZ2_ORYGL:tr|A0A0D3GZB1|A0A0D3GZB1_9ORYZ:tr|A0A0E0QHR7|A0A0E0QHR7_ORYRU</td></tr>
<tr><td>TPIQYKSY</td><td>50.14</td><td>998.5073</td><td>8</td><td>500.2612</td><td>8.93</td><td>Glutelin</td><td>tr|A0A0E0M7E9|A0A0E0M7E9_ORYPU:tr|T1T4G3|T1T4G3_ORYSI:tr|A0A0E0JP14|A0A0E0JP14_ORYPU:tr|A1YQG5|A1YQG5_ORYSJ:tr|I1NRU9|I1NRU9_ORYGL:tr|A0A0E0N2T5|A0A0E0N2T5_ORYRU:tr|A2WVB9|A2WVB9_ORYSI:P07728|GLUA1_ORYSJ:tr|A0A0E0M7E8|A0A0E0M7E8_ORYPU:tr|A0A0E0FTI2|A0A0E0FTI2_ORYNI:tr|A0A0D9YFB1|A0A0D9YFB1_9ORYZ:tr|A0A0E0C821|A0A0E0C821_9ORYZ:tr|A0A0D3EUB5|A0A0D3EUB5_9ORYZ:tr|Q0JJ36|Q0JJ36_ORYSJ:tr|Q40689|Q40689_ORYSA</td></tr>
<tr><td>TPLQYKSY</td><td>50.14</td><td>998.5073</td><td>8</td><td>500.2612</td><td>8.93</td><td>Glutelin</td><td>tr|T1T4Y4|T1T4Y4_ORYSI:tr|A1YQG3|A1YQG3_ORYSJ:P07730|GLUA2_ORYSJ:tr|A2Z708|A2Z708_ORYSI:tr|I1QU95|I1QU95_ORYGL:tr|A0A0E0BA65|A0A0E0BA65_9ORYZ:tr|A0A0E0IRV3|A0A0E0IRV3_ORYNI:tr|A0A0D3HDD6|A0A0D3HDD6_9ORYZ:tr|A0A0E0QYR7|A0A0E0QYR7_ORYRU:tr|A0A0E0EXG9|A0A0E0EXG9_9ORYZ:tr|A0A0E0BA63|A0A0E0BA63_9ORYZ:tr|A0A0E0BA64|A0A0E0BA64_9ORYZ:tr|A0A0E0IRV1|A0A0E0IRV1_ORYNI:tr|A0A0E0QYR5|A0A0E0QYR5_ORYRU:tr|A0A0E0EXG8|A0A0E0EXG8_9ORYZ:tr|A0A0E0QYR6|A0A0E0QYR6_ORYRU:tr|A0A0D3HDD5|A0A0D3HDD5_9ORYZ:tr|A0A0E0IRV2|A0A0E0IRV2_ORYNI</td></tr>
<tr><td>VVVGTPGRVF</td><td>46.08</td><td>1029.5970</td><td>10</td><td>515.8058</td><td>17.93</td><td>Eukaryotic initiation factor 4A-2</td><td>P41377|IF4A2_ARATH:tr|A0A0D9WTS3|A0A0D9WTS3_9ORYZ:tr|A0A0D9VC68|A0A0D9VC68_9ORYZ:tr|A0A0N7KMN9|A0A0N7KMN9_ORYSJ:P41376|IF4A1_ARATH:tr|A0A0E0N9T3|A0A0E0N9T3_ORYRU:P35683|IF4A1_ORYSJ:Q6Z2Z4|IF4A3_ORYSJ:tr|J3MHA8|J3MHA8_ORYBR:tr|I1NX84|I1NX84_ORYGL:tr|J3L9J6|J3L9J6_ORYBR:Q9CAI7|IF4A3_ARATH:tr|A0A0E0Q284|A0A0E0Q284_ORYRU:tr|A0A0E0LFA0|A0A0E0LFA0_ORYPU:tr|I1Q4Z1|I1Q4Z1_ORYGL:tr|A2X0V4|A2X0V4_ORYSI:tr|A0A0D9YM91|A0A0D9YM91_9ORYZ:tr|A0A0E0JVG9|A0A0E0JVG9_ORYPU:tr|A0A0D3GKG6|A0A0D3GKG6_9ORYZ:tr|A0A0E0G162|A0A0E0G162_ORYNI:tr|A0A0E0CFK9|A0A0E0CFK9_9ORYZ:tr|A0A0E0ADY5|A0A0E0ADY5_9ORYZ:tr|A0A0E0HUY2|A0A0E0HUY2_ORYNI:tr|A0A0E0HUY1|A0A0E0HUY1_ORYNI:tr|A2YGP5|A2YGP5_ORYSI:tr|A0A0D3F0N3|A0A0D3F0N3_9ORYZ:tr|A0A0E0HUY0|A0A0E0HUY0_ORYNI:tr|A0A0D3F0N2|A0A0D3F0N2_9ORYZ</td></tr>
<tr><td>FGWDKDLAKK</td><td>44.45</td><td>1206.6396</td><td>10</td><td>403.2210</td><td>11.60</td><td>Os02g0519900 protein</td><td>tr|A0A0P0VJQ0|A0A0P0VJQ0_ORYSJ:tr|A0A0P0VJN8|A0A0P0VJN8_ORYSJ:tr|A0A0N7KIH5|A0A0N7KIH5_ORYSJ:tr|A0A0E0JIH9|A0A0E0JIH9_ORYPU:tr|A0A0E0DZ38|A0A0E0DZ38_9ORYZ:tr|A0A0P0VJL8|A0A0P0VJL8_ORYSJ:tr|A0A0D9VGF7|A0A0D9VGF7_9ORYZ:tr|Q5JKU5|Q5JKU5_ORYSJ:tr|A0A0E0N296|A0A0E0N296_ORYRU:tr|A2WUX6|A2WUX6_ORYSI:tr|A0A0D9YES5|A0A0D9YES5_9ORYZ:tr|Q01MK8|Q01MK8_ORYSA:tr|A0A0E0DCG7|A0A0E0DCG7_9ORYZ:tr|A0A0D9ZGI8|A0A0D9ZGI8_9ORYZ:tr|A0A0E0GX73|A0A0E0GX73_ORYNI:tr|A2X5F3|A2X5F3_ORYSI:tr|A0A0E0KM97|A0A0E0KM97_ORYPU:tr|A0A0E0P4M4|A0A0E0P4M4_ORYRU:tr|Q7XTK1|Q7XTK1_ORYSJ:tr|Q6H4L2|Q6H4L2_ORYSJ:tr|A0A0D3ET</td></tr>
</table>

<table>
<tr><td></td><td></td><td></td><td></td><td></td><td></td><td></td><td>H6|A0A0D3ETH6_9ORYZ:tr|A2WUL5|A2WUL5_ORYSI:tr|A0A0E0C7D6|A0A0E0C7D6_9ORYZ:tr|A0A0E0N1V6|A0A0E0N1V6_ORYRU:tr|Q8W0C4|Q8W0C4_ORYSJ:tr|A0A0E0JNF3|A0A0E0JNF3_ORYPU:tr|A2ZXD6|A2ZXD6_ORYSJ:tr|A0A0E0DA54|A0A0E0DA54_9ORYZ:tr|A0A0E0DA52|A0A0E0DA52_9ORYZ:tr|A0A0E0DA53|A0A0E0DA53_9ORYZ</td></tr>
<tr><td>SLGQKPVTFF</td><td>41.42</td><td>1122.6073</td><td>10</td><td>562.3114</td><td>22.38</td><td>Os10g0390500 protein</td><td>tr|A0A0P0XUE4|A0A0P0XUE4_ORYSJ:tr|A0A0E0IRN2|A0A0E0IRN2_ORYNI:tr|A0A0D3HD77|A0A0D3HD77_9ORYZ:tr|Q9S768|Q9S768_ORYSA:tr|I1QU38|I1QU38_ORYGL:tr|B8BGM4|B8BGM4_ORYSI:tr|Q338N8|Q338N8_ORYSJ:tr|A0A0E0QYK7|A0A0E0QYK7_ORYRU:tr|A0A0D3HD76|A0A0D3HD76_9ORYZ:tr|Q94HC5|Q94HC5_ORYSJ:tr|A0A0E0IRN1|A0A0E0IRN1_ORYNI:tr|A0A0E0QYK6|A0A0E0QYK6_ORYRU:tr|A0A0E0B9Z8|A0A0E0B9Z8_9ORYZ:tr|A0A0E0B9Z7|A0A0E0B9Z7_9ORYZ:tr|A0A0E0B9Z9|A0A0E0B9Z9_9ORYZ</td></tr>
<tr><td>SWKGPAKNWE</td><td>41.32</td><td>1201.5880</td><td>10</td><td>601.8023</td><td>10.81</td><td>Starch synthase, chloroplastic/amyloplastic</td><td>tr|V5NEJ7|V5NEJ7_ORYSA:tr|A0EQH2|A0EQH2_ORYSJ:tr|A0EQK7|A0EQK7_ORYRU:tr|A0EQD4|A0EQD4_ORYSI:tr|A0EQK4|A0EQK4_ORYRU:tr|A0EQE0|A0EQE0_ORYSA:tr|A0EQK5|A0EQK5_ORYRU:tr|A0EQK6|A0EQK6_ORYRU:P0C585|SSG1_ORYSA:tr|B8XEK3|B8XEK3_ORYSA:tr|D0TZY6|D0TZY6_ORYSI:tr|A0A3Q9T378|A0A3Q9T378_ORYSA:tr|D3U2H9|D3U2H9_ORYSA:tr|B8XEJ7|B8XEJ7_ORYSA:tr|A0A3Q9T3Z7|A0A3Q9T3Z7_ORYSA:tr|B8XEK2|B8XEK2_ORYSA:tr|A0A076FRI5|A0A076FRI5_ORYSJ:Q42968|SSG1_ORYGL:A2Y8X2|SSG1_ORYSI:tr|C6ZGE5|C6ZGE5_ORYSI:tr|C8CBL1|C8CBL1_ORYSJ:tr|B8XEJ8|B8XEJ8_ORYSA:tr|A0A3T0P6Q9|A0A3T0P6Q9_ORYSA:tr|B1B5Z0|B1B5Z0_ORYSI:tr|B1B5Z1|B1B5Z1_ORYSI:tr|A0A0D9WLF6|A0A0D9WLF6_9ORYZ:tr|A0A0E0A4K1|A0A0E0A4K1_9ORYZ</td></tr>
<tr><td>VFNGVLRPG</td><td>40.52</td><td>957.5396</td><td>9</td><td>479.7774</td><td>15.87</td><td>Glutelin</td><td>tr|A2X399|A2X399_ORYSI:P14614|GLUB4_ORYSJ:tr|Q0E261|Q0E261_ORYSJ:tr|A0A0D9YQ79|A0A0D9YQ79_9ORYZ:tr|D6BV14|D6BV14_ORYSJ:tr|A0A0D3F3E6|A0A0D3F3E6_9ORYZ:tr|A0A0E0JY90|A0A0E0JY90_ORYPU:Q6ERU3|GLUB5_ORYSJ:tr|I1NZ94|I1NZ94_ORYGL:tr|A0A0D3F3E3|A0A0D3F3E3_9ORYZ</td></tr>
<tr><td>RADYYNPR</td><td>40.39</td><td>1053.4991</td><td>8</td><td>527.7564</td><td>5.66</td><td>Glutelin</td><td>tr|T1T4G5|T1T4G5_ORYSI:tr|M1G949|M1G949_ORYSI:Q6K508|GLUD1_ORYSJ:tr|I1NZ02|I1NZ02_ORYGL:tr|A2X2Z1|A2X2Z1_ORYSI:tr|A0A0D3F331|A0A0D3F331_9ORYZ:tr|A0A0E0FMV5|A0A0E0FMV5_ORYNI:tr|A0A0E0NCE4|A0A0E0NCE4_ORYRU:tr|A0A0E0JXZ6|A0A0E0JXZ6_ORYPU:tr|A0A0D9YPW1|A0A0D9YPW1_9ORYZ:tr|A0A0E0NCE6|A0A0E0NCE6_ORYRU:tr|A0A0E0CIK2|A0A0E0CIK2_9ORYZ</td></tr>
<tr><td>STNPWHSPRQG</td><td>37.89</td><td>1265.5901</td><td>11</td><td>633.8027</td><td>6.04</td><td>Uncharacterized protein</td><td>tr|A0A0D9VE94|A0A0D9VE94_9ORYZ:tr|A2X2Z8|A2X2Z8_ORYSI</td></tr>
<tr><td>RPPKPDAPRIY</td><td>37.78</td><td>1308.7302</td><td>11</td><td>437.2507</td><td>8.06</td><td>Starch branching enzyme 1</td><td>tr|A0A0E0E6K5|A0A0E0E6K5_9ORYZ:Q01401-2|GLGB_ORYSJ:tr|B7EAH2|B7EAH2_ORYSJ:tr|A0A2S0T044|A0A2S0T044_ORYSA:tr|A0A2S0T039|A0A2S0T039_ORYSA:tr|A0A0E0AEM0|A0A0E</td></tr>
</table>

							0AEM0_9ORYZ:tr|A0A0E0AEL9|A0A0E0AEL9_9ORYZ:tr|A0A2S0T029|A0A2S0T029_ORYSA:tr|Q0D9D0|Q0D9D0_ORYSJ:Q01401|GLGB_ORYSJ:tr|A0A2S0T020|A0A2S0T020_ORYSA:tr|D0TZI4|D0TZI4_ORYSI:tr|B8B2L2|B8B2L2_ORYSI:tr|A0A0E0E6K3|A0A0E0E6K3_9ORYZ:tr|A0A0E0LFU9|A0A0E0LFU9_ORYPU:tr|A0A0E0LFV0|A0A0E0LFV0_ORYPU:tr|A0A0E0LFU8|A0A0E0LFU8_ORYPU:tr|A0A0E0LFU7|A0A0E0LFU7_ORYPU:tr|A0A0E0Q2U7|A0A0E0Q2U7_ORYRU:tr|A0A0E0Q2U8|A0A0E0Q2U8_ORYRU:tr|A0A0E0Q2U9|A0A0E0Q2U9_ORYRU
SSKPFFGGL	37.78	938.4861	9	470.2505	21.89	Nucleoside diphosphate kinase	tr|A0A0E0IWK3|A0A0E0IWK3_ORYNI:tr|A2ZAA7|A2ZAA7_ORYSI:tr|A0A0E0R2J8|A0A0E0R2J8_ORYRU:tr|Q7XC37|Q7XC37_ORYSJ:tr|I1QW04|I1QW04_ORYGL:tr|A0A0D9XMI7|A0A0D9XMI7_9ORYZ:tr|A0A0E0EGM8|A0A0E0EGM8_9ORYZ:tr|A0A0E0BDU7|A0A0E0BDU7_9ORYZ:tr|A0A0D3HGP0|A0A0D3HGP0_9ORYZ:tr|A0A0E0MAU0|A0A0E0MAU0_ORYPU:tr|J3N4Y6|J3N4Y6_ORYBR:tr|A6N077|A6N077_ORYSI
LDWYKGPT	37.31	978.4811	8	490.2484	17.90	Elongation factor	tr|E7BJ60|E7BJ60_ORYSI:tr|A0A0E0NQG9|A0A0E0NQG9_ORYRU:tr|A0A0P0VTT8|A0A0P0VTT8_ORYSJ:tr|A0A0E0CW33|A0A0E0CW33_9ORYZ:tr|A0A0E0CW26|A0A0E0CW26_9ORYZ:tr|Q10QZ5|Q10QZ5_ORYSJ:tr|A0A0E0GHL8|A0A0E0GHL8_ORYNI:tr|A0A0N7KGP4|A0A0N7KGP4_ORYSJ:tr|I1P848|I1P848_ORYGL:tr|B9FBM7|B9FBM7_ORYSJ:tr|J3LKK4|J3LKK4_ORYBR:tr|A0A0E0K9J4|A0A0E0K9J4_ORYPU:tr|A0A1L2JKK1|A0A1L2JKK1_ORYSA:tr|Q10QZ6|Q10QZ6_ORYSJ:tr|A0A0D3FEJ3|A0A0D3FEJ3_9ORYZ:tr|A0A0D9VSS7|A0A0D9VSS7_9ORYZ:tr|A0A0D3FEJ0|A0A0D3FEJ0_9ORYZ:O64937|EF1A_ORYSJ:tr|A0A0E0GHL3|A0A0E0GHL3_ORYNI:tr|B8APM5|B8APM5_ORYSI:tr|I1P851|I1P851_ORYGL:tr|A0A0E0K9J0|A0A0E0K9J0_ORYPU:tr|Q10QZ4|Q10QZ4_ORYSJ:tr|A0A0D3FEJ2|A0A0D3FEJ2_9ORYZ:tr|I1P850|I1P850_ORYGL:tr|A0A0D3FEJ4|A0A0D3FEJ4_9ORYZ:tr|A0A0D9Z2R9|A0A0D9Z2R9_9ORYZ:tr|A0A0D3FEJ1|A0A0D3FEJ1_9ORYZ
IGRPAPMPY	37.22	1000.5164	9	501.2658	14.13	Os06g0676700 protein	tr|A0A0E0Q1L2|A0A0E0Q1L2_ORYRU:tr|A0A0E0LEN1|A0A0E0LEN1_ORYPU:tr|A0A0D3GJV3|A0A0D3GJV3_9ORYZ:tr|A0A0E0GD25|A0A0E0GD25_ORYNI:tr|A0A0E0E5A4|A0A0E0E5A4_9ORYZ:tr|A0A0E0Q1K4|A0A0E0Q1K4_ORYRU:tr|A0A0E0Q1K2|A0A0E0Q1K2_ORYRU:tr|A0A0E0AD77|A0A0E0AD77_9ORYZ:tr|A3BEL8|A3BEL8_ORYSJ:tr|A0A0E0Q1K3|A0A0E0Q1K3_ORYRU:tr|A0A0E0AD76|A0A0E0AD76_9ORYZ:tr|A0A0D3GJV2|A0A0D3GJV2_9ORYZ:Q653V7|AGLU_ORYSJ:tr|Q653V4|Q653V4_ORYSJ:tr|A2YG59|A2YG59_ORYSI:tr|A0A0E0AD89|A0A0E0AD89_9ORYZ:tr|B8B1F4|B8B1F4_ORYSI:tr|B8AD31|B8AD31_ORYSI:tr|Q9LGC6|Q9LGC6_ORYSJ:tr|A0A0D9UWE3|A0A0D9UWE3_9ORYZ:tr|A0A0E0BWI1|A0A0E0BWI1_9ORYZ:tr|Q0JQZ2|Q0JQZ2_ORYSJ:tr|A0A0E0MQY3|A0A0E0MQY3_ORYRU:tr|A0A0E0FFR9|A0A0E0FFR9_ORYNI:tr|A0A0D3EJ82|A0A0D3EJ82_9ORYZ:tr|A0A0E0JDN4|A0A0E0JDN4_ORYPU:tr|A0A0E0LEM2|A0A0E0LEM2_ORYPU:tr|A0A0E0AD80|A0A0E0AD80_9ORYZ:tr|A0A0E0GD31|A0A0E0GD31_ORYNI:tr|A0A0E0E598|A0A0E0E598_9ORYZ

<table>
<tr><td>VFDGVLRPG</td><td>36.94</td><td>958.5236</td><td>9</td><td>480.2693</td><td>16.61</td><td>Uncharacterized protein</td><td>tr|A0A0D9VE94|A0A0D9VE94_9ORYZ:tr|A2X2Z8|A2X2Z8_ORYSI:tr|A0A0E0JY02|A0A0E0JY02_ORYPU</td></tr>
<tr><td>NLNNNPYFK</td><td>36.57</td><td>1122.5458</td><td>9</td><td>562.2811</td><td>13.73</td><td>Starch synthase, chloroplastic/amyloplastic</td><td>tr|V5NEJ7|V5NEJ7_ORYSA:tr|A0EQH2|A0EQH2_ORYSJ:tr|A0EQK7|A0EQK7_ORYRU:tr|A0EQD4|A0EQD4_ORYSI:tr|A0EQK4|A0EQK4_ORYRU:tr|A0EQE0|A0EQE0_ORYSA:tr|A0EQK5|A0EQK5_ORYRU:tr|A0EQK6|A0EQK6_ORYRU:P0C585|SSG1_ORYSA:tr|B8XEK3|B8XEK3_ORYSA:tr|D0TZY6|D0TZY6_ORYSI:tr|A0A3Q9T378|A0A3Q9T378_ORYSA:tr|D3U2H9|D3U2H9_ORYSA:tr|B8XEJ7|B8XEJ7_ORYSA:tr|A0A3Q9T3Z7|A0A3Q9T3Z7_ORYSA:tr|B8XEK2|B8XEK2_ORYSA:tr|A0A076FRI5|A0A076FRI5_ORYSJ:Q42968|SSG1_ORYGL:A2Y8X2|SSG1_ORYSI:tr|C6ZGE5|C6ZGE5_ORYSI:tr|C8CBL1|C8CBL1_ORYSJ:tr|B8XEJ8|B8XEJ8_ORYSA:tr|A0A3T0P6Q9|A0A3T0P6Q9_ORYSA:tr|B1B5Z0|B1B5Z0_ORYSI:tr|B1B5Z1|B1B5Z1_ORYSI:tr|A0A0D9WLF6|A0A0D9WLF6_9ORYZ:tr|A0A0E0A4K1|A0A0E0A4K1_9ORYZ</td></tr>
<tr><td>GYVGANPRL</td><td>35.78</td><td>945.5032</td><td>9</td><td>473.7595</td><td>12.69</td><td>Os04g0404400 protein</td><td>tr|Q0JDG9|Q0JDG9_ORYSJ:tr|A0A0P0WA63|A0A0P0WA63_ORYSJ:tr|Q01L47|Q01L47_ORYSA:tr|A0A0D9ZKB8|A0A0D9ZKB8_9ORYZ:tr|A0A0E0H0B3|A0A0E0H0B3_ORYNI:tr|A0A0D3FVG1|A0A0D3FVG1_9ORYZ:tr|A2XT28|A2XT28_ORYSI:tr|Q7X6I8|Q7X6I8_ORYSJ:tr|I1PKX3|I1PKX3_ORYGL:tr|A0A0E0P8P0|A0A0E0P8P0_ORYRU</td></tr>
<tr><td>KGQTPVFPR</td><td>34.53</td><td>1028.5767</td><td>9</td><td>515.2948</td><td>8.15</td><td>ADH1</td><td>tr|A0A0E0MDS3|A0A0E0MDS3_ORYPU:tr|A0A0E0F3I6|A0A0E0F3I6_9ORYZ:tr|D7PPG5|D7PPG5_ORYSJ:tr|Q75ZX2|Q75ZX2_9ORYZ:tr|Q4R1G8|Q4R1G8_ORYRU:tr|B6F2D3|B6F2D3_9ORYZ:tr|B9V0Q8|B9V0Q8_9ORYZ:tr|B6F2D0|B6F2D0_ORYPU:tr|Q75ZX3|Q75ZX3_9ORYZ:Q2R8Z5|ADH1_ORYSJ:tr|B6F2B9|B6F2B9_ORYSI:tr|D7PPI1|D7PPI1_ORYSJ:tr|Q4R1F4|Q4R1F4_ORYRU:tr|Q75ZX1|Q75ZX1_9ORYZ:tr|D7PPK7|D7PPK7_ORYSJ:tr|Q4R1F0|Q4R1F0_ORYRU:Q0ITW7|ADH2_ORYSJ:tr|Q75ZX6|Q75ZX6_ORYRU:tr|B6F2D1|B6F2D1_ORYMI:tr|B9V0R7|B9V0R7_ORYPU:tr|D7PPG9|D7PPG9_ORYRU:tr|Q760C7|Q760C7_ORYRU:tr|B9V0N7|B9V0N7_ORYNI:Q75ZX4|ADH1_ORYSI:Q4R1E8|ADH2_ORYSI:tr|B9V0R8|B9V0R8_ORYPU:tr|B9V0Q7|B9V0Q7_9ORYZ:tr|A0A0E0F3I5|A0A0E0F3I5_9ORYZ:tr|A2ZCK1|A2ZCK1_ORYSI:tr|A0A0E0F3I4|A0A0E0F3I4_9ORYZ:tr|A0A0E0F3I3|A0A0E0F3I3_9ORYZ:tr|A0A0E0BGL8|A0A0E0BGL8_9ORYZ:tr|A0A0E0F3I2|A0A0E0F3I2_9ORYZ:tr|A0A0E0F3I0|A0A0E0F3I0_9ORYZ</td></tr>
<tr><td>HGAFTPR</td><td>33.94</td><td>784.3980</td><td>7</td><td>393.2070</td><td>5.48</td><td>Uncharacterized protein</td><td>tr|A0A0D9VE94|A0A0D9VE94_9ORYZ</td></tr>
<tr><td>VANPKKPF</td><td>33.03</td><td>899.5228</td><td>8</td><td>450.7681</td><td>6.57</td><td>Phosphoglycerate kinase</td><td>tr|A6N1P1|A6N1P1_ORYSI:tr|A0A0E0GWR2|A0A0E0GWR2_ORYNI:tr|A2YG06|A2YG06_ORYSI:tr|A0A0D3GJQ6|A0A0D3GJQ6_9ORYZ:tr|A0A0E0E560|A0A0E0E560_9ORYZ:tr|A0A0E0AD06|A0A0E0AD06_9ORYZ:tr|Q655T1|Q655T1_ORYSJ:tr|A0A0D9WT19|A0A0D9WT19_9ORYZ:tr|A0A0E</td></tr>
</table>

							0Q1D7|A0A0E0Q1D7_ORYRU:tr|J3MGP8|J3MGP8_ORYBR:tr|A0A0E0LEG8|A0A0E0LEG8_ORYPU:tr|I1Q4G7|I1Q4G7_ORYGL:tr|Q09HR1|Q09HR1_ORYSI:tr|A0A0E0HQB3|A0A0E0HQB3_ORYNI:tr|B8AIH2|B8AIH2_ORYSI:tr|A0A0E0CGA6|A0A0E0CGA6_9ORYZ:tr|A0A0E0NAE9|A0A0E0NAE9_ORYRU:tr|Q09HR2|Q09HR2_ORYSI:tr|J3L9Z3|J3L9Z3_ORYBR:tr|A0A0D9YMV6|A0A0D9YMV6_9ORYZ:tr|I1NXN0|I1NXN0_ORYGL:tr|Q6H6C7|Q6H6C7_ORYSJ:tr|A0A0E0CGA5|A0A0E0CGA5_9ORYZ:tr|A0A0D3F185|A0A0D3F185_9ORYZ:tr|A0A0D9VCN8|A0A0D9VCN8_9ORYZ:tr|A0A0E0JW27|A0A0E0JW27_ORYPU
TNPWHSPRQGS	32.91	1265.5901	11	633.8027	6.04	Uncharacterized protein	tr|A0A0D9VE94|A0A0D9VE94_9ORYZ:tr|A2X2Z8|A2X2Z8_ORYSI
HGAFTPRF	32.08	931.4664	8	466.7403	12.66	Uncharacterized protein	tr|A0A0D9VE94|A0A0D9VE94_9ORYZ
VVGTPGRVF	31.45	930.5287	9	466.2720	15.13	Eukaryotic initiation factor 4A-2	P41377|IF4A2_ARATH:tr|A0A0D9WTS3|A0A0D9WTS3_9ORYZ:tr|A0A0D9VC68|A0A0D9VC68_9ORYZ:tr|A0A0N7KMN9|A0A0N7KMN9_ORYSJ:P41376|IF4A1_ARATH:tr|A0A0E0N9T3|A0A0E0N9T3_ORYRU:P35683|IF4A1_ORYSJ:Q6Z2Z4|IF4A3_ORYSJ:tr|J3MHA8|J3MHA8_ORYBR:tr|I1NX84|I1NX84_ORYGL:tr|J3L9J6|J3L9J6_ORYBR:Q9CAI7|IF4A3_ARATH:tr|A0A0E0Q284|A0A0E0Q284_ORYRU:tr|A0A0E0LFA0|A0A0E0LFA0_ORYPU:tr|I1Q4Z1|I1Q4Z1_ORYGL:tr|A2X0V4|A2X0V4_ORYSI:tr|A0A0D9YM91|A0A0D9YM91_9ORYZ:tr|A0A0E0JVG9|A0A0E0JVG9_ORYPU:tr|A0A0D3GKG6|A0A0D3GKG6_9ORYZ:tr|A0A0E0G162|A0A0E0G162_ORYNI:tr|A0A0E0CFK9|A0A0E0CFK9_9ORYZ:tr|A0A0E0ADY5|A0A0E0ADY5_9ORYZ:tr|A0A0E0HUY2|A0A0E0HUY2_ORYNI:tr|A0A0E0HUY1|A0A0E0HUY1_ORYNI:tr|A2YGP5|A2YGP5_ORYSI:tr|A0A0D3F0N3|A0A0D3F0N3_9ORYZ:tr|A0A0E0HUY0|A0A0E0HUY0_ORYNI:tr|A0A0D3F0N2|A0A0D3F0N2_9ORYZ
H(+26.02)GAFTPR	30.35	810.4136	7	406.2147	7.27	Uncharacterized protein	tr|A0A0D9VE94|A0A0D9VE94_9ORYZ
HS(-18.01)AFTPR	30.20	796.3980	7	399.2061	5.66	Uncharacterized protein	tr|A2X2Z8|A2X2Z8_ORYSI
RNNQVWQQL	29.65	1184.6051	9	593.3105	16.99	Os05g0328333 protein	tr|A0A0P0WKT5|A0A0P0WKT5_ORYSJ:tr|Q5W6A6|Q5W6A6_ORYSJ:tr|E5D3L5|E5D3L5_ORYSJ:tr|Q40730|Q40730_ORYSA:tr|E5D3L6|E5D3L6_ORYSJ:tr|C7J346|C7J346_ORYSJ:tr|A0A0P0WKY1|A0A0P0WKY1_ORYSJ:tr|A0A0P0WKV9|A0A0P0WKV9_ORYSJ:tr|Q5W6A1|Q5W6A1_ORYSJ:tr|A0A0D3G667|A0A0D3G667_9ORYZ:tr|Q5W743|Q5W743_ORYSJ:tr|Q5W695|Q5W695_ORYSJ:tr|Q43603|Q43603_ORYSA:tr|P93412|P93412_

<table>
<tr><td></td><td></td><td></td><td></td><td></td><td></td><td></td><td>ORYSJ:tr|Q0DJ44|Q0DJ44_ORYSJ:tr|A0A0P0WKT6|A0A0P0WKT6_ORYSJ:tr|E0X6Y1|E0X6Y1_ORYSJ:tr|Q5W755|Q5W755_ORYSJ:P0C5E5|PRO7_ORYSI:tr|A1YQE8|A1YQE8_ORYSJ:tr|Q5W6A3|Q5W6A3_ORYSJ:tr|A0A0E0PKU8|A0A0E0PKU8_ORYRU:Q0DJ45|PRO7_ORYSJ:tr|A0A0E0DQ09|A0A0E0DQ09_9ORYZ:tr|A0A0E0PKV4|A0A0E0PKV4_ORYRU:tr|A0A0N7KKJ8|A0A0N7KKJ8_ORYSJ:tr|Q0DJ38|Q0DJ38_ORYSJ:tr|A1YQF0|A1YQF0_ORYSJ:tr|A0A0D3G661|A0A0D3G661_9ORYZ</td></tr>
<tr><td>FNNRPNSF</td><td>29.33</td><td>994.4620</td><td>8</td><td>498.2391</td><td>9.53</td><td>Starch branching enzyme 1</td><td>tr|A0A0E0E6K5|A0A0E0E6K5_9ORYZ:Q01401-2|GLGB_ORYSJ:tr|B7EAH2|B7EAH2_ORYSJ:tr|A0A2S0T044|A0A2S0T044_ORYSA:tr|A0A2S0T039|A0A2S0T039_ORYSA:tr|A0A0E0AEM0|A0A0E0AEM0_9ORYZ:tr|A0A0E0AEL9|A0A0E0AEL9_9ORYZ:tr|A0A2S0T029|A0A2S0T029_ORYSA:tr|Q0D9D0|Q0D9D0_ORYSJ:Q01401|GLGB_ORYSJ:tr|A0A2S0T020|A0A2S0T020_ORYSA:tr|D0TZI4|D0TZI4_ORYSI:tr|B8B2L2|B8B2L2_ORYSI:tr|A0A0E0E6K3|A0A0E0E6K3_9ORYZ:tr|A0A0E0LFU9|A0A0E0LFU9_ORYPU:tr|A0A0E0LFV0|A0A0E0LFV0_ORYPU:tr|A0A0E0LFU8|A0A0E0LFU8_ORYPU:tr|A0A0E0LFU7|A0A0E0LFU7_ORYPU:tr|A0A0E0Q2U7|A0A0E0Q2U7_ORYRU:tr|A0A0E0Q2U8|A0A0E0Q2U8_ORYRU:tr|A0A0E0Q2U9|A0A0E0Q2U9_ORYRU</td></tr>
<tr><td>RLPVPRPGQ</td><td>29.33</td><td>1018.6036</td><td>9</td><td>510.3096</td><td>8.72</td><td>Os11g0546000 protein</td><td>tr|A2ZF62|A2ZF62_ORYSI:tr|A0A0P0Y344|A0A0P0Y344_ORYSJ:tr|J3N8W8|J3N8W8_ORYBR:tr|Q8L8I4|Q8L8I4_ORYSJ:tr|Q2R2X2|Q2R2X2_ORYSJ:tr|I1NZF9|I1NZF9_ORYGL:tr|Q6K3B8|Q6K3B8_ORYSJ:tr|A3CC60|A3CC60_ORYSJ:tr|B8AFM4|B8AFM4_ORYSI</td></tr>
<tr><td>H(+68.06)GAFTPR</td><td>28.29</td><td>852.4606</td><td>7</td><td>427.2376</td><td>14.13</td><td>Uncharacterized protein</td><td>tr|A0A0D9VE94|A0A0D9VE94_9ORYZ</td></tr>
<tr><td>WIDFPRAPQ</td><td>28.26</td><td>1128.5715</td><td>9</td><td>565.2932</td><td>21.73</td><td>Os02g0528200 protein</td><td>tr|A0A0N7KFE7|A0A0N7KFE7_ORYSJ:tr|A0A0D3F5W2|A0A0D3F5W2_9ORYZ:tr|A0A0E0K0K5|A0A0E0K0K5_ORYPU:tr|A0A0E0G7M7|A0A0E0G7M7_ORYNI:tr|A0A0E0NFS6|A0A0E0NFS6_ORYRU:tr|A0A0D9YT34|A0A0D9YT34_9ORYZ:tr|A0A0E0K0K4|A0A0E0K0K4_ORYPU:tr|A0A0E0CLF1|A0A0E0CLF1_9ORYZ:tr|Q6H6P8|Q6H6P8_ORYSJ:tr|Q40663|Q40663_ORYSA:tr|A2X5K0|A2X5K0_ORYSI:tr|D0TZK1|D0TZK1_ORYSI:tr|I1P0X2|I1P0X2_ORYGL:tr|B3VDJ4|B3VDJ4_ORYSJ:tr|I6VRB8|I6VRB8_ORYSJ:tr|A0A0D9VGL1|A0A0D9VGL1_9ORYZ</td></tr>
<tr><td>H(+40.03)GAFTPR</td><td>28.06</td><td>824.4293</td><td>7</td><td>413.2221</td><td>7.91</td><td>Uncharacterized protein</td><td>tr|A0A0D9VE94|A0A0D9VE94_9ORYZ</td></tr>
<tr><td>IDFPRAPQ</td><td>27.69</td><td>942.4922</td><td>8</td><td>472.2540</td><td>15.83</td><td>Os02g0528200 protein</td><td>tr|A0A0N7KFE7|A0A0N7KFE7_ORYSJ:tr|A0A0D3F5W2|A0A0D3F5W2_9ORYZ:tr|A0A0E0K0K5|A0A0E0K0K5_ORYPU:tr|A0A0E0G7M7|A0A0E0G7M7_ORYNI:tr|A0A0E0NFS6|A0A0E0NFS6_ORYRU:tr|A0A0D9YT34|A0A0D9YT34_9ORYZ:tr|A0A0E0K0K4|A0A0E0K0K4_ORYPU:tr|A0A0E0CLF1|A0A0E0CLF1_9ORYZ:tr|Q6H6P8|Q6H6P8_ORYSJ:tr|Q40663|Q40663_ORYSA:tr|A2X5K0|A2X5K0_ORYSI:tr|D0TZK1|D0TZK1_ORYSI:tr|I1P0X2</td></tr>
</table>

							|I1P0X2_ORYGL:tr|B3VDJ4|B3VDJ4_ORYSJ:tr|I6VRB8|I6VRB8_ORYSJ:tr|A0A0D9VGL1|A0A0D9VGL1_9ORYZ
NKPDWFLK	26.79	1046.5549	8	524.2847	16.07	Uncharacterized protein	tr|A0A0E0H8N7|A0A0E0H8N7_ORYNI
SRPDFRF	26.27	923.4613	7	462.7386	13.65	Os10g0189100 protein	tr|A0A0E0EWF3|A0A0E0EWF3_9ORYZ:tr|A0A0E0EWF5|A0A0E0EWF5_9ORYZ:tr|A0A0E0EWF4|A0A0E0EWF4_9ORYZ:tr|J3N1F5|J3N1F5_ORYBR:tr|A0A0E0IQA4|A0A0E0IQA4_ORYNI:tr|A0A0D3HBW4|A0A0D3HBW4_9ORYZ:tr|A0A0D9XIC3|A0A0D9XIC3_9ORYZ:tr|B8BG13|B8BG13_ORYSI:tr|A0A0E0QX42|A0A0E0QX42_ORYRU:tr|A0A0E0B8J4|A0A0E0B8J4_9ORYZ:tr|Q33AE4|Q33AE4_ORYSJ:tr|I1QT43|I1QT43_ORYGL:tr|Q53QR8|Q53QR8_ORYSJ:tr|A0A0E0M659|A0A0E0M659_ORYPU
H(+154.10)GAFTPR	26.25	938.4974	7	470.2562	16.82	Uncharacterized protein	tr|A0A0D9VE94|A0A0D9VE94_9ORYZ
LGYPRTPR	24.61	958.5348	8	480.2737	6.87	Allergenic protein	tr|Q40719|Q40719_ORYSJ:tr|Q40718|Q40718_ORYSJ:tr|Q40720|Q40720_ORYSJ:tr|A0A0E0E948|A0A0E0E948_9ORYZ:tr|A0A0D3GNN6|A0A0D3GNN6_9ORYZ:tr|A3BHT2|A3BHT2_ORYSJ:tr|A2YJF9|A2YJF9_ORYSI:tr|A0A0E0HY52|A0A0E0HY52_ORYNI:Q01883|RAG1_ORYSJ:tr|A2YJG1|A2YJG1_ORYSI:Q01882|RAG2_ORYSJ:tr|B7SDG9|B7SDG9_ORYSJ:tr|I1Q920|I1Q920_ORYGL:tr|A0A0E0AHA3|A0A0E0AHA3_9ORYZ:tr|A0A0E0HY53|A0A0E0HY53_ORYNI:tr|A0A0E0AHA2|A0A0E0AHA2_9ORYZ:tr|A0A0E0Q5M4|A0A0E0Q5M4_ORYRU:tr|A0A0D3GNN7|A0A0D3GNN7_9ORYZ:tr|A0A0E0E950|A0A0E0E950_9ORYZ:tr|A0A0E0LII4|A0A0E0LII4_ORYPU
TGKSPYF	24.59	798.3912	7	400.2043	11.33	Globulin 2	tr|O65043|O65043_ORYSA:tr|Q9ZRH1|Q9ZRH1_ORYSA:tr|Q9ZRH0|Q9ZRH0_ORYSA:tr|A0A0D3FQ29|A0A0D3FQ29_9ORYZ:tr|A3ANJ6|A3ANJ6_ORYSJ:B8AL97|CUCIN_ORYSI:tr|A0A0E0P2F5|A0A0E0P2F5_ORYRU:tr|A0A0E0GUU5|A0A0E0GUU5_ORYNI:Q852L2|CUCIN_ORYSJ:tr|A0A0E0GUU4|A0A0E0GUU4_ORYNI
YVGANPRLL	24.17	1001.5658	9	501.7905	16.74	Os04g0404400 protein	tr|Q0JDG9|Q0JDG9_ORYSJ:tr|A0A0P0WA63|A0A0P0WA63_ORYSJ:tr|Q01L47|Q01L47_ORYSA:tr|A0A0D9ZKB8|A0A0D9ZKB8_9ORYZ:tr|A0A0E0H0B3|A0A0E0H0B3_ORYNI:tr|A0A0D3FVG1|A0A0D3FVG1_9ORYZ:tr|A2XT28|A2XT28_ORYSI:tr|Q7X6I8|Q7X6I8_ORYSJ:tr|I1PKX3|I1PKX3_ORYGL:tr|A0A0E0P8P0|A0A0E0P8P0_ORYRU
KKPVPDFS	23.98	916.5018	8	459.2580	7.72	Glucose-1-phosphate adenylyltransferase	tr|A0A0E0LZH2|A0A0E0LZH2_ORYPU:tr|D0TZC6|D0TZC6_ORYSJ:tr|A0A0E0EQI0|A0A0E0EQI0_9ORYZ:tr|B8BE16|B8BE16_ORYSI:tr|A0A0E0B114|A0A0E0B114_9ORYZ:tr|A0A0D3H4W0|A0A0D3H4W0_9ORYZ:tr|A7IZE4|A7IZE4_ORYSI:tr|D4AIA3|D4AIA3_ORYSI:tr|B7EVB8|B7EVB8_ORYSJ:tr|A0A0D3GZB2|A0A0D3GZB2_9ORYZ:tr|A0A0E0H7V9|A0A0E0H7V9_ORYNI:tr|A0A0E0QHR8|A0A0E0QHR8_ORYRU:tr|B8XED8|B8XED8_O

<table>
<tr><td></td><td></td><td></td><td></td><td></td><td></td><td></td><td>RYSA:tr|B8XED9|B8XED9_ORYSI:tr|B8XED7|B8XED7_ORYSI:tr|B8XEE8|B8XEE8_ORYSA:tr|B8XEF0|B8XEF0_ORYSI:tr|B8XEF2|B8XEF2_ORYSI:tr|B8XEE9|B8XEE9_ORYSI:tr|B8XEE1|B8XEE1_ORYSI:tr|B8XEE2|B8XEE2_ORYSJ:tr|Q9ARH9|Q9ARH9_ORYSA:tr|D3U2H7|D3U2H7_ORYSA:tr|D0TZC9|D0TZC9_ORYSI:Q69T99|GLGS1_ORYSJ:tr|B8XEE5|B8XEE5_ORYSJ:tr|B8XEE6|B8XEE6_ORYSA:tr|A2YU91|A2YU91_ORYSI:tr|I1QHZ2|I1QHZ2_ORYGL:tr|A0A0D3GZB1|A0A0D3GZB1_9ORYZ:tr|A0A0E0QHR7|A0A0E0QHR7_ORYRU</td></tr>
<tr><td>HGAFTPRL(sub F)</td><td>23.86</td><td>897.4821</td><td>8</td><td>449.7492</td><td>11.83</td><td>Uncharacterized protein</td><td>tr|A0A0D9VE94|A0A0D9VE94_9ORYZ</td></tr>
<tr><td>FDWVGGR</td><td>22.81</td><td>835.3976</td><td>7</td><td>418.7066</td><td>16.93</td><td>Glucose-6-phosphate isomerase</td><td>tr|Q84P59|Q84P59_ORYSJ:tr|A0A0D3H7U8|A0A0D3H7U8_9ORYZ:tr|A0A0E0IKZ3|A0A0E0IKZ3_ORYNI:tr|A0A0E0B412|A0A0E0B412_9ORYZ:tr|A0A0D9X8Y6|A0A0D9X8Y6_9ORYZ:tr|A0A0D9XEL8|A0A0D9XEL8_9ORYZ:tr|Q6ZB94|Q6ZB94_ORYSJ:tr|A3BU52|A3BU52_ORYSJ:tr|A0A0E0M265|A0A0E0M265_ORYPU:tr|I1QJH8|I1QJH8_ORYGL:tr|J3MY99|J3MY99_ORYBR:tr|A0A0D3H1D9|A0A0D3H1D9_9ORYZ:tr|A0A0E0AWN3|A0A0E0AWN3_9ORYZ:tr|A0A0E0IDE9|A0A0E0IDE9_ORYNI:tr|Q76E42|Q76E42_ORYSJ:tr|Q6YXI1|Q6YXI1_ORYSJ:tr|A0A0D9X8Y5|A0A0D9X8Y5_9ORYZ:tr|A0A0E0ET12|A0A0E0ET12_9ORYZ:tr|J3MTV1|J3MTV1_ORYBR</td></tr>
<tr><td>LGGDGGGGGGGGPLP</td><td>21.98</td><td>1123.5258</td><td>15</td><td>562.7701</td><td>14.73</td><td>Cellulose synthase-like protein D3</td><td>Q7EZW6|CSLD3_ORYSJ:tr|A0A0P0XES5|A0A0P0XES5_ORYSJ:tr|C7J5V0|C7J5V0_ORYSJ:tr|A0A0E0H7V4|A0A0E0H7V4_ORYNI</td></tr>
<tr><td>VRPRPGVWE</td><td>21.74</td><td>1094.5985</td><td>9</td><td>548.3068</td><td>9.41</td><td>Sucrose synthase</td><td>tr|Q10LP3|Q10LP3_ORYSJ:tr|I1PB61|I1PB61_ORYGL:tr|A0A0D3FIA2|A0A0D3FIA2_9ORYZ:tr|A0A0E0GM66|A0A0E0GM66_ORYNI:tr|A0A0D9Z732|A0A0D9Z732_9ORYZ:tr|A0A0E0D095|A0A0E0D095_9ORYZ:tr|A0A0E0NUU1|A0A0E0NUU1_ORYRU:Q10LP5|SUS4_ORYSJ:tr|K4FDV3|K4FDV3_ORYSJ:tr|A0A0E0KDI7|A0A0E0KDI7_ORYPU:tr|B8APD5|B8APD5_ORYSI:tr|A0A0D9VTU5|A0A0D9VTU5_9ORYZ:tr|J3LNM1|J3LNM1_ORYBR</td></tr>
<tr><td>HGAFTPRV(sub F)</td><td>21.15</td><td>883.4664</td><td>8</td><td>442.7405</td><td>8.61</td><td>Uncharacterized protein</td><td>tr|A0A0D9VE94|A0A0D9VE94_9ORYZ</td></tr>
<tr><td>RRVVVIEPR</td><td>21.01</td><td>1122.6985</td><td>9</td><td>375.2407</td><td>5.41</td><td>cDNA clone:J023074I17, full insert sequence</td><td>tr|I1P695|I1P695_ORYGL:tr|B7F6S9|B7F6S9_ORYSJ:tr|Q0JDI6|Q0JDI6_ORYSJ:tr|A0A0E0DDI4|A0A0E0DDI4_9ORYZ:tr|A0A0E0DDI3|A0A0E0DDI3_9ORYZ:tr|A0A0E0H077|A0A0E0H077_ORYNI:tr|A2XT06|A2XT06_ORYSI:tr|A0A0E0P8L3|A0A0E0P8L3_ORYRU:tr|A0A0D3FVE0|A0A0D3FVE0_9ORYZ:tr|A0A0D9ZK91|A0A0D9ZK91_9ORYZ:tr|I1PKV4|I1PKV4_ORYGL:tr|Q01LG9|Q01LG9_ORYSA:tr|Q7XV93|Q7XV93_ORYSJ</td></tr>
<tr><td>LVPRYSNTPGM</td><td>20.90</td><td>1233.6176</td><td>11</td><td>617.8156</td><td>14.62</td><td>Glutelin</td><td>tr|A2X399|A2X399_ORYSI:P14614|GLUB4_ORYSJ:tr|Q0E261|Q0E261_ORYSJ:tr|A0A0D9YQ79|A0A0D9YQ79_9ORYZ:tr|D6BV14|D6BV14_ORY</td></tr>
</table>

							SJ:tr|A0A0D3F3E6|A0A0D3F3E6_9ORYZ:tr|A0A0E0JY90|A0A0E0JY90_ORYPU:Q6ERU3|GLUB5_ORYSJ:tr|I1NZ94|I1NZ94_ORYGL:tr|A0A0D3F3E3|A0A0D3F3E3_9ORYZ:tr|A0A0E0JY02|A0A0E0JY02_ORYPU
TNPWHSPRQGSR	20.29	1421.6912	12	711.8474	7.19	Uncharacterized protein	tr|A0A0D9VE94|A0A0D9VE94_9ORYZ
HGALLRSM	20.17	883.4698	8	442.7405	8.61	MFS transporter	tr|A0A3N4TK14|A0A3N4TK14_9ACTN
